# Clinical metabolomics in type 2 diabetes mellitus: from pathogenesis to biomarkers

**DOI:** 10.3389/fendo.2025.1501305

**Published:** 2025-02-25

**Authors:** Chuanxin Liu, Hetao Chen, Yujin Ma, Lei Zhang, Lulu Chen, Jiarui Huang, Zizhe Zhao, Hongwei Jiang, Jiao Kong

**Affiliations:** ^1^ Luoyang Key Laboratory of Clinical Multiomics and Translational Medicine, Henan Key Laboratory of Rare Diseases, Endocrinology and Metabolism Center, The First Affiliated Hospital, and College of Clinical Medicine of Henan University of Science and Technology, Luoyang, China; ^2^ Department of Clinical Laboratory, The First Affiliated Hospital, College of Clinical Medicine of Henan University of Science and Technology, Luoyang, China; ^3^ Department of Integrative Medicine, The First Affiliated Hospital, and College of Clinical Medicine of Henan University of Science and Technology, Luoyang, China; ^4^ Department of Critical Care Medicine, The First Affiliated Hospital, and College of Clinical Medicine of Henan University of Science and Technology, Luoyang, China; ^5^ Institute of Drug Metabolism and Pharmaceutical Analysis, College of Pharmaceutical Sciences, Zhejiang University, Hangzhou, China

**Keywords:** clinical metabolomics, type 2 diabetes mellitus (T2DM), complications, biomarkers, stratification

## Abstract

As a multidimensional metabolic disorder, the disability and death rate of type 2 diabetes mellitus (T2DM) has increased over time. T2DM covers a wide range of pathological manifestations ranging from hyperglycemia to multi-organ failure, and it has the potential to evolve into acute complications, including ketosis and chronic complications such as peripheral neuropathy, retinopathy, and nephropathy. T2DM mainly occurs in microvascular and large vessels and thus it is restricted for the clinician to diagnose and prescribe. However, the pathological mechanism and clinical diagnosis are inadequate. High-throughput metabolomics, characterized by non-invasive diagnostic techniques to identify potential biomarkers and distinct stages of T2DM, has been increasingly recognized as a vigorous tool with latent capacity for clinical translation. The pathological stratification of T2DM can significantly reduce disability and mortality rates. By tracing the metabolome and associated pathways from impaired fasting blood glucose or impaired glucose tolerance to severe organ failure, the chief contributions of large, independent population-based cohorts are summarized herein. These results facilitate understanding the pathophysiology and mechanism and supports research in accurate diagnosis, risk prediction, curative effect, distinct stages, and prognosis judgment of T2DM.

## 1 Introduction

Type 2 diabetes mellitus (T2DM) is one of the most common endocrine and metabolic disorders characterized by chronic hyperglycemia, which can lead to acute and chronic complications related to microvascular (i.e., neuropathy, nephropathy, and retinopathy) and macrovascular (atherosclerosis-related vascular disease, coronary heart disease, cerebrovascular disease, and peripheral vascular disease) (1, 2). Common complications in T2DM include Type 2 diabetic retinopathy (T2DR), Type 2 diabetic retinopathy (T2DR), Type 2 diabetic peripheral neuropathy(T2DPN), and Type 2 diabetic ketosis (T2DK) (3, 4). Such diseases pose a huge economic burden (5). Prediabetes mellitus (PM) refers to the transition stage of normal glucose metabolism into T2DM. This stage is in a healthy state, and blood sugar can be recalled through exercise and diet regulation. There are multiple risks due to abnormal blood glucose, represented by diabetic macrovascular disease and microvascular disease, which do not fulfill the diagnostic criteria for diabetes, namely, two clinical conditions: impaired fasting blood glucose (IFG) and impaired glucose tolerance (IGT).

The incidence of diabetes is mainly related to genetic inheritance and environmental factors. According to the 10th edition of the International Diabetes Federation, the number of diabetes patients worldwide is estimated to increase to 783.2 million by 2045 (6). It is widely recognized that approximately one-third of these patients will develop at least one complication within about 10 years after the onset of diabetes (7). T2DM accounts for 90%-95% of all diabetes cases (8). T2DM is a chronic disease characterized by two primary pathophysiological mechanisms: ① a reduction in the mass and function of pancreatic β cells, ranging from 20% to 65%, which leads to impaired insulin secretion; ② insulin resistance, where cells in muscles, fat, and liver tissues fail to respond adequately to insulin (9). Consequently, higher levels of insulin are required to maintain normal blood glucose concentrations by inhibiting hepatic glucose production and promoting glucose uptake in muscle and adipose tissues. Prolonged exposure to elevated levels of circulating insulin leads to the development of insulin resistance in peripheral tissues, and over time, the pancreas fails to produce sufficient insulin to overcome this cellular resistance (10). However, due to the long latent period and absence of obvious symptoms initially, reversing T2DM with drug intervention is difficult after the symptoms are exposed or clinically confirmed in light of clear diagnostic criteria. According to the literature, the pathogenesis and process of metabolic syndromes such as diabetes and its complications are mainly reflected in the metabolite network, and the mechanism changes at the gene level are also found in the network. Studies have shown that some related metabolites in patients with diabetes have changed before the occurrence of obvious organic damage (11). Therefore, it is necessary to scientifically prevent T2DM in the early stages of disease onset. Fortunately, clinical metabolomics were employed to understand the progression pathologies of T2DM and its corresponding complications in detail (12). Studies have demonstrated that metabolomic analysis enables the exploration of metabolic disorders associated with T2DM, thereby deepening our understanding of disease progression (13, 14). This approach has the potential to facilitate novel clinical diagnoses and the development of effective treatment strategies. Moreover, identifying specific metabolites may provide promising biomarkers for the early prediction, prevention, and management of hyperglycemia and its complications (15). In recent years, excellent progress has been made in the study of T2DM and its complications through High throughput sequencing method, i.e., a discipline specifically focused on metabolic small molecules.

Clinical metabolomics is a type of systems biology research closely linked to phenotype. Clinical metabolomics is based on clinical cohorts, with metabolomics as a tool, and supplemented by bioinformatics techniques to globally analyze the homeostasis imbalance of endogenous metabolites under external stimulation and the mechanism of prognosis after treatment intervention. This technology reveals the potential biomarkers and targets of disease, widely used in cardio-cerebrovascular research in the fields of, for example, toxicity study, malignant tumors, diabetes, and neurodegenerative diseases (16–21). For instance, Suhre et al. (22) analyzed serum samples from 2820 subjects by ultra-performance liquid chromatography-tandem mass spectrometry (UPLC-MS) and obtained 295 metabolites and 37 related gene loci in 60 biochemical pathways. This report provides a new perspective for the study of cardiovascular disease, kidney disease, diabetes, and tumors. Clinical metabolomics is characterized by its advantages: it is non-invasive and low cost and has high throughput, providing strong technical support for type 2 diabetes and its complications. In addition, the China National Academy of Sciences Dalian Physical Chemistry Institute Xu Guowang Group (23) established a fingerprint of a body fluid metabolism that can be used to identify type 2 diabetes, based on liquid chromatography-electrospray ionization linear ion trap mass spectrometry and screened four phospholipid molecules that can be used as biomarkers. The application of metabolomics in clinical sample cohort studies of type 2 diabetes and its complications can help with exploring the physiological and pathological mechanisms and the law of metabolite variation. Early diagnostic indicators such as glucose are expected to have broad application prospects and development space. Despite the promising advancements, several challenges need to be addressed to effectively integrate metabolomics into the clinical management of diabetes and its complications. The metabolome is sensitive to a variety of genetic and environmental stimuli and susceptible to genetic, environmental, and gut microbiome pressures, so subtle differences between individuals can lead to large perturbations in metabolite concentrations and fluxes (15, 24). At present, cystatin C has become an ideal endogenous marker for evaluating glomerular filtration function because it is not affected by sex, age or muscle mass (25). In addition, more and more evidence shows that serum CysC is involved in the pathological process of vascular remodeling and neovascularization, which is closely related to the occurrence and development of diabetic microangiopathy (26).

Eighty-four papers were included in this review and obtained through database searches, namely, PubMed, Cochrane Library, China national knowledge internet(CNKI), General Purpose, and VIP Database. The keywords for the searches were “metabolomics” and “type 2 diabetes mellitus” and its complications. The papers were incorporated by reading and summarizing the literature according to the classification standards (27). The profound analysis of clinical differential metabolites identified in type 2 diabetes and its complications were conducted concerning composition, frequency of category, sample type, and pathways to explore the pathological mechanism of type 2 diabetes and its complications to provide a systematic basis for clinical diagnosis, risk stratification, comprehending disease progression, prognosis assessment, and drug efficacy. Our goal is to apply metabolomics to clinical diagnostic biomarkers, metabolic mechanisms, and prognostic observations, and early diagnosis can be made through metabolites to avoid progression to more serious complications.

## 2 Profiles of literature retrieval and clinical characteristics

In this review, we applied the strategy of “meta-analysis” to search for all literature included in diabetes and clinical metabolomics to interpret related biomarkers. Other details in the literature were extracted by intensive reading.

### 2.1 Search and study identification

#### 2.1.1 Literature retrieval strategy

To retrieve and include papers, we completed five separate and sequential literature searches using PubMed (http://www.ncbi.nlm.nih.gov/pubmed), the Cochrane Library (https://www.cochrane.org/welcome), China National Knowledge Infrastructure Database (https://www.cnki.net/), WangFang Database (http://www.wanfangdata.com.cn/index.html), and VIP Database (http://www.cqvip.com/). The aim of the first search was to find all related free words (synonyms) according to the subject words. Next, we used the search term “#1 OR #2 OR #3 AND *1 OR *2 OR*3,” “#MeSH AND *MeSH” (#: Subject words and free words of type 2 diabetes and its complications;* Subject words and free words in metabolomics). The retrieval time of the literature was set from January 1, 2000, to January 1, 2020, and the clinical research depended on the official publication time of the literature. The specific retrieval strategies are provided in **Supplementary Method S1**.

#### 2.1.2 Data extraction

The initial literature screening process was conducted by reviewing the titles and abstracts. Subsequently, full-text versions of potential articles were obtained for further assessment. Next, data were extracted following the pre-designed form, including the title, name of the first author and corresponding author, publication institution, publication year, patient characteristics (sample size, sex, age, patient’s baseline, and sample category), study design, and domains of risk of bias. Finally, biomarkers for the early diagnosis of type 2 diabetes and its complications were identified.

#### 2.1.3 Literature quality assessment

The Cochrane Collaboration risk of bias tool was used to evaluate the baseline indicators of the eligibility of the included studies. The following items were assessed: 1) sample size of each group, 2) age, and 3) sex. If the baseline basic indicators of included research are complete and there is no statistical difference, the quality of literature can be rated as “low risk” (indicating low bias risk). And the quality of literature can be rated as “high risk” (high bias risk) if any one of the three items with a statistical difference, and it can be rated as “unclear” if the data are incomplete.

Moreover, the statistical differences in age, sex, and baseline characteristics of the study results are presented in **Supplementary Table S1**, and they were given a degree of bias assessment because there were different baseline characterizations in each case. When the statistical result of baseline was *p* > 0.05, it was defined as “ low risk;” *p*<0.05 was defined as “high risk.” The absence of baseline results was defined as “uncertain”.

### 2.2 Literature search results and baseline characterization

**Figure 1:**
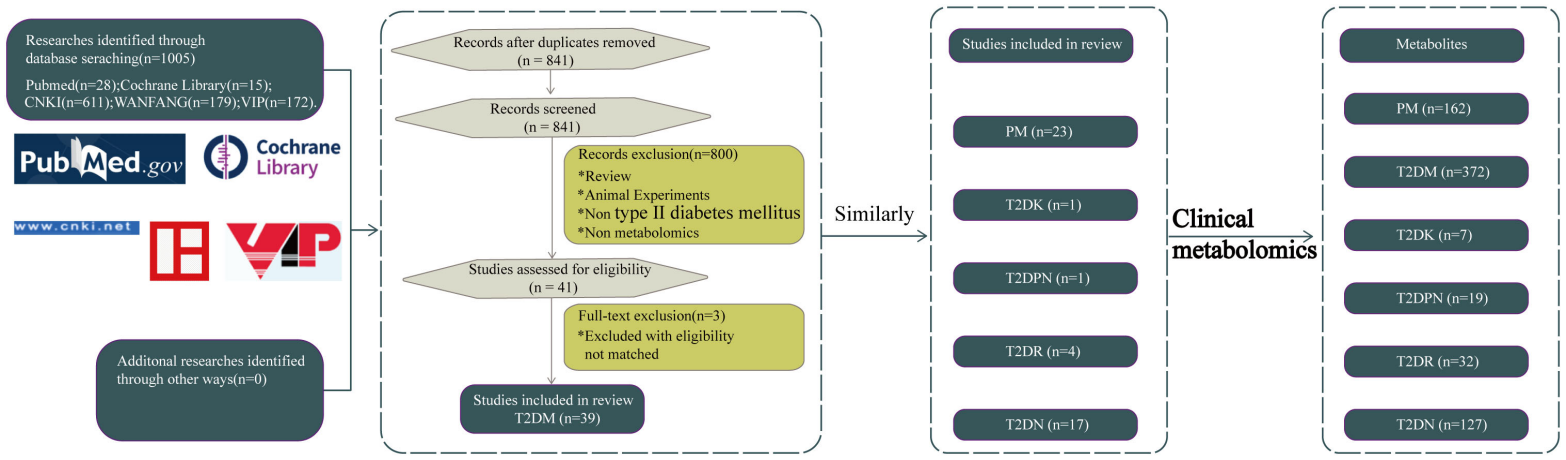
Identification of different metabolites of T2DM and its complications based on clinical metabolomics.

All literature records were identified from the five databases. The duplications were removed, and some records were excluded through screening titles and abstracts because they were irrelevant studies such as reviews and animal experiments. The full texts of the remaining records were screened, and the remaining records were excluded for eligibility of the abovementioned exclusion criteria. The remaining studies were ultimately included in this review. All the studies were published between 2000 and 2020. The process and results of the literature screening are shown in **Figure 1**. There are 23 pieces of literature on type 2 PM, 39 on T2DM, 1 on T2DK, 1 on type 2 diabetic peripheral neuropathy, 4 on T2DR, and 17 on T2DN.

## 3 Pathogenesis

**Figure 2:**
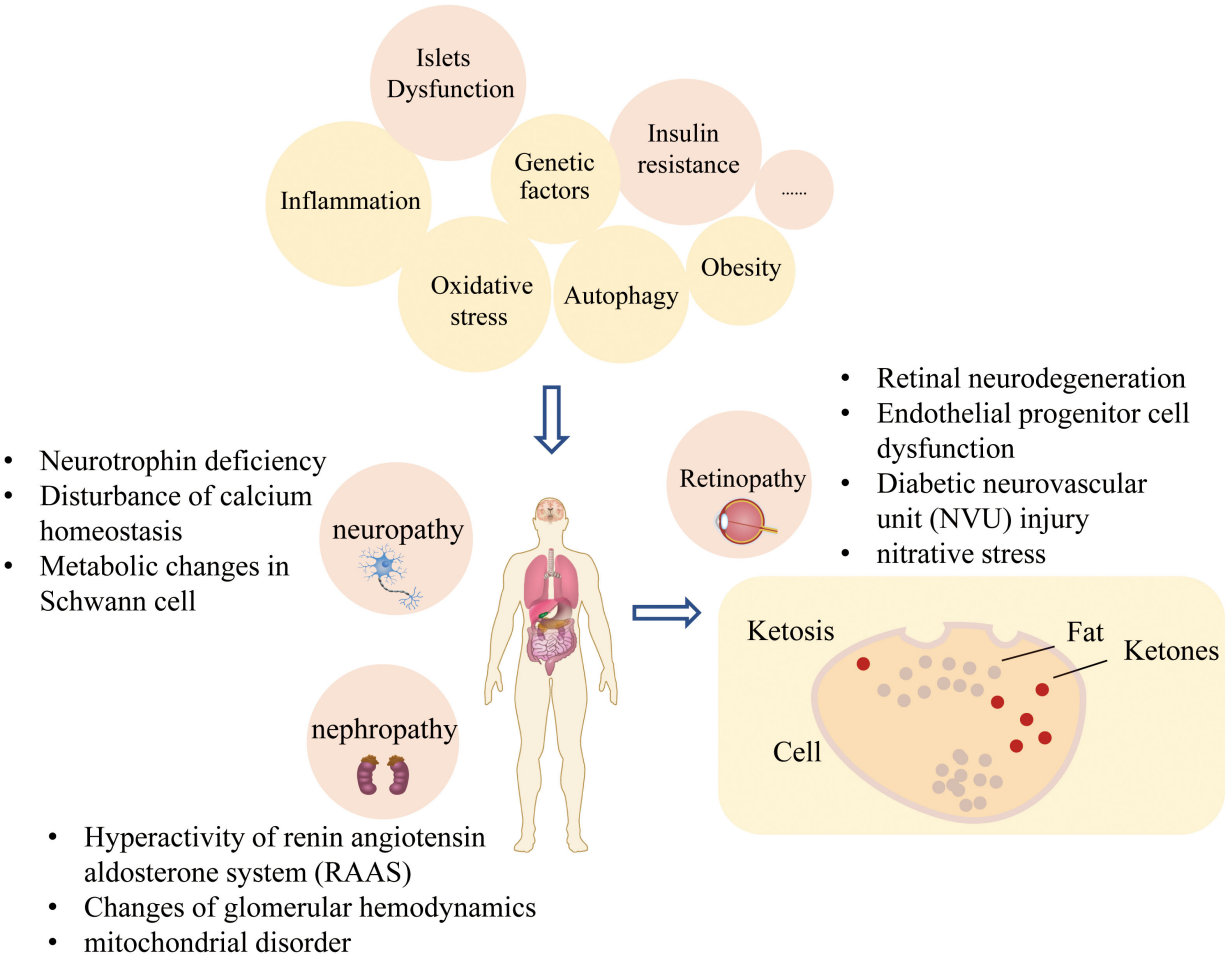
Pathophysiological factors of diabetes and its complications.

The pathogenesis of T2DM and its complications are complex and unclear, but the following mechanisms have been proven closely related to the onset of T2DM and its complications (**Figure 2**). Many types of pathogenesis and adverse consequences are induced by the state of high glucose.

### 3.1 Common pathogenesis

#### 3.1.1 Genetic factors

There are two crucial pathophysiological bases of T2DM, including β cell dysfunction and insulin resistance, and genetic factors play an indispensable role in its development. T2DM is related to the angiotensin-converting enzyme (ACE) gene, which regulates the angiotensin system. Among this process, the AGE receptor, AR, and ApoE genes are implicated in abnormal lipid metabolism. The HP gene is associated with oxidative stress response, and the CCR5 gene, VEGFA gene, and EPO gene are related to the increased secretion of various inflammatory factors.

Under the action of polygenes, kidney damage is caused in some patients with diabetes. In addition, overexpression of miRNA in diabetic nephropathy (DN) leads to proteinuria and hyperglycemia in patients with DN, leading to the occurrence and development of the disease (28).

#### 3.1.2 Obesity

Approximately 80% of patients with T2DM are obese, and mainly central obesity is observed, characterized by abnormal glucose and lipid metabolism. Central obesity is defined by the World Health Organization as a waist circumference exceeding 94 cm for males and 80 cm for females. The International Diabetes Federation (IDF) has proposed different cut-off points for various ethnic groups, such as 94 cm for males and 80 cm for females for Europeans, and 90 cm for males and 80 cm for females for Asians (29). Abdominal fat metabolism is more active than hip and thigh fat metabolism. There was a significant negative correlation between the area of intra-abdominal fat and insulin-mediated glucose utilization. In patients with central obesity, the amount of intra-abdominal fat increased, and the inhibitory effect of insulin on hepatic glycogen production weakened. Free fatty acids in the blood affect blood glucose levels in two ways, leading to the onset of T2DM. On the one hand, excessive free fatty acids lead to ectopic deposition of fatty acids, and the function of insulin secretion in β cells is impaired. On the other hand, there are excessive free fatty acids in the blood, which increase the output of liver glycogen and hinder the clearance of glucose through via a free fatty acid substrate competition mechanism (30). The elevation of plasma free fatty acids can inhibit glucose oxidation, impede glucose uptake by cells, suppress muscle glycogen synthesis, and promote gluconeogenesis, ultimately leading to glucose metabolic disorders. Furthermore, the inhibition of cellular insulin receptor tyrosine kinase activity and reduced expression and activity of insulin receptor substrates exacerbate these metabolic disturbances, thereby aggravating insulin resistance (30).

#### 3.1.3 Inflammation

Inflammation is a crucial pathogenesis, and various inflammatory factors, such as TNF-α, Interleukin-1 and interferon γ, are elevated (31). TNF-α induces serine phosphorylation of serine phosphorylation of insulin receptor substrate-1 (IRS-1) at Ser312, thereby impairing IRS-1 function and subsequently blocking insulin signaling (32, 33). IL-1 inhibits insulin signaling by activating the NF-κB inflammatory signaling pathway, leading to serine phosphorylation of IRS-1 (34). IFN-γ can modulate the phosphorylation status of IRS through activation of the p38-MAPK signaling pathway, thereby disrupting insulin signaling and ultimately leading to insulin resistance (35). Inflammation interferes with insulin signal transduction through blood and/or paracrine functions and leads to insulin resistance (36). Inflammation is a nonspecific response to injury or stress. In patients with diabetes with hyperglycemia, glial cells are activated by changes in biological pathways such as polyols, protein kinase C, advanced glycation end products, and the renin–angiotensin system, and release many inflammatory factors (37). Increased inflammatory factors can damage the retina by promoting angiogenesis and neurodegeneration (38, 39). The upregulation of IL-8, TNF-α, ICAM-1, and other inflammatory factors and leukocytosis, glial cell activation, and other inflammatory reactions, inhibiting inflammation, can effectively alleviate the development of DR (40).

#### 3.1.4 Oxidative stress

Oxidative stress can change renal hemodynamics and destroy glomerular endothelial cells, leading to extracellular matrix deposition and thickening of the glomerular basement membrane (41). NADPH oxidase and mitochondrial dysfunction are the two principal mechanisms of oxidative stress in patients with T2DN (42). High glucose levels can stimulate the production of reactive oxygen species (ROS) and nitrogen species (43). ROS can initiate polyunsaturated fatty acids in cell membranes, leading to lipid peroxidation, which produces a series of harmful by-products that disrupt the integrity and function of cell membranes (44). ROS also can directly induce DNA damage, encompassing base modifications, single and double strand breaks, as well as DNA cross-linking (45). Studies have shown that in T2DM, persistent high blood sugar is a stress stimulus capable of increasing oxidative stress, which can be seen at both cellular and plasma levels (46, 47). Cellular responses to elevated oxidative stress include increased poly ADP-ribose polymerase activity. Elevated poly ADP-ribose levels are important predictors of elevated IL-6 and TNF-α levels, and their role in modulating inflammatory pathways in T2DM suggests that oxidative stress can regulate inflammatory responses (46).

#### 3.1.5 Metabolic disorder

##### 3.1.5.1 High glucose

Under continuous hyperglycemia, the increase in glucose oxidation and the production of mitochondrial reactive oxygen species in patients aggravates oxidative stress and accelerates the speed of apoptosis and the degree of DNA damage, resulting in renal tubular and interstitial fibrosis. Hyperglycemia directly induces the activation of the polyol pathway, changes in the redox state of cells, an increase in triglyceride production, the activation of the protein kinase C (PKC) pathway, and the generation of advanced glycation end products (AGEs), resulting in abnormalities in the retinal nerve tissue and microvascular system (48–50).

#### 3.1.6 Advanced glycation end products

AGEs can be generated through the reaction between sugars and amino groups of dicarbonyl-modified proteins. Under conditions of hyperglycemia, the production rate of AGEs increases, leading to abnormal accumulation in the retina. This process not only directly alters the normal structure of proteins, nucleic acids, and lipids but also activates multiple signal transduction pathways, resulting in apoptosis of retinal pericytes, dysfunction of endothelial cells, increased secretion of vascular endothelial growth factor (VEGF), and induction of oxidative stress and inflammatory responses. These mechanisms are primarily responsible for the onset and progression of diabetic retinopathy (DR) (51).

#### 3.1.7 Protein kinase C

PKC activation induces the downregulation of endothelin-1 receptor and upregulation of VEGF. Endothelin-1 is a powerful vasoconstrictor that can cause hemodynamic changes. VEGF plays an crucial role in signal transmission in cells, leading to changes in vascular permeability, and promoting endothelial cell proliferation, migration, and angiogenesis (52). PKC activation plays a role in the abnormal regulation of blood vessels and retinal neovascularization (53).

### 3.2 Pathogenesis by complications

#### 3.2.1 Type 2 diabetic peripheral neuropathy

Studies have indicated that a high expression of IL-6 is detected in the DRG of diabetic mice, and IL-6 can bind to and induce the dimerization of multiple receptor complexes and then activate JAK in the cell membrane to induce the transcription of related neuroprotective genes. Diabetic neuropathy is closely related to oxidative stress, and the anti-oxidative stress effect of the JAK-STAT signaling pathway provides a new approach for the treatment of DPN (54). Moreover, neurons are supported by the nutritional support of their innervating tissues, which produce nerve growth factors and nutritional factors. The lack of cell nutrition, nerve growth, and nutritional factors affects the normal function of nerve fibers, leading to varying degrees of nerve fiber damage (55).

#### 3.2.2 Type 2 diabetic retinopathy

Endothelial progenitor cells (EPCs) may help increase the growth of collateral vessels to ischemic tissues (therapeutic angiogenesis) and deliver antiangiogenic agents or proangiogenic agents to pathological or functional angiogenesis sites, respectively. In patients with DR, the fate of EPCs is also affected by the mitochondrial state and oxidative stress. On the one hand, a high concentration of glucose can induce autophagy of EPCs, accompanied by an increase in mitochondrial oxidative stress and the destruction of mitochondrial oxidative permeability; the number and function of mitochondria in DR patients are damaged, leading to a decline in EPC metabolism and function (56, 57). DR-related retinal neurodegeneration occurs before vascular changes, which are called diabetic retinal neurodegeneration (DRN). Many animal studies have also shown that DRN is an early component of DR in terms of structure and function. Park et al. observed that photoreceptor apoptosis first occurred within 4 weeks of diabetes onset, and the thickness of the outer nuclear layer decreased significantly at 24 weeks. The outer nuclear layer is the nucleus of the photoreceptor, which is not only the main site of superoxide production in diabetic mice but can produce inflammatory proteins, resulting in increased permeability and death of retinal endothelial cells (58–60).

#### 3.2.3 Type 2 diabetic nephropathy

Hyperglycemia can promote the release of various vasoactive mediators, namely, insulin-like growth factor-1 (IGF-1), VEGF, and nitric oxide (NO) from the kidney, resulting in the dilation of renal arterioles; however, due to the local elevation of angiotensin II (Ang II) and endothelin-1 (ET-1), the efferent arterioles contract, leading to the formation of glomerular hypertension and the occurrence of DN (61). Another metabolic pathway is the renin–angiotensin–aldosterone system (RAAS). Overactivity of RAAS is a key factor in renal fibrosis, which is also closely related to the occurrence of DN. A mouse-based animal experiment showed that the use of RAAS inhibitors, ACE, or an angiotensin-converting enzyme inhibitor significantly reduced renal fibrosis (62).

Mitochondria are crucial organelles for energy production and the main source of reactive oxygen species (ROS) production. Dysfunction can also lead to DN. When the body’s blood glucose increases, the load of the mitochondrial electron transport chain increases, resulting in insufficient production of adenosine triphosphate (ATP), NO, and nuclear factor κB(NF-κB). This phenomenon can promote inflammation and vascular dysfunction in the kidney and finally form DN. Based on the aforementioned theory, some scholars have long proposed improving mitochondrial oxidative phosphorylation activity through exercise, heat restriction, or drug stimulation of AMPK to reduce the risk of DN (63).

## 4 Metabolic information statistics of clinical differences

**Figure 3:**
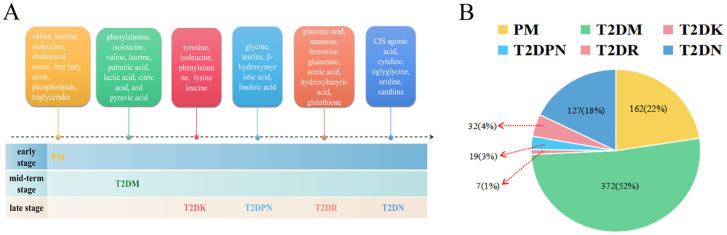
Metabolites of diabetes mellitus and its complications. (**A**: Metabolite profiles evolve from prediabetes to complications, **B**: Proportional representation of metabolites across stages).

According to the aforementioned 85 pieces of literature, 589 differential metabolites were identified (**Figure 3**, **Supplementary Table S1, S2**). The classification of differential metabolites in the class column were obtained through the following steps: we upload the names of metabolites into the human metabolome database (HMDB, https://hmdb.ca/) and inquiries the results.

### 4.1 Alterations of different metabolites in different stage

**Figure 4:**
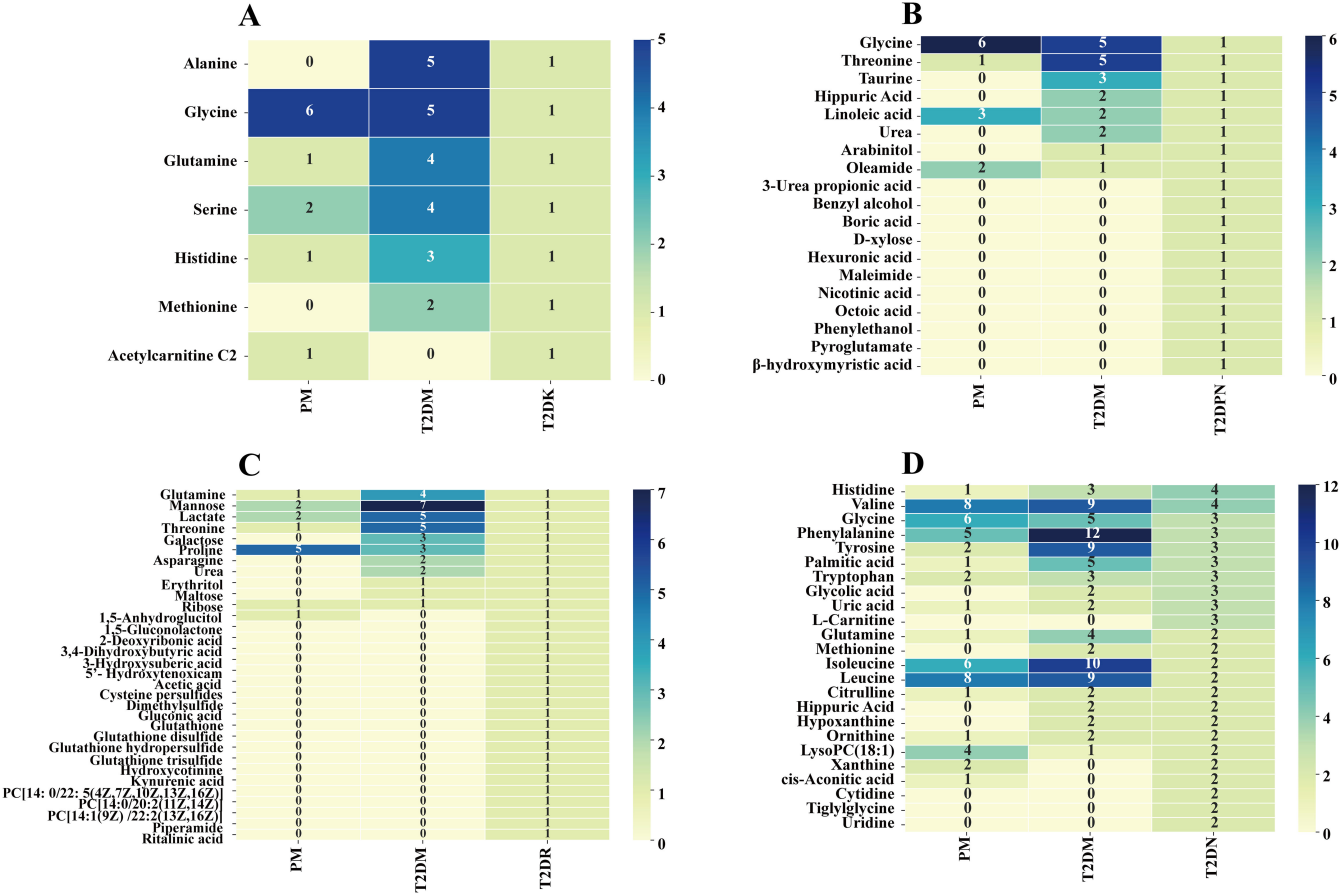
Clinical metabolic profile in T2DM complications. (**A**: T2DK, **B**: T2DPN, **C**: T2DR, **D**: T2DN).

First, the type and frequency of different metabolites of type 2 diabetes and its complications were analyzed (**Figure 4**). Clinical metabolite analysis revealed changes in metabolites associated with disease progression, both longitudinally (across disease stages) and laterally (from prediabetes to complications). The metabolic profile covers a wide range of metabolic products, including glycerol phospholipids, amino acids, carboxylic acids, and fatty acids.

A total of 162 differential metabolites were obtained from the PM (**Supplementary Table S3**). The top 25 differential metabolites included 12 amino acids, 6 glycerophosphatidylcholine, 2 glucose, and other components. The most frequent type was branched-chain amino acids, namely, valine, leucine, and isoleucine. There were 372 differential metabolites in T2DM among which there were 14 amino acids, including branched-chain amino acids and aromatic amino acids, 4 monosaccharides (e.g., α-glucose), 7 organic acids, and other components (**Supplementary Table S4**). There were 7 differential metabolites in T2DK: 7 straight-chain amino acids, including the special amino acid acetylcarnitine C2 (**Supplementary Table S5**). There were 19 metabolites in T2DPN, which were ranked as 8 organic acids, 4 amino acids and their derivatives, 3 alcohols, 2 organic nitrogen compounds, 1 inorganic acid, and 1 salt compound (**Supplementary Table S6**). There were 32 metabolites in T2DR. According to the order of frequency, 7 organic acids, 5 sugars, 3 glycerophosphatides, 3 peptides and derivatives, and 3 linear amino acids can be obtained (**Supplementary Table S7**). A total of 127 differential metabolites were identified in T2DN. Among the top 25 metabolites, the main types included 15 amino acids, 5 organic acids, and 3 phosphatidylcholines (**Supplementary Table S8**). In total, the number of investigations focusing on the metabolic profile of T2DK was the lowest, followed by T2DPN and T2DR (**Figure 3B**). This result is mainly due to the large sample size of patients with T2DM and reflects the maturity of detection technology.

**Figure 4** demonstrates that progressive research remains scarce, and there is a shortage of the early diagnosis and prediction of diabetic complications. Additionally, the PM stage can be a significant stage in terms of diet and exercise. Notably, amino acids account for the largest proportion of various complications when using PM and T2DM as references. Acetylcarnitine C2 shows a different research trend from other amino acids in T2DK. In a study on T2DM, a significant reduction in acetylcarnitine was observed, suggesting either decreased acetyl-CoA production or increased diversion of acetyl-CoA into oxidation or synthesis pathways (64). The research and metabolic features of chronic complications, including T2DPN, T2DR, and T2DN, are more abundant and can provide information concerning biomarkers for further study. For instance, glycine and urea can be detected in several stages and thus require further clinical development. Dysregulation of glycine and urea metabolic pathways was found in the metabolomics analysis of diabetic mice (65). The results showed that creatine metabolites in the model group were significantly higher than those in the control group, indicating that the relative concentrations of glycine and urea were up-regulated, and their concentrations were positively correlated with the progression of T2DM.

### 4.2 Other characteristics of differential metabolites

**Figure 5:**
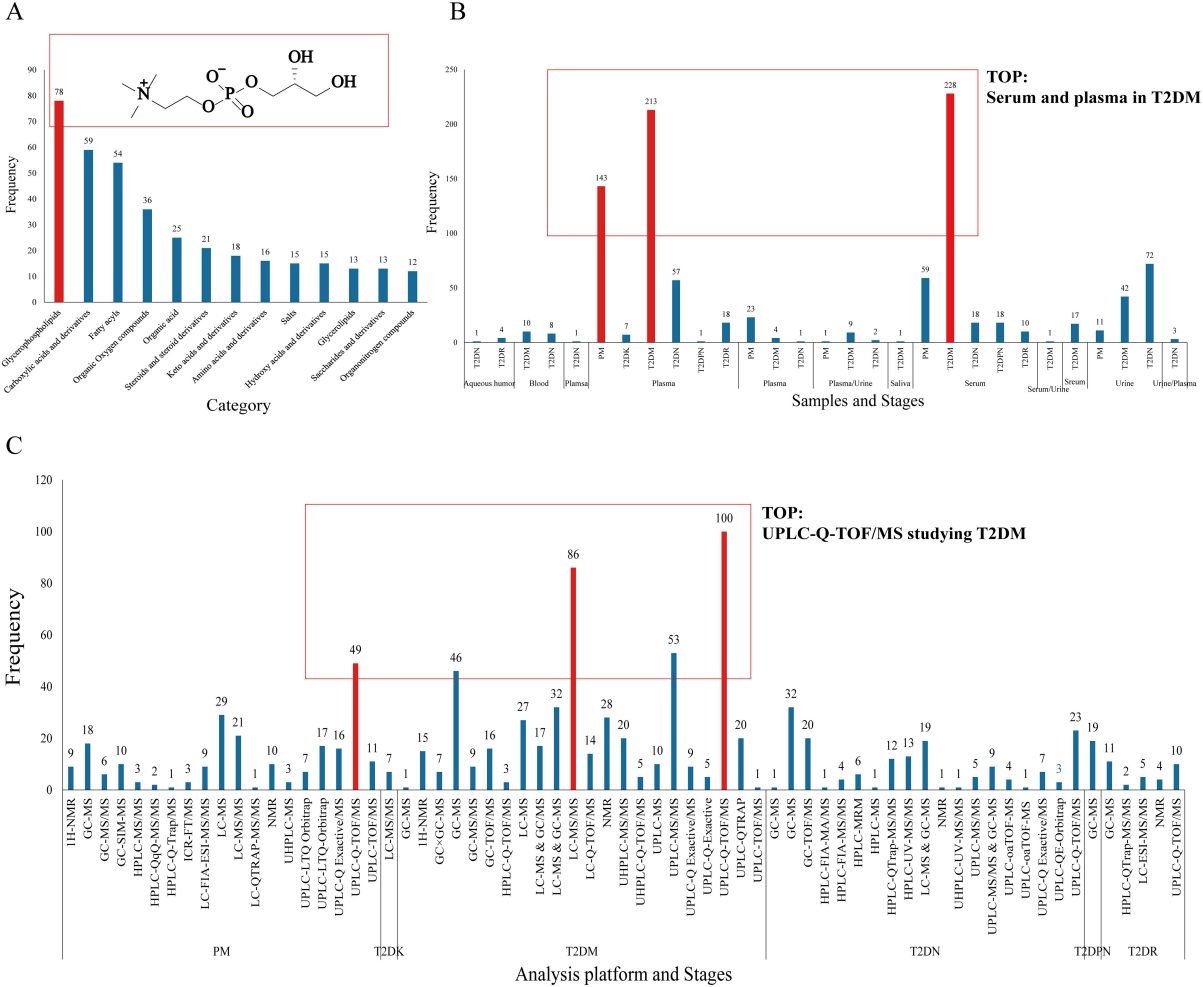
Differential metabolite frequency. (**A**: Category and structure of choline glycerophosphate, **B**: sample type, **C**: analysis platform).

The differential metabolites were classified and summarized according to the type of metabolite of HMDB. The results showed that 12 types of compounds had a frequency of ≥10 times (details in **Figure 5**). Glycerophosphatides are the most common substance, followed by carboxylic acids and derivatives, fatty acids, organic oxygen compounds, and so forth. The typical structure of glycerophosphatides is choline glycerophosphate. In this section, ipidome consists of the cell membrane. We focus on the identification of novel diagnostic biomarkers, as well as the numerous therapeutic targets. Additionally, LC-MS/MS could be an extensive, informative technique for lipidome. In **Supplementary Table S2**, the detection technologies for differential metabolites mainly include H-NMR, GC-MS, LC-MS, and UPLC-MS; among them, there are 11 types of MS detectors, including SIM, TOF, FIA, MRM, QqQ, and LTQ Orbitrap, among which UPLC-Q-TOF/MS is the most frequently used. NMR has been replaced by LC-MS/MS and GC-MS because of its high sensitivity, high resolution, and excellent qualitative and quantitative efficiency.

In terms of sample types, there are six types of serum and plasma samples—aqueous humor, blood, plasma, serum, urine, and saliva—with the largest number of serum and plasma samples in the T2DM period, totaling approximately 450 samples. Another feature is that serum and plasma samples ranked the highest, and their advantages are stability, easy access, and rich metabolic characteristics in blood (66). Urine, as an end product of metabolism, is an crucial sample type in metabolic research.

### 4.3 Pathway analysis

**Figure 6:**
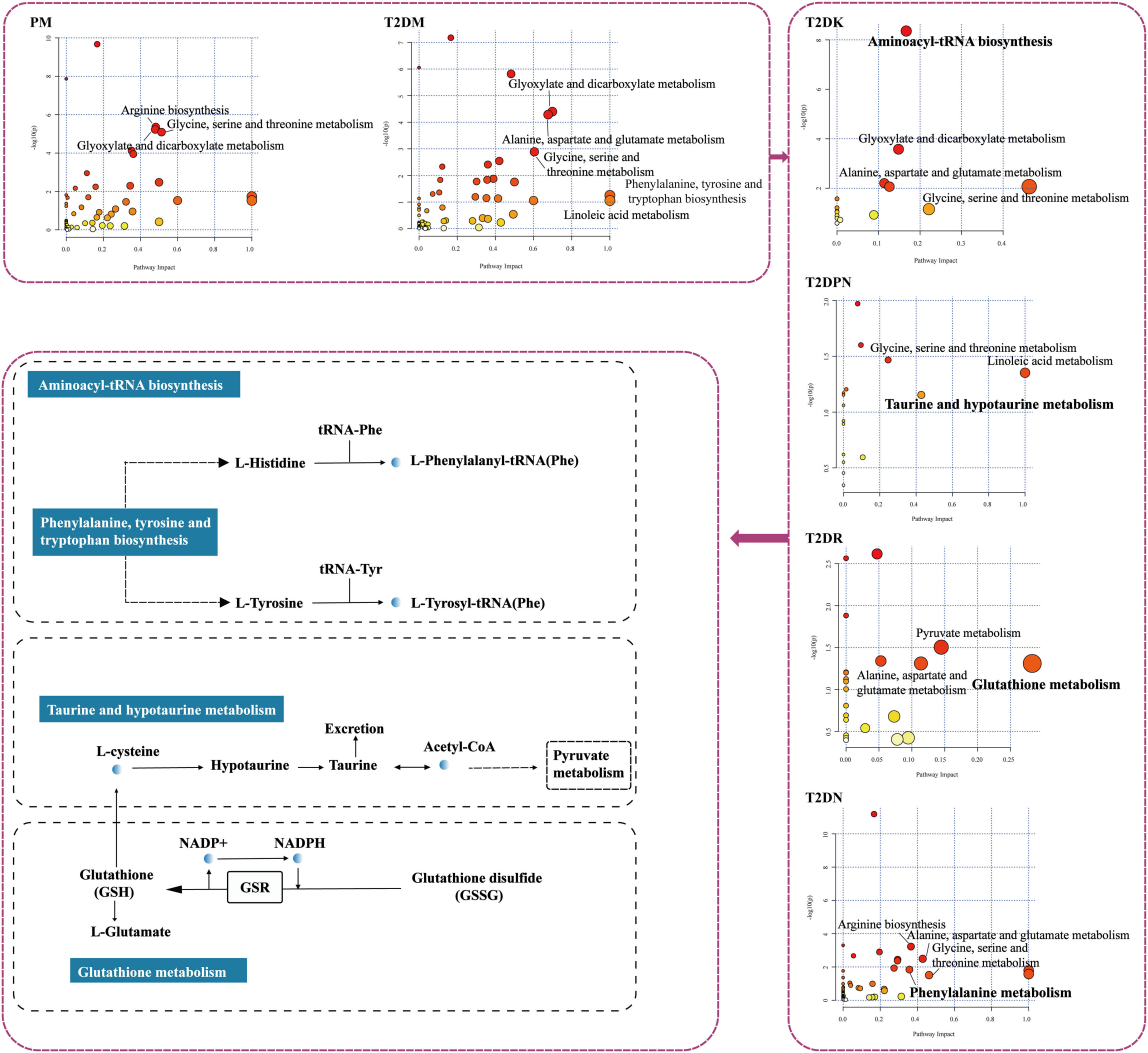
Results of pathway analysis. (The color of the circle represents the significance of the statistical difference; the size of the circle represents the magnitude of the influence value of the pathway). Based on the aforementioned information, the pathway with higher impact value and the significant high -log (p) of pathway coverage is more important. Considering the time sequence from the development of prediabetes to the complications of diabetes, the key pathways obtained by each stage are shown in the Discussion section.

The metabolomics analysis platform MetaboAnalyst was used to analyze the different metabolites at different stages (http://www.metaboanalyst.ca/faces/ModuleView.xhtmL). The database was used for pathway analysis, and the HMDB IDS corresponding to different stages was imported. The channel database type was Homo sapiens, and the pathway calculation method was the hypergeometric test; next, the pathway topology analysis method was selected as the relative centrality. Through the *p* value of the calculation and impact value of pathway topology analysis, MetaboAnaylst displays all matching pathways in the form of a metabolomics diagram. The results are shown in **Figure 6**. The ordinate of metabolomics is -log(p), where *p* represents significance. The larger -log(p), the more significant the statistical difference; the abscissa path impact is the pathway impact value, and the greater the pathway impact value, the more metabolites are queried to hit the pathway.

## 5 Biological function of metabolites

### 5.1 Analysis of metabolites of T2DM over time

#### 5.1.1 PM

The main metabolites of PM are amino acids, lipids, sugars, and fatty acids. IFG and IGT can be observed in patients with PM. IGT and IFG can be regarded as an transitional stage from normal to diabetes. At this time, insulin sensitivity is reduced due to insulin resistance. Long-term fatigue of beta cells can easily lead to functional failure. If properly treated, this status can be reversed to normal. If not treated properly, diabetes develops. Lipid compounds comprise include cholesterol esters, free fatty acids, phospholipids, and triglycerides (67). In **Figure 1**, phospholipids are the lipid with the highest frequency in the PM phase. Phospholipids include glycerin and sphingomyelin. Glycerophosphatidylcholine is also known as glycerophosphate. Depending on the different hydroxyl groups of phospholipid acids, they can be divided into PC, phosphatidylethanolamine, phosphatidylserine, and phosphatidylinositol. LysoPC is the most abundant lysophospholipid in the human blood. LysoPC(18:1), lysoPC(18:0), lysoPC(16:0), lysoPC(17:0), lysoPC(18:2), and lysoPC(14:0) are six types of phospholipids. LysoPC(18:2) has been shown to be related to BMI and T2DM (68). Secretory phospholipase A2 (sPLA2) produced by vascular smooth muscle cells and macrophages A2 can hydrolyze cell membranes and lipoproteins, including low-density lipoprotein and high-density lipoprotein phosphatidylcholine second acyl group, to produce lysophosphatidylcholine and free fatty acids, mediating the production of inflammatory mediators. However, a long-term increase in lipid compounds leads to initial insulin resistance in peripheral tissues, which is in the early stage of the disease, and the body has not yet developed diabetes. The increase in inflammatory mediators accelerates the occurrence and development of chronic complications in T2DM (69).

The main amino acids are branched-chain amino acids (BCAAs), including valine, leucine, and isoleucine. BCAAs can promote glucose uptake and glycogen synthesis in the liver and skeletal muscles. Under the condition of a high-fat diet, high-sugar diet, or overnutrition, the catabolism of BCAAs in various tissues of the body, especially in adipocytes, is inhibited, increasing blood concentration (70). Tulipani et al. (71). demonstrated that valine levels in BCAAs are positively correlated with insulin resistance, which can regulate the growth and proliferation of beta cells and insulin secretion; aromatic amino acids such as phenylalanine was studied in the course of diabetes (72), and the increase in serum phenylalanine concentration is due to insulin deficiency. When insulin secretion is insufficient, the body cannot make full use of glucose and fatty acids for the energy supply; free phenylalanine and other amino acids are the main sources of the energy supply; thus, they appear in a large number in serum. In a study of 1302 individuals aged 40-79, higher levels of BCAAs were associated with metabolic syndrome, obesity, cardiovascular risk, dyslipidemia and hypertension (73). Increases in BCAAs observed in prediabetic individuals with obesity are primarily attributed to insulin resistance; however, once elevated, BCAAs may contribute causally to the progression from prediabetes to full-blown T2DM (74).

#### 5.1.2 T2DM

**Figure 7:**
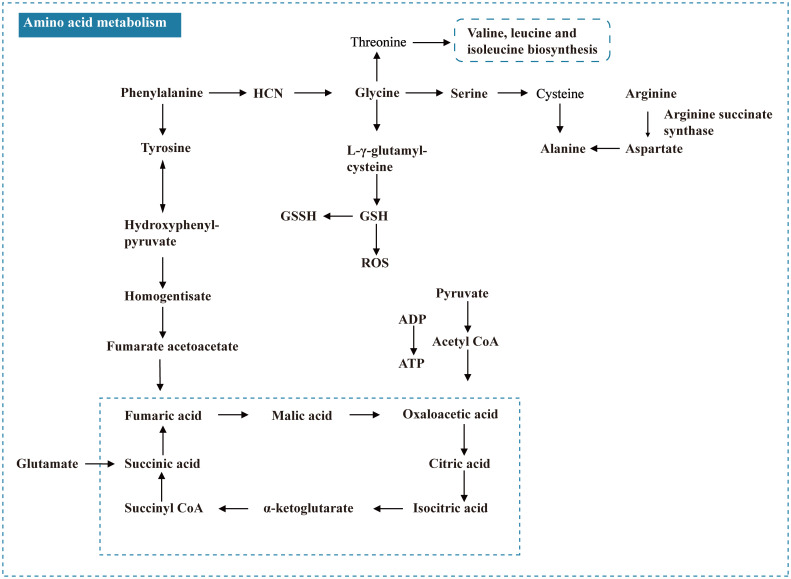
Amino acid metabolism of T2DM.

The differential metabolites of diabetes include phenylalanine, isoleucine, valine, taurine, and other amino acids (**Figure 7**); fatty acids such as palmitic acid; and intermediate products of the tricarboxylic acid cycle, such as lactic acid, citric acid, and pyruvic acid. There are two reasons why lactic acid can be used as a marker for T2DM. First, the increase in lactic acid levels in patients with T2DM can indicate a disorder of glucose metabolism. The process of dehydrogenation and oxidation of lactic acid to pyruvate is inhibited because of microcirculation disorder and the poor oxygen supply of cells, leading to an increase in lactic acid *in vivo*; in addition, in the hyperglycemia state, D-lactic acid, an isomer of lactic acid, changes from elevated malonic acid to pyruvate ketoaldehyde and can intensify oxidative stress and the glycosylation process of tissues, promote the formation of advanced glycation end products, and accelerate the process of microvascular complications and peripheral neuropathy in T2DM (75).

Amino acid metabolism is a major metabolic change related to insulin sensitivity. There are BCAAs, aromatic amino acids, and special amino acids such as taurine. Clinical studies have shown that elevated BCAA levels are associated with the risk of T2DM (76, 77), and genetic studies have also confirmed the crucial role of BCAA metabolism in the development of DM (78). For example, 3-hydroxyisobutyric acid (3-HIB), a newly found paracrine regulator of trans-endothelial fatty acid transport, can activate trans-endothelial fatty acid transport and promote the absorption and accumulation of fatty acids in muscle, producing insulin resistance (79), which can be used as a link between BCAA catabolism and insulin resistance. Additionally, Winiarska et al. (80). found that taurine affects the prevention and control of T2DM complications, which may be achieved by inhibiting gluconeogenesis, and taurine has antioxidant and neuroprotective effects.

O’Connell (81) found that the accumulation of BCAAs can activate mammalian rapamycin targeted gene complex I (mTORC1) and then affect the downstream target ribosomal protein S6 kinase I (S6K1), affecting insulin sensitivity in the body. This regulation effectively confirmed the close relationship between DM and amino acid metabolic disorders. Branched-chain amino acid metabolism (82) was also found to affect the energy supply of the body by influencing the TCA cycle, which is closely related to the metabolic process of the disease. According to the theory of lipotoxicity in DM, the long-term increase in free fatty acids results in cell and tissue toxicity, which can cause secretion dysfunction of the pancreas or isolated islets. FFAs are donors of the lipid structure of the cell membrane and prostaglandin synthesis. They are the intermediate products of fat metabolism and the main source of energy for the body. Under normal physiological conditions, the concentration is in a relative equilibrium state. When pathological changes occur in the body, the balanced state is destroyed, and the concentration in the body changes (83). Excessive FFAs produce many free radicals and participate in oxidative stress. Palmitic acid is the most abundant free fatty acid in the body. High concentrations of palmitic acid can increase endothelial cell apoptosis (84, 85) and ROS (86).

#### 5.1.3 T2DK

For patients with DM with ketosis or ketoacidosis and other complications, the discovery of its differential metabolites can provide diagnostic features for clinical diagnosis. In patients with diabetes, insulin deficiency leads to increased gluconeogenesis (hepatic glucose production), accompanied by impaired glucose uptake and use in peripheral tissues, leading to hyperglycemia. The increase in gluconeogenesis is due to the increased utilization of gluconeogenic precursors, such as lactic acid, glycerol, and several gluconeogenic amino acids, including alanine, glycine, and serine. In addition, low insulin concentrations lead to muscle protein catabolism, releasing gluconeogenic and ketogenic amino acids (e.g., tyrosine, isoleucine, and phenylalanine), or pure ketogenic amino acids (e.g., lysine and leucine), which lead to an increase in ketone bodies and excessive accumulation in the body, causing “acidosis” (87).

#### 5.1.4 T2DPN

The differential metabolites of diabetic peripheral neuropathy included amino acids such as glycine and taurine and fatty acids such as β-hydroxymyristic acid and linoleic acid (FFA), which indicated that amino acid metabolism and lipid metabolism might be abnormal in the pathogenesis of DPN. Gou Xiaojun (88) found that this compound is one of the raw materials for glutathione synthesis (GSH). GSH can scavenge oxygen free radicals, reduce the occurrence and development of neuropathic pain caused by ROS, and may have an analgesic effect in the neuropathic pain model; taurine is also an antioxidant and can directly inhibit the production of ROS to reduce oxidative stress in patients with diabetes by upregulating the antioxidant defense system in cells. Oxidative stress is the pathogenesis of diabetes mellitus and its associated complications. It can damage nerve microcirculation through microvascular damage, resulting in decreased blood perfusion and DPN. Due to the relative or absolute deficiency of insulin, long-term hyperglycemia further leads to dyslipidemia and excessive FFA (89), which can cause ischemia and hypoxia of nerve tissue, and *in vitro* experiments have shown that FFA directly inhibits the synthesis or release of neuropeptides, which may exert a direct cytotoxic effect on the nervous system, and can participate in the oxidative stress process of the human body by generating many free radicals, mediating neuropathy.

#### 5.1.5 T2DR

DR is the most common ocular complication of DM. The organic acid differential metabolites are, for example, lactic acid, gluconic acid, and fatty acid, which are converted into pyruvate and then oxidized to acetyl coenzyme A, which enters the TCA cycle. Lactic acid is the end product of glycolysis and is a special material for aerobic metabolism. High glucose-induced mitochondrial dysfunction leads to cell division and reduced respiratory capacity. Therefore, the decrease in lactate levels may be related to mitochondrial damage and accompanied by decreased energy metabolism. Studies have shown that mannose and erythritol can be used as biomarkers of DM and IFG and Mannose and erythritol can also be used as potential clinical metabolomic markers of T2DR. Amino acids (e.g., threonine and glutamine) are decomposed into oxaloacetic acid, α-ketoglutarate, or succinyl coenzyme A, which are oxidized into CO_2_ and H_2_O in the TCA cycle and then oxidized and phosphorylated to ATP. Studies have shown that hyperglycemia and hypoxia exert additional effects on the accumulation of electrons and protons in the intracellular free NADH pool, and the ratio of NAD/NADH in the diabetic retina is significantly reduced. Therefore, the changes in these amino acids may be due to the inhibition of the TCA cycle and catabolism by high-level NADH/NAD+ in DR patients.

#### 5.1.6 T2DN

DN is the main lethal factor in diabetes. In the summary results of this study, the changes in BCAAs and fatty acids in patients with DN are obvious. Elevated serum levels of BCAAs, such as valine, can predict insulin resistance and diabetes. Insulin resistance in patients with diabetes leads to T2DN, which increases the level of serum BCAAs in patients with DN. However, with the development of DN, acidosis may be caused by a decrease in renal function and glomerular filtration rate, enhancing branched-chain amino acid catabolism and decreasing its level. In addition, glutamine, the most abundant amino acid in the human body, can improve insulin resistance as an important intermediate of glucose metabolism and amino acid metabolism, When the serum taurine level in patients with T2DN is lower than the normal, taurine can inhibit renal local lipid peroxidation, and AGEs formation and reduce high glucose-induced renal tubular injury.

IR and fatty acid metabolism affect each other. The disorder of fatty acid metabolism can lead to excessive accumulation of free fatty acids in the liver, skeletal muscle, and other parts, leading to or aggravating insulin resistance, which may lead to diabetes. Insulin resistance can also aggravate the metabolic disorder of fatty acids, mainly through the influence of glucose metabolism to increase the decomposition of fat metabolism, and a large amount of glucose and free fatty acid deposition in the liver can synthesize many low-density lipoproteins, which can lead to hyperlipidemia. Semba conducted a plasma metabolomics study on 472 patients with an average age of 70.7 years. The results showed that 16 types of polyunsaturated fatty acids were the lipid metabolites associated with abnormal glucose metabolism and insulin resistance in elderly individuals, including various PC and LPC. In addition, *in vitro* experiments confirmed that elevated levels of free fatty acids in urine can cause tubulointerstitial damage in patients with DN, and palmitic acid can induce apoptosis of renal tubular epithelial cells.

### 5.2 Temporality analysis of differential metabolites

According to the summary results of the aforementioned differential metabolites, **Supplementary Table S2** and **Figure 1** show that the most frequently occurring metabolites are amino acids, carbohydrates, and lipid metabolites. The discussion and analysis in this paper are based on the changes in patients with symptoms emerging from PM to various diabetic complications, and the diabetic complications that have occurred before the development of diabetes are not discussed. Subsequently, the horizontal development of detailed metabolites from light glucose damage to severe glucose injury in acute and chronic complications was analyzed as follows:

From PM to T2DM: LysoPC components were higher in the PM stage compared with T2DM, and the amino acids also increased during this period; additionally, the frequency of BCAAs was higher than that of straight-chain amino acids, and phenylalanine had the highest frequency. On the one hand, the increase in the frequency of α-glucose indicates that the related research is increasing; on the other hand, it is easy to detect and highly representative in the T2DM stage.

From T2DM to T2DK: Because few patients have such acute complications, few have been conducted studies on metabolic biomarkers in T2DK. Notably, amino acids and glutamine are both present in T2DM and appear frequently. Acetylcarnitine C2, a small metabolic molecule in the body, plays a crucial role in cellular respiration by removing acetyl groups from cells and facilitating energy transfer. It acts as a carrier for fatty acids (FAs) to enter the mitochondria, where ATP is produced during cellular respiration (90). Acetylcarnitine is an ester of acetyl coenzyme A (acetyl-CoA) and carnitine. It facilitates the transport of long-chain fatty acids into the mitochondria for β-oxidation, which is crucial during periods of ketosis when the body relies on fat as a primary energy source. In T2DK, where insulin sensitivity is impaired, the ability to utilize glucose is compromised, making fatty acid metabolism and the utilization of ketone bodies increasingly important. Acetylcarnitine helps bridge the gap in energy metabolism by enhancing fatty acid oxidation (91). Moreover, acetylcarnitine C2 is the sole metabolite reduced in the T2DK stage and elevated as quantified by MRM before the occurrence of ketosis (92); thus, it may become the key marker of follow-up research.

From T2DM to T2DPN: Few studies have investigated the different metabolites in the T2DPN stage, which may be due to the following: the occurrence of neuropathy with other complications, that there are few patients with simple peripheral neuropathy, and that the evaluation index of neuropathy is relatively complex. The highest frequency of components in diabetes was amino acids, and in T2DPN, amino acid types decreased. Compared with the 373 metabolites of T2DPN, there were eight different metabolites coincident, and the other 11 were unique to T2DPN: 3-Urea propionic acid, benzyl alcohol, boric acid, d-xylose, hexuronic acid, maleimide, nicotinic acid, octoic acid, phenylethanol, pyroglutamate, and β-hydroxymyristic acid.

From T2DM to T2DR: Compared with the 32 components in T2DR, the organic acids, organic nitrogen compounds, and phosphatidylcholine increased in the T2DM stage, including acetic acid, hydroxybutyric acid, glutathione, and its sulfide and three phosphatidylcholines (PC[14:0/22:5(4Z, 7Z, 10Z, 13Z, 16Z)], PC[14:0/20:2(11Z,14Z)], and PC[14:1(9Z)/22:2(13Z,16Z)]).

From T2DM to T2DN: Compared with 127 differential metabolites in T2DN, five differential metabolites (frequency ≥2) of CIS agonic acid, cytidine, tiglyglycine, uridine, and xanthine were exclusive to T2DN.

From PM to T2DM to T2DK, aminoacyl-tRNA biosynthesis. Aminoacylation of transfer RNAs establishes the rules of the genetic code. The reactions are catalyzed by an ancient group of 20 enzymes (one for each amino acid) known as aminoacyl-tRNA synthetases (AARSs). The etiology of specific diseases is related to the specific AARS, that is, the heritable mutation in the tRNA synthetase gene, which has a causal relationship with disease. Both the dominant and invisible pathogenic mutations were annotated. For example, intracellular synthetases form multiple protein complexes with each other or with other regulatory factors, controlling multiple signaling pathways. Mitochondrial interleukin tRNA synthetase (Mito LRS), encoded by the nuclear genome, stimulates the accumulation of mitochondrial tRNA mutations by inducing oxidative stress in hyperglycemia and hyperinsulinemia during the development of DM. Nevertheless, a single nucleotide polymorphism of Mito LRS was also found in patients with type 2 diabetes, resulting in an amino acid substitution (h324q) (93); however, h324qmito LRS has normal aminoacylation and editing activity; thus, the causal relationship between mito-lrsh324q mutation and type 2 diabetes is not clear. Mutations in mitochondrial tRNAs (the substrate of AARS) have similar causal relationships with many diseases, including diabetes mellitus (94, 95). In addition, AIMP 1 regulated glucose homeostasis (96). AIMP 1 gene deletion in mice leads to a hypoglycemic phenotype, but whether this pathological phenotype is also applicable to humans has not been confirmed.

From PM to T2DM to T2DPN, taurine and hypotaurine metabolism. Taurine is a sulfur-containing β-amino acid that can significantly promote the dendritic differentiation and proliferation of human brain nerve cells, increase the total number of human brain nerve cells, promote the synthesis of nucleic acids, accelerate the formation of neural networks, and prolong the survival time of nerve cells. In mammals, taurine is high in the brain, retina, myocardium, liver, kidney, and muscle, as well as in platelets, lymphocytes, and cerebrospinal fluid (97). In hyperglycemia, an increase in intracellular glucose leads to the production of sorbitol by aldose reductase. Organic osmotic pressure (sorbitol, inositol, and taurine) controls cell volume according to changes in extracellular osmotic pressure. Stevens found that inositol and taurine decreased in STZ(Streptozotocin)-induced diabetic rats, but the use of aldose reductase inhibitors helped avoid this situation, suggesting that the accumulation of sorbitol led to a decrease in other organic osmolality (98). Notably, cell exposure to high glucose reduces the expression of taurine transporter; however, the use of antioxidants or aldose reductase inhibitors in high glucose leads to the recovery of taurine transporter expression (99, 100), indicating the importance of sorbitol in intracellular taurine concentration. Taurine supplementation has been used to treat neurologic impairment, such as hyperalgesia and nerve conduction defects, and insufficient nerve blood flow in STZ diabetic models (101–103). Taurine attenuates neuropathy by inhibiting the expression of NF-κB and increasing the expression of GLUT3, GLUT1, HO-1, and Nrf2 in STZ-induced diabetic rats (104).

From PM to T2DM to T2DR, glutathione metabolism. Glutathione is a major antioxidant in all life forms and an indicator of oxidative stress in cells. NADPH is used as an electron donor to reduce glutathione. When the NADPH/NAD+ ratio is decreased, the ratio of GSH reduced to oxidized is also reduced, weakening the antioxidant capacity of the body and deepening the vascular injury (105). Reduced glutathione is metabolized in various ways, leading to the biosynthesis of amino acids, such as sulfhydryl acid, glutamic acid, glycine, and cysteine. Van Hove Inge et al. (106) performed single-cell transcriptome analysis of neurons, glial cells, and inflammatory cells in a DR Akimba rat model. The results showed that glial cells play a key role in retinal neurodegenerative diseases. The differential gene string analysis of macroglia showed that glutathione metabolism regulated astrocytes; subsequently, through the subcellular aggregation of glial cells, class analysis focuses on glutathione metabolism and the specific growth factor pathway.

From PM to T2DM to T2DN. Through the joint analysis of metabolomics and proteomics, Wang et al. (107). The 14 metabolites with significant differences related to insulin resistance were obtained through the joint analysis of metabolomics and proteomics. Bioinformatics annotation analysis showed that phenylalanine, tyrosine, and tryptophan biosynthesis and phenylalanine metabolism pathways were the two most significant pathways in the process of T2DM. Phenylalanine, tyrosine, and tryptophan are aromatic amino acids (AAAS). Tyrosine is most closely related to the occurrence and development of DN. A project published in *Diabetes* in 2015 explored the correlation between amino acid levels in different ethnic groups and the risk of diabetes. Therefore, higher levels of BCAAs and AAAS, especially tyrosine, can increase the risk of diabetes and is expected to become a new mechanism and potential therapeutic target for diabetes in South Asia. In addition, tyrosine-derived phenyl sulfate (PS) can lead to albuminuria in patients with DN. Reducing tyrosine intake in patients with diabetes may reduce PS production. Inhibition of tyrosine in the PS enzyme tyrosine phenol lyase can also protect against T2DN (108).

### 5.3 Limitations

As a clinical diagnostic index, differential metabolites have the advantages of easy detection, high sensitivity, and accurate reflection of physiological and pathological characteristics (109, 110). According to the diagnostic criteria for diabetes published by the World Health Organization, blood glucose content was used as the clinical diagnostic criteria for T2DM. The review results show that more than 18 papers have detected differential metabolites by using the clinical metabolomics technology α-glucose and β-glucose. In addition, urea, a clinical evaluation index of renal function, has also been detected in serum samples in the clinical metabolomics of DN. Therefore, the differential metabolites of clinical metabolomics have great development space for clinical diagnosis. However, this study has limitations:

First, there is a certain gap between the differential metabolites reviewed in this paper and the clinical diagnostic indicators to be examined, which requires further verification. Basic metabolomics research often involves the measurement of hundreds of metabolites, an approach that is neither practical nor cost-effective for large-scale implementation. Furthermore, the specialized laboratory equipment required for metabolomics analysis is both expensive and not readily accessible in clinical settings. Consequently, only a limited subset of clinically relevant metabolites, which can be evaluated using standard equipment or assays, should be integrated into routine clinical practice (111). While metabolomics provides insights into metabolites, its ability to fully reveal the mechanisms of type 2 diabetes is limited, and in order to address this issue, omics and systems biology techniques need to be combined with traditional approaches (14, 112). Finally, indicators for clinical diagnosis must have certain specificities and sensitivities. Therefore, further research on the obtained differential metabolites is necessary, such as using targeted metabolomics for clinical large cohort validation, and the specificity and sensitivity of target metabolites were calculated.

In addition, this paper summarizes the database of differential metabolites through the research literature. Thus, there may be bias in different clinical research stages, and there are many types of bias due to the merger process, specifically, selection bias, information bias, and confounding bias.

The first is selection bias, and the first is the admission rate bias. Due to the differences in population and sample size in the literature integration, for example, there is a gap in the research results of T2DM between Tianjin Fourth Central Hospital and the pathology department of Pittsburgh University Medical School in Pittsburgh, Pennsylvania, due to the differences in living habits, environmental factors, and course changes of the included samples. Nevertheless, there was a distinct difference in the number and type of differential metabolites among the various sample types. For instance, the quantity of serum and plasma samples was the highest, and only five metabolites were detected in the aqueous humor. In addition, there was migration bias: patients with T2DM were compared with the blank group, but the results of this group were compared with those of the T2DR group in another study.

The second factor is information bias. In clinical research, there can be information errors or omissions of research objects. There is a tendency for some indicators in the research of investigators in clinical diagnosis. By expanding the scope of data collection, researchers and research objects can be distracted, and the bias caused by the loss of objective data or the lack of measurement tools and methods can be reduced.

The third factor is confounding bias, that is, the influence of a factor related to both the disease and the factor studied, which conceals or exaggerates the strength of the association between the exposure studied and the disease. In the presence of DM and its complications, the highest level of glucose is detected in metabolite research. However, this phenomenon does not indicate a weak correlation between blood glucose and T2DM. If this is ignored, misleading conclusions may be obtained, and the sensitivity and detection conditions of the instrument detection may be different. Then, for some complications of diabetes, such as T2DPN and T2DK, the included literature is comparatively less than the other complications, and the metabonomic profile analysis results are not generally representative. Therefore, the differential metabolites reviewed in this paper advance the understanding of the mechanism of diabetes and its complications and provide a reference for the discovery of biomarkers and treatment methods. To verify the clinical diagnosis, a large team conducting multicenter targeted studies is necessary.

Additionally, a significant limitation in the practical application of clinical metabolomics is the variability in metabolomics analysis results across different populations and platforms. This variability substantially impacts the reproducibility and generalizability of research findings. Firstly, the serum metabolome varies significantly among populations based on region, gender, and age, indicating that a one-size-fits-all reference standard cannot be simply applied for disease diagnosis and biomarker screening using metabolomics. Secondly, different analytical platforms, such as GC-MS and LC-MS, produce varying spectra for the same metabolite due to differences in detection principles and conditions, increasing the complexity of metabolite identification and affecting the accuracy and comparability of research results. Moreover, pre-analytical factors, including sample storage and preparation, can also influence the metabolic profile, further contributing to result inconsistencies. Therefore, to enhance the reliability and practicality of metabolomics in T2DM research, greater emphasis should be placed on rigorous experimental design, including the selection of appropriate samples, platforms, and data analysis methods, as well as the establishment of standardized operating procedures and data sharing strategies. These measures will help reduce inter-study variability and improve the reproducibility and generalizability of results.

## 6 Conclusion and prospects

A total of 85 metabolomics studies in clinical practice on T2DM and its complications were included from five literature databases. The types and frequency, time sequence changes, sample types, and detection methods for differential metabolites were analyzed and studied. A total of 589 differential metabolites were found in six stages, ranging from PM to T2DN. The highest frequency of T2DM was 372, the lowest frequency of T2DK was 7, and the highest frequency of BCAAs was found in the PM phase, including BCAAs and AAAS in the T2DM stage; 7 amino acids and their derivatives in the T2DK phase; organic acids in T2DPN and T2DR stages; organic acids and amino acids in the T2DN stage. Glycophorophospholipids were the most frequently used differential metabolite types, and UPLC-Q-TOF/MS was the most commonly used detection technology in clinical metabolomics. Serum and plasma samples were the most frequently studied samples. The dominant metabolite types in the disease stratification of T2DM are various, and amino acids, phosphatidylcholine, and organic acids are the common types. Furthermore, the limited availability of data on T2DK and T2DPN presents numerous challenges in conducting robust research. These complications are understudied due to their relative infrequency or complex metabolic profiles. This highlights the critical need for more focused studies to elucidate metabolomic changes in T2DK and T2DPN in T2DM. Future research should aim to recruit larger, more diverse patient cohorts and involve collaborative efforts to develop targeted biomarkers and therapies, thereby enhancing patient care.

Clinical metabolomics has the potential to identify novel biomarkers for early diagnosis, risk assessment, and treatment monitoring in T2DM and its complications. For instance, detecting metabolites associated with diabetic complications can facilitate timely interventions. Metabolomic data can also inform personalized treatments by elucidating individual metabolic responses. Integrating metabolomics into clinical decision-making can optimize therapeutic strategies, leading to improved glycemic control and reduced complication risks. Future research should focus on clinically validating these biomarkers and assessing their impact on patient outcomes through rigorous studies and trials. In addition, clinical metabolomics holds significant potential for the clinical translation of T2DM and its complications, but practical barriers exist. Standardizing metabolomic protocols is essential to mitigate variability in sample collection, processing, and analysis, which can lead to inconsistent findings across studies. Additionally, the high cost of advanced mass spectrometry and data processing software poses a significant barrier to clinical adoption. Overcoming these challenges by establishing robust standardized protocols and developing more cost-effective technologies will facilitate the integration of metabolomics into routine clinical workflows, thereby enhancing the diagnosis and management of T2DM and its complications.

Clinical metabolomics is the application of metabolomics in clinical practice. By comprehensively analyzing the characteristics of metabolomics in distinct stages and combining them with pattern recognition technology, we obtained potential biomarkers related to the occurrence, development, and outcome of diseases and determined the relationship between health and disease to provide the basis for clinical intervention and master clinical detection. After the types and frequencies of different metabolites are obtained from the samples, and the changes over time in distinct stages of diabetes and its complications are assessed, a quantitative verification study of differential metabolites in a specific period can be conducted, the correlation between the differential metabolites and clinical indicators can be studied, and whether the substance can be used as a clinical diagnostic marker for sensitivity and specificity can be determined. If the sample capacity can be expanded, the prevention and treatment of T2DM will make a great breakthrough in this prospective study.

The future research directions for clinical metabolomics in T2DM encompass identifying biomarkers for early diagnosis and risk prediction, exploring the pathogenesis of the disease, investigating biomarkers associated with complications, and evaluating drug mechanisms and efficacy. In terms of clinical applications, metabolomics can enhance diagnostic accuracy by integrating traditional indicators; facilitate personalized treatment plans based on patients’ metabolic profiles to optimize drug selection; monitor disease progression and assess therapeutic outcomes; and support preventive health management by intervening in high-risk populations.

## References

[1] Abel ED Gloyn AL Evans-Molina C Joseph JJ Misra S Pajvani UB. Diabetes mellitus-progress and opportunities in the evolving epidemic. Cell. (2024) 187:3789–820. doi: 10.1016/j.cell.2024.06.029 PMC11299851 39059357

[2] Harding JL Pavkov ME Magliano DJ Shaw JE Gregg EW. Global trends in diabetes complications: A review of current evidence. Diabetologia. (2019) 62:3–16. doi: 10.1007/s00125-018-4711-2 30171279

[3] Madkor HR Abd-El-Aziz MK Abd-El-Maksoud MS Ibrahim IM Ali FEM. Stem cells reprogramming in diabetes mellitus and diabetic complications: recent advances. Curr Diabetes Rev. (2025) 21:21–37. doi: 10.2174/0115733998275428231210055650 38173073

[4] Kostopoulou E Sinopidis X Fouzas S Gkentzi D Dassios T Roupakias S. Diabetic ketoacidosis in children and adolescents; diagnostic and therapeutic pitfalls. Diagnostics (Basel Switzerland). (2023) 13:2602. doi: 10.3390/diagnostics13152602 37568965 PMC10416834

[5] Taimur H Ahmad I Khan H Shirayama Y Okamoto M Aung MN. A scoping review of type 2 diabetes mellitus in Pakistan investigating the status of glycemic control, awareness, treatment adherence, complications and cost. Front Endocrinol (Lausanne). (2024) 15:1441591. doi: 10.3389/fendo.2024.1441591 39649227 PMC11621625

[6] Sun H Saeedi P Karuranga S Pinkepank M Ogurtsova K Duncan BB. Idf diabetes atlas: global, regional and country-level diabetes prevalence estimates for 2021 and projections for 2045. Diabetes Res Clin Pract. (2022) 183:109119. doi: 10.1016/j.diabres.2021.109119 34879977 PMC11057359

[7] Gregg EW Pratt A Owens A Barron E Dunbar-Rees R Slade ET. The burden of diabetes-associated multiple long-term conditions on years of life spent and lost. Nat Med. (2024) 30:2830–7. doi: 10.1038/s41591-024-03123-2 PMC11485235 39090411

[8] Fan C Zhang J Qiu D. Causal relationship between genetically predicted type 2 diabetes mellitus and male infertility. Front Endocrinol (Lausanne). (2024) 15:1357279. doi: 10.3389/fendo.2024.1357279 38529400 PMC10961381

[9] Cerf ME. Beta cell dysfunction and insulin resistance. Front Endocrinol (Lausanne). (2013) 4:37. doi: 10.3389/fendo.2013.00037 23542897 PMC3608918

[10] Le TN Bright R Truong VK Li J Juneja R Vasilev K. Key biomarkers in type 2 diabetes patients: A systematic review. Diabetes Obes Metab. (2025) 27:7–22. doi: 10.1111/dom.15991 39355932 PMC11618249

[11] Weiss RH Kim K. Metabolomics in the study of kidney diseases. Nat Rev Nephrol. (2011) 8:22–33. doi: 10.1038/nrneph.2011.152 22025087

[12] Lindon JC Holmes E Bollard ME Stanley EG Nicholson JK. Metabonomics technologies and their applications in physiological monitoring, drug safety assessment and disease diagnosis. Biomarkers. (2004) 9:1–31. doi: 10.1080/13547500410001668379 15204308

[13] Hameed A Mojsak P Buczynska A Suleria HAR Kretowski A Ciborowski M. Altered metabolome of lipids and amino acids species: A source of early signature biomarkers of T2dm. J Clin Med. (2020) 9:2257. doi: 10.3390/jcm9072257 32708684 PMC7409008

[14] Li J Zhu N Wang Y Bao Y Xu F Liu F. Application of metabolomics and traditional chinese medicine for type 2 diabetes mellitus treatment. Diabetes Metab syndrome obesity: Targets Ther. (2023) 16:4269–82. doi: 10.2147/dmso.s441399 PMC10758184 38164418

[15] Aleidi SM Al Fahmawi H Masoud A Rahman AA. Metabolomics in diabetes mellitus: clinical insight. Expert Rev Proteomics. (2023) 20:451–67. doi: 10.1080/14789450.2023.2295866 38108261

[16] Mariani LH Pendergraft WF 3rd Kretzler M. Defining glomerular disease in mechanistic terms: implementing an integrative biology approach in nephrology. Clin J Am Soc Nephrol. (2016) 11:2054–60. doi: 10.2215/CJN.13651215 PMC5108211 27630182

[17] Mathe EA Patterson AD Haznadar M Manna SK Krausz KW Bowman ED. Noninvasive urinary metabolomic profiling identifies diagnostic and prognostic markers in lung cancer. Cancer Res. (2014) 74:3259–70. doi: 10.1158/0008-5472.CAN-14-0109 PMC4100625 24736543

[18] Wu T Qiao S Shi C Wang S Ji G. Metabolomics window into diabetic complications. J Diabetes Investig. (2018) 9:244–55. doi: 10.1111/jdi.12723 PMC5835462 28779528

[19] Liu C Zhang C He T Sun L Wang Q Han S. Study on potential toxic material base and mechanisms of hepatotoxicity induced by dysosma versipellis based on toxicological evidence chain (Tec) concept. Ecotoxicol Environ Saf. (2020) 190:110073. doi: 10.1016/j.ecoenv.2019.110073 31851898

[20] Tan Z Shen P Wen Y Sun HY Liang HY Qie HJ. Assessment of Metabolomic Variations among Individuals Returning to Plain Areas after Exposure to High Altitudes:A Metabolomic Analysis of Human Plasma Samples with High-Altitude De-Acclimatization Syndrome. Frontiers in molecular biosciences (2024) 11:1375360. doi: 10.3389/fmolb.2024.1375360 38962282 PMC11220191

[21] Cheng C Zhou MX He X Liu Y Huang Y Niu M. Metabolomic Analysis Uncovers Lipid and Amino Acid Metabolism Disturbance During the Development of Ascites in Alcoholic Liver diseases. The Front Med (the Lausanne) (2022), 15467. doi: 10.3389/fmed.2022.815467 PMC9234647 35770013

[22] Carrola J Rocha CM Barros AS Gil AM Goodfellow BJ Carreira IM. Metabolic signatures of lung cancer in biofluids: nmr-based metabonomics of urine. J Proteome Res. (2011) 10:221–30. doi: 10.1021/pr100899x 21058631

[23] Wang C Yang J Nie J. Plasma phospholipid metabolic profiling and biomarkers of rats following radiation exposure based on liquid chromatography-mass spectrometry technique. BioMed Chromatogr. (2009) 23:1079–85. doi: 10.1002/bmc.1226 19382245

[24] Johnson CH Gonzalez FJ. Challenges and opportunities of metabolomics. J Cell Physiol. (2012) 227:2975–81. doi: 10.1002/jcp.24002 PMC6309313 22034100

[25] Mende C Katz A. Cystatin C- and creatinine-based estimates of glomerular filtration rate in dapagliflozin phase 3 clinical trials. Diabetes therapy: research Treat Educ Diabetes related Disord. (2016) 7:139–51. doi: 10.1007/s13300-016-0158-y PMC4801818 26899432

[26] Zhao P Li N Lin L Li Q Wang Y Luo Y. Correlation between serum cystatin C level and renal microvascular perfusion assessed by contrast-enhanced ultrasound in patients with diabetic kidney disease. Renal failure. (2022) 44:1732–40. doi: 10.1080/0886022x.2022.2134026 PMC9586683 36254386

[27] Papaioannou D Brazier J Paisley S. Systematic searching and selection of health state utility values from the literature. Value health: J Int Soc Pharmacoeconomics Outcomes Res. (2013) 16:686–95. doi: 10.1016/j.jval.2013.02.017 23796303

[28] Lu Z Liu N Wang F. Epigenetic regulations in diabetic nephropathy. J Diabetes Res. (2017) 2017:7805058. doi: 10.1155/2017/7805058 28401169 PMC5376412

[29] Wong MCS Huang J Wang J Chan PSF Lok V Chen X. Global, regional and time-trend prevalence of central obesity: A systematic review and meta-analysis of 13.2 million subjects. Eur J Epidemiol. (2020) 35:673–83. doi: 10.1007/s10654-020-00650-3 PMC7387368 32448986

[30] Kashyap S Belfort R Gastaldelli A Pratipanawatr T Berria R Pratipanawatr W. A sustained increase in plasma free fatty acids impairs insulin secretion in nondiabetic subjects genetically predisposed to develop type 2 diabetes. Diabetes. (2003) 52:2461–74. doi: 10.2337/diabetes.52.10.2461 14514628

[31] Thounaojam MC Montemari A Powell FL Malla P Gutsaeva DR Bachettoni A. Monosodium urate contributes to retinal inflammation and progression of diabetic retinopathy. Diabetes. (2019) 68:1014–25. doi: 10.2337/db18-0912 PMC6477903 30728185

[32] Aguirre V Werner ED Giraud J Lee YH Shoelson SE White MF. Phosphorylation of ser307 in insulin receptor substrate-1 blocks interactions with the insulin receptor and inhibits insulin action. J Biol Chem. (2002) 277:1531–7. doi: 10.1074/jbc.M101521200 11606564

[33] Sykiotis GP Papavassiliou AG. Serine phosphorylation of insulin receptor substrate-1: A novel target for the reversal of insulin resistance. Mol Endocrinol. (2001) 15:1864–9. doi: 10.1210/mend.15.11.0725 11682617

[34] Su D Coudriet GM Hyun Kim D Lu Y Perdomo G Qu S. Foxo1 links insulin resistance to proinflammatory cytokine il-1beta production in macrophages. Diabetes. (2009) 58:2624–33. doi: 10.2337/db09-0232 PMC2768186 19651810

[35] Friedline RH Noh HL Suk S Albusharif M Dagdeviren S Saengnipanthkul S. Ifnγ-il12 axis regulates intercellular crosstalk in metabolic dysfunction-associated steatotic liver disease. Nat Commun. (2024) 15:5506. doi: 10.1038/s41467-024-49633-y 38951527 PMC11217362

[36] Satheesan A Kumar J Leela KV Murugesan R Chaithanya V Angelin M. Review on the role of nucleotide-binding oligomerization domain-like receptor protein 3 (Nlrp3) inflammasome pathway in diabetes: mechanistic insights and therapeutic implications. Inflammopharmacology. (2024) 32:2753–79. doi: 10.1007/s10787-024-01556-2 39160391

[37] Callan A Jha S Valdez L Tsin A. Cellular and molecular mechanisms of neuronal degeneration in early-stage diabetic retinopathy. Curr Vasc Pharmacol. (2024) 22:301–15. doi: 10.2174/0115701611272737240426050930 PMC13237750 38693745

[38] Wang Y Tao J Jiang M Yao Y. Apocynin ameliorates diabetic retinopathy in rats: involvement of tlr4/nf-Κb signaling pathway. Int Immunopharmacol. (2019) 73:49–56. doi: 10.1016/j.intimp.2019.04.062 31078925

[39] Mohammad HMF Sami MM Makary S Toraih EA Mohamed AO El-Ghaiesh SH. Neuroprotective effect of levetiracetam in mouse diabetic retinopathy: effect on glucose transporter-1 and gap43 expression. Life Sci. (2019) 232:116588. doi: 10.1016/j.lfs.2019.116588 31226418

[40] Liu Q Zhang F Zhang X Cheng R Ma JX Yi J. Fenofibrate ameliorates diabetic retinopathy by modulating nrf2 signaling and nlrp3 inflammasome activation. Mol Cell Biochem. (2018) 445:105–15. doi: 10.1007/s11010-017-3256-x 29264825

[41] Noh H Ha H. Reactive oxygen species and oxidative stress. Contrib Nephrol. (2011) 170:102–12. doi: 10.1159/000324955 21659763

[42] Su S Ma Z Wu H Xu Z Yi H. Oxidative stress as a culprit in diabetic kidney disease. Life Sci. (2023) 322:121661. doi: 10.1016/j.lfs.2023.121661 37028547

[43] Inoguchi T Sonta T Tsubouchi H Etoh T Kakimoto M Sonoda N. Protein kinase C-dependent increase in reactive oxygen species (Ros) production in vascular tissues of diabetes: role of vascular nad(P)H oxidase. J Am Soc Nephrol. (2003) 14:S227–32. doi: 10.1097/01.asn.0000077407.90309.65 12874436

[44] Lee H Jose PA. Coordinated contribution of nadph oxidase- and mitochondria-derived reactive oxygen species in metabolic syndrome and its implication in renal dysfunction. Front Pharmacol. (2021) 12:670076. doi: 10.3389/fphar.2021.670076 34017260 PMC8129499

[45] Lima JEBF Moreira NCS Takahashi P Xavier DJ Sakamoto-Hojo ET. Oxidative stress, DNA damage, and transcriptional expression of DNA repair and stress response genes in diabetes mellitus. In: Passos GA, editor. Transcriptomics in Health and Disease. Springer International Publishing, Cham (2022). p. 341–65.

[46] Zampieri M Karpach K Salerno G Raguzzini A Barchetta I Cimini FA. Par level mediates the link between ros and inflammatory response in patients with type 2 diabetes mellitus. Redox Biol. (2024) 75:103243. doi: 10.1016/j.redox.2024.103243 38906011 PMC11253151

[47] Rains JL Jain SK. Oxidative stress, insulin signaling, and diabetes. Free Radical Biol Med. (2011) 50:567–75. doi: 10.1016/j.freeradbiomed.2010.12.006 PMC3557825 21163346

[48] Wang N Zhang C. Oxidative stress: A culprit in the progression of diabetic kidney disease. Antioxidants (Basel Switzerland). (2024) 13:455. doi: 10.3390/antiox13040455 38671903 PMC11047699

[49] Bejarano E Taylor A. Too sweet: problems of protein glycation in the eye. Exp eye Res. (2019) 178:255–62. doi: 10.1016/j.exer.2018.08.017 PMC8351608 30145354

[50] Tang G Li S Zhang C Chen H Wang N Feng Y. Clinical efficacies, underlying mechanisms and molecular targets of chinese medicines for diabetic nephropathy treatment and management. Acta Pharm Sin B. (2021) 11:2749–67. doi: 10.1016/j.apsb.2020.12.020 PMC8463270 34589395

[51] Xu J Chen LJ Yu J Wang HJ Zhang F Liu Q. Involvement of advanced glycation end products in the pathogenesis of diabetic retinopathy. Cell Physiol Biochem. (2018) 48:705–17. doi: 10.1159/000491897 30025404

[52] Pathak D Gupta A Kamble B Kuppusamy G Suresh B. Oral targeting of protein kinase C receptor: promising route for diabetic retinopathy? Curr Drug Delivery. (2012) 9:405–13. doi: 10.2174/156720112801323080 22520069

[53] Fahmideh F Marchesi N Campagnoli LIM Landini L Caramella C Barbieri A. Effect of troxerutin in counteracting hyperglycemia-induced vegf upregulation in endothelial cells: A new option to target early stages of diabetic retinopathy? Front Pharmacol. (2022) 13:951833. doi: 10.3389/fphar.2022.951833 36046820 PMC9420903

[54] Jiang L Zhao Y. The value of color doppler ultrasound in the diagnosis of lower extremity vascular disease in type 2 diabetes and an analysis of related factors. Minerva Endocrinol. (2017) 42:223–7. doi: 10.23736/S0391-1977.16.02352-X 28627865

[55] Kim BJ Jin HK Bae JS. Bone marrow-derived mesenchymal stem cells improve the functioning of neurotrophic factors in a mouse model of diabetic neuropathy. Lab Anim Res. (2011) 27:171–6. doi: 10.5625/lar.2011.27.2.171 PMC3146005 21826178

[56] Shao Y Li XR Wood JW Ma JX. Mitochondrial dysfunctions, endothelial progenitor cells and diabetic retinopathy. J Diabetes Complicat. (2018) 32:966–73. doi: 10.1016/j.jdiacomp.2018.06.015 30068485

[57] Kim KA Shin YJ Akram M Kim ES Choi KW Suh H. High glucose condition induces autophagy in endothelial progenitor cells contributing to angiogenic impairment. Biol Pharm Bull. (2014) 37:1248–52. doi: 10.1248/bpb.b14-00172 24989016

[58] Tonade D Liu H Palczewski K Kern TS. Photoreceptor cells produce inflammatory products that contribute to retinal vascular permeability in a mouse model of diabetes. Diabetologia. (2017) 60:2111–20. doi: 10.1007/s00125-017-4381-5 PMC5660634 28755268

[59] Tonade D Liu H Kern TS. Photoreceptor cells produce inflammatory mediators that contribute to endothelial cell death in diabetes. Invest Ophthalmol Vis Sci. (2016) 57:4264–71. doi: 10.1167/iovs.16-19859 PMC5015981 27548900

[60] Lin Y Du W Fu X Huang L Hong Y Tan H. Hyperglycemia-independent neonatal streptozotocin-induced retinopathy (Nsir) in rats. Front Pharmacol. (2024) 15:1395887. doi: 10.3389/fphar.2024.1395887 39108749 PMC11300211

[61] Tonneijck L Muskiet MH Smits MM van Bommel EJ Heerspink HJ van Raalte DH. Glomerular hyperfiltration in diabetes: mechanisms, clinical significance, and treatment. J Am Soc Nephrol. (2017) 28:1023–39. doi: 10.1681/ASN.2016060666 PMC5373460 28143897

[62] Koszegi S Molnar A Lenart L Hodrea J Balogh DB Lakat T. Raas inhibitors directly reduce diabetes-induced renal fibrosis via growth factor inhibition. J Physiol. (2019) 597:193–209. doi: 10.1113/JP277002 30324679 PMC6312411

[63] Hallan S Sharma K. The role of mitochondria in diabetic kidney disease. Curr Diabetes Rep. (2016) 16:61. doi: 10.1007/s11892-016-0748-0 27155611

[64] Patel SG Hsu JW Jahoor F Coraza I Bain JR Stevens RD. Pathogenesis of a⁻Β⁺ Ketosis-prone diabetes. Diabetes. (2013) 62:912–22. doi: 10.2337/db12-0624 PMC3581228 23160531

[65] Jiang X Xu Q Zhang A Liu Y Li Z Tang H. Revealing the hypoglycemic effects and mechanism of gaba-rich germinated adzuki beans on T2dm mice by untargeted serum metabolomics. Front Nutr. (2021) 8:791191. doi: 10.3389/fnut.2021.791191 34970582 PMC8712313

[66] Lehmann R. From bedside to bench-practical considerations to avoid pre-analytical pitfalls and assess sample quality for high-resolution metabolomics and lipidomics analyses of body fluids. Analytical bioanalytical Chem. (2021) 413:5567–85. doi: 10.1007/s00216-021-03450-0 PMC8410705 34159398

[67] Vessby B Tengblad S Lithell H. Insulin sensitivity is related to the fatty acid composition of serum lipids and skeletal muscle phospholipids in 70-year-old men. Diabetologia. (1994) 37:1044–50. doi: 10.1007/BF00400468 7851683

[68] Wang Y Simar D Fiatarone Singh MA. Adaptations to exercise training within skeletal muscle in adults with type 2 diabetes or impaired glucose tolerance: a systematic review. Diabetes Metab Res Rev. (2009) 25:13–40. doi: 10.1002/dmrr.v25:1 19143033

[69] Lin XH Xu MT Tang JY Mai LF Wang XY Ren M. Effect of intensive insulin treatment on plasma levels of lipoprotein-associated phospholipase A2 and secretory phospholipase A2 in patients with newly diagnosed type 2 diabetes. Lipids Health Dis. (2016) 15:203. doi: 10.1186/s12944-016-0368-3 27881128 PMC5120429

[70] Chen T Ni Y Ma X Bao Y Liu J Huang F. Branched-chain and aromatic amino acid profiles and diabetes risk in chinese populations. Sci Rep. (2016) 6:20594. doi: 10.1038/srep20594 26846565 PMC4742847

[71] Tulipani S Palau-Rodriguez M Minarro Alonso A Cardona F Marco-Ramell A Zonja B. Biomarkers of morbid obesity and prediabetes by metabolomic profiling of human discordant phenotypes. Clin Chim Acta. (2016) 463:53–61. doi: 10.1016/j.cca.2016.10.005 27720726

[72] Rivas-Tumanyan S Pacheco LS Haslam DE Morou-Bermudez E Liang L Tucker KL. Branched-chain and aromatic amino acids, type 2 diabetes, and cardiometabolic risk factors among puerto rican adults. Nutrients. (2024) 16:2562. doi: 10.3390/nu16152562 39125441 PMC11313859

[73] Hu W Sun L Gong Y Zhou Y Yang P Ye Z. Relationship between branched-chain amino acids, metabolic syndrome, and cardiovascular risk profile in a chinese population: A cross-sectional study. Int J Endocrinol. (2016) 2016:8173905. doi: 10.1155/2016/8173905 27528871 PMC4977397

[74] Wang Q Holmes MV Davey Smith G Ala-Korpela M. Genetic support for a causal role of insulin resistance on circulating branched-chain amino acids and inflammation. Diabetes Care. (2017) 40:1779–86. doi: 10.2337/dc17-1642 PMC5701741 29046328

[75] Kalapos MP. Methylglyoxal in living organisms. Toxicol Lett. (1999) 110:145–75. doi: 10.1016/s0378-4274(99)00160-5 10597025

[76] Tillin T Hughes AD Wang Q Wurtz P Ala-Korpela M Sattar N. Diabetes risk and amino acid profiles: cross-sectional and prospective analyses of ethnicity, amino acids and diabetes in a south asian and european cohort from the sabre (Southall and brent revisited) study. Diabetologia. (2015) 58:968–79. doi: 10.1007/s00125-015-3517-8 PMC4392114 25693751

[77] Wang TJ Larson MG Vasan RS Cheng S Rhee EP McCabe E. Metabolite profiles and the risk of developing diabetes. Nat Med. (2011) 17:448–53. doi: 10.1038/nm.2307 PMC3126616 21423183

[78] Lotta LA Scott RA Sharp SJ Burgess S Luan J Tillin T. Genetic predisposition to an impaired metabolism of the branched-chain amino acids and risk of type 2 diabetes: A mendelian randomisation analysis. PLoS Med. (2016) 13:e1002179. doi: 10.1371/journal.pmed.1002179 27898682 PMC5127513

[79] Jang C Oh SF Wada S Rowe GC Liu L Chan MC. A branched-chain amino acid metabolite drives vascular fatty acid transport and causes insu- lin resistance. Nat Med. (2016) 22:421–6. doi: 10.1038/nm.4057 PMC4949205 26950361

[80] Winiarska K Szymanski K Gorniak P Dudziak M Bryla J. Hypoglycaemic, antioxidative and nephroprotective effects of taurine in alloxan diabetic rabbits. Biochimie. (2009) 91:261–70. doi: 10.1016/j.biochi.2008.09.006 18957317

[81] O'Connell TM. The complex role of branched chain amino acids in diabetes and cancer. Metabolites. (2013) 3:931–45. doi: 10.3390/metabo3040931 PMC3937834 24958258

[82] Sas KM Karnovsky A Michailidis G Pennathur S. Metabolomics and diabetes: analytical and computational approaches. Diabetes. (2015) 64:718–32. doi: 10.2337/db14-0509 PMC4338589 25713200

[83] Shang W Yasuda K Takahashi A Hamasaki A Takehiro M Nabe K. Effect of high dietary fat on insulin secretion in genetically diabetic goto-kakizaki rats. Pancreas. (2002) 25:393–9. doi: 10.1097/00006676-200211000-00012 12409835

[84] Artwohl M Roden M Waldhausl W Freudenthaler A Baumgartner-Parzer SM. Free fatty acids trigger apoptosis and inhibit cell cycle progression in human vascular endothelial cells. FASEB J. (2004) 18:146–8. doi: 10.1096/fj.03-0301fje 14597560

[85] Kwon G Pappan KL Marshall CA Schaffer JE McDaniel ML. Camp dose-dependently prevents palmitate-induced apoptosis by both protein kinase a- and camp-guanine nucleotide exchange factor-dependent pathways in beta-cells. J Biol Chem. (2004) 279:8938–45. doi: 10.1074/jbc.M310330200 14688288

[86] Kim JE Song SE Kim YW Kim JY Park SC Park YK. Adiponectin inhibits palmitate-induced apoptosis through suppression of reactive oxygen species in endothelial cells: involvement of camp/protein kinase a and amp-activated protein kinase. J Endocrinol. (2010) 207:35–44. doi: 10.1677/JOE-10-0093 20675307

[87] Dhatariya KK Glaser NS Codner E Umpierrez GE. Diabetic ketoacidosis. Nat Rev Dis Primers. (2020) 6:40. doi: 10.1038/s41572-020-0165-1 32409703

[88] Gou X Li G-P Zhang C Cheng W Chen F Wang H. Urinary metabolomics study in patients with diabetic peripheral neuropathy. Chin J Hosp Pharm. (2019) 039:2512–9. doi: 10.13286/j.cnki.chinhosppharmacyj.2019.24.10

[89] Qf S. Relationship between free fatty acid and peripheral neuropathy in type 2 diabetes mellitus and its mechanism. Doctor thesis of Hebei Medical University, Shijiazhuang, China (2009).

[90] Breit M Netzer M Weinberger KM Baumgartner C. Modeling and classification of kinetic patterns of dynamic metabolic biomarkers in physical activity. PLoS Comput Biol. (2015) 11:e1004454. doi: 10.1371/journal.pcbi.1004454 26317529 PMC4552566

[91] Jahoor F Hsu JW Mehta PB Keene KR Gaba R Mulukutla SN. Metabolomics profiling of patients with a-Β+ Ketosis-prone diabetes during diabetic ketoacidosis. Diabetes. (2021) 70:1898–909. doi: 10.2337/db21-0066 PMC8385613 34021044

[92] Villarreal-Perez JZ Villarreal-Martinez JZ Lavalle-Gonzalez FJ Torres-Sepulveda-Mdel R Ruiz-Herrera C Cerda-Flores RM. Plasma and urine metabolic profiles are reflective of altered beta-oxidation in non-diabetic obese subjects and patients with type 2 diabetes mellitus. Diabetol Metab Syndr. (2014) 6:129. doi: 10.1186/1758-5996-6-129 25937838 PMC4416397

[93] t Hart LM Hansen T Rietveld I Dekker JM Nijpels G Janssen GM. Evidence that the mitochondrial leucyl trna synthetase (Lars2) gene represents a novel type 2 diabetes susceptibility gene. Diabetes. (2005) 54:1892–5. doi: 10.2337/diabetes.54.6.1892 15919814

[94] Florentz C. Molecular investigations on trnas involved in human mitochondrial disorders. Biosci Rep. (2002) 22:81–98. doi: 10.1023/a:1016065107165 12418552

[95] Florentz C Sohm B Tryoen-Toth P Putz J Sissler M. Human mitochondrial trnas in health and disease. Cell Mol Life Sci. (2003) 60:1356–75. doi: 10.1007/s00018-003-2343-1 PMC11138538 12943225

[96] Park SG Kang YS Kim JY Lee CS Ko YG Lee WJ. Hormonal activity of aimp1/P43 for glucose homeostasis. Proc Natl Acad Sci U.S.A. (2006) 103:14913–8. doi: 10.1073/pnas.0602045103 PMC1595450 17001013

[97] Wright CE Tallan HH Lin YY Gaull GE. Taurine: biological update. Annu Rev Biochem. (1986) 55:427–53. doi: 10.1146/annurev.bi.55.070186.002235 3527049

[98] Stevens MJ Lattimer SA Kamijo M Van Huysen C Sima AA Greene DA. Osmotically-induced nerve taurine depletion and the compatible osmolyte hypothesis in experimental diabetic neuropathy in the rat. Diabetologia. (1993) 36:608–14. doi: 10.1007/BF00404069 8359577

[99] Askwith T Zeng W Eggo MC Stevens MJ. Oxidative stress and dysregulation of the taurine transporter in high-glucose-exposed human schwann cells: implications for pathogenesis of diabetic neuropathy. Am J Physiol Endocrinol Metab. (2009) 297:E620–8. doi: 10.1152/ajpendo.00287.2009 PMC3833996 19602579

[100] Stevens MJ Hosaka Y Masterson JA Jones SM Thomas TP Larkin DD. Downregulation of the human taurine transporter by glucose in cultured retinal pigment epithelial cells. Am J Physiol. (1999) 277:E760–71. doi: 10.1152/ajpendo.1999.277.4.E760 10516137

[101] Li F Obrosova IG Abatan O Tian D Larkin D Stuenkel EL. Taurine replacement attenuates hyperalgesia and abnormal calcium signaling in sensory neurons of stz-D rats. Am J Physiol Endocrinol Metab. (2005) 288:E29–36. doi: 10.1152/ajpendo.00168.2004 15585600

[102] Obrosova IG Fathallah L Stevens MJ. Taurine counteracts oxidative stress and nerve growth factor deficit in early experimental diabetic neuropathy. Exp Neurol. (2001) 172:211–9. doi: 10.1006/exnr.2001.7789 11681853

[103] Obrosova IG Minchenko AG Marinescu V Fathallah L Kennedy A Stockert CM. Antioxidants attenuate early up regulation of retinal vascular endothelial growth factor in streptozotocin-diabetic rats. Diabetologia. (2001) 44:1102–10. doi: 10.1007/s001250100631 11596663

[104] Agca CA Tuzcu M Hayirli A Sahin K. Taurine ameliorates neuropathy via regulating nf-kappab and nrf2/ho-1 signaling cascades in diabetic rats. Food Chem Toxicol. (2014) 71:116–21. doi: 10.1016/j.fct.2014.05.023 24907624

[105] Wang P Yang X Zhang Z Song J Guan YF Zou DJ. Depletion of nad pool contributes to impairment of endothelial progenitor cell mobilization in diabetes. Metabolism. (2016) 65:852–62. doi: 10.1016/j.metabol.2016.03.006 27173464

[106] Van Hove I De Groef L Boeckx B Modave E Hu TT Beets K. Single-cell transcriptome analysis of the akimba mouse retina reveals cell-type-specific insights into the pathobiology of diabetic retinopathy. Diabetologia. (2020) 63:2235–48. doi: 10.1007/s00125-020-05218-0 32734440

[107] Wang C Yu J Zhang R Wang W Shi Z Liu Y. Small intestine proteomics coupled with serum metabolomics reveal disruption of amino acid metabolism in chinese hamsters with type 2 diabetes mellitus. J Proteomics. (2020) 223:103823. doi: 10.1016/j.jprot.2020.103823 32428569

[108] Kikuchi K Saigusa D Kanemitsu Y Matsumoto Y Thanai P Suzuki N. Gut microbiome-derived phenyl sulfate contributes to albuminuria in diabetic kidney disease. Nat Commun. (2019) 10:1835. doi: 10.1038/s41467-019-09735-4 31015435 PMC6478834

[109] Shi X Xi B Jasbi P Turner C Jin Y Gu H. Comprehensive isotopic targeted mass spectrometry: reliable metabolic flux analysis with broad coverage. Anal Chem. (2020) 92:11728–38. doi: 10.1021/acs.analchem.0c01767 PMC7546585 32697570

[110] Chavez-Pineda OG Rodriguez-Moncayo R Cedillo-Alcantar DF Guevara-Pantoja PE Amador-Hernandez JU Garcia-Cordero JL. Microfluidic systems for the analysis of blood-derived molecular biomarkers. Electrophoresis. (2022) 43:1667–700. doi: 10.1002/elps.202200067 35767850

[111] Klein MS Shearer J. Metabolomics and type 2 diabetes: translating basic research into clinical application. J Diabetes Res. (2016) 2016:3898502. doi: 10.1155/2016/3898502 26636104 PMC4655283

[112] Zhu L Huang Q Li X Jin B Ding Y Chou CJ. Serological phenotyping analysis uncovers a unique metabolomic pattern associated with early onset of type 2 diabetes mellitus. Front Mol Biosci. (2022) 9:841209. doi: 10.3389/fmolb.2022.841209 35463946 PMC9024215

