# Supplementary material for: Clinical metabolomics in type 2 diabetes mellitus: from pathogenesis to biomarkers
Source: Front Endocrinol (Lausanne). 2025 Feb 25;16:1501305. doi: 10.3389/fendo.2025.1501305 (PMC11893406; doi:10.3389/fendo.2025.1501305)
Supplement: Supplementary file 1 [file SupplementaryFile1.doc]

**Clinical metabolomics in type 2 diabetes mellitus: from pathogenesis to biomarkers**

Jiao Kong^2,†^，Hetao Chen^1,3,†^，Yujin Ma^1^，Lei Zhang^4^，Lulu Chen^1,3^，Jiarui Huang^5^, Zizhe Zhao^1^, Hongwei Jiang^1,*^, Chuanxin Liu^1,*^

^1^ Luoyang Key Laboratory of Clinical Multiomics and Translational Medicine, Henan Key Laboratory of Rare Diseases, Endocrinology and Metabolism Center, The First Affiliated Hospital, and College of Clinical Medicine of Henan University of Science and Technology, Luoyang, China,

^2^ Institute of Drug Metabolism and Pharmaceutical Analysis, College of Pharmaceutical Sciences, Zhejiang University, Hangzhou, China,

^3^ Department of Clinical Laboratory, The First Affiliated Hospital, College of Clinical Medicine of Henan University of Science and Technology, Luoyang, China,

^4^ Department of Integrative Medicine, The First Affiliated Hospital, and College of Clinical Medicine of Henan University of Science and Technology, Luoyang, China,

^5^ Department of Critical Care Medicine, The First Affiliated Hospital, and College of Clinical Medicine of Henan University of Science and Technology, Luoyang, China

^†^ These authors contributed equally to this work and share first authorship

* Correspondence: Hongwei Jiang✉: jianghw@haust.edu.cn; Chuanxin Liu✉:15222003775@163.com.

**Contents:**

**Method S1: Literature retrieval strategy**

**Method S2: Detailed inclusion criteria and exclusion criteria**

**Method S3: Literature retrieval and inclusion results**

**Method S4: Baseline characteristic**

**Method S1: Literature retrieval strategy**

**1.1 Type 2 diabetes mellitus**

Retrieval strategy(China National Knowledge Infrastructure, WANGFANG, VIP): (published between (2000-01-01, 2020-01-01) and (subject = synonym extension (diabetes)) or (title = synonym extension (diabetes)) or (v_subject = synonym extension (diabetes) or (v_ Subject = synonym extension (diabetes, both Chinese and English)) or (title = Chinese English extension (diabetes)) or (title = Chinese English extension (metabolomics)) or (title = synonym extension (metabolomics)) or (v_subject = synonym extension (metabolomics)) or (v_subject = synonym extension (metabolomics, Chinese English control)) or (title = Chinese English extension (metabolomics)) or (title = Chinese English extension (metabolomics, Chinese English control)) (fuzzy matching).

Retrieval strategy(PubMed, The Cochrane Library): ("Metabolomics"[Mesh] AND "Diabetes Mellitus"[Mesh]) AND (Clinical Study[ptyp]) AND ("2000/01/01"[PDAT]: "2020/01/01"[PDAT]) AND (English[lang]) AND ("adult"[MeSH Terms]).

**1.2 Type 2 diabetic ketosis**

Retrieval strategy(China National Knowledge Infrastructure, WANGFANG, VIP):(published between (2000-01-01, 2020-01-01) and (subject = synonym extension (diabetic ketosis)) or (title = synonym extension (diabetic ketosis)) or (v_subject = synonym extension (diabetic ketosis)) or (v_subject = synonym extension (diabetic ketosis, Chinese and English)) or (title = Chinese English extension (diabetic ketosis)) or (title = synonym extension (metabolomics)) or (title = synonym extension (metabolomics)) or (v_subject = synonym extension (metabolomics) or (v_subject = synonym extension (metabolomics, Chinese and English)) or (title = Chinese English extension (metabolomics, Chinese English control)) (fuzzy matching).

Retrieval strategy(PubMed, The Cochrane Library): ("Diabetic Ketoacidosis"[Mesh]) AND ("Metabolomics"[Mesh]).

**1.3 Type 2** **diabetic peripheral neuropathy**

Retrieval strategy(China National Knowledge Infrastructure, WANGFANG, VIP): (published between (2000-01-01, 2020-01-01)) and (subject = synonym extension (diabetic peripheral neuropathy)) or (title = synonym extension (diabetic peripheral neuropathy)) or (v_subject = synonym extension (diabetic peripheral neuropathy)) or (v_subject = synonym extension (diabetic peripheral neuropathy, Chinese English control)) or (title = Chinese English extension (diabetic peripheral neuropathy)) or (title = synonym extension (metabolomics)) or (title = synonym extension (metabolomics)) or (title = synonym extension (metabolomics)) or (v_subject = synonym extension (metabolomics)) or (v_subject = synonym extension (metabolomics, Chinese English control)) or (title = Chinese English extension (metabolomics)) or (title = Chinese English extension (metabolomics, Chinese English control)) (fuzzy matching).

Retrieval strategy(PubMed, The Cochrane Library): (Search: ("Metabolomics"[Mesh]) AND ("Diabetic Neuropathies"[Mesh]) Filters: (Clinical Study, Adult: 19+ years, English).

**1.4 Type 2 diabetic retinopathy**

Retrieval strategy(China National Knowledge Infrastructure, WANGFANG, VIP):(published between (2000-01-01, 2020-01-01)) and (subject = synonym extension (diabetic retinopathy)) or (title = synonym extension (diabetic retinopathy)) or (v_subject = synonym extension (diabetic retinopathy)) or (v_subject = synonym extension (diabetic retinopathy, Chinese and English)) or (title = Chinese-English extended (diabetic retinopathy)) or (title = synonym extension (metabolomics)) or (title = synonym extension (metabolomics)) or (v_subject = synonym extension (metabolomics)) or (v_subject = synonym extension (metabolomics, Chinese-English control)) or (title = Chinese-English extension (metabolomics)) or (title = Chinese-English extension (metabolomics, Chinese English control)) (fuzzy matching).

Retrieval strategy(PubMed, The Cochrane Library): (Search: ("Metabolomics"[Mesh]) AND ("Diabetic Retinopathy"[Mesh]) Filters: (Adult: 19+ years, English).

**1.5 Type 2** **diabetic nephropathy**

Retrieval strategy(China National Knowledge Infrastructure, WANGFANG, VIP):(published between (2000-01-01, 2020-01-01)) AND (subject = synonym extension (diabetic nephropathy)) OR (title = synonym extension(diabetic nephropathy)) OR (v_subject = synonym extension (diabetic nephropathy)) OR (v_subject = synonym extension (diabetic nephropathy, Chinese AND English)) OR (title = Chinese English extension (diabetic nephropathy)) OR (title = synonym extension (metabolomics)) OR (title = synonym extension (metabolomics)) OR (v_subject = synonym extension (metabolomics)) OR (v_subject = synonym extension (metabolomics, Chinese English control)) OR (title = Chinese English extension (metabolomics)) OR (title = Chinese English extension (metabolomics, Chinese English control)) (fuzzy matching).

Retrieval strategy(PubMed, The Cochrane Library): ("Metabolomics"[MeSH Terms]) AND ("Diabetic Nephropathies"[MeSH Terms]).

**Method S2 Inclusion and exclusion criteria**

**2.1 Inclusion criteria**

**2.1.1 PM**

A study would be included in the review if it satisfied the following predefined criteria:

(1) Patients’ symptoms in the literature must fulfill the diagnostic criteria of prediabetes, namely, impaired fasting glucose (IFG) or impaired glucose tolerance (IGT) or impaired fasting glucose with impaired glucose tolerance (IFG + IGT). IFG was defined as a fasting blood glucose level of 100 mg/dl (5.6 mmol/L) to 125 mg/dl (6.9 mmol/L); IGT was defined based on the standard 75 g glucose tolerance test (OGTT). The 2-hour postprandial blood glucose level was 140 mg/dl (7.8 mmol/L) to 199 mg/dl(11.0 mmol/L); (2) there were no strict restrictions on age and gender.

(3) There was a clear, healthy control group.

(4) Urine or blood sample.

(5) The clinical design included cross-sectional, case-control, and prospective cohort studies.

(6) In a randomized controlled trial, a healthy control group was set up separately.

**2.1.2 T2DM**

A study would be included in the review if it satisfied the following criteria:

(1) Patients’ symptoms in the literature must fulfill the diabetes diagnostic criteria published by the World Health Organization in1999(1), namely, a fasting blood glucose level greater than 7.0 mmol/L (126 mg/dL) and postprandial blood glucose ≥11.1 mmol/L (200 mg/dL).

(2) All study populations had no strict restrictions on age, gender, and course of diabetes.

(3) There is a clear, healthy control group.

(4) The source of the sample was blood, urine, or tissue.

(5) The clinical design was a cross-sectional, case-control, prospective cohort observational study.

(6) A randomized controlled trial established a healthy control group.

**2.1.3 T2DK**

A study would be included in the review if it satisfied the following predefined criteria:

(1) Patients’ symptoms in the literature must fulfill the clinical diagnostic criteria for type 2 diabetes mellitus. The clinical diagnosis of type 2 diabetes ketosis refers to the American Diabetes Association criteria(2), namely, fasting blood glucose level ≥7.0 mmol/L, whole blood pH <7.30 or serum bicarbonate level <18 mmol/L, blood glucose level >11.1 mmol/L (200 mg/dL), and the urine ketone body is positive by routine urine examination.

(2) All study populations had no strict restrictions on age, gender, and course of diabetes.

(3) There is a clear, healthy control group.

(4) The source of the sample was blood, urine, or tissue.

(5) The clinical design was a cross-sectional, case-control, prospective cohort observational study.

(6) A randomized controlled trial established a healthy control group.

**2.1.4 T2DPN**

(1) Patients’ symptoms in the literature must fulfill the clinical diagnostic criteria for type 2 diabetic peripheral neuropathy. The clinical diagnosis of type 2 diabetic peripheral neuropathy refers to the modified Neuropathy Disability Score and Vibration Perception Threshold (3, 4), that is, a fasting blood glucose level of ≥7.0 mmol/L. Patients have severe peripheral sensory defects (acupuncture, temperature, or vibration).

(2) All study populations had no strict restrictions on age, gender, and course of diabetes.

(3) There is a clear, healthy control group.

(4) The source of the sample was blood, urine, or tissue.

(5) The clinical design was a cross-sectional, case-control, prospective cohort observational study.

(6) A randomized controlled trial established a healthy control group.

**2.1.5 T2DR**

(1) Patients’ symptoms in the literature must fulfill the clinical diagnostic criteria for type 2 diabetic retinopathy. The clinical diagnosis of type 2 diabetic retinopathy was based on the American Diabetes Association criteria (5), namely, fasting blood glucose level ≥ 7.0 mmol / L and at least one eye diagnosed as having diabetic retinopathy by fundus microscopy, fundus color photography, and angiography. Diagnosis of non-proliferative lesions is based on macular hemorrhage, microaneurysms, cotton wadding spots, or retinal microvascular abnormalities; diagnosis of proliferative diseases is based on the existence of iris or retinal neovascularization; clinical staging of type 2 diabetic retinopathy refers to the classification standard of diabetic retinopathy formulated by the fundus disease group of the Chinese Medical Association(6). And according to the fundus changes, diabetic retinopathy can be were divided into the non-proliferative type and proliferative type. From stages 1 to 6, the disease gradually worsened. In stage I, there was microaneurysms or small bleeding spots in the retina. In stage 2, there was yellow white "hard exudation" in the retina. In stage 2I, the retina had gray white "soft exudation" or combined with the stage I or Ⅱ lesions; in stage IV, retinal neovascularization or vitreous hemorrhage; in stage V, retinal neovascularization and fiber proliferation; and in stage VI, retinal neovascularization and fiber proliferation, leading to retinal detachment.

(2) There were no strict restrictions on age, sex, and duration of diabetes mellitus in all study groups.

(3) There was a clear, healthy control group.

(4) Samples were collected from blood, urine, or tissue.

(5) The clinical design included cross-sectional, case-control, and prospective cohort studies.

(6) In a randomized controlled trial, a healthy control group was set up separately.

**2.1.6 T2DN**

(1) Patients’ symptoms in the literature must fulfill the clinical diagnostic criteria for type 2 diabetic nephropathy. The clinical diagnosis of type 2 diabetic retinopathy was based on the American Diabetes Association criteria (7-9), namely, fasting blood glucose level ≥ 7.0 mmol/L, urinary albumin/creatinine (ACR) higher than 30 mg/g for more than 3 months, or estimated glomerular filtration rate eGFR < 60 ml/min/1.73M2.

(2) There were no strict restrictions on age, sex, and duration of diabetes mellitus in all study groups.

(3) There was a clear, healthy control group.

(4) Samples were collected from blood, urine, or tissue.

(5) The clinical design included cross-sectional, case-control, and prospective cohort studies.

(6) In a randomized controlled trial, a healthy control group was set up separately.

**2.2 Exclusion criteria**

**2.2.1 PM**

(1) Animal experiments, literature review, repeated collection, and published literature

(2) Methods for non-metabolomics

(3) Classification or research of TCM(Traditional Chinese Medicine) syndrome types

**2.2.2 T2DM**

(1) Animal experiments, literature review, repeated collection, and published literature

(2) Methods for non-metabolomics

(3) Type I diabetes mellitus, gestational diabetes mellitus, and special diabetes mellitus

(4) Classification and research on TCM syndrome types

**2.2.3 T2DK**

(1) Animal experiments, literature review, repeated collection, and published literature

(2) Methods for non-metabolomics

(3) Type I diabetic ketosis, gestational diabetes ketosis, special diabetic ketosis-related research

(4) Classification and research on TCM syndrome types

**2.4 T2DPN**

(1) Animal experiments, literature review, repeated collection, and published literature

(2) Methods of non-metabolomics

(3) Type I diabetic peripheral neuropathy, pregnancy peripheral neuropathy, special diabetic peripheral neuropathy

(4) Other non-high glucose-induced neuropathy-related research

(5) Classification or research of TCM syndrome types

**2.2.5** **T2DR**

(1) Animal experiments, literature review, repeated collection, and published literature

(2) Methods for non-metabolomics

(3) Related research on type I diabetic retinopathy, gestational diabetic retinopathy, and special diabetic retinopathy

(4) Other non-high glucose-induced retinopathy (e.g., uvea, optic neuritis, glaucoma).

(5) Classification and research on TCM syndrome types

**2.2.6 T2DN**

(1) Animal experiments, literature review, repeated collection, and published literature

(2) Methods for non-metabolomics

(3) Related research on type I diabetic nephropathy, gestational diabetic nephropathy, and special diabetic nephropathy

(4) Other non-high glucose-induced retinopathy (e.g., uvea, optic neuritis, glaucoma)

(5) Classification and research on TCM syndrome types

**2.3 Literature Searching Strategy**

To retrieve and include papers, we completed five separate and sequential literature searches using PubMed (http://www.ncbi.nlm.nih.gov/pubmed), the Cochrane Library (https://www.cochrane.org/welcome), China National Knowledge Infrastructure Database (https://www.cnki.net/), WangFang Database (http://www.wanfangdata.com.cn/index.html), and VIP Database (http://www.cqvip.com/). The aim of the first search was to find all related free words (synonyms) according to the subject words. Next, we used the search term “#1 OR #2 OR #3 AND *1 OR *2 OR*3,” “#MeSH AND *MeSH” (#: Subject words and free words of type 2 diabetes and its complications；* Subject words and free words in metabolomics). The retrieval time of the literature was set from January 1, 2000, to January 1, 2020, and the clinical research depended on the official publication time of the literature.

**2.4** **Data Extraction**

The initial literature screening process was conducted by reviewing the titles and abstracts. Subsequently, full-text versions of potential articles were obtained for further assessment. Next, data were extracted following the pre-designed form, including the title, name of the first author and corresponding author, publication institution, publication year, patient characteristics (sample size, sex, age, patient’s baseline, and sample category), study design, and domains of risk of bias. Finally, biomarkers for the early diagnosis of type 2 diabetes and its complications were identified.

**2.5 Literature quality assessment**

The Cochrane Collaboration risk of bias tool was used to evaluate the baseline indicators of the eligibility of the included studies. The following items were assessed: 1) sample size of each group, 2) age, and 3) sex. If the baseline basic indicators of included research are complete and there is no statistical difference, the quality of literature can be rated as "low risk" (indicating low bias risk). And the quality of literature can be rated as "high risk" (high bias risk) if any one of the three items with a statistical difference, and it can be rated as "unclear" if the data are incomplete.

**Method S3 Literature retrieval and inclusion results**

**3.1 Type 2 prediabetes mellitus**

A total of 339 records were identified from the five databases. Then, 42 records were removed for duplication, and 212 records were excluded through screening titles and abstracts because they were irrelevant studies, reviews and animal experiments. The full texts of the remaining records were screened, and 62 records were excluded for the eligibility of abovementioned exclusion criteria. Ultimately, 23 literatures were included in this review. They were all published from 2000 to 2020. The process and results of literature screening are shown in Figure 1.

**3.2 Type 2 diabetes mellitus**

A total of 1005 records were identified from the five databases. Then, 164 records were removed for duplication, and 800 records were excluded through screening titles and abstracts because they were irrelevant studies, reviews and animal experiments. The full texts of the remaining records were screened, and 3 records were excluded for the eligibility of abovementioned exclusion criteria. Ultimately, 38 literatures were included in this review. They were all published from 2000 to 2020. The process and results of literature screening are shown in Figure 1.

**3.3** **Type 2 diabetic ketosis**

A total of 7 records were identified from the five databases. Then, 3 records were removed for duplication, and 3 records were excluded through screening titles and abstracts because they were irrelevant studies, reviews and animal experiment and the eligibility of abovementioned exclusion criteria. Ultimately, 1 literature were included in this review. They were all published from 2000 to 2020. The process and results of literature screening are shown in Figure 1.

**3.4** **Type 2 diabetic peripheral neuropathy**

A total of 11 records were identified from the five databases. Then, 3 records were removed for duplication, and 13 records were excluded through screening titles and abstracts because they were irrelevant studies, reviews and animal experiment and the eligibility of abovementioned exclusion criteria. Ultimately, only 1 literature were included in this review. They were all published from 2000 to 2020. The process and results of literature screening are shown in Figure 1.

**3.5** **Type 2 diabetic retinopathy**

A total of 21 records were identified from the five databases. Then, 3 records were removed for duplication, and 14 records were excluded through screening titles and abstracts because they were irrelevant studies, reviews and animal experiment and the eligibility of abovementioned exclusion criteria. Ultimately, 4 literatures were included in this review. They were all published from 2000 to 2020. The process and results of literature screening are shown in Figure 1.

**3.6** **Type 2 diabetic nephropathy**

A total of 295 records were identified from the five databases. Then, 32 records were removed for duplication, and 238 records were excluded through screening titles and abstracts because they were irrelevant studies, reviews and animal experiments. The full texts of the remaining records were screened, and 8 records were excluded for the eligibility of above mentioned exclusion criteria. Ultimately, 17 literatures were included in this review. They were all published from 2000 to 2020. The process and results of literature screening are shown in Figure 1.

**Method S4 Baseline characteristic**

**4.1 Type 2 prediabetes mellitus**

In this study, 23 articles were finally included, involving 4665 subjects, including 2445 cases in the early diabetic group and 2220 cases in the blank control group. Among them, there were 11 articles with no statistical difference in age, gender and baseline (*P* > 0.05) (10-13),(14-20), given "low-risk" bias assessment; 1 literature (21) baseline instability (*P* < 0.05), research results may have a certain risk, given "high-risk" bias; 11 literature (22-32) Clinical baseline information is incomplete, given "unclear" assessment, the specific information is shown in table S1.

**4.2 Type 2 diabetes mellitus**

A total of 39 articles were included in this study, with a total of 24961 subjects, including 2445 cases in the diabetes group and 2220 cases in the control group. Among them, there were 12 articles (33-43) with no significant difference in age, gender and baseline (*P* > 0.05), and 10 articles (44-53), respectively, the baseline was unstable (*P* < 0.05), and the research results may have certain risk, so the bias of "high risk" was given; 17 literatures (46, 54-69) had incomplete clinical baseline information, and were assessed as "unclear". The specific information is shown in table S1.

**4.3 Type 2 diabetic ketosis**

Finally, a literature(70) was included in this study, including 20 cases of diabetic ketosis group and 19 cases of control group. The clinical baseline information was incomplete and was assessed as "unclear". The specific information is shown in table S1.

**4.4 Type 2 diabetic peripheral neuropathy**

Finally, a literature(71) was included in this study, including 40 cases of diabetic peripheral neuropathy and 40 cases of control group. There was no significant difference in age, gender and baseline (*P* > 0.05). The "low risk" bias was assessed. The specific information is shown in table S1.

**4.5 Type 2 diabetic retinopathy**

Four articles(72-75) were included in this study, including 92 cases of diabetic retinopathy group and 90 cases of control group. Two articles (72, 74) had no statistical difference in age, gender and other baseline (*P* > 0.05), so they were given "low-risk" bias assessment; one literature(75) had unstable baseline (*P* < 0.05), and the research results may have certain risks, so they were given "high-risk" bias; one literature had incomplete clinical baseline information and was given "unclear" assessment. The specific information is shown in table S1.

**4.6 Type 2 diabetic nephropathy**

Seventeen articles (76-92) were included in this study, including 1 880 subjects, including 1269 cases of diabetic nephropathy and 611 cases of control group. Among them, 5 articles (76, 79, 82, 85, 92) had no statistical difference in age, gender and other baseline (P > 0.05), and they were given "low-risk" bias assessment; and 9 articles (80, 81, 83, 86-91) the baseline was unstable (P < 0.05), and the research results may have certain risks, so the bias of "high risk" was given; one literature with incomplete clinical baseline information was given "unclear" evaluation, and the specific information is shown in table S1.

**Reference**

1. Mellitus ECotDaCoD. Report of the Expert Committee on the Diagnosis and Classification of Diabetes Mellitus. *Diabetes care* (2003):S5-20. doi: 10.2337/diacare.26.2007.s5.

2. Tiercelin C, Lemoine AY, Ratheau L, Larger E. High Frequency of Transaminase Elevation Following Diabetic Ketoacidosis. *Diabetes Metab* (2021) 47(1):101123. Epub 2019/10/14. doi: 10.1016/j.diabet.2019.09.001.

3. Allet L, Armand S, Aminian K, Pataky Z, Golay A, Bie RAD, et al. An Exercise Intervention to Improve Diabetic Patients' Gait in a Real-Life Environment. *Gait & Posture* (2010) 32(2):185-90.

4. Allet L, Armand S, Bie RAD, Golay A, Monnin D, Aminian K, et al. The Gait and Balance of Patients with Diabetes Can Be Improved: A Randomised Controlled Trial. *Diabetologia* (2010) 53(3):458-66.

5. Sumarriva K, Uppal K, Ma C, Herren DJ, Wang Y, Chocron IM, et al. Arginine and Carnitine Metabolites Are Altered in Diabetic Retinopathy. *Investigative Ophthalmology Visual ence* (2019) 60(8).

6. Oellers P, Mahmoud T. Surgery for Proliferative Diabetic Retinopathy: New Tips and Tricks. *Journal of Ophthalmic Vision Research* (2016) 11(1).

7. Gross JL, de Azevedo MJ, Silveiro SP, Canani LH, Caramori ML, Zelmanovitz T. Diabetic Nephropathy: Diagnosis, Prevention, and Treatment. *Diabetes Care* (2005) 28(1):164-76. Epub 2004/12/24. doi: 10.2337/diacare.28.1.164.

8. Kdoqi. Kdoqi Clinical Practice Guidelines and Clinical Practice Recommendations for Diabetes and Chronic Kidney Disease. *Am J Kidney Dis* (2007) 49(2 Suppl 2):S12-154. Epub 2007/02/06. doi: 10.1053/j.ajkd.2006.12.005.

9. Lin HT, Cheng ML, Lo CJ, Lin G, Liu FC. 1h Nuclear Magnetic Resonance (Nmr)-Based Cerebrospinal Fluid and Plasma Metabolomic Analysis in Type 2 Diabetic Patients and Risk Prediction for Diabetic Microangiopathy. *Journal of Clinical Medicine* (2019) 8(6):874. doi: 10.3390/jcm8060874.

10. Hongfu Z. The Applications of Liquid Chromatography Combined with Mass Spectrometry in Impaired Glucose Tolerance and Liver Cirrhosis: East China University of Science and Technology (2011).

11. Meihua Y. Research on Mechanism of Female Impaired Glucose Regulation (2012).

12. Xiao Ji ZL, Ma Mingkun, Yan Weili, Liu Na, Liu Shuye. Serum Metabonomics in Patients with Abnormal Glucose Metabolism. *Chinese Journal of Clinical Laboratory Science* (2014) 32(012):909-11.

13. Zhengzhen LBJMZNLCWYZXZYW. Effects of Aerobic Exercise on Plasma Metabolites in Prediabetes Subjects. *Chinese Journal of Sports Medicine* (2018) 37(4):301-8.

14. Andersson-Hall U, Gustavsson C, Pedersen A, Malmodin D, Joelsson L, Holmäng A. Higher Concentrations of Bcaas and 3-Hib Are Associated with Insulin Resistance in the Transition from Gestational Diabetes to Type 2 Diabetes. *Journal of diabetes research* (2018) 2018:4207067. doi: 10.1155/2018/4207067.

15. Cobb J, Eckhart A, Motsinger-Reif A, Carr B, Groop L, Ferrannini E. Α-Hydroxybutyric Acid Is a Selective Metabolite Biomarker of Impaired Glucose Tolerance. *Diabetes Care* (2016).

16. Kujala UM, Markku P, Laine MK, Jaakko K, Heinonen OJ, Jouko S, et al. Branched-Chain Amino Acid Levels Are Related with Surrogates of Disturbed Lipid Metabolism among Older Men. *Frontiers in Medicine* (2016) 3:57-.

17. Savolainen O, Lind M, Bergström G, Fagerberg B, Sandberg A, Ross A. Biomarkers of Food Intake and Nutrient Status Are Associated with Glucose Tolerance Status and Development of Type 2 Diabetes in Older Swedish Women. *The American journal of clinical nutrition* (2017) 106(5):1302-10. doi: 10.3945/ajcn.117.152850.

18. Tulipani S, Palau-Rodriguez M, Alonso AM, Cardona F, Marco-Ramell A, Zonja B, et al. Biomarkers of Morbid Obesity and Prediabetes by Metabolomic Profiling of Human Discordant Phenotypes. *Clinica Chimica Acta* (2016).

19. Wang-Sattler R, Yu Z, Herder C, Messias AC, Illig T. Novel Biomarkers for Pre-Diabetes Identified by Metabolomics. *Molecular Systems Biology* (2012) 8(1):615.

20. Ju L. Serum Metabonornios of Impaired Giucose Reguiation with Uplciq-Tof Ms: JiLin University (2018).

21. Lokhov PG, Trifonova OP, Maslov DL, Balashova EE, Archakov AI, Shestakova EA, et al. Diagnosing Impaired Glucose Tolerance Using Direct Infusion Mass Spectrometry of Blood Plasma. *Plos One* (2014) 9(9):e105343.

22. YU Huan LQ-l, LI Li. Effects of Tianqijiangtang Capsule on Lipid Metabolomics in Impaired Glucose Tolerance (Igt) Volunteers. *CHINESE JOURNAL OF DIABETES* (2011). doi: 10.3969/j.issn.1006-6187.2011.05.008.

23. Kim Minjoo SG, Kang Miso. Replacing Carbohydrate with Protein and Fat in Prediabetes or Type-2 Diabetes: Greater Effect on Metabolites in Pbmc Than Plasma. *Nutrition & Metabolism* (2016) 13(1):3.

24. Kumar AA, Satheesh G, Vijayakumar G, Chandran M, Jaleel A. Postprandial Metabolism Is Impaired in Overweight Normoglycemic Young Adults without Family History of Diabetes. *entific Reports* (2020) 10(1).

25. Liu R, Zhao J, Guo J, Liu X, Yu J, Wang H, et al. Postprandial Metabolomics: Gc-Ms Analysis Reveals Differences in Organic Acid Profiles of Impaired Fasting Glucose Individuals in Response to Highland Barley Loads. *Food & Function* (2019).

26. Gar C, Rottenkolber M, Prehn C, Adamski J, Seissler J, Lechner A. Serum and Plasma Amino Acids as Markers of Prediabetes, Insulin Resistance, and Incident Diabetes. *Critical reviews in clinical laboratory sciences* (2018) 55(1):21-32. doi: 10.1080/10408363.2017.1414143.

27. Inken P, Erik P, Sandra G-M, Henning W, Matthias M, Tanja W, et al. A New Metabolomic Signature in Type-2 Diabetes Mellitus and Its Pathophysiology. *Plos One* (2014) 9(1):e85082.

28. Jun G, Aguilar D, Evans C, Burant C, Hanis C. Metabolomic Profiles Associated with Subtypes of Prediabetes among Mexican Americans in Starr County, Texas, USA. *Diabetologia* (2020) 63(2):287-95. doi: 10.1007/s00125-019-05031-4.

29. Lucio M, Fekete A, Weigert C, Wagele B, Zhao X, Chen J, et al. Insulin Sensitivity Is Reflected by Characteristic Metabolic Fingerprints--a Fourier Transform Mass Spectrometric Non-Targeted Metabolomics Approach. *PLoS One* (2010) 5(10):e13317. Epub 2010/10/27. doi: 10.1371/journal.pone.0013317.

30. Menni C, Fauman E, Erte I, Perry J, Kastenmüller G, Shin S, et al. Biomarkers for Type 2 Diabetes and Impaired Fasting Glucose Using a Nontargeted Metabolomics Approach. *Diabetes* (2013) 62(12):4270-6. doi: 10.2337/db13-0570.

31. Wei H, Pasman W, Rubingh C, Wopereis S, Tienstra M, Schroen J, et al. Urine Metabolomics Combined with the Personalized Diagnosis Guided by Chinese Medicine Reveals Subtypes of Pre-Diabetes. *Molecular Biosystems* (2012) 8(5):1482-91.

32. Zhao S, Zheng H, Lu X, Liu Y, Su B, Xu G. [Metabonomics and Phospholipid Metabolic Profiling of Abnormal Glucose Metabolism Based on High Performance Liquid Chromatography-Electrospray Mass Spectrometry]. *Se pu = Chinese journal of chromatography* (2011) 29(4):307-13. doi: 10.3724/sp.j.1123.2011.00307.

33. Wang TJ, Ngo D, Psychogios N, Dejam A, Gerszten RE. 2-Aminoadipic Acid Is a Biomarker for Diabetes Risk. *Journal of Clinical Investigation* (2013) 123(10):4309-17.

34. Rawat A, Misra G, Saxena M, Tripathi S, Dubey D, Saxena S, et al. (1)H Nmr Based Serum Metabolic Profiling Reveals Differentiating Biomarkers in Patients with Diabetes and Diabetes-Related Complication. *Diabetes Metab Syndr* (2019) 13(1):290-8. Epub 2019/01/16. doi: 10.1016/j.dsx.2018.09.009.

35. Park JE, Jeong GH, Lee IK, Yoon YR, Liu KH, Gu N, et al. A Pharmacometabolomic Approach to Predict Response to Metformin in Early-Phase Type 2 Diabetes Mellitus Patients. *Molecules* (2018) 23(7). Epub 2018/07/04. doi: 10.3390/molecules23071579.

36. Liyan L, Ying L, Cheng W, Rennan F, Changhao S. Free Fatty Acid Metabolic Profile and Biomarkers of Isolated Post-Challenge Diabetes Based on Gc-Ms and Multivariate Statistical Analysis. *Nutrition New sletter* (2012) (1):12-7.

37. Liu X, Gao X, Zhang R, Liu Z, Shen N, Di Y, et al. Discovery and Comparison of Serum Biomarkers for Diabetes Mellitus and Metabolic Syndrome Based on Uplc-Q-Tof/Ms. *Clin Biochem* (2020) 82:40-50. Epub 2020/03/21. doi: 10.1016/j.clinbiochem.2020.03.007.

38. Liao X, Liu B, Qu H, Zhang L, Lu Y, Xu Y, et al. A High Level of Circulating Valine Is a Biomarker for Type 2 Diabetes and Associated with the Hypoglycemic Effect of Sitagliptin. *Mediators Inflamm* (2019) 2019:8247019. Epub 2019/12/13. doi: 10.1155/2019/8247019.

39. Chailurkit LO, Paiyabhroma N, Sritara P, Vathesatogkit P, Yamwong S, Thonmung N, et al. Independent and Opposite Associations between Branched-Chain Amino Acids and Lysophosphatidylcholines with Incident Diabetes in Thais. *Metabolites* (2020) 10(2). Epub 2020/02/26. doi: 10.3390/metabo10020076.

40. de Mello VD, Paananen J, Lindstrom J, Lankinen MA, Shi L, Kuusisto J, et al. Indolepropionic Acid and Novel Lipid Metabolites Are Associated with a Lower Risk of Type 2 Diabetes in the Finnish Diabetes Prevention Study. *Sci Rep* (2017) 7:46337. Epub 2017/04/12. doi: 10.1038/srep46337.

41. Drogan D, Dunn WB, Lin W, Buijsse B, Schulze MB, Langenberg C, et al. Untargeted Metabolic Profiling Identifies Altered Serum Metabolites of Type 2 Diabetes Mellitus in a Prospective, Nested Case Control Study. *Clin Chem* (2015) 61(3):487-97. Epub 2014/12/20. doi: 10.1373/clinchem.2014.228965.

42. Lu Y, Wang Y, Ong CN, Subramaniam T, Choi HW, Yuan JM, et al. Metabolic Signatures and Risk of Type 2 Diabetes in a Chinese Population: An Untargeted Metabolomics Study Using Both Lc-Ms and Gc-Ms. *Diabetologia* (2016) 59(11):2349-59. Epub 2016/08/16. doi: 10.1007/s00125-016-4069-2.

43. Sun L, Liang L, Gao X, Zhang H, Yao P, Hu Y, et al. Early Prediction of Developing Type 2 Diabetes by Plasma Acylcarnitines: A Population-Based Study. *Diabetes Care* (2016) 39(9):1563-70. Epub 2016/07/09. doi: 10.2337/dc16-0232.

44. Al-Sulaiti H, Diboun I, Agha MV, Mohamed FFS, Atkin S, Domling AS, et al. Metabolic Signature of Obesity-Associated Insulin Resistance and Type 2 Diabetes. *J Transl Med* (2019) 17(1):348. Epub 2019/10/24. doi: 10.1186/s12967-019-2096-8.

45. Cao YF, Li J, Zhang Z, Liu J, Sun XY, Feng XF, et al. Plasma Levels of Amino Acids Related to Urea Cycle and Risk of Type 2 Diabetes Mellitus in Chinese Adults. *Front Endocrinol (Lausanne)* (2019) 10:50. Epub 2019/03/06. doi: 10.3389/fendo.2019.00050.

46. Ha CY, Kim JY, Paik JK, Kim OY, Paik YH, Lee EJ, et al. The Association of Specific Metabolites of Lipid Metabolism with Markers of Oxidative Stress, Inflammation and Arterial Stiffness in Men with Newly Diagnosed Type 2 Diabetes. *Clin Endocrinol (Oxf)* (2012) 76(5):674-82. Epub 2011/10/01. doi: 10.1111/j.1365-2265.2011.04244.x.

47. Li J, Cao YF, Sun XY, Han L, Li SN, Gu WQ, et al. Plasma Tyrosine and Its Interaction with Low High-Density Lipoprotein Cholesterol and the Risk of Type 2 Diabetes Mellitus in Chinese. *J Diabetes Investig* (2019) 10(2):491-8. Epub 2018/07/13. doi: 10.1111/jdi.12898.

48. Lin H, Cheng M, Lo C, Lin G, Lin S, Yeh J, et al. H Nuclear Magnetic Resonance (Nmr)-Based Cerebrospinal Fluid and Plasma Metabolomic Analysis in Type 2 Diabetic Patients and Risk Prediction for Diabetic Microangiopathy. *Journal of clinical medicine* (2019) 8(6). doi: 10.3390/jcm8060874.

49. Merino J, Leong A, Liu CT, Porneala B, Walford GA, von Grotthuss M, et al. Metabolomics Insights into Early Type 2 Diabetes Pathogenesis and Detection in Individuals with Normal Fasting Glucose. *Diabetologia* (2018) 61(6):1315-24. Epub 2018/04/08. doi: 10.1007/s00125-018-4599-x.

50. Mook-Kanamori DO, El-Din SMM, Takiddin AH, Hala AH, Al-Mahmoud KAS, Amina AO, et al. 1,5-Anhydroglucitol in Saliva Is a Noninvasive Marker of Short-Term Glycemic Control. *J Clin Endocrinol Metab* (3):479-83.

51. Liu J, Semiz S, van der Lee SJ, van der Spek A, Verhoeven A, van Klinken JB, et al. Metabolomics Based Markers Predict Type 2 Diabetes in a 14-Year Follow-up Study. *Metabolomics* (2017) 13(9):104. Epub 2017/08/15. doi: 10.1007/s11306-017-1239-2.

52. Tam ZY, Ng SP, Tan LQ, Lin CH, Rothenbacher D, Klenk J, et al. Metabolite Profiling in Identifying Metabolic Biomarkers in Older People with Late-Onset Type 2 Diabetes Mellitus. *Sci Rep* (2017) 7(1):4392. Epub 2017/07/01. doi: 10.1038/s41598-017-01735-y.

53. Yu D, Moore SC, Matthews CE, Xiang YB, Zhang X, Gao YT, et al. Plasma Metabolomic Profiles in Association with Type 2 Diabetes Risk and Prevalence in Chinese Adults. *Metabolomics* (2016) 12. Epub 2016/11/15. doi: 10.1007/s11306-015-0890-8.

54. Carter TC, Rein D, Padberg I, Peter E, Rennefahrt U, David DE, et al. Validation of a Metabolite Panel for Early Diagnosis of Type 2 Diabetes. *Metabolism Clinical & Experimental* (2016):1399-408.

55. Doorn MV, Vogels J, Tas A, Hoogdalem EJV, Burggraaf J, Cohen A, et al. Evaluation of Metabolite Profiles as Biomarkers for the Pharmacological Effects of Thiazolidinediones in Type 2 Diabetes Mellitus Patients and Healthy Volunteers. *British Journal of Clinical Pharmacology* (2010) 63(5):562-74.

56. Hai-zhen GYZPLJL. Serum Metabonomics Study on Type 2 Diabetes by Using Gas Chromatography/Mass Spectrometry. *Journal of Shanxi Datong University(Natural Science Edition)* (2017) (33):33-5.

57. Jun FHLXYWZLZDW. Screening of Urinary Biomarkers in Patients with Type 2 Diabetes Mellitus. *Journal of Hygiene Research* (2013) 42(006):907-14.

58. Kailong Y, Xianzhe S, Xin L, Peng G, Guowang X. Assessment of Therapeutic Effect of Losartan on Diabetes Mellitus with Gas Chromatography-Based Metabonomics. *Acta Academiae Medicinae Sinicae* (2007) 29(006):719-24.

59. Vangipurapu J, Silva LF, Kuulasmaa T, Smith U, Laakso M. Microbiota-Related Metabolites and the Risk of Type 2 Diabetes. *Diabetes Care* (2020) 43(6):dc192533.

60. Xiao-li M, Lei M, Xin-xia L, Lin-lin L, Ye W, Xin-min M. Urine Metabonomics Study on Diabetes Patients by Uplc /Q-Tof Ms. *Journal of Instrumental Analysis* (2014) 33(006):621-7.

61. Chou J, Liu R, Yu J, Liu X, Zhao X, Li Y, et al. Fasting Serum Alphahydroxybutyrate and Pyroglutamic Acid as Important Metabolites for Detecting Isolated Post-Challenge Diabetes Based on Organic Acid Profiles. *J Chromatogr B Analyt Technol Biomed Life Sci* (2018) 1100-1101:6-16. Epub 2018/09/30. doi: 10.1016/j.jchromb.2018.09.004.

62. Kaur P, Rizk N, Ibrahim S, Luo Y, Younes N, Perry B, et al. Quantitative Metabolomic and Lipidomic Profiling Reveals Aberrant Amino Acid Metabolism in Type 2 Diabetes. *Mol Biosyst* (2013) 9(2):307-17. Epub 2012/12/19. doi: 10.1039/c2mb25384d.

63. Lee Y, Pamungkas AD, Medriano CAD, Park J, Hong S, Jee SH, et al. High-Resolution Metabolomics Determines the Mode of Onset of Type 2 Diabetes in a 3-Year Prospective Cohort Study. *Int J Mol Med* (2018) 41(2):1069-77. Epub 2017/12/06. doi: 10.3892/ijmm.2017.3275.

64. Lo CJ, Tang HY, Huang CY, Lin CM, Ho HY, Shiao MS, et al. Metabolic Signature Differentiated Diabetes Mellitus from Lipid Disorder in Elderly Taiwanese. *J Clin Med* (2018) 8(1). Epub 2018/12/24. doi: 10.3390/jcm8010013.

65. Mack CI, Ferrario PG, Weinert CH, Egert B, Hoefle AS, Lee YM, et al. Exploring the Diversity of Sugar Compounds in Healthy, Prediabetic, and Diabetic Volunteers. *Mol Nutr Food Res* (2020) 64(9):e1901190. Epub 2020/03/15. doi: 10.1002/mnfr.201901190.

66. Rebholz CM, Yu B, Zheng Z, Chang P, Tin A, Kottgen A, et al. Serum Metabolomic Profile of Incident Diabetes. *Diabetologia* (2018) 61(5):1046-54. Epub 2018/03/21. doi: 10.1007/s00125-018-4573-7.

67. Savolainen O, Fagerberg B, Vendelbo Lind M, Sandberg AS, Ross AB, Bergstrom G. Biomarkers for Predicting Type 2 Diabetes Development-Can Metabolomics Improve on Existing Biomarkers? *PLoS One* (2017) 12(7):e0177738. Epub 2017/07/12. doi: 10.1371/journal.pone.0177738.

68. Suhre K, Meisinger C, Doring A, Altmaier E, Belcredi P, Gieger C, et al. Metabolic Footprint of Diabetes: A Multiplatform Metabolomics Study in an Epidemiological Setting. *PLoS One* (2010) 5(11):e13953. Epub 2010/11/19. doi: 10.1371/journal.pone.0013953.

69. Zhang N, Geng F, Hu ZH, Liu B, Li LJ. Preliminary Study of Urine Metabolism in Type Two Diabetic Patients Based on Gc-Ms. *American Journal of Translational Research* (2016) 8(7):2889.

70. Patel SG, Hsu JW, Jahoor F, Coraza I, Bain JR, Stevens RD, et al. Pathogenesis of a(-)Beta(+) Ketosis-Prone Diabetes. *Diabetes* (2013) 62(3):912-22. Epub 2012/11/20. doi: 10.2337/db12-0624.

71. GOU Xiao-jun ZS-x, LI Guang-ping, ZHANG Cheng, CHENG Wen, CHEN Fei, WANG Hua, ZHANG Lu, CHEN Yue. Urinary Metabolomics Study in Patients with Diabetic Peripheral Neuropathy. *Chinese Journal of Hospital Pharmacy* (2019) 039(024):2512-9.

72. Chen L, Cheng CY, Choi H, Ikram MK, Sabanayagam C, Tan GS, et al. Plasma Metabonomic Profiling of Diabetic Retinopathy. *Diabetes* (2016) 65(4):1099-108. Epub 2016/01/30. doi: 10.2337/db15-0661.

73. Jin H, Zhu B, Liu X, Jin J, Zou H. Metabolic Characterization of Diabetic Retinopathy: An (1)H-Nmr-Based Metabolomic Approach Using Human Aqueous Humor. *J Pharm Biomed Anal* (2019) 174:414-21. Epub 2019/06/19. doi: 10.1016/j.jpba.2019.06.013.

74. Jinkui ZXYFLJCXYGXRFJY. Plasma Metabolomic Profiling of Proliferative Diabetic Retinopathy. *Journal of Capital Medical University* (2020).

75. Kunikata H, Ida T, Sato K, Aizawa N, Sawa T, Tawarayama H, et al. Metabolomic Profiling of Reactive Persulfides and Polysulfides in the Aqueous and Vitreous Humors. *Sci Rep* (2017) 7:41984. Epub 2017/02/09. doi: 10.1038/srep41984.

76. Chen CJ, Liao WL, Chang CT, Lin YN, Tsai FJ. Identification of Urinary Metabolite Biomarkers of Type 2 Diabetes Nephropathy Using an Untargeted Metabolomic Approach. *J Proteome Res* (2018) 17(11):3997-4007. Epub 2018/09/29. doi: 10.1021/acs.jproteome.8b00644.

77. Devi S, Nongkhlaw B, Limesh M, Pasanna RM, Thomas T, Kuriyan R, et al. Acyl Ethanolamides in Diabetes and Diabetic Nephropathy: Novel Targets from Untargeted Plasma Metabolomic Profiles of South Asian Indian Men. *Sci Rep* (2019) 9(1):18117. Epub 2019/12/04. doi: 10.1038/s41598-019-54584-2.

78. Ibarra-Gonzalez I, Cruz-Bautista I, Bello-Chavolla OY, Vela-Amieva M, Pallares-Mendez R, Ruiz de Santiago YND, et al. Optimization of Kidney Dysfunction Prediction in Diabetic Kidney Disease Using Targeted Metabolomics. *Acta Diabetol* (2018) 55(11):1151-61. Epub 2018/09/03. doi: 10.1007/s00592-018-1213-0.

79. Jiang Hong SX, Jia Sha. The Mitochondria-Targeted Metabolic Tubular Injury in Diabetic Kidney Disease. *Cell Physiol Biochem* (2019). doi: 10.1159/000000011.

80. Li L, Wang C, Yang H, Liu S, Lu Y, Fu P, et al. Metabolomics Reveal Mitochondrial and Fatty Acid Metabolism Disorders That Contribute to the Development of Dkd in T2dm Patients. *Mol Biosyst* (2017) 13(11):2392-400. Epub 2017/09/29. doi: 10.1039/c7mb00167c.

81. Liu Y, Chen X, Liu Y, Chen T, Zhang Q, Zhang H, et al. Metabolomic Study of the Protective Effect of Gandi Capsule for Diabetic Nephropathy. *Chem Biol Interact* (2019) 314:108815. Epub 2019/09/10. doi: 10.1016/j.cbi.2019.108815.

82. Pena MJ, Lambers Heerspink HJ, Hellemons ME, Friedrich T, Dallmann G, Lajer M, et al. Urine and Plasma Metabolites Predict the Development of Diabetic Nephropathy in Individuals with Type 2 Diabetes Mellitus. *Diabet Med* (2014) 31(9):1138-47. Epub 2014/03/26. doi: 10.1111/dme.12447.

83. Sharma K, Karl B, Mathew AV, Gangoiti JA, Wassel CL, Saito R, et al. Metabolomics Reveals Signature of Mitochondrial Dysfunction in Diabetic Kidney Disease. *J Am Soc Nephrol* (2013) 24(11):1901-12. Epub 2013/08/21. doi: 10.1681/ASN.2013020126.

84. Solini A, Manca ML, Penno G, Pugliese G, Cobb JE, Ferrannini E. Prediction of Declining Renal Function and Albuminuria in Patients with Type 2 Diabetes by Metabolomics. *J Clin Endocrinol Metab* (2016) 101(2):696-704. Epub 2015/12/20. doi: 10.1210/jc.2015-3345.

85. Tavares G, Venturini G, Padilha K, Zatz R, Pereira AC, Thadhani RI, et al. 1,5-Anhydroglucitol Predicts Ckd Progression in Macroalbuminuric Diabetic Kidney Disease: Results from Non-Targeted Metabolomics. *Metabolomics* (2018) 14(4):39. Epub 2019/03/05. doi: 10.1007/s11306-018-1337-9.

86. Xia JF, Liang QL, Liang XP, Wang YM, Hu P, Li P, et al. Ultraviolet and Tandem Mass Spectrometry for Simultaneous Quantification of 21 Pivotal Metabolites in Plasma from Patients with Diabetic Nephropathy. *J Chromatogr B Analyt Technol Biomed Life Sci* (2009) 877(20-21):1930-6. Epub 2009/06/09. doi: 10.1016/j.jchromb.2009.05.047.

87. Xufang W, Mengjie L, Yongchun G, Weisong Q, Jiye A, Jinhua H, et al. Serum and Urinary Metabolomic Analysis in Patients with Diabetic Nephropathy. *Chinese Journal of Nephrology,Dialysis & Transplantation* (2012) (3):201-9.

88. Yang L. Metabonomics Study on the Biochemical Profiles of Diabetic Nephropathy: Xin (2011).

89. Yuhua M. Urine Metababonomics Study on the Biochemical Profiles of Diabetic Nephropathy: Xinjiang University (2014).

90. Zhang J, Yan L, Chen W, Lin L, Song X, Yan X, et al. Metabonomics Research of Diabetic Nephropathy and Type 2 Diabetes Mellitus Based on Uplc-Oatof-Ms System. *Anal Chim Acta* (2009) 650(1):16-22. Epub 2009/09/02. doi: 10.1016/j.aca.2009.02.027.

91. Zhu C, Liang QL, Hu P, Wang YM, Luo GA. Phospholipidomic Identification of Potential Plasma Biomarkers Associated with Type 2 Diabetes Mellitus and Diabetic Nephropathy. *Talanta* (2011) 85(4):1711-20. Epub 2011/08/30. doi: 10.1016/j.talanta.2011.05.036.

92. Zongmiao J. Preliminary Screening of Metabolic Markers Reiated to Diabetic Nephropathy (2019).
