# Supplementary material for: Clinical metabolomics in type 2 diabetes mellitus: from pathogenesis to biomarkers
Source: Front Endocrinol (Lausanne). 2025 Feb 25;16:1501305. doi: 10.3389/fendo.2025.1501305 (PMC11893406; doi:10.3389/fendo.2025.1501305)
Supplement: Supplementary file 2 [file SupplementaryFile2.doc]

**Clinical metabolomics in type 2 diabetes mellitus: from pathogenesis to biomarkers**

Jiao Kong^2,†^，Hetao Chen^1,3,†^，Yujin Ma^1^，Lei Zhang^4^，Lulu Chen^1,3^，Jiarui Huang^5^, Zizhe Zhao^1^, Hongwei Jiang^1,*^, Chuanxin Liu^1,*^

^1^ Luoyang Key Laboratory of Clinical Multiomics and Translational Medicine, Henan Key Laboratory of Rare Diseases, Endocrinology and Metabolism Center, The First Affiliated Hospital, and College of Clinical Medicine of Henan University of Science and Technology, Luoyang, China,

^2^ Institute of Drug Metabolism and Pharmaceutical Analysis, College of Pharmaceutical Sciences, Zhejiang University, Hangzhou, China,

^3^ Department of Clinical Laboratory, The First Affiliated Hospital, College of Clinical Medicine of Henan University of Science and Technology, Luoyang, China,

^4^ Department of Integrative Medicine, The First Affiliated Hospital, and College of Clinical Medicine of Henan University of Science and Technology, Luoyang, China,

^5^ Department of Critical Care Medicine, The First Affiliated Hospital, and College of Clinical Medicine of Henan University of Science and Technology, Luoyang, China

^†^ These authors contributed equally to this work and share first authorship

* Correspondence: Hongwei Jiang✉: jianghw@haust.edu.cn; Chuanxin Liu✉:15222003775@163.com.

**Contents:**

**Table S1 Baseline characterization of included literatures**

Table S1 Baseline characterization of included literatures.

| No | Include studies | Samples  (Ca/Co) | Gender  (M/F) | Year | Study type | Baseline stable | Journal | IF  (2019) | Bias risk | Stages |
| --- | --- | --- | --- | --- | --- | --- | --- | --- | --- | --- |
| 1 | Xiao Ji2014(1) | 43/45 | Ca:21/22  Co:22/23 | Ca:56.3±13.1  Co:55.1±12.2 | CCS | P＞0.05 | Chin J Clin Lab Sci | 0.73 | Low | PM |
| 2 | Yan Meihua2012(2) | 14/14 | Ca:0/14  Co:0/14 | Ca:39.64±13.3  Co:30.95±7.5 | CSS | P＞0.05 | - | - | Low | PM |
| 3 | Yu Huan 2011(3) | 29/32 | - | - | RCT | - | Chin J Diabetes | 1.391 | Unclear | PM |
| 4 | Li Bowen 2018(4) | 25/10 | Ca:8/17  Co:3/7 | Ca:54.41±10.3  Co:54.9±11.6 | RCT | P＞0.05 | Chin J Sports Med | 0.763 | Low | PM |
| 5 | Zhong Hongfu 2011(5) | 32/19 | Ca:8/17  Co:3/7 | Ca:53±7  Co:52±8 | RCT | P＞0.05 | - | - | Low | PM |
| 6 | Kumar A A 2020(6) | 20/30 | Ca:10/10  Co:15/15 | - | RCT | - | Scientific Reports | 4.011 | Unclear | PM |
| 7 | Liu Rui 2019(7) | 30/30 | - | - | CSS | P＞0.05 | Food&Function | 3.241 | Unclear | PM |
| 8 | Savolainen Otto 2017(8) | 357/383 | Ca:0/357  Co:0/383 | Ca:64  Co:64 | NCCS | P＞0.05 | Am. J. Clin. Nutr | 6.568 | Low | PM |
| 9 | Tulipani Sara 2016(9) | 12/19 | Ca:4/8  Co:4/15 | Ca:47±15  Co:53.67±14.1 | CSS | P＞0.05 | Clin. Chim. Acta | 2.735 | Low | PM |
| 10 | Cobb Jeff 2016(10) | 623/56 | Ca:249/374  Co:24/31 | Ca:46±8  Co:51±9 | CSS | P＞0.05 | Diabetes Care | 15.27 | Low | PM |
| 11 | Kim Minjoo 2016(11) | 40/40 | - | - | RCT | - | Nutr Metab | 3.599 | Unclear | PM |
| 12 | Lokhov Petr G 2014(12) | 20/30 | Ca:10/10  Co:15/15 | Ca:61.8±12.2  Co:53.3±14.0 | CSS | P<0.05 | Plos One | 2.776 | High | PM |
| 13 | Wang-Sattler Rui 2012(13) | 118/471 | Ca:10/10  Co:225/246 | Ca:63.9±5.5  Co:62.4±5.4 | CSS+CS | P＞0.05 | Mol. Syst. Biol | 9.8 | Low | PM |
| 14 | Padberg Inken 2014(14) | 177/28 | - | - | CS+CSS | P＞0.05 | Plos One | 2.776 | Unclear | PM |
| 15 | Wei Heng 2012(15) | 50/20 | - | - | CSS | - | Mol Biosyst | 2.855 | Unclear | PM |
| 16 | Zhao Sumin 2011(16)] | 32/31 | - | Ca:54.41±1.75  Co:53.42±1.77 | CSS | P＞0.05 | Chin J of Chrom | 1.853 | Unclear | PM |
| 17 | Lucio Marianna 2010(17) | 23/23 | - | - | CSS | - | Plos One | 2.776 | Unclear | PM |
| 18 | Goo Jun 2020(18) | 7/120 | Ca:0/7  Co:0/120 | 20-77 | CS | - | Diabetologia | 7.113 | Unclear | PM |
| 19 | Gar C 2018(19) | 38/113 | - | Ca:36.5±4.1  Co:3.53±3.8 | CSS | P＞0.05 | Crit Rev Clin Lab Sci | 4.817 | Unclear | PM |
| 20 | Cristina Menni 2013(20) | 536/184 | Ca:339/197  Co: 60/124 | Ca:64.2  Co:63.2 | CSS | - | Diabetes | 7.199 | Unclear | PM |
| 21 | Andersson-Hall Ulrika 2018(21) | 46/139 | Ca:0/46  Co: 0/139 | Ca:34±5  Co:35±6 | CS | P＞0.05 | J Diabetes Res | 3.04 | Low | PM |
| 22 | Luo Ju 2015(22) | 30/50 | Ca:10/20  Co: 18/32 | Ca:64.59±7.7  Co:60.21±13.2 | CSS | P＞0.05 | - | - | Low | PM |
| 23 | Kujala U M 2016(23) | 252/214 | Ca:252/0  Co: 214/0 | Ca:72.7±5.9  Co:71.6±6.1 | CSS | P＞0.05 | Frontiers in Medicine | 1.847 | Low | PM |
| 24 | Liu Liyang2012(24) | 53/50 | Ca:31/22  Co: 30/20 | Ca:53.5±10.5  Co: 55.7±10.4 | CSS | P＞0.05 | Nutrition Newsletter | - | Low | T2DM |
| 25 | Ma Xiaoli2014(25) | 38/32 | - | Ca:55±2  Co: 46±1 | CSS | - | Journal of Instrumental Analysis | 1.619 | Unclear | T2DM |
| 26 | Yuan Kailong2007(26) | 18/14 | Ca:9/9  Co: - | - | CSS | - | Acta Academiae Medicinae Sinicae | 1.135 | Unclear | T2DM |
| 27 | Gu Yan2017(27) | 36/40 | - | - | CSS | - | Journal of Shanxi Datong University(Natural Science Edition) | 0.327 | Unclear | T2DM |
| 28 | Fu Han2013(28) | 98/96 | Ca:47/51  Co: 47/49 | Ca:51.93±4.69  Co: 49.60±4.93 | CSS | - | Journal of Hygiene Research | 1.133 | Unclear | T2DM |
| 29 | Vangipurapu Jagadish2020(29) | 5169/522 | - | Ca:-  Co: 57.7±7 | CS | - | Diabetes Care | 15.27 | Unclear | T2DM |
| 30 | Liao X2019(30) | 20/19 | Ca:11/9  Co: 7/12 | Ca:51.90±3.18  Co: 48.58±3.19 | CSS | P>0.05 | Mediators of Inflammation | 3.55 | Low | T2DM |
| 31 | Atul R 2018(31) | 38/50 | Ca:21/17  Co: 29/21 | Ca:46.64±3.40  Co: 47.83±4.97 | CSS | P>0.05 | Diabetes Metab Syndr | - | Low | T2DM |
| 32 | Carter T C2016(32) | Discovery61/78  Validation  56/445 | - | - | CCS | - | Metabolism | 6.513 | Unclear | T2DM |
| 33 | Mook-Kanamori D O2013(33) | 188/181 | Ca:81/107  Co: 99/82 | Ca:53.8  Co: 38.5 | CCS | P<0.05 | Journal of Clinical Endocrinology & Metabolism | 5.605 | High | T2DM |
| 34 | Lin H T2019(34) | 40/36 | Ca:34/6  Co: 21/15 | Ca:55.15±10.46  Co: 47.56±14.18 | CSS | P<0.05 | J Clin Med | 5.688 | High | T2DM |
| 35 | Wang T J2013(35) | 188/188 | Ca:107/81  Co:107/81 | Ca:56±9  Co:57±8 | NCCS | P>0.05 | J. Clin. Invest. | 12.282 | Low | T2DM |
| 36 | Doorn M V2007(36) | 18/16 | Ca:9/9  Co:8/8 | Ca:56.8  Co:22.1 | CS | - | Br J Clin Pharmacol | 3.867 | Unclear | T2DM |
| 37 | Liu Xuan2020(37) | 34/50 | Ca:17/17  Co:25/25 | Ca:55.37±8.92  Co:54.76±6.74 | CSS | P>0.05 | Clin. Biochem | 2.43 | Low | T2DM |
| 38 | Mack Carina I2020(38) | 11/15 | Ca:15/0  Co:3/8 | Ca:65.3±4.7  Co:26.3±2.5 | CSS | - | Mol Nutr Food Res | 4.653 | Unclear | T2DM |
| 39 | Al-Sulaiti Haya2019(39) | 14/32 | Ca:9/5  Co:0/32 | Ca:43  Co:28 | CSS | P<0.05 | J Transl Med | 4.098 | High | T2DM |
| 40 | Li Jing2019(40) | 1032/1522 | Ca1131/391  Co:549/973 | Ca:57.2±13.8  Co:46.3±13.7 | CSS | P<0.05 | J Diabetes Investig | 3.902 | High | T2DM |
| 41 | Chailurkit La-Or2020(41) | 38/41 | Ca:34/4  Co:35/6 | Ca:43.89  Co:43.10 | CSS | P>0.05 | Metabolites | 3.303 | Low | T2DM |
| 42 | Chou Jing2018(42) | 47/48 | Ca:24/23  Co:20/28 | Ca:53.1±6.4  Co:52.5±6.7 | CSS | - | PLoS ONE | 2.776 | Unclear | T2DM |
| 43 | Park J E2018(43) | 9/13 | Ca:2/7  Co:8/5 | Ca:54.3±8.90  Co:52.2±7.75 | CSS | P>0.05 | Molecules | 3.060 | Low | T2DM |
| 44 | Merino Jordi2018(44) | 95/1055 | Ca:43/52  Co:636/419 | Ca:54±9  Co:53±10 | CSS | P<0.05 | Diabetologia | 7.113 | High | T2DM |
| 45 | Lee Y2018(45) | 24/24 | Ca:20/4  Co:19/5 | Ca:45.13±7.01  Co:39.29±7.33 | CS | - | International Journal of Molecular Medicine | 2.928 | Unclear | T2DM |
| 46 | Wang Huijuan2018(46) | 30/30 | Ca:15/15  Co:15/15 | Ca:52  Co:53.5 | CSS | P>0.05 | Clin. Chim. Acta | 2.735 | Low | T2DM |
| 47 | Savolainen Otto2017(47) | 202/188 | - | - | CSS+CS | - | PLoS ONE | 2.776 | Unclear | T2DM |
| 48 | de Mello Vanessa D2017(48) | 96/104 | Ca:35/61  Co:37/67 | Ca:55.3±7.2  Co:56.3±6.6 | CS | P>0.05 | Sci Rep | 4.011 | Low | T2DM |
| 49 | Lu, Yonghai2016(49) | 197/197 | Ca:80/117  Co:80/117 | Ca:55.1±2.7  Co:55.2±2.9 | CCS | P>0.05 | Diabetologia | 7.113 | Low | T2DM |
| 50 | Sun L2016(50) | 507/1596 | Ca:225/282  Co:673/923 | Ca:58.3  Co:58.4 | CS | P>0.05 | Diabetes Care | 15.27 | Low | T2DM |
| 51 | Mihalik S J 2012(51) | 17/39 | Ca:8/9  Co:20/19 | Ca:15.3±1.8  Co:13.0±0.23 | CCS | P<0.05 | Diabetes Care | 15.27 | High | T2DM |
| 52 | Ha Chang Young2012(52) | 26/27 | - | Ca:49.2±1.4  Co:50.0±1.27 | CCS | - | Clin. Endocrinol. (Oxf) | 2.897 | Unclear | T2DM |
| 53 | Cao Yun-Feng2012(53) | 401/1522 | Ca:223/178  Co:1131/391 | Ca:52.5±14.5  Co:46.3±13.7 | CCS | P<0.05 | Front Endocrinol (Lausanne) | 3.634 | High | T2DM |
| 54 | Lo Chi-Jen2018(54) | 49/57 | - | Ca:79.8±7.7  Co:80.1±7.3 | CCS | - | J Clin Med | 5.688 | Unclear | T2DM |
| 55 | Rebholz C M2018(55) | 1126/1813 | Ca:674/452  Co:1081/732 | Ca:52.3  Co:53.6 | CS | - | Diabetologia | 7.113 | Unclear | T2DM |
| 56 | Liu J 2017(56) | 212/2564 | Ca:108/104  Co:1132/1432 | Ca:59.8±11.8  Co:48.2±11.3 | CS | P<0.05 | Metabolomics | 3.167 | High | T2DM |
| 57 | Tam Zhi Yang2017(57) | 80/78 | Ca:36/44  Co:51/27 | Ca:81.25  Co:73.80 | CCS | P<0.05 | Sci Rep | 4.011 | High | T2DM |
| 58 | Yu Danxia2016(58) | 68/835 | Ca:12/56  Co:177/658 | Ca:60.7±6.9  Co:57.4±7.9 | CS | P<0.05 | Metabolomics | 3.167 | High | T2DM |
| 59 | Zhang Ning2016(59) | 68/58 | - | - | CCS | - | Am J Transl Res | 3.266 | Unclear | T2DM |
| 60 | Dagmar Drogan2015(60) | Substudy 1  150/150  Substudy 2  150/150 | Substudy 1  Ca:81/69  Co:81/69  Substudy 2  Ca:81/69  Co:81/69 | Substudy 1  Ca:54.2  Co:54.2  Substudy 2  Ca:55.1  Co:55.1 | CCS | P>0.05 | Clinical Chemistry | 6.891 | Low | T2DM |
| 61 | Kaur Prabhjit2013(61) | 105/77 | Ca:82/23  Co:52/25 | Ca:53.6±8.99  Co:32.2±8.75 | CSS | - | Mol Biosyst | 2.855 | Unclear | T2DM |
| 62 | Karsten S2010(62) | 40/60 | - | Ca:67.7  Co:65.6 | CSS | - | PLoS ONE | 2.776 | Unclear | T2DM |
| 63 | Patel SG2013(63) | 20/19 | - | Ca:41.9±2.1  Co:50.7±2.7 | CSS | - | Diabetes | 7.199 | Unclear | T2DK |
| 64 | Gou Xiaojun2014(64) | 40/40 | Ca:18/22  Co:21/19 | Ca:61.16±13.24  Co:57.18±8.57 | CSS | P>0.05 | Chinese Journal of Hospital Pharmacy | 1.392 | Low | T2DPN |
| 65 | Zhu Xiaorong2020(65) | 21/21 | Ca:9/12  Co:9/12 | Ca:51.6±7.7  Co:53.8±6.8 | CSS | P>0.05 | Journal of Capital Medical University | 1.326 | Low | T2DR |
| 66 | Jin H2019(66) | 13/7 | - | Ca:61.77±8.34  Co:66.71±12.16 | CSS | - | Journal of Pharmaceutical and Biomedical Analysis | 2.983 | Unclear | T2DR |
| 67 | Chen Liyan2016(67) | 40/40 | Ca:20/20  Co:23/17 | Ca:59  Co:62 | CSS | P>0.05 | Diabetes | 7.199 | Low | T2DR |
| 68 | Kunikata Hiroshi2017(68) | 18/22 | Ca:9/9  Co:9/13 | Ca:57.7±16.1  Co:71.0±13.4 | CSS | P<0.05 | Sci Rep | 4.011 | High | T2DR |
| 69 | Jiang Zongmiao2019(69) | 45/46/36 | Ca2:26/10  Ca1:29/17  Co:30/15 | Ca2:55.58±11.44  Ca1:58.22±11.77  Co:55.16±9.13 | CSS | P>0.05 | - | - | Low | T2DN |
| 70 | Ma Yuhua2014(70) | 23/29 | Ca:11/12  Co:14/15 | Ca:27±1  Co:59±13 | CSS | - | - | - | Unclear | T2DN |
| 71 | Wang Xufang2012(71) | 30/30/30 | - | Ca2:49.5±9.8  Ca1:45.6±9.0  Co:49.7±10.3 | CSS | - | Chinese Journal of Nephrology,Dialysis & Transplantation | 1.047 | Unclear | T2DN |
| 72 | Liu Yang2011(72) | 8/7/4 | Ca2:5/3  Ca1:3/4  Co:2/2 | Ca2:68.4±7.3  Ca1:65.6±9.4  Co:24.8±1.5 | CSS | - | - | - | Unclear | T2DN |
| 73 | Sharma K2013(73) | 61/41 | Ca:25/36  Co:18/23 | Ca:60.6±11.9  Co:59.1±6.8 | CSS | - | J. Am. Soc. Nephrol | 8.547 | Unclear | T2DN |
| 74 | Ibarra-González Isabel2018(74) | 42/102/55 | Ca2:20/35  Ca1:69/33  Co:28/14 | Ca2:61.71±8.22  Ca1:60.40±8.22  Co:54.65±9.08 | CSS | P<0.05 | Acta Diabetol | 2.996 | High | T2DN |
| 75 | Liu Yue2019(75) | 13/13 | Ca:2/11  Co:3/10 | Ca:61.6±9.7  Co:63.1±7.9 | CSS | - | Chem. Biol. Interact | 3.407 | Unclear | T2DN |
| 76 | Tavares Gesiane2018(76) | 17/37 | Ca:8/29  Co:2/35 | Ca:57.5±10.3  Co:57.7±10.3 | CSS | P>0.05 | Metabolomics | 3.167 | Low | T2DN |
| 77 | Jiang Hong2019(77) | 60/48/65 | Ca2:42/18  Ca1:29/19  Co:35/30 | Ca2:53.35±1.51  Ca1:53.98±2.32  Co:49.72±1.52 | CSS | P>0.05 | Cell. Physiol. Biochem | 5.5 | Low | T2DN |
| 78 | Chen C J2018(78) | 52/86/76 | Ca2:40/36  Ca1:46/40  Co:26/26 | Ca2:65  Ca1:60.5  Co:61 | CSS | P>0.05 | J Proteome Res | 3.78 | Low | T2DN |
| 79 | Li L2017(79) | 20/25/24 | - | Ca2:59.50  Ca1:61.00  Co:58.00 | CSS | - | Mol Biosyst | 2.855 | Unclear | T2DN |
| 80 | Zhang J2009(80) | 8/33/25 | Ca2:4/4  Ca1:17/16  Co:13/12 | Ca2:50±16  Ca1:48±17  Co:52±19 | CSS | - | Anal. Chim. Acta | 5.256 | Unclear | T2DN |
| 81 | Devi Sarita2019(81) | 31/29/30 | - | Ca2:52.8±7.2  Ca1:48.8±8.1  Co:52±19 | CSS | P<0.05 | Sci Rep | 4.011 | High | T2DN |
| 82 | Xia J F2009(82) | 88/50 | - | Ca:45-70  Co:45-70 | CCS | - | J. Chromatogr. B | 2.813 | Unclear | T2DN |
| 83 | Pena M J2014(83) | 21/21/21 | Ca2:18/3  Ca1:17/4  Co:17/4 | Ca2:65.6±6.9  Ca1:62.3±9.5  Co:64.8±10 | CCS | P>0.05 | Diabet. Med | 3.107 | Low | T2DN |
| 84 | Solini Anna2016(84) | 241/45 | Ca:152/89  Co:25/20 | Ca:61±7  Co:68±5 | CCS | P<0.05 | J. Clin. Endocrinol. Metab | 5.605 | High | T2DN |
| 85 | Zhu Chao2011(85) | 52/30/30 | Ca2:27/25  Ca1:16/14  Co:19/11 | Ca2:56.9±8.5  Ca1:59.5±7.2  Co:48.8±5.6 | CCS | - | Talanta | 4.916 | Unclear | T2DN |

Note：Ca:Case; Co: Control; CCS: Case control study; NCCS: Nested case control study; CSS: Cross sectional study; CS: Cohort study; RCT: Randomized Controlled Trial.

**Reference**

1. Xiao Ji ZL, Ma Mingkun, Yan Weili, Liu Na, Liu Shuye. Serum Metabonomics in Patients with Abnormal Glucose Metabolism. *Chinese Journal of Clinical Laboratory Science* (2014) 32(012):909-11.

2. Meihua Y. Research on Mechanism of Female Impaired Glucose Regulation (2012).

3. YU Huan LQ-l, LI Li. Effects of Tianqijiangtang Capsule on Lipid Metabolomics in Impaired Glucose Tolerance (Igt) Volunteers. *CHINESE JOURNAL OF DIABETES* (2011). doi: 10.3969/j.issn.1006-6187.2011.05.008.

4. Zhengzhen LBJMZNLCWYZXZYW. Effects of Aerobic Exercise on Plasma Metabolites in Prediabetes Subjects. *Chinese Journal of Sports Medicine* (2018) 37(4):301-8.

5. Hongfu Z. The Applications of Liquid Chromatography Combined with Mass Spectrometry in Impaired Glucose Tolerance and Liver Cirrhosis: East China University of Science and Technology (2011).

6. Kumar AA, Satheesh G, Vijayakumar G, Chandran M, Jaleel A. Postprandial Metabolism Is Impaired in Overweight Normoglycemic Young Adults without Family History of Diabetes. *entific Reports* (2020) 10(1).

7. Liu R, Zhao J, Guo J, Liu X, Yu J, Wang H, et al. Postprandial Metabolomics: Gc-Ms Analysis Reveals Differences in Organic Acid Profiles of Impaired Fasting Glucose Individuals in Response to Highland Barley Loads. *Food & Function* (2019).

8. Savolainen O, Lind M, Bergström G, Fagerberg B, Sandberg A, Ross A. Biomarkers of Food Intake and Nutrient Status Are Associated with Glucose Tolerance Status and Development of Type 2 Diabetes in Older Swedish Women. *The American journal of clinical nutrition* (2017) 106(5):1302-10. doi: 10.3945/ajcn.117.152850.

9. Tulipani S, Palau-Rodriguez M, Alonso AM, Cardona F, Marco-Ramell A, Zonja B, et al. Biomarkers of Morbid Obesity and Prediabetes by Metabolomic Profiling of Human Discordant Phenotypes. *Clinica Chimica Acta* (2016).

10. Cobb J, Eckhart A, Motsinger-Reif A, Carr B, Groop L, Ferrannini E. Α-Hydroxybutyric Acid Is a Selective Metabolite Biomarker of Impaired Glucose Tolerance. *Diabetes Care* (2016).

11. Kim Minjoo SG, Kang Miso. Replacing Carbohydrate with Protein and Fat in Prediabetes or Type-2 Diabetes: Greater Effect on Metabolites in Pbmc Than Plasma. *Nutrition & Metabolism* (2016) 13(1):3.

12. Lokhov PG, Trifonova OP, Maslov DL, Balashova EE, Archakov AI, Shestakova EA, et al. Diagnosing Impaired Glucose Tolerance Using Direct Infusion Mass Spectrometry of Blood Plasma. *Plos One* (2014) 9(9):e105343.

13. Wang-Sattler R, Yu Z, Herder C, Messias AC, Illig T. Novel Biomarkers for Pre-Diabetes Identified by Metabolomics. *Molecular Systems Biology* (2012) 8(1):615.

14. Inken P, Erik P, Sandra G-M, Henning W, Matthias M, Tanja W, et al. A New Metabolomic Signature in Type-2 Diabetes Mellitus and Its Pathophysiology. *Plos One* (2014) 9(1):e85082.

15. Wei H, Pasman W, Rubingh C, Wopereis S, Tienstra M, Schroen J, et al. Urine Metabolomics Combined with the Personalized Diagnosis Guided by Chinese Medicine Reveals Subtypes of Pre-Diabetes. *Molecular Biosystems* (2012) 8(5):1482-91.

16. Zhao S, Zheng H, Lu X, Liu Y, Su B, Xu G. [Metabonomics and Phospholipid Metabolic Profiling of Abnormal Glucose Metabolism Based on High Performance Liquid Chromatography-Electrospray Mass Spectrometry]. *Se pu = Chinese journal of chromatography* (2011) 29(4):307-13. doi: 10.3724/sp.j.1123.2011.00307.

17. Lucio M, Fekete A, Weigert C, Wagele B, Zhao X, Chen J, et al. Insulin Sensitivity Is Reflected by Characteristic Metabolic Fingerprints--a Fourier Transform Mass Spectrometric Non-Targeted Metabolomics Approach. *PLoS One* (2010) 5(10):e13317. Epub 2010/10/27. doi: 10.1371/journal.pone.0013317.

18. Jun G, Aguilar D, Evans C, Burant C, Hanis C. Metabolomic Profiles Associated with Subtypes of Prediabetes among Mexican Americans in Starr County, Texas, USA. *Diabetologia* (2020) 63(2):287-95. doi: 10.1007/s00125-019-05031-4.

19. Gar C, Rottenkolber M, Prehn C, Adamski J, Seissler J, Lechner A. Serum and Plasma Amino Acids as Markers of Prediabetes, Insulin Resistance, and Incident Diabetes. *Critical reviews in clinical laboratory sciences* (2018) 55(1):21-32. doi: 10.1080/10408363.2017.1414143.

20. Menni C, Fauman E, Erte I, Perry J, Kastenmüller G, Shin S, et al. Biomarkers for Type 2 Diabetes and Impaired Fasting Glucose Using a Nontargeted Metabolomics Approach. *Diabetes* (2013) 62(12):4270-6. doi: 10.2337/db13-0570.

21. Andersson-Hall U, Gustavsson C, Pedersen A, Malmodin D, Joelsson L, Holmäng A. Higher Concentrations of Bcaas and 3-Hib Are Associated with Insulin Resistance in the Transition from Gestational Diabetes to Type 2 Diabetes. *Journal of diabetes research* (2018) 2018:4207067. doi: 10.1155/2018/4207067.

22. Ju L. Serum Metabonornios of Impaired Giucose Reguiation with Uplciq-Tof Ms: JiLin University (2018).

23. Kujala UM, Markku P, Laine MK, Jaakko K, Heinonen OJ, Jouko S, et al. Branched-Chain Amino Acid Levels Are Related with Surrogates of Disturbed Lipid Metabolism among Older Men. *Frontiers in Medicine* (2016) 3:57-.

24. Liyan L, Ying L, Cheng W, Rennan F, Changhao S. Free Fatty Acid Metabolic Profile and Biomarkers of Isolated Post-Challenge Diabetes Based on Gc-Ms and Multivariate Statistical Analysis. *Nutrition New sletter* (2012) (1):12-7.

25. Xiao-li M, Lei M, Xin-xia L, Lin-lin L, Ye W, Xin-min M. Urine Metabonomics Study on Diabetes Patients by Uplc /Q-Tof Ms. *Journal of Instrumental Analysis* (2014) 33(006):621-7.

26. Kailong Y, Xianzhe S, Xin L, Peng G, Guowang X. Assessment of Therapeutic Effect of Losartan on Diabetes Mellitus with Gas Chromatography-Based Metabonomics. *Acta Academiae Medicinae Sinicae* (2007) 29(006):719-24.

27. Hai-zhen GYZPLJL. Serum Metabonomics Study on Type 2 Diabetes by Using Gas Chromatography/Mass Spectrometry. *Journal of Shanxi Datong University(Natural Science Edition)* (2017) (33):33-5.

28. Jun FHLXYWZLZDW. Screening of Urinary Biomarkers in Patients with Type 2 Diabetes Mellitus. *Journal of Hygiene Research* (2013) 42(006):907-14.

29. Vangipurapu J, Silva LF, Kuulasmaa T, Smith U, Laakso M. Microbiota-Related Metabolites and the Risk of Type 2 Diabetes. *Diabetes Care* (2020) 43(6):dc192533.

30. Liao X, Liu B, Qu H, Zhang L, Lu Y, Xu Y, et al. A High Level of Circulating Valine Is a Biomarker for Type 2 Diabetes and Associated with the Hypoglycemic Effect of Sitagliptin. *Mediators Inflamm* (2019) 2019:8247019. Epub 2019/12/13. doi: 10.1155/2019/8247019.

31. Rawat A, Misra G, Saxena M, Tripathi S, Dubey D, Saxena S, et al. (1)H Nmr Based Serum Metabolic Profiling Reveals Differentiating Biomarkers in Patients with Diabetes and Diabetes-Related Complication. *Diabetes Metab Syndr* (2019) 13(1):290-8. Epub 2019/01/16. doi: 10.1016/j.dsx.2018.09.009.

32. Carter TC, Rein D, Padberg I, Peter E, Rennefahrt U, David DE, et al. Validation of a Metabolite Panel for Early Diagnosis of Type 2 Diabetes. *Metabolism Clinical & Experimental* (2016):1399-408.

33. Mook-Kanamori DO, El-Din SMM, Takiddin AH, Hala AH, Al-Mahmoud KAS, Amina AO, et al. 1,5-Anhydroglucitol in Saliva Is a Noninvasive Marker of Short-Term Glycemic Control. *J Clin Endocrinol Metab* (3):479-83.

34. Lin H, Cheng M, Lo C, Lin G, Lin S, Yeh J, et al. H Nuclear Magnetic Resonance (Nmr)-Based Cerebrospinal Fluid and Plasma Metabolomic Analysis in Type 2 Diabetic Patients and Risk Prediction for Diabetic Microangiopathy. *Journal of clinical medicine* (2019) 8(6). doi: 10.3390/jcm8060874.

35. Wang TJ, Ngo D, Psychogios N, Dejam A, Gerszten RE. 2-Aminoadipic Acid Is a Biomarker for Diabetes Risk. *Journal of Clinical Investigation* (2013) 123(10):4309-17.

36. Doorn MV, Vogels J, Tas A, Hoogdalem EJV, Burggraaf J, Cohen A, et al. Evaluation of Metabolite Profiles as Biomarkers for the Pharmacological Effects of Thiazolidinediones in Type 2 Diabetes Mellitus Patients and Healthy Volunteers. *British Journal of Clinical Pharmacology* (2010) 63(5):562-74.

37. Liu X, Gao X, Zhang R, Liu Z, Shen N, Di Y, et al. Discovery and Comparison of Serum Biomarkers for Diabetes Mellitus and Metabolic Syndrome Based on Uplc-Q-Tof/Ms. *Clin Biochem* (2020) 82:40-50. Epub 2020/03/21. doi: 10.1016/j.clinbiochem.2020.03.007.

38. Mack CI, Ferrario PG, Weinert CH, Egert B, Hoefle AS, Lee YM, et al. Exploring the Diversity of Sugar Compounds in Healthy, Prediabetic, and Diabetic Volunteers. *Mol Nutr Food Res* (2020) 64(9):e1901190. Epub 2020/03/15. doi: 10.1002/mnfr.201901190.

39. Al-Sulaiti H, Diboun I, Agha MV, Mohamed FFS, Atkin S, Domling AS, et al. Metabolic Signature of Obesity-Associated Insulin Resistance and Type 2 Diabetes. *J Transl Med* (2019) 17(1):348. Epub 2019/10/24. doi: 10.1186/s12967-019-2096-8.

40. Li J, Cao YF, Sun XY, Han L, Li SN, Gu WQ, et al. Plasma Tyrosine and Its Interaction with Low High-Density Lipoprotein Cholesterol and the Risk of Type 2 Diabetes Mellitus in Chinese. *J Diabetes Investig* (2019) 10(2):491-8. Epub 2018/07/13. doi: 10.1111/jdi.12898.

41. Chailurkit LO, Paiyabhroma N, Sritara P, Vathesatogkit P, Yamwong S, Thonmung N, et al. Independent and Opposite Associations between Branched-Chain Amino Acids and Lysophosphatidylcholines with Incident Diabetes in Thais. *Metabolites* (2020) 10(2). Epub 2020/02/26. doi: 10.3390/metabo10020076.

42. Chou J, Liu R, Yu J, Liu X, Zhao X, Li Y, et al. Fasting Serum Alphahydroxybutyrate and Pyroglutamic Acid as Important Metabolites for Detecting Isolated Post-Challenge Diabetes Based on Organic Acid Profiles. *J Chromatogr B Analyt Technol Biomed Life Sci* (2018) 1100-1101:6-16. Epub 2018/09/30. doi: 10.1016/j.jchromb.2018.09.004.

43. Park JE, Jeong GH, Lee IK, Yoon YR, Liu KH, Gu N, et al. A Pharmacometabolomic Approach to Predict Response to Metformin in Early-Phase Type 2 Diabetes Mellitus Patients. *Molecules* (2018) 23(7). Epub 2018/07/04. doi: 10.3390/molecules23071579.

44. Merino J, Leong A, Liu CT, Porneala B, Walford GA, von Grotthuss M, et al. Metabolomics Insights into Early Type 2 Diabetes Pathogenesis and Detection in Individuals with Normal Fasting Glucose. *Diabetologia* (2018) 61(6):1315-24. Epub 2018/04/08. doi: 10.1007/s00125-018-4599-x.

45. Lee Y, Pamungkas AD, Medriano CAD, Park J, Hong S, Jee SH, et al. High-Resolution Metabolomics Determines the Mode of Onset of Type 2 Diabetes in a 3-Year Prospective Cohort Study. *Int J Mol Med* (2018) 41(2):1069-77. Epub 2017/12/06. doi: 10.3892/ijmm.2017.3275.

46. Wang H, Zhang H, Yao L, Cui L, Zhang L, Gao B, et al. Serum Metabolic Profiling of Type 2 Diabetes Mellitus in Chinese Adults Using an Untargeted Gc/Tofms. *Clin Chim Acta* (2018) 477:39-47. Epub 2017/12/05. doi: 10.1016/j.cca.2017.11.036.

47. Savolainen O, Fagerberg B, Vendelbo Lind M, Sandberg AS, Ross AB, Bergstrom G. Biomarkers for Predicting Type 2 Diabetes Development-Can Metabolomics Improve on Existing Biomarkers? *PLoS One* (2017) 12(7):e0177738. Epub 2017/07/12. doi: 10.1371/journal.pone.0177738.

48. de Mello VD, Paananen J, Lindstrom J, Lankinen MA, Shi L, Kuusisto J, et al. Indolepropionic Acid and Novel Lipid Metabolites Are Associated with a Lower Risk of Type 2 Diabetes in the Finnish Diabetes Prevention Study. *Sci Rep* (2017) 7:46337. Epub 2017/04/12. doi: 10.1038/srep46337.

49. Lu Y, Wang Y, Ong CN, Subramaniam T, Choi HW, Yuan JM, et al. Metabolic Signatures and Risk of Type 2 Diabetes in a Chinese Population: An Untargeted Metabolomics Study Using Both Lc-Ms and Gc-Ms. *Diabetologia* (2016) 59(11):2349-59. Epub 2016/08/16. doi: 10.1007/s00125-016-4069-2.

50. Sun L, Liang L, Gao X, Zhang H, Yao P, Hu Y, et al. Early Prediction of Developing Type 2 Diabetes by Plasma Acylcarnitines: A Population-Based Study. *Diabetes Care* (2016) 39(9):1563-70. Epub 2016/07/09. doi: 10.2337/dc16-0232.

51. Mihalik SJ, Michaliszyn SF, de las Heras J, Bacha F, Lee S, Chace DH, et al. Metabolomic Profiling of Fatty Acid and Amino Acid Metabolism in Youth with Obesity and Type 2 Diabetes: Evidence for Enhanced Mitochondrial Oxidation. *Diabetes Care* (2012) 35(3):605-11. Epub 2012/01/24. doi: 10.2337/DC11-1577.

52. Ha CY, Kim JY, Paik JK, Kim OY, Paik YH, Lee EJ, et al. The Association of Specific Metabolites of Lipid Metabolism with Markers of Oxidative Stress, Inflammation and Arterial Stiffness in Men with Newly Diagnosed Type 2 Diabetes. *Clin Endocrinol (Oxf)* (2012) 76(5):674-82. Epub 2011/10/01. doi: 10.1111/j.1365-2265.2011.04244.x.

53. Cao YF, Li J, Zhang Z, Liu J, Sun XY, Feng XF, et al. Plasma Levels of Amino Acids Related to Urea Cycle and Risk of Type 2 Diabetes Mellitus in Chinese Adults. *Front Endocrinol (Lausanne)* (2019) 10:50. Epub 2019/03/06. doi: 10.3389/fendo.2019.00050.

54. Lo CJ, Tang HY, Huang CY, Lin CM, Ho HY, Shiao MS, et al. Metabolic Signature Differentiated Diabetes Mellitus from Lipid Disorder in Elderly Taiwanese. *J Clin Med* (2018) 8(1). Epub 2018/12/24. doi: 10.3390/jcm8010013.

55. Rebholz CM, Yu B, Zheng Z, Chang P, Tin A, Kottgen A, et al. Serum Metabolomic Profile of Incident Diabetes. *Diabetologia* (2018) 61(5):1046-54. Epub 2018/03/21. doi: 10.1007/s00125-018-4573-7.

56. Liu J, Semiz S, van der Lee SJ, van der Spek A, Verhoeven A, van Klinken JB, et al. Metabolomics Based Markers Predict Type 2 Diabetes in a 14-Year Follow-up Study. *Metabolomics* (2017) 13(9):104. Epub 2017/08/15. doi: 10.1007/s11306-017-1239-2.

57. Tam ZY, Ng SP, Tan LQ, Lin CH, Rothenbacher D, Klenk J, et al. Metabolite Profiling in Identifying Metabolic Biomarkers in Older People with Late-Onset Type 2 Diabetes Mellitus. *Sci Rep* (2017) 7(1):4392. Epub 2017/07/01. doi: 10.1038/s41598-017-01735-y.

58. Yu D, Moore SC, Matthews CE, Xiang YB, Zhang X, Gao YT, et al. Plasma Metabolomic Profiles in Association with Type 2 Diabetes Risk and Prevalence in Chinese Adults. *Metabolomics* (2016) 12. Epub 2016/11/15. doi: 10.1007/s11306-015-0890-8.

59. Zhang N, Geng F, Hu ZH, Liu B, Li LJ. Preliminary Study of Urine Metabolism in Type Two Diabetic Patients Based on Gc-Ms. *American Journal of Translational Research* (2016) 8(7):2889.

60. Drogan D, Dunn WB, Lin W, Buijsse B, Schulze MB, Langenberg C, et al. Untargeted Metabolic Profiling Identifies Altered Serum Metabolites of Type 2 Diabetes Mellitus in a Prospective, Nested Case Control Study. *Clin Chem* (2015) 61(3):487-97. Epub 2014/12/20. doi: 10.1373/clinchem.2014.228965.

61. Kaur P, Rizk N, Ibrahim S, Luo Y, Younes N, Perry B, et al. Quantitative Metabolomic and Lipidomic Profiling Reveals Aberrant Amino Acid Metabolism in Type 2 Diabetes. *Mol Biosyst* (2013) 9(2):307-17. Epub 2012/12/19. doi: 10.1039/c2mb25384d.

62. Suhre K, Meisinger C, Doring A, Altmaier E, Belcredi P, Gieger C, et al. Metabolic Footprint of Diabetes: A Multiplatform Metabolomics Study in an Epidemiological Setting. *PLoS One* (2010) 5(11):e13953. Epub 2010/11/19. doi: 10.1371/journal.pone.0013953.

63. Patel SG, Hsu JW, Jahoor F, Coraza I, Bain JR, Stevens RD, et al. Pathogenesis of a(-)Beta(+) Ketosis-Prone Diabetes. *Diabetes* (2013) 62(3):912-22. Epub 2012/11/20. doi: 10.2337/db12-0624.

64. GOU Xiao-jun ZS-x, LI Guang-ping, ZHANG Cheng, CHENG Wen, CHEN Fei, WANG Hua, ZHANG Lu, CHEN Yue. Urinary Metabolomics Study in Patients with Diabetic Peripheral Neuropathy. *Chinese Journal of Hospital Pharmacy* (2019) 039(024):2512-9.

65. Jinkui ZXYFLJCXYGXRFJY. Plasma Metabolomic Profiling of Proliferative Diabetic Retinopathy. *Journal of Capital Medical University* (2020).

66. Jin H, Zhu B, Liu X, Jin J, Zou H. Metabolic Characterization of Diabetic Retinopathy: An (1)H-Nmr-Based Metabolomic Approach Using Human Aqueous Humor. *J Pharm Biomed Anal* (2019) 174:414-21. Epub 2019/06/19. doi: 10.1016/j.jpba.2019.06.013.

67. Chen L, Cheng CY, Choi H, Ikram MK, Sabanayagam C, Tan GS, et al. Plasma Metabonomic Profiling of Diabetic Retinopathy. *Diabetes* (2016) 65(4):1099-108. Epub 2016/01/30. doi: 10.2337/db15-0661.

68. Kunikata H, Ida T, Sato K, Aizawa N, Sawa T, Tawarayama H, et al. Metabolomic Profiling of Reactive Persulfides and Polysulfides in the Aqueous and Vitreous Humors. *Sci Rep* (2017) 7:41984. Epub 2017/02/09. doi: 10.1038/srep41984.

69. Zongmiao J. Preliminary Screening of Metabolic Markers Reiated to Diabetic Nephropathy (2019).

70. Yuhua M. Urine Metababonomics Study on the Biochemical Profiles of Diabetic Nephropathy: Xinjiang University (2014).

71. Xufang W, Mengjie L, Yongchun G, Weisong Q, Jiye A, Jinhua H, et al. Serum and Urinary Metabolomic Analysis in Patients with Diabetic Nephropathy. *Chinese Journal of Nephrology,Dialysis & Transplantation* (2012) (3):201-9.

72. Yang L. Metabonomics Study on the Biochemical Profiles of Diabetic Nephropathy: Xin (2011).

73. Sharma K, Karl B, Mathew AV, Gangoiti JA, Wassel CL, Saito R, et al. Metabolomics Reveals Signature of Mitochondrial Dysfunction in Diabetic Kidney Disease. *J Am Soc Nephrol* (2013) 24(11):1901-12. Epub 2013/08/21. doi: 10.1681/ASN.2013020126.

74. Ibarra-Gonzalez I, Cruz-Bautista I, Bello-Chavolla OY, Vela-Amieva M, Pallares-Mendez R, Ruiz de Santiago YND, et al. Optimization of Kidney Dysfunction Prediction in Diabetic Kidney Disease Using Targeted Metabolomics. *Acta Diabetol* (2018) 55(11):1151-61. Epub 2018/09/03. doi: 10.1007/s00592-018-1213-0.

75. Liu Y, Chen X, Liu Y, Chen T, Zhang Q, Zhang H, et al. Metabolomic Study of the Protective Effect of Gandi Capsule for Diabetic Nephropathy. *Chem Biol Interact* (2019) 314:108815. Epub 2019/09/10. doi: 10.1016/j.cbi.2019.108815.

76. Tavares G, Venturini G, Padilha K, Zatz R, Pereira AC, Thadhani RI, et al. 1,5-Anhydroglucitol Predicts Ckd Progression in Macroalbuminuric Diabetic Kidney Disease: Results from Non-Targeted Metabolomics. *Metabolomics* (2018) 14(4):39. Epub 2019/03/05. doi: 10.1007/s11306-018-1337-9.

77. Jiang Hong SX, Jia Sha. The Mitochondria-Targeted Metabolic Tubular Injury in Diabetic Kidney Disease. *Cell Physiol Biochem* (2019). doi: 10.1159/000000011.

78. Chen CJ, Liao WL, Chang CT, Lin YN, Tsai FJ. Identification of Urinary Metabolite Biomarkers of Type 2 Diabetes Nephropathy Using an Untargeted Metabolomic Approach. *J Proteome Res* (2018) 17(11):3997-4007. Epub 2018/09/29. doi: 10.1021/acs.jproteome.8b00644.

79. Li L, Wang C, Yang H, Liu S, Lu Y, Fu P, et al. Metabolomics Reveal Mitochondrial and Fatty Acid Metabolism Disorders That Contribute to the Development of Dkd in T2dm Patients. *Mol Biosyst* (2017) 13(11):2392-400. Epub 2017/09/29. doi: 10.1039/c7mb00167c.

80. Zhang J, Yan L, Chen W, Lin L, Song X, Yan X, et al. Metabonomics Research of Diabetic Nephropathy and Type 2 Diabetes Mellitus Based on Uplc-Oatof-Ms System. *Anal Chim Acta* (2009) 650(1):16-22. Epub 2009/09/02. doi: 10.1016/j.aca.2009.02.027.

81. Devi S, Nongkhlaw B, Limesh M, Pasanna RM, Thomas T, Kuriyan R, et al. Acyl Ethanolamides in Diabetes and Diabetic Nephropathy: Novel Targets from Untargeted Plasma Metabolomic Profiles of South Asian Indian Men. *Sci Rep* (2019) 9(1):18117. Epub 2019/12/04. doi: 10.1038/s41598-019-54584-2.

82. Xia JF, Liang QL, Liang XP, Wang YM, Hu P, Li P, et al. Ultraviolet and Tandem Mass Spectrometry for Simultaneous Quantification of 21 Pivotal Metabolites in Plasma from Patients with Diabetic Nephropathy. *J Chromatogr B Analyt Technol Biomed Life Sci* (2009) 877(20-21):1930-6. Epub 2009/06/09. doi: 10.1016/j.jchromb.2009.05.047.

83. Pena MJ, Lambers Heerspink HJ, Hellemons ME, Friedrich T, Dallmann G, Lajer M, et al. Urine and Plasma Metabolites Predict the Development of Diabetic Nephropathy in Individuals with Type 2 Diabetes Mellitus. *Diabet Med* (2014) 31(9):1138-47. Epub 2014/03/26. doi: 10.1111/dme.12447.

84. Solini A, Manca ML, Penno G, Pugliese G, Cobb JE, Ferrannini E. Prediction of Declining Renal Function and Albuminuria in Patients with Type 2 Diabetes by Metabolomics. *J Clin Endocrinol Metab* (2016) 101(2):696-704. Epub 2015/12/20. doi: 10.1210/jc.2015-3345.

85. Zhu C, Liang QL, Hu P, Wang YM, Luo GA. Phospholipidomic Identification of Potential Plasma Biomarkers Associated with Type 2 Diabetes Mellitus and Diabetic Nephropathy. *Talanta* (2011) 85(4):1711-20. Epub 2011/08/30. doi: 10.1016/j.talanta.2011.05.036.
