# Supplementary material for: Clinical metabolomics in type 2 diabetes mellitus: from pathogenesis to biomarkers
Source: Front Endocrinol (Lausanne). 2025 Feb 25;16:1501305. doi: 10.3389/fendo.2025.1501305 (PMC11893406; doi:10.3389/fendo.2025.1501305)
Supplement: Supplementary file 3 [file SupplementaryFile3.docx]

**Clinical metabolomics in type 2 diabetes mellitus: from pathogenesis to biomarkers**

Jiao Kong^2,†^，Hetao Chen^1,3,†^，Yujin Ma^1^，Lei Zhang^4^，Lulu Chen^1,3^，Jiarui Huang^5^, Zizhe Zhao^1^, Hongwei Jiang^1,*^, Chuanxin Liu^1,*^

^1^ Luoyang Key Laboratory of Clinical Multiomics and Translational Medicine, Henan Key Laboratory of Rare Diseases, Endocrinology and Metabolism Center, The First Affiliated Hospital, and College of Clinical Medicine of Henan University of Science and Technology, Luoyang, China,

^2^ Institute of Drug Metabolism and Pharmaceutical Analysis, College of Pharmaceutical Sciences, Zhejiang University, Hangzhou, China,

^3^ Department of Clinical Laboratory, The First Affiliated Hospital, College of Clinical Medicine of Henan University of Science and Technology, Luoyang, China,

^4^ Department of Integrative Medicine, The First Affiliated Hospital, and College of Clinical Medicine of Henan University of Science and Technology, Luoyang, China,

^5^ Department of Critical Care Medicine, The First Affiliated Hospital, and College of Clinical Medicine of Henan University of Science and Technology, Luoyang, China

^†^ These authors contributed equally to this work and share first authorship

*Correspondence:HongweiJiang✉:jianghw@haust.edu.cn;ChuanxinLiu✉:15222003775@163.com.

**Table S2. Differential metabolites of T2DM and its complications based on clinical metabolomics.**

| No | Metabolites | Class | Sample | Analysis platform | Level | Quantify | Stages | Ref |
| --- | --- | --- | --- | --- | --- | --- | --- | --- |
| 1 | Glycine | Carboxylic acids and derivatives | Serum | UPLC-LTQ Orbitrap | ↓ | - | PM | (1) |
|  |  |  | Plasma | LC-MS/MS | ↓ | √ | PM | (2) |
|  |  |  | Serum | LC-FIA-ESI-MS/MS | ↓ | √ | PM | (3) |
|  |  |  | Serum | LC-MS | - | - | PM | (4) |
|  |  |  | Serum | UHPLC-MS | ↓ | - | PM | (5) |
|  |  |  | Serum | NMR | ↓ | - | PM | (6) |
|  |  |  | Serum | GC-MS | ↓ | - | T2DM | (7) |
|  |  |  | Plasma | LC-MS/MS | - | √ | T2DM | (8) |
|  |  |  | Serum | LC-MS & GC-MS | ↑ | - | T2DM | (9) |
|  |  |  | Plasma | LC-MS/MS | - | √ | T2DM | (10) |
|  |  |  | Plasma | LC-MS & GC-MS | - | - | T2DM | (11) |
|  |  |  | Plasma | LC-MS/MS | ↑ | √ | T2DK | (12) |
|  |  |  | Serum | GC-MS | ↓ | - | T2DPN | (13) |
|  |  |  | Plasma | HPLC-QTrap-MS/MS | ↑/↑ | √ | T2DN | (14) |
|  |  |  | Urine | GC-TOF/MS | ↓ | - | T2DN | (15) |
|  |  |  | Serum | UPLC-oaTOF-MS | ↑ | - | T2DN | (16) |
| 2 | Lysine | Carboxylic acids and derivatives | Serum | UPLC-LTQ Orbitrap | ↓ | - | PM | (1) |
|  |  |  | Plasma | UPLC-Q-TOF/MS | ↑ | - | PM | (17) |
|  |  |  | Plasma | UPLC-Q-TOF/MS | ↑ | - | T2DM | (18) |
|  |  |  | Serum | UPLC-oaTOF-MS | ↑ | - | T2DN | (16) |
| 3 | trans-Cinnamic acid | Cinnamic acids and derivatives | Serum | UPLC-LTQ Orbitrap | ↓ | - | PM | (1) |
| 4 | LysoPC[18:2(9Z,12Z)] | Glycerophospholipids | Serum | UPLC-LTQ Orbitrap | ↓ | - | PM | (1) |
| 5 | LysoPC(16:0) | Glycerophospholipids | Serum | UPLC-LTQ Orbitrap | ↓ | - | PM | (1) |
|  |  |  | Plasma | UPLC-LTQ-Orbitrap | - | - | PM | (19) |
|  |  |  | Serum | LC-FIA-ESI-MS/MS | ↑ | √ | PM | (3) |
|  |  |  | Serum | LC-MS | - | - | PM | (20) |
|  |  |  | Plasma | UPLC-Q-TOF/MS | ↑ | - | T2DM | (18) |
|  |  |  | Serum | UPLC-MS | - | - | T2DM | (21) |
|  |  |  | Plasma | UPLC-MS/MS | ↑ | √ | T2DN | (22) |
| 6 | LysoPC[16:1(9Z)] | Glycerophospholipids | Serum | UPLC-LTQ Orbitrap | ↓ | - | PM | (1) |
| 7 | LysoPC(14:0) | Glycerophospholipids | Serum | UPLC-LTQ Orbitrap | ↓ | - | PM | (1) |
|  |  |  | Plasma | UPLC-LTQ-Orbitrap | - | - | PM | (19) |
|  |  |  | Plasma | UPLC-Q-TOF/MS | ↑ | - | T2DM | (18) |
|  |  |  | Serum | LC-MS & GC-MS | ↓ | - | T2DN | (23) |
| 8 | Leucine | Carboxylic acids and derivatives | Plasma | 1H-NMR | ↓ | - | PM | (24) |
|  |  |  |  | UPLC-Q-TOF/MS | - | - | PM | (6) |
|  |  |  |  | UPLC-Q Exactive/MS | ↑ | - | PM | (25) |
|  |  |  |  | NMR | ↑ | - | PM | (26) |
|  |  |  |  | UHPLC-MS | ↑ | - | PM | (5) |
|  |  |  |  | UPLC-Q-TOF/MS | ↓ | - | PM | (17) |
|  |  |  |  | LC-MS/MS | ↑ | √ | PM | (2) |
|  |  |  |  | UPLC-LTQ-Orbitrap | - | - | PM | (19) |
|  |  |  | Serum | GC-MS | ↑ | - | T2DM | (7) |
|  |  |  | Plasma | NMR | ↑ | - | T2DM | (27) |
|  |  |  | Plasma/Urine | NMR | ↓ | - | T2DM | (28) |
|  |  |  | Plasma | UPLC-Q-TOF/MS | ↓ | - | T2DM | (29) |
|  |  |  | Serum | LC-MS/MS | ↑ | √ | T2DM | (30) |
|  |  |  | Serum | LC-MS & GC-MS | ↑ | - | T2DM | (9) |
|  |  |  | Plasma | UPLC-Q-TOF/MS | ↑ | - | T2DM | (18) |
|  |  |  | Urine | GC-MS | ↓ | - | T2DM | (31) |
|  |  |  | Plasma | UPLC-Q-TOF/MS | ↑ | √ | T2DM | (32) |
|  |  |  | Plasma | HPLC-QTrap-MS/MS | ↑/↑ | √ | T2DN | (14) |
|  |  |  | Serum | UPLC-oaTOF-MS | ↓ | - | T2DN | (16) |
| 9 | Valine | Carboxylic acids and derivatives | Plasma | ^1^H-NMR | ↓ | - | PM | (24) |
|  |  |  |  | UPLC-Q-TOF/MS | - | - | PM | (6) |
|  |  |  |  | UPLC-Q Exactive/MS | ↑ | - | PM | (25) |
|  |  |  |  | NMR | ↑ | - | PM | (26) |
|  |  |  |  | UPLC-Q-TOF/MS | ↑ | - | PM | (17) |
|  |  |  |  | LC-MS/MS | ↑ | √ | PM | (2) |
|  |  |  |  | UPLC-LTQ-Orbitrap | - | - | PM | (19) |
|  |  |  | Plasma | LC-MS | ↑ | - | PM | (33) |
|  |  |  | Serum | GC-MS | ↑ | - | T2DM | (7) |
|  |  |  | Plasma | GC-MS | ↑ | - | T2DM | (34) |
|  |  |  | Serum | ^1^H-NMR | ↓ | - | T2DM | (35) |
|  |  |  | Plasma | NMR | ↑ | - | T2DM | (27) |
|  |  |  | Plasma/Urine | NMR | ↓ | - | T2DM | (27) |
|  |  |  | Serum | LC-MS/MS | ↑ | √ | T2DM | (30) |
|  |  |  | Serum | LC-MS & GC-MS | ↑ | - | T2DM | (9) |
|  |  |  | Plasma | LC-MS/MS | - | - | T2DM | (10) |
|  |  |  | Serum | UPLC-Q-Exactive | - | - | T2DM | (36) |
|  |  |  | Plasma | HPLC-QTrap-MS/MS | ↑/↑ | √ | T2DN | (14) |
|  |  |  | Serum | LC-MS & GC-MS | ↑ | - | T2DN | (23) |
|  |  |  | Blood | HPLC-MRM | ↑ | √ | T2DN | (37) |
|  |  |  | Urine | UPLC-Q-TOF/MS | - | - | T2DN | (38) |
| 10 | Alanine | Carboxylic acids and derivatives | Plasma | ^1^H-NMR | ↓ | - | PM | (24) |
|  |  |  | Plasma | GC-MS/MS | ↑ | - | PM | (39) |
| 11 | Proline | Carboxylic acids and derivatives | Plasma | ^1^H-NMR | ↑ | - | PM | (24) |
|  |  |  |  | UPLC-Q-TOF/MS | ↓ | - | PM | (17) |
|  |  |  | Serum | ^1^H-NMR | ↓ | - | T2DM | (35) |
|  |  |  | Plasma | LC-MS/MS | ↑ | √ | T2DM | (12) |
| 12 | Creatine | Carboxylic acids and derivatives | Plasma | ^1^H-NMR | ↓ | - | PM | (24) |
|  |  |  |  | LC-MS/MS | ↑ | - | PM | (2) |
| 13 | Choline | Organonitrogen compounds | Plasma | ^1^H-NMR | ↓ | - | PM | (24) |
|  |  |  | Plasma | HPLC-UV-MS/MS | ↓ | √ | T2DN | (40) |
|  |  |  | Plasma | UPLC-Q-TOF/MS | ↓ | - | PM | (29) |
|  |  |  | Serum | UPLC-QTRAP | ↓ | - | T2DM | (41) |
| 14 | Histidine | Carboxylic acids and derivatives | Plasma | ^1^H-NMR | ↑ | - | PM | (24) |
|  |  |  | Serum | ^1^H-NMR | ↑ | - | T2DM | (35) |
|  |  |  | Plasma | NMR | ↑ | - | T2DM | (27) |
|  |  |  | Plasma | LC-MS/MS | - | √ | T2DM | (10) |
|  |  |  | Plasma | LC-MS/MS | ↑ | √ | T2DK | (12) |
|  |  |  | Aqueous humor | NMR | ↑ | - | T2DN | (42) |
|  |  |  | Plasma | HPLC-QTrap-MS/MS | ↑/↑ | √ | T2DN | (14) |
|  |  |  | Urine | UPLC-Q-TOF/MS | - | - | T2DN | (38) |
|  |  |  | Plasma/Urine | HPLC-FIA-MS/MS | ↑ | √ | T2DN | (43) |
| 15 | α-Glucose | Organic Oxygen compounds | Plasma | ^1^H-NMR | ↑ | - | PM | (24) |
|  |  |  | Urine | GC-MS | ↑ | - | PM | (44) |
|  |  |  | Plasma | GC-SIM-MS | ↑ | - | PM | (45) |
|  |  |  | Plasma | LC-MS | ↑ | - | PM | (33) |
|  |  |  | Plasma | UPLC-Q-TOF/MS | - | - | PM | (6) |
|  |  |  | Serum | ^1^H-NMR | ↑ | - | T2DM | (35) |
|  |  |  | Serum | GC-MS | ↑ | - | T2DM | (7) |
|  |  |  | Blood | LC-MS | - | - | T2DM | (46) |
|  |  |  | Plasma | NMR | ↑ | - | T2DM | (27) |
|  |  |  | Plasma | NMR | ↑ | - | T2DM | (29) |
|  |  |  | Plasma | GC×GC-MS | ↑ | - | T2DM | (47) |
|  |  |  | Plasma | LC-MS/MS | - | √ | T2DM | (8) |
|  |  |  | Plasma | LC-MS & GC-MS | - | - | T2DM | (11) |
|  |  |  | Urine | GC-MS | ↑ | - | T2DM | (31) |
|  |  |  | Plasma | UHPLC-MS/MS | - | √ | T2DM | (48) |
|  |  |  | Urine | GC-MS | - | - | T2DM | (49) |
|  |  |  | Serum | LC-MS & GC-MS | ↑ | - | T2DN | (23) |
| 16 | β-Glucose | Organic Oxygen compounds | Plasma | ^1^H-NMR | ↑ | - | PM | (24) |
|  |  |  | Urine | GC-MS | ↑ | - | PM | (44) |
|  |  |  | Plasma | GC-SIM-MS | ↑ | - | PM | (45) |
|  |  |  | Plasma | LC-MS | ↑ | - | PM | (33) |
|  |  |  | Plasma | UPLC-TOF/MS | - | - | PM | (6) |
| 17 | Arachidyl carnitine | Fatty acyls | Serum | UPLC-TOF/MS | - | - | PM | (50) |
| 18 | Trihexosylcerarnide | Sphingolipids | Serum | UPLC-TOF/MS | - | - | PM | (50) |
| 19 | Ganglioside GA2(d18:1/22:0) | Sphingolipids | Serum | UPLC-TOF/MS | - | - | PM | (50) |
| 20 | Vitamin D2 3-glucuronide | Steroids and steroid derivatives | Serum | UPLC-TOF/MS | - | - | PM | (50) |
| 21 | Diguanosine diphosphate | (5'->5')-dinucleotides | Serum | UPLC-TOF/MS | - | - | PM | (50) |
| 22 | PC(22:6/20:4) | Glycerophospholipids | Serum | UPLC-TOF/MS | - | - | PM | (50) |
| 23 | PE(15:0/18:3) | Glycerophospholipids | Serum | UPLC-TOF/MS | - | - | PM | (50) |
| 24 | PI(18:0/16:0) | Glycerophospholipids | Serum | UPLC-TOF/MS | - | - | PM | (50) |
| 25 | PS(20:4/18:0) | Glycerophospholipids | Serum | UPLC-TOF/MS | - | - | PM | (50) |
| 26 | PG(16:0/22:5) | Glycerophospholipids | Serum | UPLC-TOF/MS | - | - | PM | (50) |
| 27 | PC[14:1(9Z)/22:1(13Z) | Glycerophospholipids | Plasma | UPLC-Q Exactive/MS | ↓ | - | PM | (51) |
| 28 | Lactic acid | Hydroxy acids and derivatives | Plasma | UPLC-Q Exactive/MS | ↑ | - | PM | (51) |
|  |  |  | Serum | LC-MS & GC-MS | ↑ | - | T2DM | (9) |
| 29 | PC[16:1(9Z)/20:3(8Z，11Z，14Z) | Glycerophospholipids | Plasma | UPLC-Q Exactive/MS | ↓ | - | PM | (51) |
| 30 | Linoleic acid | Fatty acyls | Plasma | UPLC-Q Exactive/MS | ↑ | - | PM | (51) |
|  |  |  |  | HPLC-QqQ-MS/MS | - | √ | PM | (3) |
|  |  |  |  | UPLC-Q-TOF/MS | - | - | PM | (52) |
|  |  |  | Plasma | UPLC-Q-TOF/MS | ↓ | - | T2DM | (29) |
|  |  |  | Serum | LC-MS & GC-MS | ↑ | - | T2DM | (9) |
|  |  |  | Serum | GC-MS | ↓ | - | T2DPN | (13) |
|  |  |  | Plasma | UHPLC-Q-Exactive/MS | ↓ | - | T2DN | (53) |
| 31 | Oleic acid | Fatty acyls | Plasma | UPLC-Q Exactive/MS | ↑ | - | PM | (51) |
|  |  |  | Serum | HPLC-QqQ-MS/MS | - | √ | PM | (3) |
|  |  |  | Plasma | LC-MS/MS | ↑ | √ | PM | (2) |
|  |  |  | Serum | LC-MS & GC-MS | ↑ | - | T2DM | (9) |
|  |  |  | Urine | GC-MS | ↑ | - | T2DM | (31) |
|  |  |  | Plasma | UHPLC-Q-Exactive/MS | ↓ | - | T2DN | (53) |
| 32 | Palmitic acid | Fatty acyls | Plasma | UPLC-Q-Exactive/MS | ↑ | - | PM | (51) |
|  |  |  | Urine | UPLC-Q-TOF/MS | ↑ | - | T2DM | (54) |
|  |  |  | Plasma | UPLC-Q-TOF/MS | ↓ | - | T2DM | (29) |
|  |  |  | Serum | LC-MS & GC-MS | ↑ | - | T2DM | (9) |
|  |  |  | Urine | GC-MS | ↑ | - | T2DM | (31) |
|  |  |  | Serum | GC-MS | ↓ | - | T2DM | (13) |
|  |  |  | Serum | LC-MS & GC-MS | ↑ | - | T2DN | (23) |
|  |  |  | Urine | GC-TOF/MS | ↑ | - | T2DN | (23) |
|  |  |  | Plasma | UPLC-Q-Exactive/MS | ↓ | - | T2DN | (16) |
| 33 | SM[d18:0/16:1(9Z)] | Sphingolipids | Plasma | UPLC-Q-Exactive/MS | ↓ | - | PM | (51) |
| 34 | Betaine | Carboxylic acids and derivatives | Plasma | UPLC-Q-Exactive/MS | ↑ | - | PM | (51) |
|  |  |  | Serum | 1H-NMR | ↓ | - | T2DM | (35) |
| 35 | Arachidonic acid | Fatty acyls | Plasma | UPLC-Q-Exactive/MS | ↑ | - | PM | (51) |
|  |  |  | Urine | UPLC-Q-TOF/MS | ↑ | - | T2DM | (54) |
|  |  |  | Urine | GC-MS | ↑ | - | T2DM | (31) |
|  |  |  | Plasma | UPLC-Q-Exactive/MS | ↑ | - | T2DN | (53) |
| 36 | Uric acid | Imidazopyrimidines | Serum | HPLC-MS/MS | ↓ | √ | PM | (25) |
|  |  |  | Plasma | UPLC-Q-TOF/MS | ↑ | - | PM | (20) |
|  |  |  | Plasma | GC-MS/MS | - | - | T2DM | (55) |
|  |  |  | Plasma | UPLC-Q-TOF/MS | ↓ |  | T2DM | (32) |
|  |  |  | Urine | UPLC-Q-TOF/MS | - | - | T2DN | (38) |
|  |  |  | Urine | GC-TOF/MS | ↑ | - | T2DN | (15) |
|  |  |  | Plasma | HPLC-UV-MS/MS | ↑ | √ | T2DN | (40) |
| 37 | Xanthine | Imidazopyrimidines | Serum | HPLC-MS/MS | ↑ | √ | PM | (25) |
|  |  |  | Serum | LC-MS | - | - | PM | (20) |
|  |  |  | Urine | UPLC-Q-TOF/MS | - | - | T2DN | (38) |
|  |  |  | Plasma | HPLC-UV-MS/MS | ↑ | √ | T2DN | (40) |
| 38 | Creatinine | Carboxylic acids and derivatives | Serum | HPLC-MS/MS | ↓ | √ | PM | (25) |
|  |  |  | Plasma | NMR | ↑ | - | T2DM | (27) |
|  |  |  | Plasma | HPLC-UV-MS/MS | ↑ | √ | T2DN | (40) |
| 39 | MG(22:2(13Z,16Z)/0:0/0:0) | Glycerolipids | Plasma | UPLC-Q-TOF/MS | ↓ | - | PM | (20) |
| 40 | LysoPC (15:0) | Glycerophospholipids | Plasma | UPLC-Q-TOF/MS | ↓ | - | PM | (20) |
| 41 | Glycochenodeoxycholic-3-glucuronide | Steroids and steroid derivatives | Plasma | UPLC-Q-TOF/MS | ↓ | - | PM | (20) |
| 42 | LysoPE (0:0/16:0) | Glycerophospholipids | Plasma | UPLC-Q-TOF/MS | - | - | PM | (20) |
| 43 | LysoPC(17:0) | Glycerophospholipids | Plasma | UPLC-Q-TOF/MS | ↑ | √ | PM | (20) |
|  |  |  |  | UPLC-LTQ-Orbitrap | - | - | PM | (19) |
|  |  |  |  | LC-MS | ↓ | - | PM | (4) |
|  |  |  | Serum | LC-Q-TOF/MS | - | - | T2DM | (56) |
| 44 | LysoPC(18:0) | Glycerophospholipids | Plasma | UPLC-Q-TOF/MS | - | - | PM | (20) |
|  |  |  |  | UPLC-LTQ-Orbitrap | ↑ | - | PM | (19) |
|  |  |  |  | HPLC-Q-Trap/MS | ↓ | - | PM | (57) |
|  |  |  | Serum | LC-FIA-ESI-MS/MS | ↑ | √ | PM | (3) |
|  |  |  | Plasma | UPLC-Q-TOF/MS | ↓ | - | T2DM | (18) |
|  |  |  | Plasma | UPLC-MS/MS | ↑ | √ | T2DN | (22) |
| 45 | LysoPC[(18:1)9Z] | Glycerophospholipids | Plasma | UPLC-Q-TOF/MS | - | - | PM | (20) |
| 46 | LysoPC[(18:2)9Z,12Z] | Glycerophospholipids | Plasma | UPLC-Q-TOF/MS | - | - | PM | (20) |
| 47 | LysoPE[(0/0:20:1)11Z] | Glycerophospholipids | Plasma | UPLC-Q-TOF/MS | - | - | PM | (20) |
| 48 | S-(PGA2)-glutathione | Carboxylic acids and derivatives | Plasma | UPLC-Q-TOF/MS | - | - | PM | (20) |
| 49 | Phytosphingosine | Organonitrogen compounds | Plasma | UPLC-Q-TOF/MS | - | - | PM | (20) |
|  |  |  | Serum | UPLC-oaTOF-MS | ↓ | - | T2DN | (16) |
| 50 | Citrate | Salts | Serum | GC-MS | - | - | PM | (58) |
|  |  |  |  | NMR | ↑ | - | PM | (6) |
|  |  |  | Serum | 1H-NMR | ↓ | - | T2DM | (35) |
|  |  |  | Plasma | NMR | ↓ | - | T2DM | (27) |
|  |  |  | Plasma/Urine | NMR | ↓ | - | T2DM | (27) |
| 51 | cis-Aconitic acid | Carboxylic acids and derivatives | Serum | GC-MS | - | - | PM | (58) |
|  |  |  | Urine | GC-MS | ↓ | - | T2DN | (59) |
|  |  |  | Urine | GC-TOF/MS | ↓ | - | T2DN | (15) |
| 52 | Fumarate | Fatty acyls | Serum | GC-MS | - | - | PM | (58) |
|  |  |  | Serum | LC-MS/MS | ↑ | - | T2DM | (60) |
| 53 | Succinate | Fatty acyls | Serum | GC-MS | - | - | PM | (58) |
|  |  |  | Serum | 1H-NMR | ↓ | - | T2DM | (35) |
|  |  |  | Serum | LC-MS/MS | ↑ | √ | T2DM | (60) |
|  |  |  | Plasma | UPLC-Q-TOF/MS | ↓ | √ | T2DM | (32) |
| 54 | Pyruvate | Keto acids and derivatives | Serum | GC-MS | - | - | PM | (58) |
|  |  |  |  | NMR | ↑ | - | PM | (6) |
|  |  |  | Serum | 1H-NMR | ↓ | - | T2DM | (35) |
|  |  |  | Plasma | NMR | ↑ | - | T2DM | (27) |
|  |  |  | Serum | NMR | ↑ | - | T2DM | (27) |
|  |  |  | Serum | LC-MS & GC-MS | ↑ | - | T2DM | (9) |
|  |  |  | Serum | LC-MS | - | - | T2DM | (61) |
| 55 | Malate | Hydroxy acids and derivatives | Serum | GC-MS | - | - | PM | (58) |
|  |  |  | Plasma | LC-MS | ↑ | - | PM | (33) |
| 56 | Pyroglutamic acid | Carboxylic acids and derivatives | Serum | GC-MS | - | - | PM | (58) |
|  |  |  | Plasma | UPLC-Q-TOF/MS | ↓ | - | T2DM | (29) |
| 57 | Alkylresorcinols C17 | NA | Plasma | GC-MS/MS | - | - | PM | (39) |
| 58 | Alkylresorcinols C19 | NA | Plasma | GC-MS/MS | - | - | PM | (39) |
| 59 | Eicosapentaenoic acid | Fatty acyls | Plasma | GC-MS/MS | - | - | PM | (39) |
| 60 | 3-Carboxy-4-methyl-5-propyl-2-furanpropanoic acid | Fatty acyls | Plasma | GC-MS/MS | - | - | PM | (39) |
| 61 | α-Tocopherol | Prenol lipids | Plasma | GC-MS/MS | - | - | PM | (39) |
| 62 | LysoPC(18:1) | Glycerophospholipids | Serum | LC-FIA-ESI-MS/MS | - | - | PM | (19) |
|  |  |  |  | LC-QTRAP-MS/MS | ↑ | - | PM | (57) |
|  |  |  |  | LC-FIA-ESI-MS/MS | ↑ | √ | PM | (3) |
|  |  |  | Plasma | LC-MS | ↓ | - | PM | (4) |
|  |  |  | Plasma | UPLC-Q-TOF/MS | ↑ | - | T2DM | (18) |
|  |  |  | Plasma | UPLC-MS/MS | ↑ | √ | T2DN | (22) |
| 63 | LysoPC(18:2) | Glycerophospholipids | Serum | LC-FIA-ESI-MS/MS | ↑ | √ | PM | (3) |
|  |  |  |  | - | - | - | PM | (19) |
|  |  |  | Serum | LC-MS | - | - | PM | (4) |
|  |  |  | Serum | LC-MS/MS | - | √ | T2DM | (8) |
|  |  |  | Plasma | UPLC-Q-TOF/MS | ↑ | - | T2DM | (18) |
| 64 | Glutamate | Carboxylic acids and derivatives | Serum | LC-FIA-ESI-MS/MS | ↑ | √ | PM | (3) |
|  |  |  | Serum | 1H-NMR | ↓ | - | T2DM | (35) |
|  |  |  | Plasma | LC-MS/MS | ↑ | √ | T2DM | (62) |
| 65 | LysoPE(18:0) | Glycerophospholipids | Serum | LC-FIA-ESI-MS/MS | ↑ | √ | PM | (3) |
| 66 | LysoPE(18:1) | Glycerophospholipids | Serum | LC-FIA-ESI-MS/MS | ↑ | √ | PM | (3) |
| 67 | α-Hydroxybutyric Acid | Hydroxy acids and derivative | Plasma | LC-MS/MS | ↑ | - | PM | (2) |
| 68 | α-Ketobutyric acid | Keto acids and derivatives | Plasma | LC-MS/MS | ↑ | - | PM | (2) |
| 69 | α-Ketoglutaric acid | Keto acids and derivatives | Plasma | LC-MS/MS | ↑ | - | PM | (2) |
| 70 | β-Hydroxybutyric Acid | Hydroxy acids and derivatives | Plasma | LC-MS/MS | ↑ | - | PM | (2) |
| 71 | 2-Aminoadipic acid | Carboxylic acids and derivatives | Plasma | LC-MS/MS | ↑ | - | PM | (2) |
|  |  |  | Plasma | UPLC-QTRAP | ↓ | √ | T2DM | (63) |
| 72 | 3-Hydroxyisobutyric acid | Hydroxy acids and derivatives | Plasma | LC-MS/MS | ↑ | - | PM | (2) |
| 73 | 3-Methyl-2-oxobutyric acid | Keto acids and derivatives | Plasma | LC-MS/MS | ↑ | - | PM | (2) |
| 74 | 3-Methyl-2-oxopentanoic acid | Keto acids and derivatives | Plasma | LC-MS/MS | ↑ | - | PM | (2) |
|  |  |  | Plasma | GC-MS/MS | - | - | T2DM | (55) |
| 75 | 4-Methyl-2-oxobutyric acid | Organic acid | Plasma | LC-MS/MS | ↑ | - | PM | (2) |
| 76 | Hydroxyisovaleroyl carnitine | Fatty acyls | Plasma | LC-MS/MS | ↑ | - | PM | (2) |
| 77 | Isoleucine | Carboxylic acids and derivatives | Plasma | LC-MS/MS | ↑ | - | PM | (2) |
|  |  |  | Serum | NMR | ↑ | - | PM | (26) |
|  |  |  | Serum | UHPLC-MS | ↑ | - | PM | (5) |
|  |  |  | Plasma | UPLC-Q-TOF/MS | ↑ | - | PM | (17) |
|  |  |  | Plasma | UPLC-Q-TOF/MS | - | - | PM | (6) |
|  |  |  | Plasma/Urine | - | - | - | PM | (33) |
|  |  |  | Serum | GC-MS | ↑ | - | T2DM | (7) |
|  |  |  | Plasma | NMR | ↓ | - | T2DM | (27) |
|  |  |  | Serum/Urine | NMR | ↑ | - | T2DM | (27) |
|  |  |  | Serum | LC-MS/MS | ↑ | √ | T2DM | (30) |
|  |  |  | Serum | LC-MS/MS | - | - | T2DM | (56) |
|  |  |  | Serum | LC-MS & GC-MS | ↑ | - | T2DM | (9) |
|  |  |  | Plasma | LC-MS/MS | - | √ | T2DM | (10) |
|  |  |  | Plasma | UPLC-Q-Exactive | - | - | T2DM | (36) |
|  |  |  | Serum | LC-MS | - | - | T2DM | (61) |
|  |  |  | Urine | GC-MS | ↓ | - | T2DM | (31) |
|  |  |  | Serum | LC-MS & GC-MS | ↑ | - | T2DN | (32) |
|  |  |  | Urine | UPLC-Q-TOF/MS | ↑ | - | T2DN | (64) |
| 78 | Linoleoyl-glycerophosphocholine | NA | Plasma | LC-MS/MS | ↑ | √ | PM | (2) |
| 79 | Phenylalanine | Carboxylic acids and derivatives | Plasma | LC-MS/MS | ↑ | √ | PM | (2) |
|  |  |  |  | UPLC-Q-TOF/MS | ↓ | - | PM | (65) |
|  |  |  |  | UPLC-Q-TOF/MS | ↑ | - | PM | (17) |
|  |  |  |  | UPLC-LTQ-Orbitrap | - | - | PM | (19) |
|  |  |  | Serum | NMR | ↑ | - | PM | (6) |
|  |  |  | Urine | UPLC-Q-TOF/MS | ↑ | - | T2DM | (54) |
|  |  |  | Serum | 1H-NMR | ↓ | - | T2DM | (35) |
|  |  |  | Serum | GC-MS | ↓ | - | T2DM | (7) |
|  |  |  | Plasma | NMR | ↑ | - | T2DM | (27) |
|  |  |  | Plasma | NMR | ↑ | - | T2DM | (29) |
|  |  |  | Plasma | LC-MS/MS | - | √ | T2DM | (8) |
|  |  |  | Serum | LC-Q-TOF/MS | - | - | T2DM | (56) |
|  |  |  | Plasma | LC-MS/MS | - | √ | T2DM | (10) |
|  |  |  | Plasma | UPLC-Q-TOF/MS | ↑ | - | T2DM | (18) |
|  |  |  | Urine | LC-MS/MS | ↓ | √ | T2DM | (66) |
|  |  |  | Urine | GC-MS | ↑ | - | T2DM | (31) |
|  |  |  | Plasma | UHPLC-MS/MS | - | √ | T2DM | (48) |
|  |  |  | Blood | GC-MS | ↑ | √ | T2DN | (37) |
|  |  |  | Urine | UPLC-Q-TOF/MS | ↑ | - | T2DN | (64) |
|  |  |  | Plasma | GC-MS | - | - | T2DN | (67) |
| 80 | Serine | Carboxylic acids and derivatives | Plasma | LC-MS/MS | ↓ | - | PM | (2) |
|  |  |  | Urine | GC-MS | - | - | PM | (44) |
|  |  |  | Serum | GC-MS | ↓ | - | T2DM | (7) |
|  |  |  | Serum | LC-MS & GC-MS | ↓ | - | T2DM | (9) |
|  |  |  | Plasma | LC-MS/MS | - | √ | T2DM | (10) |
|  |  |  | Plasma | UPLC-Q-TOF/MS | ↓ | - | T2DM | (18) |
|  |  |  | Plasma | LC-MS/MS | ↑ | √ | T2DK | (12) |
|  |  |  | Urine | GC-TOF/MS | ↓ | - | T2DN | (15) |
| 81 | Trigonelline | Alkaloids and derivatives | Plasma | LC-MS/MS | ↑ | - | PM | (2) |
| 82 | Tyrosine | Carboxylic acids and derivatives | Plasma | LC-MS/MS | ↑ | - | PM | (2) |
|  |  |  | Serum | NMR | ↑ | - | PM | (6) |
|  |  |  | Plasma | NMR | ↑ | - | T2DM | (27) |
|  |  |  | Plasma/Urine | NMR | ↓ | - | T2DM | (28) |
|  |  |  | Plasma | LC-MS/MS | - | √ | T2DM | (68) |
|  |  |  | Urine | GC-MS | ↓ | - | T2DM | (31) |
|  |  |  | Blood | HPLC-MRM | ↓ | - | T2DN | (37) |
| 83 | Pantothenic acid | Organic oxygen compounds | Plasma | LC-MS/MS | ↑ | - | PM | (2) |
| 84 | Oleamide | Fatty acyls | Plasma | UPLC-LTQ-Orbitrap | - | - | PM | (19) |
| 85 | C17 Sphinganine | Organonitrogen compounds | Plasma | UPLC-LTQ-Orbitrap | - | - | PM | (19) |
| 86 | (4E,8E,10E-d18:3) Sphingosine | Organonitrogen compounds | Plasma | UPLC-LTQ-Orbitrap | - | - | PM | (19) |
| 87 | Anandamide (18:4, n-3) | Organonitrogen compounds | Plasma | UPLC-LTQ-Orbitrap | - | - | PM | (19) |
| 88 | LysoPC(16:1) | Glycerophospholipids | Plasma | UPLC-LTQ-Orbitrap | - | - | PM | (19) |
| 89 | LysoPC(18:3) | Glycerophospholipids | Plasma | UPLC-LTQ-Orbitrap | - | - | PM | (19) |
|  |  |  | Plasma | UPLC-Q-TOF/MS | ↑ | - | T2DM | (18) |
| 90 | LysoPC(20:5) | Glycerophospholipids | Plasma | UPLC-LTQ-Orbitrap | - | - | PM | (19) |
|  |  |  | Plasma | UPLC-Q-TOF/MS | ↑ | - | T2DM | (18) |
| 91 | LysoPC(20:4) | Glycerophospholipids | Plasma | UPLC-LTQ-Orbitrap | - | - | PM | (19) |
|  |  |  | Plasma | UPLC-MS/MS | ↑ | √ | T2DN | (22) |
| 92 | LysoPC (22:6) | Glycerophospholipids | Plasma | UPLC-LTQ-Orbitrap | - | - | PM | (19) |
|  |  |  | Plasma | UPLC-Q-TOF/MS | ↑ | - | T2DM | (18) |
| 93 | SM (d18:0/16:1) | Sphingolipids | Plasma | UPLC-LTQ-Orbitrap | - | - | PM | (19) |
| 94 | Lactosylceramide (d18:1/12:0) | NA | Plasma | UPLC-LTQ-Orbitrap | - | - | PM | (19) |
| 95 | 2,3-Butanediol | Organic Oxygen compounds | Plasma | UPLC-Q-TOF/MS | - | - | PM | (52) |
| 96 | Oleamide | Fatty acyls | Plasma | UPLC-Q-TOF/MS | - | - | PM | (52) |
|  |  |  | Plasma | UPLC-Q-TOF/MS | ↑ | - | T2DM | (18) |
|  |  |  | Serum | GC-MS | ↓ | - | T2DPN | (13) |
| 97 | Stearamide | Carboximidic acids and derivatives | Plasma | UPLC-Q-TOF/MS | - | - | PM | (52) |
| 98 | Decenedioic acid | Fatty acyls | Plasma | UPLC-Q-TOF/MS | - | - | PM | (52) |
| 99 | Ketooctanoic acid | Keto acids and derivatives | Plasma | UPLC-Q-TOF/MS | - | - | PM | (52) |
| 100 | Octenoic acid | Fatty acyls | Plasma | UPLC-Q-TOF/MS | - | - | PM | (52) |
| 101 | Malic acid | Hydroxy acids and derivatives | Plasma | UPLC-Q-TOF/MS | - | - | PM | (52) |
| 102 | Glucuronic acid | Organic Oxygen compounds | Plasma | UPLC-Q-TOF/MS | - | - | PM | (52) |
|  |  |  | Urine | UPLC-Q-TOF/MS | ↑ | - | T2DM | (54) |
| 103 | Phosphoglycolic acid | Organic phosphoric acids and derivatives | Plasma | UPLC-Q-TOF/MS | - | - | PM | (52) |
| 104 | p-Cresol sulfate | Organic sulfuric acids and derivatives | Plasma | UPLC-Q-TOF/MS | - | - | PM | (52) |
| 105 | Ornithine | Carboxylic acids and derivatives | Plasma | UPLC-Q-TOF/MS | - | - | PM | (52) |
|  |  |  | Serum | LC-MS & GC-MS | ↓ | - | T2DM | (9) |
|  |  |  | Plasma | LC-MS | - | - | T2DM | (69) |
|  |  |  | Plasma | HPLC-QTrap-MS/MS | ↑/↑ | √ | T2DN | (14) |
|  |  |  | Blood | HPLC-MRM | ↓ | √ | T2DN | (37) |
| 106 | Phosphatidylcholine | Organic dithiophosphoric acids and derivatives | Plasma | UPLC-Q-TOF/MS | - | - | PM | (52) |
| 107 | Acetylcarnitine C2 | Fatty acyls | Plasma | LC-MS | ↑ | - | PM | (4) |
|  |  |  | Plasma | LC-MS/MS | ↓ | √ | T2DK | (12) |
| 108 | Hexose | Organic Oxygen compounds | Plasma | LC-MS | ↑ | - | PM | (4) |
|  |  |  | Urine/Plasma | HPLC-FIA-MA/MS | ↑ | √ | T2DN | (43) |
| 109 | 1,5-Anhydrosorbitol | Organic Oxygen compounds | Plasma | GC-SIM-MS | ↓ | - | PM | (45) |
| 110 | Glucosamine | Organic Oxygen compounds | Plasma | GC-SIM-MS | ↑ | - | PM | (45) |
|  |  |  | Blood | LC-MS | - | - | T2DM | (46) |
| 111 | Mannosamine | Organic Oxygen compounds | Plasma | GC-SIM-MS | ↑ | - | PM | (45) |
| 112 | Mannose | Organic Oxygen compounds | Plasma | GC-SIM-MS | ↑ | - | PM | (45) |
|  |  |  | Plasma | LC-MS | ↑ | - | T2DM | (33) |
|  |  |  | Serum | NMR | ↑ | - | PM | (6) |
|  |  |  | Blood | LC-MS | - | - | T2DM | (46) |
|  |  |  | Plasma | GC×GC-MS | ↑ | - | T2DM | (46) |
|  |  |  | Plasma | GC-MS/MS | - | - | T2DM | (55) |
|  |  |  | Plasma | LC-MS & GC-MS | - | - | T2DM | (11) |
|  |  |  | Urine | GC-MS | ↑ | - | T2DM | (31) |
|  |  |  | Plasma | UHPLC-MS/MS | - | √ | T2DM | (48) |
|  |  |  | Plasma | GC-MS | ↑ | - | T2DR | (70) |
| 113 | 2-Hydroxybutyrate | Hydroxy acids and derivatives | Plasma | GC-SIM-MS | ↑ | - | PM | (45) |
|  |  |  | Plasma | LC-MS | ↑ | - | PM | (33) |
|  |  |  | Serum | GC-MS | ↑ | - | T2DM | (7) |
|  |  |  | Blood | LC-MS | - | - | T2DM | (46) |
|  |  |  | Serum | LC-MS | - | - | T2DM | (61) |
| 114 | 3-Hydroxybutyrate | Hydroxy acids and derivatives | Plasma | GC-SIM-MS | ↑ | - | PM | (45) |
|  |  |  | Plasma | UHPLC-MS/MS | - | √ | T2DM | (48) |
| 115 | Lactate | Organic acid | Plasma | GC-SIM-MS | ↑ | - | PM | (45) |
|  |  |  | Plasma | LC-MS | ↑ | - | PM | (33) |
|  |  |  | Serum | 1H-NMR | ↓ | - | T2DM | (35) |
|  |  |  | Plasma | NMR | ↓ | - | T2DM | (27) |
|  |  |  | Serum | LC-MS | - | - | T2DM | (61) |
| 116 | Glyoxylate | NA | Plasma | GC-SIM-MS | ↑ | - | PM | (45) |
|  |  |  | Blood | LC-MS | - | - | T2DM | (46) |
| 117 | Pyruvic acid | Keto acids and derivatives | Urine | GC-MS | - | - | PM | (44) |
|  |  |  | Urine | UPLC-TOF/MS | ↓ | √ | T2DM | (32) |
| 118 | Ribose | Organic Oxygen compounds | Urine | GC-MS | - | - | PM | (44) |
|  |  |  | Serum | GC-TOF/MS | ↓ | - | T2DM | (71) |
|  |  |  | Plasma | GC-MS | ↑ | - | T2DR | (70) |
|  |  |  | Urine | GC-MS | - | - | T2DN | (72) |
| 119 | Citric acid | Carboxylic acids and derivatives | Urine | GC-MS | - | - | PM | (44) |
|  |  |  | Urine | GC-MS | ↑ | - | T2DM | (73) |
|  |  |  | Serum | GC-TOF/MS | ↑ | - | T2DM | (71) |
|  |  |  | Urine | GC-MS | ↓ | - | T2DM | (59) |
| 120 | Ribulose | Organic Oxygen compounds | Urine | GC-MS | - | - | PM | (44) |
| 121 | Cysteine | Carboxylic acids and derivatives | Urine | GC-MS | - | - | PM | (44) |
|  |  |  | Plasma | HPLC-QTrap-MS/MS | ↑ | √ | T2DN | (14) |
| 122 | meso-Erythritol | Organic Oxygen compounds | Urine | GC-MS | - | - | PM | (44) |
|  |  |  | Plasma | LC-MS | - | - | PM | (33) |
| 123 | Nicotinamide | Pyridines and derivatives | Urine | GC-MS | - | - | PM | (44) |
| 124 | Tryptophan | Indoles and derivatives | Urine | GC-MS | - | - | PM | (44) |
|  |  |  | Plasma | UPLC-Q-TOF/MS | - | - | PM | (17) |
|  |  |  | Urine | UPLC-Q-TOF/MS | ↓ | - | T2DM | (54) |
|  |  |  | Serum | GC-MS | ↓ | - | T2DM | (7) |
|  |  |  | Urine | HPLC-Q-TOF/MS | ↓ | √ | T2DM | (74) |
|  |  |  | Plasma | HPLC-QTrap-MS/MS | ↑/↑ | √ | T2DN | (14) |
|  |  |  | Urine | UPLC-Q-TOF/MS | ↓ | - | T2DN | (64) |
|  |  |  | Plasma | GC-MS | - | - | T2DN | (67) |
| 125 | Diacylglycerol | Glycerolipids | Plasma | ICR-FT/MS | - | - | PM | (75) |
| 126 | Sphingomyelin | NA | Plasma | ICR-FT/MS | - | - | PM | (75) |
| 128 | Urobilinogen | Tetrapyrroles and derivatives | Plasma | ICR-FT/MS | - | - | PM | (75) |
| 129 | Threonine | Carboxylic acids and derivatives | Plasma | UPLC-Q-TOF/MS | ↓ | - | PM | (17) |
|  |  |  | Serum | GC-MS | ↓ | - | T2DM | (7) |
|  |  |  | Serum | 1H-NMR | ↓ | - | T2DM | (35) |
|  |  |  | Serum | LC-MS & GC-MS | ↑ | - | T2DM | (9) |
|  |  |  | Urine | GC-MS | ↑ | - | T2DM | (31) |
|  |  |  | Plasma | UHPLC-MS/MS | - | √ | T2DM | (48) |
|  |  |  | Serum | GC-MS | ↑ | - | T2DPN | (13) |
|  |  |  | Aqueous humor | NMR | ↑ | - | T2DR | (42) |
|  |  |  | Plasma | HPLC-QTrap-MS/MS | ↑/↑ | √ | T2DN | (14) |
| 130 | Arginine | Carboxylic acids and derivatives | Plasma | UPLC-Q-TOF/MS | ↑ | - | PM | (17) |
|  |  |  | Serum | NMR | ↓ | - | T2DM | (34) |
|  |  |  | Plasma | LC-MS/MS | - | √ | T2DM | (10) |
|  |  |  | Plasma | LC-MS | - | - | T2DM | (69) |
|  |  |  | Plasma | HPLC-QTrap-MS/MS | ↑/↓ | √ | T2DN | (14) |
| 131 | Glutamine | Carboxylic acids and derivatives | Plasma | UPLC-Q-TOF/MS | ↑ | - | PM | (17) |
|  |  |  | Plasma/Urine | NMR | ↓ | - | T2DM | (28) |
|  |  |  | Plasma | LC-MS/MS | ↓ | √ | T2DK | (12) |
|  |  |  | Serum | 1H-NMR | ↓ | - | T2DM | (35) |
|  |  |  | Plasma | UPLC-Q-TOF/MS | ↓ | - | T2DM | (29) |
|  |  |  | Aqueous humor | NMR | ↑ | - | T2DR | (42) |
|  |  |  | Serum | LC-MS & GC-MS | ↓ | - | T2DN | (23) |
|  |  |  | Plasma/Urine | HPLC-FIA-MS/MS | ↑ | √ | T2DN | (43) |
| 132 | Proline | Carboxylic acids and derivatives | Plasma | UPLC-Q-TOF/MS | ↑ | - | PM | (17) |
|  |  |  | Plasma | UPLC-Q-TOF/MS | - | - | PM | (5) |
|  |  |  | Plasma | - | - | - | PM | (33) |
|  |  |  | Serum | LC-MS & GC-MS | ↓ | - | T2DM | (56) |
|  |  |  | Plasma | HPLC-QTrap-MS/MS | ↑/↑ | √ | T2DR | (14) |
| 133 | Tyrosine | Carboxylic acids and derivatives | Plasma | UPLC-Q-TOF/MS | ↑ | - | PM | (17) |
|  |  |  | Serum | LC-Q-TOF/MS | - | - | T2DM | (56) |
|  |  |  | Urine | UPLC-Q-TOF/MS | ↑ | - | T2DM | (54) |
|  |  |  | Serum | LC-Q-TOF/MS | - | - | T2DM | (56) |
|  |  |  | Serum | LC-MS | - | - | T2DM | (61) |
|  |  |  | Urine | UPLC-Q-TOF/MS | ↓ | - | T2DM | (64) |
|  |  |  | Plasma | GC-MS | - | - | T2DN | (67) |
|  |  |  | Urine/Plasma | HPLC-FIA-MS/MS | ↑ | √ | T2DN | (43) |
| 134 | Glutamate | Carboxylic acids and derivatives | Plasma | UPLC-Q-TOF/MS | ↑ | - | PM | (17) |
|  |  |  | Plasma | UPLC-Q-TOF/MS | ↑ | - | T2DM | (5) |
| 135 | N-acetylglycine | Carboxylic acids and derivatives | Plasma | - | ↓ | - | PM | (33) |
|  |  |  | Serum | UPLC-MS/MS | - | - | T2DM | (76) |
| 136 | Citrulline | Carboxylic acids and derivatives | Plasma | - | ↓ | - | PM | (33) |
|  |  |  | Plasma | LC-MS/MS | - | √ | T2DM | (10) |
|  |  |  | Plasma | LC-MS | - | - | T2DM | (69) |
|  |  |  | Plasma | HPLC-QTrap-MS/MS | ↑/↑ | √ | T2DN | (14) |
|  |  |  | Blood | HPLC-MRM | ↓ | √ | T2DN | (37) |
| 137 | Dimethylarginine | Carboxylic acids and derivatives | Plasma | - | ↓ | - | PM | (33) |
| 138 | 3-Methyl-2-oxobutyrate | Keto acids and derivatives | Plasma | - | ↑ | - | PM | (33) |
| 139 | 3-Methyl-2-oxovalerate | Fatty acyls | Plasma | - | ↑ | - | PM | (33) |
| 140 | 4-Methyl-2-oxopentanoate | Keto acids and derivatives | Plasma | - | ↑ | - | PM | (33) |
| 141 | Fructose | Organic Oxygen compounds | Plasma | - | ↑ | - | PM | (33) |
|  |  |  | Plasma | GC×GC-MS | ↑ | - | T2DM | (47) |
|  |  |  | Plasma | LC-MS & GC-MS | ↑ | - | T2DM | (11) |
|  |  |  | Urine | GC-MS | ↑ | - | T2DM | (31) |
|  |  |  | Urine | GC-MS | - | - | T2DN | (72) |
| 142 | 1,5-Anhydroglucitol | Organic Oxygen compounds | Plasma | - | ↓ | - | PM | (33) |
|  |  |  | Saliva | - | ↓ | - | T2DM | (77) |
|  |  |  | Plasma | GC×GC-MS | ↓ | - | T2DM | (47) |
|  |  |  | Plasma | LC-MS & GC-MS | - | - | T2DM | (11) |
|  |  |  | Plasma | UHPLC-MS/MS | - | √ | T2DM | (48) |
|  |  |  | Urine | GC-TOF/MS | ↑ | - | T2DN | (15) |
| 143 | Arabinose | Organic Oxygen compounds | Plasma | LC-MS | ↑ | - | PM | (33) |
| 144 | Octanoylcarnitine | Fatty acyls | Plasma | LC-MS | ↓ | - | PM | (33) |
| 145 | 15-Methylpalmitate | Fatty acyls | Plasma | LC-MS | ↓ | - | PM | (33) |
| 146 | 10-Heptadecenoate | Fatty acyls | Plasma | LC-MS | ↓ | - | PM | (33) |
| 147 | Adrenate (22:4n6) | Fatty acyls | Plasma | LC-MS | ↑ | - | PM | (33) |
| 148 | Arachidonate (20:4n6) | Fatty acyls | Plasma | LC-MS | ↑ | - | PM | (33) |
| 149 | Myristate (14:0) | Fatty acyls | Plasma | LC-MS | ↓ | - | PM | (33) |
| 150 | Myristoleate (14:1n5) | Fatty acyls | Plasma | LC-MS | ↓ | - | PM | (33) |
| 151 | Palmitoleate (16:1n7) | Fatty acyls | Plasma | LC-MS | ↓ | - | PM | (33) |
| 152 | Pentadecanoate (15:0) | Fatty acyls | Plasma | LC-MS | ↓ | - | PM | (33) |
| 153 | 5-Dodecenoate (12:1n7) | Fatty acyls | Plasma | LC-MS | ↓ | - | PM | (33) |
| 154 | Heptanoate (7:0) | Fatty acyls | Plasma | LC-MS | ↓ | - | PM | (33) |
|  |  |  | Plasma | UHPLC-MS/MS | - | √ | T2DM | (48) |
| 155 | Pelargonate (9:0) | Fatty acyls | Plasma | - | ↓ | - | PM | (33) |
|  |  |  | Plasma | UHPLC-MS/MS | - | √ | T2DM | (48) |
| 156 | Palmitoyl sphingomyelin | Sphingolipids | Plasma | LC-MS | ↓ | - | PM | (33) |
| 157 | Cholesterol | Steroids and steroid derivatives | Plasma | LC-MS | ↓ | - | PM | (33) |
| 158 | Urate | Imidazopyrimidines | Plasma | LC-MS | ↑ | - | PM | (33) |
| 159 | Glycerol | Organic Oxygen compounds | Serum | NMR | ↑ | - | PM | (6) |
|  |  |  | Serum | GC-MS | ↑ | - | T2DM | (7) |
|  |  |  | Serum | LC-MS & GC-MS | ↓ | - | T2DM | (9) |
|  |  |  | Serum | LC-MS | - | - | T2DM | (61) |
| 160 | Acetoacetate | Organic acid | Serum | NMR | ↑ | - | PM | (6) |
|  |  |  | Serum | 1H-NMR | ↓ | - | T2DM | (35) |
| 161 | Docosahexaenoic acid | Fatty acyls | Serum | UPLC-Q-TOF/MS | ↑ | - | PM | (65) |
| 162 | Sorbitan oleate | Fatty acyls | Serum | UPLC-Q-TOF/MS | ↑ | - | PM | (65) |
| 163 | Methyltestosterone | Steroids and steroid derivatives | Serum | UPLC-Q-TOF/MS | ↑ | - | PM | (65) |
| 164 | Docosanamide | Fatty acyls | Serum | UPLC-Q-TOF/MS | ↑ | - | PM | (65) |
| 165 | Cholesteryl acetate | Keto acids and derivatives | Serum | UPLC-Q-TOF/MS | ↓ | - | PM | (65) |
| 166 | Oleic acid(C18:2) | Fatty acyls | Serum | GC-MS | ↑ | - | T2DM | (78) |
| 167 | Linoleic acid(C18:3) | Fatty acyls | Serum | GC-MS | ↑ | - | T2DM | (78) |
| 168 | Linolenic acid(C18:1) | Fatty acyls | Serum | GC-MS | ↑ | - | T2DM | (78) |
| 169 | Glutaric acid | Carboxylic acids and derivatives | Urine | UPLC-Q-TOF/MS | ↑ | - | T2DM | (54) |
|  |  |  | Serum | LC-MS/MS | ↑ | - | T2DM | (60) |
| 170 | Nicotinuric acid | Carboxylic acids and derivatives | Urine | UPLC-Q-TOF/MS | ↑ | - | T2DM | (54) |
| 171 | Paramethasone acetate |  | Urine | UPLC-Q-TOF/MS | ↑ | - | T2DM | (54) |
| 172 | Dopamine | Phenols | Urine | UPLC-Q-TOF/MS | ↓ | - | T2DM | (54) |
| 173 | Aspartic acid | Carboxylic acids and derivatives | Urine | UPLC-Q-TOF/MS | ↑ | - | T2DM | (54) |
|  |  |  | Plasma | GC-MS | - | - | T2DM | (79) |
| 174 | Urea | Organic carbonic acids and derivatives | Urine | GC-MS | - | √ | T2DM | (80) |
|  |  |  | Serum | LC-MS & GC-MS | ↑ | - | T2DM | (9) |
|  |  |  | Serum | GC-MS | ↓ | - | T2DPN | (13) |
|  |  |  | Plasma | GC-MS | ↑ | - | T2DR | (70) |
| 175 | Threitol | Organic Oxygen compounds | Urine | GC-MS | - | √ | T2DM | (80) |
|  |  |  | Urine | GC-MS | - | - | T2DN | (49) |
| 176 | Sorbitol | Organic Oxygen compounds | Urine | GC-MS | - | √ | T2DM | (80) |
|  |  |  | Plasma | GC-MS/MS | - | - | T2DM | (55) |
| 177 | Inositol | Organic Oxygen compounds | Urine | GC-MS | - | √ | T2DM | (80) |
| 178 | Arabinitol | Organic Oxygen compounds | Urine | GC-MS | - | √ | T2DM | (80) |
|  |  |  | Serum | GC-MS | ↓ | - | T2DPN | (13) |
| 179 | Trihydroxybutyric acid | Organic Oxygen compounds | Urine | GC-MS | - | √ | T2DM | (80) |
| 180 | 2-Ketoisovalerate | Keto acids and derivatives | Serum | GC-MS | ↑ | - | T2DM | (7) |
| 181 | 2-Ketoisocaproate | Keto acids and derivatives | Serum | GC-MS | ↑ | - | T2DM | (7) |
| 182 | Asparagine | Carboxylic acids and derivatives | Serum | GC-MS | ↓ | - | T2DM | (7) |
|  |  |  | Serum | UPLC-Q-Exactive | - | - | T2DM | (36) |
|  |  |  | Aqueous humor | NMR | ↑ | - | T2DR | (42) |
|  |  |  | Plasma | HPLC-QTrap-MS/MS | ↑/↑ | √ | T2DN | (14) |
| 183 | 2-Ketoglutarate | Keto acids and derivatives | Serum | GC-MS | ↓ | - | T2DM | (7) |
| 184 | Galactose | Organic Oxygen compounds | Serum | GC-MS | ↓ | - | T2DM | (7) |
|  |  |  | Plasma | GC-MS/MS | - | - | T2DM | (55) |
|  |  |  | Serum | LC-MS & GC-MS | ↑ | - | T2DM | (9) |
|  |  |  | Plasma | GC-MS | ↑ | - | T2DR | (70) |
| 185 | Canine uric acid | Imidazopyrimidines | Urine | HPLC-Q-TOF/MS | ↓ | √ | T2DM | (74) |
| 186 | Procurinogen canis | NA | Urine | HPLC-Q-TOF/MS | ↑ | √ | T2DM | (74) |
| 189 | Glycocholate | Steroids and steroid derivatives | Serum | UPLC-MS/MS | - | - | T2DM | (76) |
| 190 | Taurochenodeoxycholate | Steroids and steroid derivatives | Serum | UPLC-MS/MS | - | - | T2DM | (76) |
| 191 | Ursodeoxycholate | Steroids and steroid derivatives | Serum | UPLC-MS/MS | - | - | T2DM | (76) |
| 192 | Glycodeoxycholate | Steroids and steroid derivatives | Serum | UPLC-MS/MS | - | - | T2DM | (76) |
| 193 | Glycocholenate sulfate | NA | Serum | UPLC-MS/MS | - | - | T2DM | (76) |
| 194 | Taurocholenate sulfate | Salts | Serum | UPLC-MS/MS | - | - | T2DM | (76) |
| 195 | Glycoursodeoxycholate | Steroids and steroid derivatives | Serum | UPLC-MS/MS | - | - | T2DM | (76) |
| 196 | Methyl indole-3-acetate | Indoles and derivatives | Serum | UPLC-MS/MS | - | - | T2DM | (76) |
| 197 | Indolepropionate | Indoles and derivatives | Serum | UPLC-MS/MS | - | - | T2DM | (76) |
| 198 | Phenyllactate | Phenylpropanoic acids | Serum | UPLC-MS/MS | - | - | T2DM | (76) |
| 199 | Xanthurenate | Quinolines and derivatives | Serum | UPLC-MS/MS | - | - | T2DM | (76) |
| 200 | 3-(4-Hydroxyphenyl)lactate | Phenylpropanoic acids | Serum | UPLC-MS/MS | - | - | T2DM | (76) |
|  |  |  | Serum | UPLC-Q-Exactive | - | - | T2DM | (36) |
| 201 | 4-Hydroxyphenylacetate | Phenol esters | Serum | UPLC-MS/MS | - | - | T2DM | (76) |
| 202 | 3-Phenylpropionate (hydrocinnamate) | Phenylpropanoic acids | Serum | UPLC-MS/MS | - | - | T2DM | (76) |
| 203 | Phenylacetate | Benzene and substituted derivatives | Serum | UPLC-MS/MS | - | - | T2DM | (76) |
| 204 | Phenol sulfate | Organic sulfuric acids and derivatives | Serum | UPLC-MS/MS | - | - | T2DM | (76) |
| 205 | Indolelactate | Indoles and derivatives | Serum | UPLC-MS/MS | - | - | T2DM | (76) |
| 206 | N-acetyltryptophan | Carboxylic acids and derivatives | Serum | UPLC-MS/MS | - | - | T2DM | (76) |
| 207 | Indoleacetylglutamine | Carboxylic acids and derivatives | Serum | UPLC-MS/MS | - | - | T2DM | (76) |
| 208 | 3-Aminoisobutyrate | Carboxylic acids and derivatives | Serum | UPLC-MS/MS | - | - | T2DM | (76) |
| 209 | Imidazole propionate | Azoles | Serum | UPLC-MS/MS | - | - | T2DM | (76) |
| 210 | Hippurate | Carboxylic acids and derivatives | Serum | UPLC-MS/MS | - | - | T2DM | (76) |
| 211 | 2-Hydroxyhippurate (salicylurate) | Benzenoids | Serum | UPLC-MS/MS | - | - | T2DM | (76) |
| 212 | Lactate | Organic acid | Serum | UPLC-MS/MS | - | - | T2DM | (76) |
|  |  |  | Plasma/Urine | NMR | - | - | T2DM | (28) |
|  |  |  | Aqueous humor | NMR | ↓ | - | T2DR | (42) |
| 213 | Isovalerate (i5:0) | Salts | Serum | UPLC-MS/MS | - | - | T2DM | (76) |
| 214 | 2-Hydroxybutyrate/2-Hydroxyisobutyrate | Hydroxy acids and derivatives | Serum | UPLC-MS/MS | - | - | T2DM | (76) |
| 215 | 3-Hydroxyisobutyrate | Hydroxy acids and derivatives | Serum | UPLC-MS/MS | - | - | T2DM | (76) |
|  |  |  | Plasma | LC-MS & GC-MS | - | - | T2DM | (11) |
| 216 | Homovanillate | Phenols | Serum | UPLC-MS/MS | - | - | T2DM | (76) |
| 217 | Serotonin | Indoles and derivatives | Serum | UPLC-MS/MS | - | - | T2DM | (76) |
|  |  |  | Plasma | UPLC-Q-TOF/MS | ↑ | √ | T2DM | (32) |
| 218 | Spermidine | Organonitrogen compounds | Serum | UPLC-MS/MS | - | - | T2DM | (76) |
| 219 | N-acetylputrescine | Carboximidic acids and derivatives | Serum | UPLC-MS/MS | - | - | T2DM | (76) |
| 220 | 1-Linolenoyl-GPC (18:3) | Glycerophospholipids | Serum | UPLC-MS/MS | - | - | T2DM | (76) |
| 221 | 1-Linoleoyl-GPC (18:2) | Glycerophospholipids | Serum | UPLC-MS/MS | - | - | T2DM | (76) |
| 222 | 1-Oleoyl-GPC (18:1) | Glycerophospholipids | Serum | UPLC-MS/MS | - | - | T2DM | (76) |
| 223 | 1-Palmitoyl-GPC (16:0) | Glycerophospholipids | Serum | UPLC-MS/MS | - | - | T2DM | (76) |
| 224 | 1-Lignoceroyl-GPC (24:0) | Glycerophospholipids | Serum | UPLC-MS/MS | - | - | T2DM | (76) |
| 225 | 1-Stearoyl-GPC (18:0) | Glycerophospholipids | Serum | UPLC-MS/MS | - | - | T2DM | (76) |
| 226 | 1-Arachidonoyl-GPE (20:4n6) | Glycerophospholipids | Serum | UPLC-MS/MS | - | - | T2DM | (76) |
| 227 | 1-Linoleoyl-GPE (18:2) | Glycerophospholipids | Serum | UPLC-MS/MS | - | - | T2DM | (76) |
| 228 | 1-Palmitoyl-GPE (16:0) | Glycerophospholipids | Serum | UPLC-MS/MS | - | - | T2DM | (76) |
| 229 | Cholate | Steroids and steroid derivatives | Serum | UPLC-MS/MS | - | - | T2DM | (76) |
| 230 | Chenodeoxycholate | Steroids and steroid derivatives | Serum | UPLC-MS/MS | - | - | T2DM | (76) |
| 231 | Glycochenodeoxycholate | Steroids and steroid derivatives | Serum | UPLC-MS/MS | - | - | T2DM | (76) |
| 232 | Kynurenine | Organic Oxygen compounds | Serum | UPLC-MS/MS | - | - | T2DM | (76) |
| 233 | Kynurenate | Quinolines and derivatives | Serum | UPLC-MS/MS | - | - | T2DM | (76) |
| 234 | 1-Dihomo-linolenylglycerol (20:3) | Glycerolipids | Serum | UPLC-MS/MS | - | - | T2DM | (76) |
| 235 | 1-Myristoylglycerol (14:0) |  | Serum | UPLC-MS/MS | - | - | T2DM | (76) |
| 236 | 1-Oleoylglycerol (18:1) | Glycerolipids | Serum | UPLC-MS/MS | - | - | T2DM | (76) |
| 237 | 1-Palmitoylglycerol (16:0) | Glycerolipids | Serum | UPLC-MS/MS | - | - | T2DM | (76) |
| 238 | 2-Oleoylglycerol (18:1) | Glycerolipids | Serum | UPLC-MS/MS | - | - | T2DM | (76) |
| 239 | 1-Palmitoleoylglycerol (16:1) | NA | Serum | UPLC-MS/MS | - | - | T2DM | (76) |
| 240 | 1-Linolenoylglycerol (18:3) | NA | Serum | UPLC-MS/MS | - | - | T2DM | (76) |
| 241 | Methylamine | Organonitrogen compounds | Serum | 1H-NMR | ↓ | - | T2DM | (35) |
| 242 | Trimethylamine | Organonitrogen compounds | Serum | 1H-NMR | ↓ | - | T2DM | (35) |
| 243 | Cholesteryl ester (C18:1) | Steroids and steroid derivatives | Blood | LC-MS | - | - | T2DM | (46) |
| 244 | Glycochenodeoxycholic acid | Steroids and steroid derivatives | Blood | LC-MS | - | - | T2DM | (46) |
| 245 | Taurochenodeoxycholic acid | Steroids and steroid derivatives | Blood | LC-MS | - | - | T2DM | (46) |
| 246 | Lysophosphatidylcholine (C18:2) | Glycerophospholipids | Blood | LC-MS | - | - | T2DM | (46) |
| 247 | Lignoceric acid (C24:0) | Fatty acyls | Blood | LC-MS | - | - | T2DM | (46) |
| 248 | N-acetyl Glycoprotein | Proteins | Plasma | NMR | ↑ | - | T2DM | (27) |
| 249 | Formate | Salts | Plasma | NMR | ↑ | - | T2DM | (27) |
|  |  |  | Plasma/Urine | NMR | ↓ | - | T2DM | (28) |
| 250 | Acetate | Salts | Plasma | NMR | ↑ | - | T2DM | (27) |
| 251 | Alanine | Carboxylic acids and derivatives | Plasma | NMR | ↓ | - | T2DM | (27) |
|  |  |  | Plasma/Urine | NMR | ↓ | - | T2DM | (28) |
|  |  |  | Serum | LC-Q-TOF/MS | - | - | T2DM | (56) |
|  |  |  | Plasma | LC-MS/MS | - | √ | T2DM | (10) |
|  |  |  | Plasma | LC-MS/MS | ↑ | √ | T2DM | (62) |
|  |  |  | Plasma | LC-MS/MS | ↑ | √ | T2DK | (12) |
|  |  |  | Plasma | HPLC-QTrap-MS/MS | ↑/↑ | √ | T2DN | (14) |
| 252 | Glutamine | Carboxylic acids and derivatives | Plasma | NMR | ↓ | - | T2DM | (27) |
| 253 | 3-D-hydroxybutyrate | Hydroxy acids and derivatives | Plasma/Urine | NMR | ↓ | - | T2DM | (28) |
| 254 | Quinovose | Monosaccharide | Plasma | UPLC-Q-TOF/MS | ↓ | - | T2DM | (29) |
| 255 | Dulcitol | Organic Oxygen compounds | Plasma | UPLC-Q-TOF/MS | ↓ | - | T2DM | (29) |
| 256 | Lyxose | Organic Oxygen compounds | Plasma | UPLC-Q-TOF/MS | ↑ | - | T2DM | (29) |
| 257 | Hypoxanthine | Imidazopyrimidines | Plasma | UPLC-Q-TOF/MS | ↓ | - | T2DM | (29) |
| 258 | 1-Oleoyl-L-.alpha.-lysophosphatidic acid | Organic acid | Plasma | UPLC-Q-TOF/MS | ↑ | - | T2DM | (29) |
| 259 | m-Chlorohippuric acid | Benzene and substituted derivatives | Plasma | UPLC-Q-TOF/MS | ↑ | - | T2DM | (29) |
| 260 | L-Gulonic gamma-lactone | Esters and esters derivatives | Plasma | UPLC-Q-TOF/MS | ↑ | - | T2DM | (29) |
| 261 | Taurine | Organic sulfonic acids and derivatives | Plasma | UPLC-Q-TOF/MS | ↑ | - | T2DM | (29) |
|  |  |  | Plasma | LC-MS/MS | - | √ | T2DM | (8) |
|  |  |  | Plasma | UPLC-Q-TOF/MS | ↓ | √ | T2DM | (32) |
|  |  |  | Plasma | GC-MS | ↓ | - | T2DPN | (13) |
| 262 | Myristic acid | Fatty acyls | Plasma | UPLC-Q-TOF/MS | ↑ | - | T2DM | (29) |
| 263 | Lactate | Organic acid | Plasma | UPLC-Q-TOF/MS | ↓ | - | T2DM | (29) |
|  |  |  | Serum | LC-MS/MS | ↑ | √ | T2DM | (60) |
| 264 | 16-Hydroxypalmitic acid | Fatty acyls | Plasma | UPLC-Q-TOF/MS | ↓ | - | T2DM | (29) |
| 265 | Dodecanoic acid | Fatty acyls | Plasma | UPLC-Q-TOF/MS | ↓ | - | T2DM | (29) |
| 266 | 2-Hydroxy-butanoic acid | Organic acid | Plasma | UPLC-Q-TOF/MS | ↑ | - | T2DM | (29) |
| 267 | Linolenic acid | Fatty acyls | Plasma | UPLC-Q-TOF/MS | ↓ | - | T2DM | (29) |
| 268 | Norethindrone Acetate | Estrogens and derivatives | Plasma | UPLC-Q-TOF/MS | ↓ | - | T2DM | (29) |
| 269 | Myristoleic acid | Fatty acyls | Plasma | UPLC-Q-TOF/MS | ↓ | - | T2DM | (29) |
| 270 | cis-9-Palmitoleic acid | Fatty acyls | Plasma | UPLC-Q-TOF/MS | ↓ | - | T2DM | (29) |
| 271 | Capric acid | Fatty acyls | Plasma | UPLC-Q-TOF/MS | ↓ | - | T2DM | (29) |
|  |  |  | Serum | LC-MS/MS | ↑ | - | T2DM | (60) |
| 272 | 2′-Deoxy-D-ribose | Organic Oxygen compounds | Plasma | UPLC-Q-TOF/MS | ↑ | - | T2DM | (29) |
| 273 | Vitamin E | Prenol lipids | Plasma | UPLC-Q-TOF/MS | ↑ | - | T2DM | (29) |
| 274 | Sunitinib | Indoles and derivatives | Plasma | UPLC-Q-TOF/MS | ↓ | - | T2DM | (29) |
| 275 | Cholesterol 3-sulfate | Steroids and steroid derivatives | Plasma | UPLC-Q-TOF/MS | ↑ | - | T2DM | (29) |
| 276 | Ketoisocaproic acid | Keto acids and derivatives | Plasma | UPLC-Q-TOF/MS | ↑ | - | T2DM | (29) |
| 277 | Methylmalonic acid | Carboxylic acids and derivatives | Plasma | UPLC-Q-TOF/MS | ↑ | - | T2DM | (29) |
| 278 | Phenol | Phenols | Plasma | UPLC-Q-TOF/MS | ↑ | - | T2DM | (29) |
| 279 | Deoxycholic acid | Steroids and steroid derivatives | Plasma | UPLC-Q-TOF/MS | ↑ | - | T2DM | (29) |
|  |  |  | Urine | UPLC-Q-TOF/MS | ↑ | - | T2DN | (64) |
| 280 | 1-Stearoyl-sn-glycerol | Glycerophospholipids | Plasma | UPLC-Q-TOF/MS | ↑ | - | T2DM | (29) |
| 281 | 2-Ethoxyethanol | Organic Oxygen compounds | Plasma | UPLC-Q-TOF/MS | ↓ | - | T2DM | (29) |
| 282 | Dioctyl phthalate | Benzene and substituted derivatives | Plasma | UPLC-Q-TOF/MS | ↑ | - | T2DM | (29) |
|  |  |  | Serum | GC-TOF/MS | ↑ | - | T2DM | (71) |
| 283 | 1-Stearoyl-sn-glycerol 3-phosphocholine | Esters and esters derivatives | Plasma | UPLC-Q-TOF/MS | ↓ | - | T2DM | (29) |
| 284 | 1-Oleoyl-sn-glycero-3-phosphocholine | Glycerophospholipids | Plasma | UPLC-Q-TOF/MS | ↓ | - | T2DM | (29) |
| 285 | Acetylcarnitine | Fatty acyls | Plasma | UPLC-Q-TOF/MS | ↓ | - | T2DM | (29) |
| 286 | Pipecolic acid | Carboxylic acids and derivatives | Plasma | UPLC-Q-TOF/MS | ↑ | - | T2DM | (29) |
| 287 | Erucamide | NA | Plasma | UPLC-Q-TOF/MS | ↑ | - | T2DM | (29) |
| 288 | 1-Palmitoylglycerol | Glycerolipids | Plasma | UPLC-Q-TOF/MS | ↑ | - | T2DM | (29) |
| 289 | Pristanic acid | Prenol lipids | Plasma | UPLC-Q-TOF/MS | ↓ | - | T2DM | (29) |
| 290 | D(-)-beta-hydroxy butyric acid | Fatty acyls | Plasma | UPLC-Q-TOF/MS | ↑ | - | T2DM | (29) |
| 291 | 1-Stearoyl-rac-glycerol | Glycerolipids | Plasma | UPLC-Q-TOF/MS | ↑ | - | T2DM | (29) |
| 292 | Palmitoyl ethanolamide | Carboximidic acids and derivatives | Plasma | UPLC-Q-TOF/MS | ↓ | - | T2DM | (29) |
| 293 | L-Carnitine | Organonitrogen compounds | Plasma | UPLC-Q-TOF/MS | ↓ | - | T2DM | (29) |
|  |  |  | Serum | LC-MS & GC-MS | ↓ | - | T2DN | (23) |
|  |  |  | Blood | HPLC-MRM | ↑ | √ | T2DN | (37) |
|  |  |  | Urine | UPLC-Q-TOF/MS | ↓ | - | T2DN | (64) |
| 294 | Betaine | Carboxylic acids and derivatives | Plasma | UPLC-Q-TOF/MS | ↑ | - | T2DM | (29) |
| 295 | Maltose | Organic Oxygen compounds | Plasma | GC×GC-MS | ↑ | - | T2DM | (47) |
|  |  |  | Plasma | GC-MS | ↑ | - | T2DR | (70) |
| 296 | Trehalose | Organic Oxygen compounds | Plasma | GC×GC-MS | ↑ | - | T2DM | (47) |
|  |  |  | Serum | UPLC-Q-Exactive | - | - | T2DM | (36) |
| 297 | Sedoheptulose | Organic Oxygen compounds | Plasma | GC×GC-MS | ↓ | - | T2DM | (47) |
| 298 | 12-Dilinoleoyl-Gpc (18:2/18:2) | NA | Serum | UPLC-QTRAP | ↓ | √ | T2DM | (41) |
| 299 | 1-Methylhistamine | Organonitrogen compounds | Serum | UPLC-QTRAP | ↑ | √ | T2DM | (41) |
| 300 | 1-Ribosyl-Imidazoleacetate | Esters and esters derivatives | Serum | UPLC-QTRAP | ↓ | √ | T2DM | (41) |
| 301 | 3-Amino-2-Piperidone | Carboxylic acids and derivatives | Serum | UPLC-QTRAP | ↓ | - | T2DM | (41) |
| 302 | 5-Methylthioadenosine | 5'-deoxyribonucleosides | Serum | UPLC-QTRAP | ↑ | - | T2DM | (41) |
| 303 | Alpha-hydroxyisovalerate | Fatty acyls | Serum | UPLC-QTRAP | ↓ | - | T2DM | (41) |
| 304 | Arachidonoylcholine | Organonitrogen compounds | Serum | UPLC-QTRAP | ↑ | - | T2DM | (41) |
| 305 | Cortisol | Steroids and steroid derivatives | Serum | UPLC-QTRAP | ↓ | - | T2DM | (41) |
| 306 | Docosatrienoate (22:3N3) | Fatty acyls | Serum | UPLC-QTRAP | ↓ | - | T2DM | (41) |
| 307 | Formiminoglutamate | Carboxylic acids and derivatives | Serum | UPLC-QTRAP | ↑ | - | T2DM | (41) |
| 308 | Gamma-tocopherol | Prenol lipids | Serum | UPLC-QTRAP | ↑ | - | T2DM | (41) |
| 309 | Glycerol 3-phosphate | Glycerophospholipids | Serum | UPLC-QTRAP | ↓ | - | T2DM | (41) |
| 310 | Glycerophosphoethanolamine | Glycerophospholipids | Serum | UPLC-QTRAP | ↑ | - | T2DM | (41) |
| 311 | Glycerophosphorylcholine | Glycerophospholipids | Serum | UPLC-QTRAP | ↑ | - | T2DM | (41) |
| 312 | N-Formylanthranilic Acid | Benzene and substituted derivatives | Serum | UPLC-QTRAP | ↑ | - | T2DM | (41) |
| 313 | N-Stearoyl-Sphinganine (D18:0/18:0) | Sphingolipids | Serum | UPLC-QTRAP | ↓ | - | T2DM | (41) |
| 314 | Pipecolate | Carboxylic acids and derivatives | Serum | UPLC-QTRAP | ↓ | - | T2DM | (41) |
| 315 | Ribitol | Organic Oxygen compounds | Serum | UPLC-QTRAP | ↓ | - | T2DM | (41) |
| 316 | LPC acyl C28:1 | Glycerophospholipids | Serum | LC-MS/MS | ↓ | √ | T2DM | (30) |
| 317 | PC diacyl C28:1 | Glycerophospholipids | Serum | LC-MS/MS | ↓ | √ | T2DM | (30) |
| 318 | PC acyl-alkyl C40:1 | Glycerophospholipids | Serum | LC-MS/MS | ↓ | √ | T2DM | (30) |
| 319 | PC diacyl C42:4 | Glycerophospholipids | Serum | LC-MS/MS | ↓ | √ | T2DM | (30) |
| 320 | PC acyl-alkyl C38:1 | Glycerophospholipids | Serum | LC-MS/MS | ↑ | √ | T2DM | (30) |
| 321 | PC acyl-alkyl C42:5 | Glycerophospholipids | Serum | LC-MS/MS | ↓ | √ | T2DM | (30) |
| 322 | PC acyl-alkyl C40:5 | Glycerophospholipids | Serum | LC-MS/MS | ↓ | √ | T2DM | (30) |
| 323 | PC acyl-alkyl C42:4 | Glycerophospholipids | Serum | LC-MS/MS | ↓ | √ | T2DM | (30) |
| 324 | PC acyl-alkyl C36:1 | Glycerophospholipids | Serum | LC-MS/MS | ↓ | √ | T2DM | (30) |
| 325 | PC acyl-alkyl C40:3 | Glycerophospholipids | Serum | LC-MS/MS | ↓ | √ | T2DM | (30) |
| 326 | PC diacyl C40:3 | Glycerophospholipids | Serum | LC-MS/MS | ↓ | √ | T2DM | (30) |
| 327 | PC acyl-alkyl C38:0 | Glycerophospholipids | Serum | LC-MS/MS | ↓ | √ | T2DM | (30) |
| 328 | PC acyl-alkyl C40:2 | Glycerophospholipids | Serum | LC-MS/MS | ↓ | √ | T2DM | (30) |
| 329 | Caprylic acid | Fatty acyls | Serum | LC-MS/MS | ↑ | √ | T2DM | (60) |
| 330 | Citrate | Fatty acyls | Serum | LC-MS/MS | ↑ | √ | T2DM | (60) |
| 331 | Ethylmalonic acid | Fatty acyls | Serum | LC-MS/MS | ↑ | √ | T2DM | (60) |
| 332 | Glycolic acid | Hydroxy acids and derivatives | Serum | LC-MS/MS | ↑ | √ | T2DM | (60) |
|  |  |  | Serum | GC-TOF/MS | ↑ | - | T2DM | (60) |
|  |  |  | Urine | GC-MS | - | - | T2DN | (72) |
|  |  |  | Urine | GC-MS | ↓ | - | T2DN | (59) |
|  |  |  | Urine | GC-TOF/MS | ↓ | - | T2DN | (15) |
| 333 | β-Hydroxybutyrate | Beta hydroxy acids and derivatives | Serum | LC-MS/MS | ↑ | √ | T2DM | (60) |
| 334 | α-Hydroxybutyrate | Organic acid derived from alpha-ketobutyrate | Serum | LC-MS/MS | ↑ | √ | T2DM | (60) |
| 335 | Orotic acid | Diazines | Serum | LC-MS/MS | ↑ | √ | T2DM | (60) |
|  |  |  | Plasma | HPLC-UV-MS/MS | ↑ | √ | T2DN | (40) |
| 336 | Oxalic acid | Carboxylic acids and derivatives | Serum | LC-MS/MS | ↑ | √ | T2DM | (60) |
|  |  |  | Plasma | GC-MS/MS | - | - | T2DM | (55) |
| 337 | Oxaloacetate | Keto acids and derivatives | Serum | LC-MS/MS | ↑ | √ | T2DM | (60) |
| 338 | Sebacic acid | Fatty acyls | Serum | LC-MS/MS | ↑ | √ | T2DM | (60) |
| 339 | Myoinositol | Organic Oxygen compounds | Serum | GC-MS | ↑ | - | T2DM | (73) |
| 340 | Pseudouridine | Nucleoside and nucleotide analogues | Serum | GC-MS | ↑ | - | T2DM | (73) |
|  |  |  | Plasma | LC-MS & GC-MS | - | - | T2DM | (11) |
|  |  |  | Plasma | UPLC-MS/MS  GC-MS | - | - | T2DN | (81) |
| 341 | p-hydroxyphenylacetic Acid | Phenols | Serum | GC-MS | ↑ | - | T2DM | (73) |
| 342 | Hippuric Acid | Benzene and substituted derivatives | Serum | GC-MS | ↑ | - | T2DM | (73) |
|  |  |  | Serum | LC-MS & GC-MS | ↑ | - | T2DM | (9) |
|  |  |  | Serum | GC-MS | ↑ | - | T2DPN | (13) |
|  |  |  | Blood | HPLC-MS | ↓ | - | T2DN | (64) |
|  |  |  | Urine | GC-TOF/MS | ↑ | - | T2DN | (15) |
| 343 | Hypoxanthine | Imidazopyrimidines | Serum | GC-MS | ↓ | - | T2DM | (73) |
|  |  |  | Plasma | HPLC-UV-MS/MS | ↑ | √ | T2DN | (40) |
|  |  |  | Urine | UPLC-Q-TOF/MS | - | - | T2DN | (38) |
| 344 | Sphingomyelin C24:0 | Sphingolipids | Plasma | LC-MS/MS | - | √ | T2DM | (8) |
| 345 | Diacylglycerol C36:1 | Glycerolipids | Plasma | LC-MS/MS | - | √ | T2DM | (8) |
|  |  |  | Serum | LC-MS & GC-MS | ↓ | - | T2DN | (23) |
| 346 | Triacylglycerols C58:11 | Esters and esters derivatives | Plasma | LC-MS/MS | - | √ | T2DM | (8) |
| 347 | 5-Hydroxyindoleacetic acid | Indoles and derivatives | Plasma | LC-MS/MS | - | √ | T2DM | (8) |
|  |  |  | Urine | UPLC-Q-TOF/MS | ↑ | - | T2DN | (64) |
| 348 | PC C36:4 | Glycerophospholipids | Plasma | LC-MS/MS | - | √ | T2DM | (8) |
| 351 | 3-Methyladipic acid | Fatty acyls | Plasma | LC-MS/MS | - | √ | T2DM | (8) |
| 350 | 2-Aminodipate | Salts | Plasma | LC-MS/MS | - | √ | T2DM | (8) |
| 351 | Isocitrate | Carboxylic acids and derivatives | Plasma | LC-MS/MS | - | √ | T2DM | (8) |
| 352 | Triacylglycerols C52:1 | Glycerolipids | Plasma | LC-MS/MS | - | √ | T2DM | (8) |
| 353 | Triacylglycerols C48:0 | Glycerolipids | Plasma | LC-MS/MS | - | √ | T2DM | (8) |
|  |  |  | Serum | LC-MS | - | - | T2DM | (61) |
| 354 | Triacylglycerols C48:1 | Glycerolipids | Plasma | LC-MS/MS | - | √ | T2DM | (8) |
|  |  |  | Serum | LC-MS | - | - | T2DM | (61) |
| 355 | Triacylglycerols C54:8 | Glycerolipids | Plasma | LC-MS/MS | - | √ | T2DM | (8) |
| 356 | Cholesterol | Steroids and steroid derivatives | Sreum | UHPLC-Q-TOF/MS | ↑ | - | T2DM | (82) |
| 357 | 25-Hydroxycholesterol | Steroids and steroid derivatives | Sreum | UHPLC-Q-TOF/MS | ↑ | - | T2DM | (82) |
| 358 | 3 α ,7 α -Dihydroxy-5 β-cholestane | Steroids and steroid derivatives | Sreum | UHPLC-Q-TOF/MS | ↑ | - | T2DM | (82) |
| 359 | 4α -Methylzymosterol-4-carboxylate | Prenol lipids | Sreum | UHPLC-Q-TOF/MS | ↑ | - | T2DM | (82) |
| 360 | 24,25-Dihydrolanosterol | Prenol lipids | Sreum | UHPLC-Q-TOF/MS | ↓ | - | T2DM | (82) |
| 361 | Gentisic acid | Benzene and substituted derivatives | Sreum | GC-TOF/MS | ↑ | - | T2DM | (71) |
| 362 | Glucosaminic acid | Organic acid | Sreum | GC-TOF/MS | ↑ | - | T2DM | (71) |
| 363 | Succinic acid | Carboxylic acids and derivatives | Sreum | GC-TOF/MS | ↑ | - | T2DM | (71) |
|  |  |  | Urine | GC-MS | ↓ | - | T2DM | (31) |
|  |  |  | Urine | GC-TOF/MS | ↓ | - | T2DN | (15) |
| 364 | 2-Hydroxybutanoic acid | Hydroxy acids and derivatives | Sreum | GC-TOF/MS | ↑ | - | T2DM | (71) |
| 365 | D-Erythro-sphingosine | Organonitrogen compounds | Sreum | GC-TOF/MS | ↑ | - | T2DM | (71) |
| 366 | D-Talose | Monosaccharide | Sreum | GC-TOF/MS | ↓ | - | T2DM | (71) |
| 367 | Β-Mannosylglycerate | Esters and esters derivatives | Sreum | GC-TOF/MS | ↓ | - | T2DM | (71) |
| 368 | Allylmalonic acid | Organic acid | Sreum | GC-TOF/MS | ↓ | - | T2DM | (71) |
| 369 | 3-Hydroxy-L-proline | Carboxylic acids and derivatives | Sreum | GC-TOF/MS | ↓ | - | T2DM | (71) |
| 370 | Conduritol b epoxide | Esters and esters derivatives | Sreum | GC-TOF/MS | ↑ | - | T2DM | (71) |
| 371 | 2-Ketovaleric acid | Keto acids and derivatives | Sreum | GC-TOF/MS | ↑ | - | T2DM | (71) |
| 372 | Glucose-1-phosphate | Organic Oxygen compounds | Sreum | GC-TOF/MS | ↑ | - | T2DM | (71) |
| 373 | Galacticol | Organic Oxygen compounds | Plasma | GC-MS/MS | - | - | T2DM | (55) |
| 374 | Glucaric acid-1,4-lactone |  | Plasma | GC-MS/MS | - | - | T2DM | (55) |
| 375 | 2-Hydroxybutyric acid | Hydroxy acids and derivatives | Plasma | GC-MS/MS | - | - | T2DM | (55) |
|  |  |  | Urine | GC-MS | - | √ | T2DN | (49) |
| 376 | Indolepropionic acid | Indoles and derivatives | Serum | LC-Q-TOF/MS | - | - | T2DM | (56) |
| 377 | PC(18:1/22:6) | Glycerophospholipids | Serum | LC-Q-TOF/MS | - | - | T2DM | (56) |
| 378 | LysoPC(20:1) | Glycerophospholipids | Serum | LC-Q-TOF/MS | - | - | T2DM | (56) |
| 379 | LysoPC(15:1) | Glycerophospholipids | Serum | LC-Q-TOF/MS | - | - | T2DM | (56) |
| 380 | PC(20:4/17:0) | Glycerophospholipids | Serum | LC-Q-TOF/MS | - | - | T2DM | (56) |
| 381 | PC(22:6/17:0) | Glycerophospholipids | Serum | LC-Q-TOF/MS | - | - | T2DM | (56) |
| 382 | PC(15:1/18:2) | Glycerophospholipids | Serum | LC-Q-TOF/MS | - | - | T2DM | (56) |
| 383 | LysoPE(16:0) | Glycerophospholipids | Serum | LC-Q-TOF/MS | - | - | T2DM | (56) |
| 384 | PC(18:2/15:0) | Glycerophospholipids | Serum | LC-Q-TOF/MS | - | - | T2DM | (56) |
| 385 | 2-Aminooctanoic acid | Carboxylic acids and derivatives | Serum | LC-MS & GC/MS | ↓ | - | T2DM | (9) |
| 386 | Aminomalonic acid | Carboxylic acids and derivatives | Serum | LC-MS & GC/MS | ↑ | - | T2DM | (9) |
| 387 | Phosphoserine | Carboxylic acids and derivatives | Serum | LC-MS & GC/MS | ↓ | - | T2DM | (9) |
| 388 | Gluconate | Organic Oxygen compounds | Serum | LC-MS & GC/MS | ↑ | - | T2DM | (9) |
|  |  |  | Serum | LC-MS & GC/MS | - | - | T2DM | (11) |
| 389 | 9-Decenoylcarnitine | Fatty acyls | Serum | LC-MS & GC/MS | ↓ | - | T2DM | (9) |
| 390 | Stearic acid | Fatty acyls | Serum | LC-MS & GC/MS | ↑ | - | T2DM | (9) |
|  |  |  | Urine | GC-TOF/MS | ↑ | - | T2DN | (15) |
| 391 | LysoPE(20:3) | Glycerophospholipids | Serum | LC-MS & GC/MS | ↓ | - | T2DM | (9) |
| 392 | LysoPE(20:3) | Glycerophospholipids | Serum | LC-MS & GC/MS | ↓ | - | T2DM | (9) |
| 393 | LysoPG(12:0) | Glycerophospholipids | Serum | LC-MS & GC/MS | ↑ | - | T2DM | (9) |
| 394 | LysoPI(16:1) | Glycerophospholipids | Serum | LC-MS & GC/MS | ↑ | - | T2DM | (9) |
| 395 | LysoPI(18:1) | Glycerophospholipids | Serum | LC-MS & GC/MS | ↑ | - | T2DM | (9) |
| 396 | LysoPI(18:2) | Glycerophospholipids | Serum | LC-MS & GC/MS | ↑ | - | T2DM | (9) |
| 397 | LysoPI(20:3) | Glycerophospholipids | Serum | LC-MS & GC/MS | ↑ | - | T2DM | (9) |
| 398 | LysoPI(20:4) | Glycerophospholipids | Serum | LC-MS & GC/MS | ↑ | - | T2DM | (9) |
| 399 | LysoPI(22:6) | Glycerophospholipids | Serum | LC-MS & GC/MS | ↑ | - | T2DM | (9) |
| 400 | 1,3-Propanediol | Polyols | Serum | LC-MS & GC/MS | ↑ | - | T2DM | (9) |
| 401 | Acylcarnitine | 5'-deoxyribonucleosides | Plasma | LC-MS/MS | - | √ | T2DM | (83) |
| 402 | Acylcarnitine C3 | NA | Plasma | LC-MS/MS | - | √ | T2DM | (10) |
| 403 | Acylcarnitine C4 | NA | Plasma | LC-MS/MS | - | √ | T2DM | (10) |
| 404 | Acylcarnitine C5 | NA | Plasma | LC-MS/MS | - | √ | T2DM | (10) |
| 405 | Acylcarnitine C6 | NA | Plasma | LC-MS/MS | - | √ | T2DM | (10) |
| 406 | Acylcarnitine C10 | NA | Plasma | LC-MS/MS | - | √ | T2DM | (10) |
| 407 | Acylcarnitine C10:1 | NA | Plasma | LC-MS/MS | - | √ | T2DM | (10) |
| 408 | Methionine | Carboxylic acids and derivatives | Plasma | LC-MS/MS | - | √ | T2DM | (10) |
|  |  |  | Serum | LC-MS | - | - | T2DM | (61) |
|  |  |  | Plasma | LC-MS/MS | ↑ | √ | T2DK | (12) |
|  |  |  | Plasma | HPLC-QTrap-MS/MS | ↑/↑ | √ | T2DN | (14) |
|  |  |  | Blood | HPLC-MRM | ↓ | √ | T2DN | (37) |
| 409 | Propionyl carnitine | Amino acids and derivatives | Plasma | UPLC-Q-TOF/MS | ↑ | - | T2DM | (18) |
| 410 | Octanoyl carnitine | Amino acids and derivatives | Plasma | UPLC-Q-TOF/MS | ↑ | - | T2DM | (18) |
| 411 | Decanoyl carnitine | Amino acids and derivatives | Plasma | UPLC-Q-TOF/MS | ↑ | - | T2DM | (18) |
| 412 | Dodecanoyl carnitine | Amino acids and derivatives | Plasma | UPLC-Q-TOF/MS | ↑ | - | T2DM | (18) |
| 413 | Heptadecanoyl carnitine | Amino acids and derivatives | Plasma | UPLC-Q-TOF/MS | ↑ | - | T2DM | (18) |
| 414 | Linoleyl carnitine | Fatty acyls | Plasma | UPLC-Q-TOF/MS | ↑ | - | T2DM | (18) |
| 415 | Vaccenyl carnitine | - | Plasma | UPLC-Q-TOF/MS | ↑ | - | T2DM | (18) |
| 416 | LysoPC(16:1) | Glycerophospholipids | Plasma | UPLC-Q-TOF/MS | ↑ | - | T2DM | (18) |
| 417 | LysoPE(18:1) | Glycerophospholipids | Plasma | UPLC-Q-TOF/MS | ↓ | - | T2DM | (18) |
| 418 | LysoPE(18:2) | Glycerophospholipids | Plasma | UPLC-Q-TOF/MS | ↑ | - | T2DM | (18) |
| 419 | LysoPE(22:6) | Glycerophospholipids | Plasma | UPLC-Q-TOF/MS | ↑ | - | T2DM | (18) |
| 420 | PC ae C34:3 | Phosphatidylcholine | Plasma | LC-MS/MS | ↓ | √ | T2DM | (62) |
| 421 | PC ae C44:6 | Phosphatidylcholine | Plasma | LC-MS/MS | ↓ | √ | T2DM | (62) |
| 422 | PC ae C42:4 | Phosphatidylcholine | Plasma | LC-MS/MS | ↓ | √ | T2DM | (62) |
| 423 | PC ae C32:2 | Phosphatidylcholine | Plasma | LC-MS/MS | ↓ | √ | T2DM | (62) |
| 424 | PC ae C44:3 | Phosphatidylcholine | Plasma | LC-MS/MS | ↓ | √ | T2DM | (62) |
| 425 | PC ae C44:4 | Phosphatidylcholine | Plasma | LC-MS/MS | ↓ | √ | T2DM | (62) |
| 426 | PC aa C42:0 | Phosphatidylcholine | Plasma | LC-MS/MS | ↓ | √ | T2DM | (62) |
| 427 | PC aa C42:2 | Phosphatidylcholine | Plasma | LC-MS/MS | ↓ | √ | T2DM | (62) |
| 428 | Erythritol | Saccharides and derivatives | Serum | UPLC-Q-Exactive | - | - | T2DM | (36) |
|  |  |  | Plasma | GC-MS | ↑ | - | T2DR | (70) |
|  |  |  | Urine | GC-MS | - | - | T2DN | (72) |
| 429 | PC(O-34:2) | Phosphatidylcholine | Serum | LC-MS | - | - | T2DM | (61) |
| 430 | 2-Oxoglutaric acid | Gamma-keto acids and derivatives | Serum | LC-MS | - | - | T2DM | (61) |
| 431 | Glycine betaine | Small N-trimethylated amino acid | Serum | LC-MS | - | - | T2DM | (61) |
| 432 | Triacylglycerols C50:5 | Esters and esters derivatives | Serum | LC-MS | - | - | T2DM | (61) |
| 433 | Cyclic AMP | Adenine nucleotide containing one phosphate group | Urine | LC-MS/MS | ↓ | √ | T2DM | (66) |
| 434 | 5′-Methylthioadenosine | 5'-deoxy-5'-thionucleosides | Urine | LC-MS/MS | ↑ | √ | T2DM | (66) |
| 435 | Acetylhistidine | Histidine and derivatives | Urine | LC-MS/MS | ↓ | √ | T2DM | (66) |
| 436 | 3-Hydroxypyruvate | Salts | Plasma | LC-MS & GC-MS | - | - | T2DM | (11) |
| 437 | 2-Hydroxybutyrate | Salts | Plasma | LC-MS & GC-MS | - | - | T2DM | (11) |
| 438 | 2-Aminobutyrate | Salts | Plasma | LC-MS & GC-MS | - | - | T2DM | (11) |
| 439 | 2-Ketobutyrate | Esters and esters derivatives | Plasma | LC-MS & GC-MS | - | - | T2DM | (11) |
| 440 | 3-Hydroxypropanoate | Salts | Plasma | LC-MS & GC-MS | - | - | T2DM | (11) |
| 441 | Pyroglutamine | Alpha amino acids and derivatives | Plasma | LC-MS & GC-MS | - | - | T2DM | (11) |
| 442 | 3-Methoxytyrosine | Tyrosine and derivatives | Plasma | LC-MS & GC-MS | - | - | T2DM | (11) |
| 443 | O-Sulfo-L-tyrosine | Phenylalanine and derivatives | Plasma | LC-MS & GC-MS | - | - | T2DM | (11) |
| 444 | γ-Glutamylglutamine | Dipeptide | Plasma | LC-MS & GC-MS | - | - | T2DM | (11) |
| 445 | Acetacetic acid | Organic acid | Urine | GC-MS | ↑ | - | T2DM | (31) |
| 446 | PC(22:2/16:0) | Phosphatidylcholine | Serum | UPLC-MS | - | - | T2DM | (21) |
| 447 | PC(20:2/18:0) | Phosphatidylcholine | Serum | UPLC-MS | - | - | T2DM | (21) |
| 448 | PC(20:1/18:1) | Phosphatidylcholine | Serum | UPLC-MS | - | - | T2DM | (21) |
| 449 | PC(O-20:0/16:0) | Phosphatidylcholine | Serum | UPLC-MS | - | - | T2DM | (21) |
| 450 | PC(O-18:0/18:0) | Phosphatidylcholine | Serum | UPLC-MS | - | - | T2DM | (21) |
| 451 | PC(O-16:0/20:0) | Phosphatidylcholine | Serum | UPLC-MS | - | - | T2DM | (21) |
| 452 | PC(O-14:0/22:0) | Phosphatidylcholine | Serum | UPLC-MS | - | - | T2DM | (21) |
| 453 | GlcCer(d18:0/22:0) | Glycosphingolipid | Serum | UPLC-MS | - | - | T2DM | (21) |
| 454 | Tetrahydroxyhexanoic acid | Medium-chain hydroxy acids and derivatives | Serum | UPLC-MS | - | - | T2DM | (21) |
| 455 | Itaconic acid | Branched fatty acids | Plasma | UPLC-Q-TOF/MS | ↑ | √ | T2DM | (32) |
| 456 | Inosine | Purine nucleosides | Plasma | UPLC-Q-TOF/MS | ↓ | √ | T2DM | (32) |
|  |  |  | Plasma | HPLC-UV-MS/MS | ↑ | √ | T2DN | (40) |
| 457 | 3-Hydroxymethyl-glutaric acid | Organic acid | Plasma | UPLC-Q-TOF/MS | ↓ | √ | T2DM | (32) |
| 458 | PC(18:0/0:0) | Phosphatidylcholine | Plasma | UPLC-Q-TOF/MS | ↓ | √ | T2DM | (32) |
| 459 | Sphingosine-1-phosphate | Phosphosphingolipids | Plasma | UPLC-Q-TOF/MS | ↑ | √ | T2DM | (32) |
| 460 | PE(P-16:0/22:6) | Glycerophospholipids | Plasma | UPLC-Q-TOF/MS | ↓ | √ | T2DM | (32) |
| 461 | PG(18:0/18:1) | Phosphatidylglycerol or glycerophospholipid | Plasma | UPLC-Q-TOF/MS | ↑ | √ | T2DM | (32) |
| 462 | 2-Ketobutyric acid | Organic acid | Plasma | UPLC-Q-TOF/MS | ↓ | √ | T2DM | (32) |
| 463 | 2-Ketoglutaric acid | Organic acid | Plasma | UPLC-Q-TOF/MS | ↓ | √ | T2DM | (32) |
| 464 | 1-Methylhistidine | Histidine and derivatives | Plasma | UPLC-Q-TOF/MS | ↓ | √ | T2DM | (32) |
| 465 | N-Acetyl-D-phenylalanine |  | Plasma | UPLC-Q-TOF/MS | ↑ | √ | T2DM | (32) |
| 466 | Xanthurenic acid | Quinoline carboxylic acids | Plasma | UPLC-Q-TOF/MS | ↓ | √ | T2DM | (32) |
| 467 | Desoxyhexose | Monosaccharide | Plasma | UHPLC-MS/MS | - | √ | T2DM | (48) |
| 468 | Uronic acid | Saccharides and derivatives | Plasma | UHPLC-MS/MS | - | √ | T2DM | (48) |
| 469 | Dihexose(2H) | Saccharides and derivatives | Plasma | UHPLC-MS/MS | - | √ | T2DM | (48) |
| 470 | Caproate (6:0) | Salts | Plasma | UHPLC-MS/MS | - | √ | T2DM | (48) |
| 471 | PC a C20:4 | Phosphatidylcholine | Plasma | UHPLC-MS/MS | - | √ | T2DM | (48) |
| 472 | PC aa (OH, COOH) C28:4 | Phosphatidylcholine | Plasma | UHPLC-MS/MS | - | √ | T2DM | (48) |
| 473 | PC aa C34:4 | Phosphatidylcholine | Plasma | UHPLC-MS/MS | - | √ | T2DM | (48) |
| 474 | Glutamylvaline | Dipeptide | Plasma | UHPLC-MS/MS | - | √ | T2DM | (48) |
| 475 | gamma-glutamylisoleucine | Dipeptide | Plasma | UHPLC-MS/MS | - | √ | T2DM | (48) |
| 476 | Phenylacetylglutamine | Amino acids and derivatives | Plasma | UHPLC-MS/MS | - | √ | T2DM | (48) |
| 477 | 3-indoxyl sulfate | Dietary protein | Plasma | UHPLC-MS/MS | - | √ | T2DM | (48) |
| 478 | Homocitrulline | Saccharides and derivatives | Plasma | UHPLC-MS/MS | - | √ | T2DM | (48) |
| 479 | Isobutyrylcarnitine | Amino acids and derivatives | Plasma | LC-MS/MS | ↑ | √ | T2DM | (12) |
| 480 | 2,3 methylbutyryl-carnitine (C5) | Amino acids and derivatives | Plasma | LC-MS/MS | ↓ | √ | T2DM | (12) |
| 481 | b-Hydroxybutyryl-carnitine (C4-OH) | Amino acids and derivatives | Plasma | LC-MS/MS | ↑ | √ | T2DM | (12) |
| 482 | Methylmalonyl/succinyl-carnitine (C4-DC) | Amino acids and derivatives | Plasma | LC-MS/MS | ↓ | √ | T2DM | (12) |
| 483 | Glutaryl-carnitine (C5-DC) | Amino acids and derivatives | Plasma | LC-MS/MS | ↓ | √ | T2DM | (12) |
| 484 | 3-Hydroxyisovaleryl-carnitine (C5-OH) | Amino acids and derivatives | Plasma | LC-MS/MS | ↓ | √ | T2DM | (12) |
| 485 | Hexuronic acid |  | Serum | GC-MS | ↓ | - | T2DPN | (13) |
| 486 | β-hydroxymyristic acid | Fatty acid | Serum | GC-MS | ↓ | - | T2DPN | (13) |
| 487 | Nicotinic acid | Water-soluble vitamin | Serum | GC-MS | ↓ | - | T2DPN | (13) |
| 488 | Phenylethanol | Alcohols | Serum | GC-MS | ↓ | - | T2DPN | (13) |
| 489 | 3-Urea propionic acid | Isocoumarans | Serum | GC-MS | ↓ | - | T2DPN | (13) |
| 490 | Benzyl alcohol | Alcohols | Serum | GC-MS | ↓ | - | T2DPN | (13) |
| 491 | Octoic acid | Organic acid | Serum | GC-MS | ↓ | - | T2DPN | (13) |
| 492 | Pyroglutamate | Salts | Serum | GC-MS | ↓ | - | T2DPN | (13) |
| 493 | D-xylose | Polysaccharide | Serum | GC-MS | ↓ | - | T2DPN | (13) |
| 494 | Maleimide | Nitrogen mustard compounds | Serum | GC-MS | ↓ | - | T2DPN | (13) |
| 495 | Boric acid | Metalloid oxides | Serum | GC-MS | ↑ | - | T2DPN | (13) |
| 496 | PC[14:1(9Z) /22:2(13Z,16Z)] | Phosphatidylcholine | Serum | UPLC-Q-TOF/MS | ↓ | - | T2DR | (84) |
| 497 | 3-Hydroxysuberic acid | Medium-chain hydroxy acids and derivatives | Serum | UPLC-Q-TOF/MS | ↑ | - | T2DR | (84) |
| 498 | Kynurenic acid | NA | Serum | UPLC-Q-TOF/MS | ↓ | - | T2DR | (84) |
| 499 | PC[14:0/20:2(11Z,14Z)] | Phosphatidylcholine | Serum | UPLC-Q-TOF/MS | ↓ | - | T2DR | (84) |
| 500 | Ritalinic acid | Beta Amino Acids and Derivatives | Serum | UPLC-Q-TOF/MS | ↓ | - | T2DR | (84) |
| 501 | Hydroxycotinine | Pyrrolidinylpyridines | Serum | UPLC-Q-TOF/MS | ↑ | - | T2DR | (84) |
| 502 | 5’- Hydroxytenoxicam | Thienothiazines | Serum | UPLC-Q-TOF/MS | ↓ | - | T2DR | (84) |
| 503 | PC[14: 0/22: 5(4Z,7Z,10Z,13Z,16Z)] | Phosphatidylcholine | Serum | UPLC-Q-TOF/MS | ↓ | - | T2DR | (84) |
| 504 | Dimethylsulfide | Volatile sulfur compound | Serum | UPLC-Q-TOF/MS | ↑ | - | T2DR | (84) |
| 505 | Acetic acid | Carboxylic acids | Serum | UPLC-Q-TOF/MS | ↑ | - | T2DR | (84) |
| 506 | 1,5-Gluconolactone | Lactone or oxidized derivative of glucose | Plasma | GC-MS | ↑ | - | T2DR | (70) |
| 507 | 1,5-Anhydroglucitol | Saccharides and derivatives | Plasma | GC-MS | ↓ | - | T2DR | (70) |
|  |  |  | Plasma | GC-MS | - | - | T2DN | (67) |
| 508 | 2-Deoxyribonic acid | Nucleotide | Plasma | GC-MS | ↑ | - | T2DR | (70) |
| 509 | 3,4-Dihydroxybutyric acid | Organic acid | Plasma | GC-MS | ↑ | - | T2DR | (70) |
| 510 | Gluconic acid | Saccharides and derivatives | Plasma | GC-MS | ↑ | - | T2DR | (70) |
| 511 | Glutathione | Peptides and derivatives | Plasma | LC-ESI-MS/MS | ↑ | - | T2DR | (85) |
| 512 | Glutathione hydropersulfide | Peptides and derivatives | Plasma | LC-ESI-MS/MS | ↓ | - | T2DR | (85) |
| 513 | Glutathione disulfide | Peptides and derivatives | Plasma | LC-ESI-MS/MS | ↑ | - | T2DR | (85) |
| 514 | Glutathione trisulfide | Peptides and derivatives | Plasma | LC-ESI-MS/MS | ↓ | - | T2DR | (85) |
| 515 | Cysteine persulfides | Amino acids | Plasma | LC-ESI-MS/MS | ↑ | - | T2DR | (85) |
| 516 | Piperamide | Benzodioxoles | Plasma | HPLC-QTrap-MS/MS | ↑/↑ | √ | T2DR | (14) |
| 517 | 3-Hydroxybutanoic acid | Organic acid | Urine | GC-MS | - | - | T2DN | (49) |
| 518 | 3,4-Dihydroxybutanoic acid | Organic acid | Urine | GC-MS | - | - | T2DN | (49) |
| 519 | Acylcoenzyme A | NA | Serum | LC-MS & GC-MS | ↓ | - | T2DN | (23) |
| 520 | PC(9:0/0:0) | Phosphatidylcholine | Serum | LC-MS & GC-MS | ↓ | - | T2DN | (23) |
| 521 | Phosphoric acid | Inorganic acid | Urine | LC-MS & GC-MS | - | - | T2DN | (72) |
| 522 | 2,3,4-Trihydroxybutyric acid | Organic acid | Urine | LC-MS & GC-MS | - | - | T2DN | (72) |
| 523 | 2,3-Dihydroxy-propionic acid | Organic acid | Urine | LC-MS & GC-MS | - | - | T2DN | (72) |
| 524 | D-Ribofuranose | Monosaccharide | Urine | LC-MS & GC-MS | - | - | T2DN | (72) |
| 525 | Xylitol | Five-carbon sugar alcohol | Urine | LC-MS & GC-MS | - | - | T2DN | (72) |
| 526 | 2,3,4,5-Tetrahydroxy-pentanal oxime | NA | Urine | LC-MS & GC-MS | - | - | T2DN | (72) |
| 527 | Glucitol | Saccharides and derivatives | Urine | LC-MS & GC-MS | - | - | T2DN | (72) |
| 528 | Glucosone | Saccharides and derivatives | Urine | LC-MS & GC-MS | - | - | T2DN | (72) |
| 529 | Glucose acid | Saccharides and derivatives | Urine | LC-MS & GC-MS | - | - | T2DN | (72) |
| 530 | 3-Hydroxy isovalerate | Salts | Urine | GC-MS | ↓ | - | T2DN | (59) |
| 531 | 2-Ethyl 3-OH propionate | Esters and esters derivatives | Urine | GC-MS | ↓ | - | T2DN | (59) |
| 532 | Uridine | Pyrimidine nucleosides | Urine | GC-MS | ↓ | - | T2DN | (59) |
|  |  |  | Plasma | HPLC-UV-MS/MS | ↓ | √ | T2DN | (40) |
| 533 | 3-Hydroxy isobutyrate | Organic acid | Urine | GC-MS | ↓ | - | T2DN | (59) |
| 534 | 3-Methyl adipic acid | Tricarboxylic acids and derivatives | Urine | GC-MS | ↓ | - | T2DN | (59) |
| 535 | Tiglylglycine | Acyl glycine | Urine | GC-MS | ↓ | - | T2DN | (59) |
|  |  |  | Urine | UPLC-Q-TOF/MS | ↓ | - | T2DN | (64) |
| 536 | 3-Methyl-crotonyl glycine | Amino acids and derivatives | Urine | GC-MS | ↓ | - | T2DN | (59) |
| 537 | 2-Methyl Acetoacetate | Menthane monoterpenoids | Urine | GC-MS | ↓ | - | T2DN | (59) |
| 538 | Homovanillic acid | Methoxyphenols | Urine | GC-MS | ↓ | - | T2DN | (59) |
| 539 | 3-Hydroxy propionate |  | Urine | GC-MS | ↓ | - | T2DN | (59) |
| 540 | 8-Hydroxy-7-methylguanine | Methylated nucleoside | Urine | UPLC-Q-TOF/MS | ↑ | - | T2DN | (64) |
| 541 | Nutriacholic acid | Bile acid | Urine | UPLC-Q-TOF/MS | ↑ | - | T2DN | (64) |
| 542 | 3-Hydroxyhippuric acid | Acyl glycine | Urine | UPLC-Q-TOF/MS | ↓ | - | T2DN | (64) |
| 543 | Indole-3-carboxylic acid | Indolecarboxylic acids and derivatives | Urine | UPLC-Q-TOF/MS | ↓ | - | T2DN | (64) |
| 544 | Deoxyadenosine | Derivative of the nucleoside adenosine | Urine | UPLC-Q-TOF/MS | ↓ | - | T2DN | (64) |
| 545 | Indolelactic acid | Organic acid | Urine | UPLC-Q-TOF/MS | ↓ | - | T2DN | (64) |
| 546 | 3,4,5-Trihydroxypentanoic acid | Organic acid | Plasma | GC-MS | - | - | T2DN | (67) |
| 547 | 3-Bromo-1-propanol | Alpha-haloketones | Plasma | GC-MS | - | - | T2DN | (67) |
| 548 | Heptadecanoic acid | Fatty acid | Plasma | GC-MS | - | - | T2DN | (67) |
| 549 | Norvaline | Non-proteinogenic branched-chain amino acid | Plasma | GC-MS | - | - | T2DN | (67) |
| 550 | Monomephyl phosphate | 1,2-diacylglycerol-3-phosphates | Plasma | GC-MS | - | - | T2DN | (67) |
| 551 | Propylene glycol | 1,2-propanediol | Plasma | GC-MS | - | - | T2DN | (67) |
| 552 | Octadecanol | Fatty alcohol | Plasma | GC-MS | - | - | T2DN | (67) |
| 553 | Galactofuranoside | Psoralens | Plasma | GC-MS | - | - | T2DN | (67) |
| 554 | Succinyl-CoA | NA | Serum | UPLC-QE-Orbitrap | ↑ | - | T2DN | (79) |
| 555 | Dihydroxyacetone phosphate | Monosaccharide phosphates | Serum | UPLC-QE-Orbitrap | ↑ | - | T2DN | (79) |
| 556 | Oxaloacetate | Short-chain keto acids and derivatives | Serum | UPLC-QE-Orbitrap | ↑ | - | T2DN | (79) |
| 557 | N1-methylguanosine | Methylated nucleoside | Urine | UPLC-Q-TOF/MS | - | - | T2DN | (38) |
| 558 | 7-Methyluric acid | Xanthines | Urine | UPLC-Q-TOF/MS | - | - | T2DN | (38) |
| 559 | Guanosine | Purine nucleosides | Urine | UPLC-Q-TOF/MS | - | - | T2DN | (38) |
| 560 | Guanine | Nucleobases | Urine | UPLC-Q-TOF/MS | - | - | T2DN | (38) |
| 561 | Aspartate | Amino acids and derivatives | Urine | UPLC-Q-TOF/MS | - | - | T2DN | (38) |
| 562 | Tagatose | natural hexoketose | Urine | GC-TOF/MS | ↓ | - | T2DN | (15) |
| 563 | Uracil | Pyrimidones | Urine | GC-TOF/MS | ↓ | - | T2DN | (15) |
| 564 | Cytidine | Nucleoside | Urine | GC-TOF/MS | ↓ | - | T2DN | (15) |
|  |  |  | Plamsa | UHPLC-UV-MS/MS | ↑ | √ | T2DN | (40) |
| 565 | Ethanolamine | 1,2-aminoalcohols | Urine | GC-TOF/MS | ↓ | - | T2DN | (15) |
| 566 | Isocitric acid | Tricarboxylic acids and derivatives | Urine | GC-TOF/MS | ↓ | - | T2DN | (15) |
| 567 | 4-Hydroxybutyrate | Salts | Urine | GC-TOF/MS | ↓ | - | T2DN | (15) |
| 568 | Threonic acid | Sugar acids and derivatives | Urine | GC-TOF/MS | ↓ | - | T2DN | (15) |
| 569 | Hydroxylamine | Hydroxylamine | Urine | GC-TOF/MS | ↑ | - | T2DN | (15) |
| 570 | 3-Hexenedioic acid | Medium-chain fatty acids | Urine | GC-TOF/MS | ↑ | - | T2DN | (15) |
| 571 | 2-Deoxyerythritol | Tetrahydropyridines | Urine | GC-TOF/MS | ↓ | - | T2DN | (15) |
| 572 | Dihydrosphingosine | Ceramide | Serum | UPLC-oaTOF-MS | ↓ | - | T2DN | (16) |
| 573 | Steric acid | Aromatic monoterpenoids | Plasma | UHPLC-Q-Exactive/MS | ↓ | - | T2DN | (16) |
| 574 | Palmitoleic acid | Unsaturated fatty acid | Plasma | UHPLC-Q-Exactive/MS | ↓ | - | T2DN | (16) |
| 575 | Thymine | Hydroxypyrimidines | Plasma | HPLC-UV-MS/MS | ↑ | √ | T2DN | (40) |
| 576 | Deoxyuridine | Pyrimidine 2'-deoxyribonucleosides | Plasma | HPLC-UV-MS/MS | ↓ | √ | T2DN | (40) |
| 577 | Adenine | Purine base | Plasma | HPLC-UV-MS/MS | ↑ | √ | T2DN | (40) |
| 578 | Thymidine | Pyrimidine 2'-deoxyribonucleosides | Plasma | HPLC-UV-MS/MS | ↑ | √ | T2DN | (40) |
| 579 | Adenosine | Nucleoside | Plasma | HPLC-UV-MS/MS | ↑ | √ | T2DN | (40) |
| 580 | Butenoylcarnitine | Fatty ester lipid | Urine/Plasma | HPLC-FIA-MS/MS | ↓ | √ | T2DN | (43) |
| 581 | C-glycosyltryptophan | Indolyl carboxylic acids and derivatives | Plasma | UPLC-MS/MS & GC-MS | - | - | T2DN | (81) |
| 582 | N-acetylthreonine | N-acyl-L-alpha-amino acids | Plasma | UPLC-MS/MS & GC-MS | - | - | T2DN | (81) |
| 583 | PC(16:0/18:2) | Phosphatidylcholine | Plasma | UPLC-MS/MS & GC-MS | ↑ | √ | T2DN | (22) |
| 584 | PC(16:0/18:0) | Phosphatidylcholine | Plasma | UPLC-MS/MS & GC-MS | ↓ | √ | T2DN | (22) |
| 585 | PC(18:0/20:4) | Phosphatidylcholine | Plasma | UPLC-MS/MS & GC-MS | ↓ | √ | T2DN | (22) |
| 586 | PE(16:0/18:1) | Phosphatidylethanolamine | Plasma | UPLC-MS/MS & GC-MS | ↑ | √ | T2DN | (22) |
| 587 | PE(16:0/20:4) | Phosphatidylethanolamine | Plasma | UPLC-MS/MS & GC-MS | ↑ | √ | T2DN | (22) |
| 588 | PE(18:0/20:4) | Phosphatidylethanolamine | Plasma | UPLC-MS/MS & GC-MS | ↑ | √ | T2DN | (22) |
| 589 | PG(18:0/18:2) | Phosphatidylglycerol | Plasma | UPLC-MS/MS & GC-MS | ↑ | √ | T2DN | (22) |

**Table S3** Metabolites of PM

| No | Metabolites | Class | Sample | Analysis platform | Level | Quantify | Stages | Ref |
| --- | --- | --- | --- | --- | --- | --- | --- | --- |
| 1 | Glycine | Carboxylic acids and derivatives | Serum | UPLC-LTQ Orbitrap | ↓ | - | PM | (1) |
|  |  |  | Plasma | LC-MS/MS | ↓ | √ | PM | (2) |
|  |  |  | Serum | LC-FIA-ESI-MS/MS | ↓ | √ | PM | (3) |
|  |  |  | Serum | LC-MS | - | - | PM | (4) |
|  |  |  | Serum | UHPLC-MS | ↓ | - | PM | (5) |
|  |  |  | Serum | NMR | ↓ | - | PM | (6) |
| 2 | Lysine | Carboxylic acids and derivatives | Serum | UPLC-LTQ Orbitrap | ↓ | - | PM | (1) |
|  |  |  | Plasma | UPLC-Q-TOF/MS | ↑ | - | PM | (17) |
| 3 | trans-Cinnamic acid | Cinnamic acids and derivatives | Serum | UPLC-LTQ Orbitrap | ↓ | - | PM | (1) |
| 4 | LysoPC[18:2(9Z,12Z)] | Glycerophospholipids | Serum | UPLC-LTQ Orbitrap | ↓ | - | PM | (1) |
| 5 | LysoPC(16:0) | Glycerophospholipids | Serum | UPLC-LTQ Orbitrap | ↓ | - | PM | (1) |
|  |  |  | Plasma | UPLC-LTQ-Orbitrap | - | - | PM | (19) |
|  |  |  | Serum | LC-FIA-ESI-MS/MS | ↑ | √ | PM | (3) |
|  |  |  | Serum | LC-MS | - | - | PM | (20) |
| 6 | LysoPC[16:1(9Z)] | Glycerophospholipids | Serum | UPLC-LTQ Orbitrap | ↓ | - | PM | (1) |
| 7 | LysoPC(14:0) | Glycerophospholipids | Serum | UPLC-LTQ Orbitrap | ↓ | - | PM | (1) |
|  |  |  | Plasma | UPLC-LTQ-Orbitrap | - | - | PM | (19) |
| 8 | Leucine | Carboxylic acids and derivatives | Plasma | 1H-NMR | ↓ | - | PM | (24) |
|  |  |  |  | UPLC-Q-TOF/MS | - | - | PM | (6) |
|  |  |  |  | UPLC-Q Exactive/MS | ↑ | - | PM | (25) |
|  |  |  |  | NMR | ↑ | - | PM | (26) |
|  |  |  |  | UHPLC-MS | ↑ | - | PM | (5) |
|  |  |  |  | UPLC-Q-TOF/MS | ↓ | - | PM | (17) |
|  |  |  |  | LC-MS/MS | ↑ | √ | PM | (2) |
|  |  |  |  | UPLC-LTQ-Orbitrap | - | - | PM | (19) |
| 9 | Valine | Carboxylic acids and derivatives | Plasma | ^1^H-NMR | ↓ | - | PM | (24) |
|  |  |  |  | UPLC-Q-TOF/MS | - | - | PM | (6) |
|  |  |  |  | UPLC-Q Exactive/MS | ↑ | - | PM | (25) |
|  |  |  |  | NMR | ↑ | - | PM | (26) |
|  |  |  |  | UPLC-Q-TOF/MS | ↑ | - | PM | (17) |
|  |  |  |  | LC-MS/MS | ↑ | √ | PM | (2) |
|  |  |  |  | UPLC-LTQ-Orbitrap | - | - | PM | (19) |
|  |  |  | Plasma | LC-MS | ↑ | - | PM | (33) |
| 10 | Alanine | Carboxylic acids and derivatives | Plasma | ^1^H-NMR | ↓ | - | PM | (24) |
|  |  |  | Plasma | GC-MS/MS | ↑ | - | PM | (39) |
| 11 | Proline | Carboxylic acids and derivatives | Plasma | ^1^H-NMR | ↑ | - | PM | (24) |
|  |  |  |  | UPLC-Q-TOF/MS | ↓ | - | PM | (17) |
| 12 | Creatine | Carboxylic acids and derivatives | Plasma | ^1^H-NMR | ↓ | - | PM | (24) |
|  |  |  |  | LC-MS/MS | ↑ | - | PM | (2) |
| 13 | Choline | Organonitrogen compounds | Plasma | ^1^H-NMR | ↓ | - | PM | (24) |
|  |  |  | Plasma | UPLC-Q-TOF/MS | ↓ | - | PM | (29) |
| 14 | Histidine | Carboxylic acids and derivatives | Plasma | ^1^H-NMR | ↑ | - | PM | (24) |
| 15 | α-Glucose | Organic Oxygen compounds | Plasma | ^1^H-NMR | ↑ | - | PM | (24) |
|  |  |  | Urine | GC-MS | ↑ | - | PM | (44) |
|  |  |  | Plasma | GC-SIM-MS | ↑ | - | PM | (45) |
|  |  |  | Plasma | LC-MS | ↑ | - | PM | (33) |
|  |  |  | Plasma | UPLC-Q-TOF/MS | - | - | PM | (6) |
| 16 | β-Glucose | Organic Oxygen compounds | Plasma | ^1^H-NMR | ↑ | - | PM | (24) |
|  |  |  | Urine | GC-MS | ↑ | - | PM | (44) |
|  |  |  | Plasma | GC-SIM-MS | ↑ | - | PM | (45) |
|  |  |  | Plasma | LC-MS | ↑ | - | PM | (33) |
|  |  |  | Plasma | UPLC-TOF/MS | - | - | PM | (6) |
| 17 | Arachidyl carnitine | Fatty acyls | Serum | UPLC-TOF/MS | - | - | PM | (50) |
| 18 | Trihexosylcerarnide | Sphingolipids | Serum | UPLC-TOF/MS | - | - | PM | (50) |
| 19 | Ganglioside GA2(d18:1/22:0) | Sphingolipids | Serum | UPLC-TOF/MS | - | - | PM | (50) |
| 20 | Vitamin D2 3-glucuronide | Steroids and steroid derivatives | Serum | UPLC-TOF/MS | - | - | PM | (50) |
| 21 | Diguanosine diphosphate | (5'->5')-dinucleotides | Serum | UPLC-TOF/MS | - | - | PM | (50) |
| 22 | PC(22:6/20:4) | Glycerophospholipids | Serum | UPLC-TOF/MS | - | - | PM | (50) |
| 23 | PE(15:0/18:3) | Glycerophospholipids | Serum | UPLC-TOF/MS | - | - | PM | (50) |
| 24 | PI(18:0/16:0) | Glycerophospholipids | Serum | UPLC-TOF/MS | - | - | PM | (50) |
| 25 | PS(20:4/18:0) | Glycerophospholipids | Serum | UPLC-TOF/MS | - | - | PM | (50) |
| 26 | PG(16:0/22:5) | Glycerophospholipids | Serum | UPLC-TOF/MS | - | - | PM | (50) |
| 27 | PC[14:1(9Z)/22:1(13Z) | Glycerophospholipids | Plasma | UPLC-Q Exactive/MS | ↓ | - | PM | (51) |
| 28 | Lactic acid | Hydroxy acids and derivatives | Plasma | UPLC-Q Exactive/MS | ↑ | - | PM | (51) |
| 29 | PC[16:1(9Z)/20:3(8Z，11Z，14Z) | Glycerophospholipids | Plasma | UPLC-Q Exactive/MS | ↓ | - | PM | (51) |
| 30 | Linoleic acid | Fatty acyls | Plasma | UPLC-Q Exactive/MS | ↑ | - | PM | (51) |
|  |  |  |  | HPLC-QqQ-MS/MS | - | √ | PM | (3) |
|  |  |  |  | UPLC-Q-TOF/MS | - | - | PM | (52) |
| 31 | Oleic acid | Fatty acyls | Plasma | UPLC-Q Exactive/MS | ↑ | - | PM | (51) |
|  |  |  | Serum | HPLC-QqQ-MS/MS | - | √ | PM | (3) |
|  |  |  | Plasma | LC-MS/MS | ↑ | √ | PM | (2) |
| 32 | Palmitic acid | Fatty acyls | Plasma | UPLC-Q-Exactive/MS | ↑ | - | PM | (51) |
| 33 | SM[d18:0/16:1(9Z)] | Sphingolipids | Plasma | UPLC-Q-Exactive/MS | ↓ | - | PM | (51) |
| 34 | Betaine | Carboxylic acids and derivatives | Plasma | UPLC-Q-Exactive/MS | ↑ | - | PM | (51) |
| 35 | Arachidonic acid | Fatty acyls | Plasma | UPLC-Q-Exactive/MS | ↑ | - | PM | (51) |
| 36 | Uric acid | Imidazopyrimidines | Serum | HPLC-MS/MS | ↓ | √ | PM | (25) |
|  |  |  | Plasma | UPLC-Q-TOF/MS | ↑ | - | PM | (20) |
| 37 | Xanthine | Imidazopyrimidines | Serum | HPLC-MS/MS | ↑ | √ | PM | (25) |
|  |  |  | Serum | LC-MS | - | - | PM | (20) |
| 38 | Creatinine | Carboxylic acids and derivatives | Serum | HPLC-MS/MS | ↓ | √ | PM | (25) |
| 39 | MG(22:2(13Z,16Z)/0:0/0:0) | Glycerolipids | Plasma | UPLC-Q-TOF/MS | ↓ | - | PM | (20) |
| 40 | LysoPC (15:0) | Glycerophospholipids | Plasma | UPLC-Q-TOF/MS | ↓ | - | PM | (20) |
| 41 | Glycochenodeoxycholic-3-glucuronide | Steroids and steroid derivatives | Plasma | UPLC-Q-TOF/MS | ↓ | - | PM | (20) |
| 42 | LysoPE (0:0/16:0) | Glycerophospholipids | Plasma | UPLC-Q-TOF/MS | - | - | PM | (20) |
| 43 | LysoPC(17:0) | Glycerophospholipids | Plasma | UPLC-Q-TOF/MS | ↑ | √ | PM | (20) |
|  |  |  |  | UPLC-LTQ-Orbitrap | - | - | PM | (19) |
|  |  |  |  | LC-MS | ↓ | - | PM | (4) |
| 44 | LysoPC(18:0) | Glycerophospholipids | Plasma | UPLC-Q-TOF/MS | - | - | PM | (20) |
|  |  |  |  | UPLC-LTQ-Orbitrap | ↑ | - | PM | (19) |
|  |  |  |  | HPLC-Q-Trap/MS | ↓ | - | PM | (57) |
|  |  |  | Serum | LC-FIA-ESI-MS/MS | ↑ | √ | PM | (3) |
| 45 | LysoPC[(18:1)9Z] | Glycerophospholipids | Plasma | UPLC-Q-TOF/MS | - | - | PM | (20) |
| 46 | LysoPC[(18:2)9Z,12Z] | Glycerophospholipids | Plasma | UPLC-Q-TOF/MS | - | - | PM | (20) |
| 47 | LysoPE[(0/0:20:1)11Z] | Glycerophospholipids | Plasma | UPLC-Q-TOF/MS | - | - | PM | (20) |
| 48 | S-(PGA2)-glutathione | Carboxylic acids and derivatives | Plasma | UPLC-Q-TOF/MS | - | - | PM | (20) |
| 49 | Phytosphingosine | Organonitrogen compounds | Plasma | UPLC-Q-TOF/MS | - | - | PM | (20) |
| 50 | Citrate | Salts | Serum | GC-MS | - | - | PM | (58) |
|  |  |  |  | NMR | ↑ | - | PM | (6) |
| 51 | cis-Aconitic acid | Carboxylic acids and derivatives | Serum | GC-MS | - | - | PM | (58) |
| 52 | Fumarate | Fatty acyls | Serum | GC-MS | - | - | PM | (58) |
| 53 | Succinate | Fatty acyls | Serum | GC-MS | - | - | PM | (58) |
| 54 | Pyruvate | Keto acids and derivatives | Serum | GC-MS | - | - | PM | (58) |
|  |  |  |  | NMR | ↑ | - | PM | (6) |
| 55 | Malate | Hydroxy acids and derivatives | Serum | GC-MS | - | - | PM | (58) |
|  |  |  | Plasma | LC-MS | ↑ | - | PM | (33) |
| 56 | Pyroglutamic acid | Carboxylic acids and derivatives | Serum | GC-MS | - | - | PM | (58) |
| 57 | Alkylresorcinols C17 | NA | Plasma | GC-MS/MS | - | - | PM | (39) |
| 58 | Alkylresorcinols C19 | NA | Plasma | GC-MS/MS | - | - | PM | (39) |
| 59 | Eicosapentaenoic acid | Fatty acyls | Plasma | GC-MS/MS | - | - | PM | (39) |
| 60 | 3-Carboxy-4-methyl-5-propyl-2-furanpropanoic acid | Fatty acyls | Plasma | GC-MS/MS | - | - | PM | (39) |
| 61 | α-Tocopherol | Prenol lipids | Plasma | GC-MS/MS | - | - | PM | (39) |
| 62 | LysoPC(18:1) | Glycerophospholipids | Serum | LC-FIA-ESI-MS/MS | - | - | PM | (19) |
|  |  |  |  | LC-QTRAP-MS/MS | ↑ | - | PM | (57) |
|  |  |  |  | LC-FIA-ESI-MS/MS | ↑ | √ | PM | (3) |
|  |  |  | Plasma | LC-MS | ↓ | - | PM | (4) |
| 63 | LysoPC(18:2) | Glycerophospholipids | Serum | LC-FIA-ESI-MS/MS | ↑ | √ | PM | (3) |
|  |  |  |  | - | - | - | PM | (19) |
|  |  |  | Serum | LC-MS | - | - | PM | (4) |
| 64 | Glutamate | Carboxylic acids and derivatives | Serum | LC-FIA-ESI-MS/MS | ↑ | √ | PM | (3) |
| 65 | LysoPE(18:0) | Glycerophospholipids | Serum | LC-FIA-ESI-MS/MS | ↑ | √ | PM | (3) |
| 66 | LysoPE(18:1) | Glycerophospholipids | Serum | LC-FIA-ESI-MS/MS | ↑ | √ | PM | (3) |
| 67 | α-Hydroxybutyric Acid | Hydroxy acids and derivative | Plasma | LC-MS/MS | ↑ | - | PM | (2) |
| 68 | α-Ketobutyric acid | Keto acids and derivatives | Plasma | LC-MS/MS | ↑ | - | PM | (2) |
| 69 | α-Ketoglutaric acid | Keto acids and derivatives | Plasma | LC-MS/MS | ↑ | - | PM | (2) |
| 70 | β-Hydroxybutyric Acid | Hydroxy acids and derivatives | Plasma | LC-MS/MS | ↑ | - | PM | (2) |
| 71 | 2-Aminoadipic acid | Carboxylic acids and derivatives | Plasma | LC-MS/MS | ↑ | - | PM | (2) |
| 72 | 3-Hydroxyisobutyric acid | Hydroxy acids and derivatives | Plasma | LC-MS/MS | ↑ | - | PM | (2) |
| 73 | 3-Methyl-2-oxobutyric acid | Keto acids and derivatives | Plasma | LC-MS/MS | ↑ | - | PM | (2) |
| 74 | 3-Methyl-2-oxopentanoic acid | Keto acids and derivatives | Plasma | LC-MS/MS | ↑ | - | PM | (2) |
| 75 | 4-Methyl-2-oxobutyric acid | Organic acid | Plasma | LC-MS/MS | ↑ | - | PM | (2) |
| 76 | Hydroxyisovaleroyl carnitine | Fatty acyls | Plasma | LC-MS/MS | ↑ | - | PM | (2) |
| 77 | Isoleucine | Carboxylic acids and derivatives | Plasma | LC-MS/MS | ↑ | - | PM | (2) |
|  |  |  | Serum | NMR | ↑ | - | PM | (26) |
|  |  |  | Serum | UHPLC-MS | ↑ | - | PM | (5) |
|  |  |  | Plasma | UPLC-Q-TOF/MS | ↑ | - | PM | (17) |
|  |  |  | Plasma | UPLC-Q-TOF/MS | - | - | PM | (6) |
|  |  |  | Plasma/Urine | - | - | - | PM | (33) |
| 78 | Linoleoyl-glycerophosphocholine | NA | Plasma | LC-MS/MS | ↑ | √ | PM | (2) |
| 79 | Phenylalanine | Carboxylic acids and derivatives | Plasma | LC-MS/MS | ↑ | √ | PM | (2) |
|  |  |  |  | UPLC-Q-TOF/MS | ↓ | - | PM | (65) |
|  |  |  |  | UPLC-Q-TOF/MS | ↑ | - | PM | (17) |
|  |  |  |  | UPLC-LTQ-Orbitrap | - | - | PM | (19) |
|  |  |  | Serum | NMR | ↑ | - | PM | (6) |
| 80 | Serine | Carboxylic acids and derivatives | Plasma | LC-MS/MS | ↓ | - | PM | (2) |
|  |  |  | Urine | GC-MS | - | - | PM | (44) |
| 81 | Trigonelline | Alkaloids and derivatives | Plasma | LC-MS/MS | ↑ | - | PM | (2) |
| 82 | Tyrosine | Carboxylic acids and derivatives | Plasma | LC-MS/MS | ↑ | - | PM | (2) |
|  |  |  | Serum | NMR | ↑ | - | PM | (6) |
| 83 | Pantothenic acid | Organic oxygen compounds | Plasma | LC-MS/MS | ↑ | - | PM | (2) |
| 84 | Oleamide | Fatty acyls | Plasma | UPLC-LTQ-Orbitrap | - | - | PM | (19) |
| 85 | C17 Sphinganine | Organonitrogen compounds | Plasma | UPLC-LTQ-Orbitrap | - | - | PM | (19) |
| 86 | (4E,8E,10E-d18:3) Sphingosine | Organonitrogen compounds | Plasma | UPLC-LTQ-Orbitrap | - | - | PM | (19) |
| 87 | Anandamide (18:4, n-3) | Organonitrogen compounds | Plasma | UPLC-LTQ-Orbitrap | - | - | PM | (19) |
| 88 | LysoPC(16:1) | Glycerophospholipids | Plasma | UPLC-LTQ-Orbitrap | - | - | PM | (19) |
| 89 | LysoPC(18:3) | Glycerophospholipids | Plasma | UPLC-LTQ-Orbitrap | - | - | PM | (19) |
| 90 | LysoPC(20:5) | Glycerophospholipids | Plasma | UPLC-LTQ-Orbitrap | - | - | PM | (19) |
| 91 | LysoPC(20:4) | Glycerophospholipids | Plasma | UPLC-LTQ-Orbitrap | - | - | PM | (19) |
| 92 | LysoPC (22:6) | Glycerophospholipids | Plasma | UPLC-LTQ-Orbitrap | - | - | PM | (19) |
| 93 | SM (d18:0/16:1) | Sphingolipids | Plasma | UPLC-LTQ-Orbitrap | - | - | PM | (19) |
| 94 | Lactosylceramide (d18:1/12:0) | NA | Plasma | UPLC-LTQ-Orbitrap | - | - | PM | (19) |
| 95 | 2,3-Butanediol | Organic Oxygen compounds | Plasma | UPLC-Q-TOF/MS | - | - | PM | (52) |
| 96 | Oleamide | Fatty acyls | Plasma | UPLC-Q-TOF/MS | - | - | PM | (52) |
| 97 | Stearamide | Carboximidic acids and derivatives | Plasma | UPLC-Q-TOF/MS | - | - | PM | (52) |
| 99 | Ketooctanoic acid | Keto acids and derivatives | Plasma | UPLC-Q-TOF/MS | - | - | PM | (52) |
| 100 | Octenoic acid | Fatty acyls | Plasma | UPLC-Q-TOF/MS | - | - | PM | (52) |
| 101 | Malic acid | Hydroxy acids and derivatives | Plasma | UPLC-Q-TOF/MS | - | - | PM | (52) |
| 102 | Glucuronic acid | Organic Oxygen compounds | Plasma | UPLC-Q-TOF/MS | - | - | PM | (52) |
| 103 | Phosphoglycolic acid | Organic phosphoric acids and derivatives | Plasma | UPLC-Q-TOF/MS | - | - | PM | (52) |
| 104 | p-Cresol sulfate | Organic sulfuric acids and derivatives | Plasma | UPLC-Q-TOF/MS | - | - | PM | (52) |
| 105 | Ornithine | Carboxylic acids and derivatives | Plasma | UPLC-Q-TOF/MS | - | - | PM | (52) |
| 106 | Phosphatidylcholine | Organic dithiophosphoric acids and derivatives | Plasma | UPLC-Q-TOF/MS | - | - | PM | (52) |
| 107 | Acetylcarnitine C2 | Fatty acyls | Plasma | LC-MS | ↑ | - | PM | (4) |
| 108 | Hexose | Organic Oxygen compounds | Plasma | LC-MS | ↑ | - | PM | (4) |
| 109 | 1,5-Anhydrosorbitol | Organic Oxygen compounds | Plasma | GC-SIM-MS | ↓ | - | PM | (45) |
| 110 | Glucosamine | Organic Oxygen compounds | Plasma | GC-SIM-MS | ↑ | - | PM | (45) |
| 111 | Mannosamine | Organic Oxygen compounds | Plasma | GC-SIM-MS | ↑ | - | PM | (45) |
| 112 | Mannose | Organic Oxygen compounds | Plasma | GC-SIM-MS | ↑ | - | PM | (45) |
|  |  |  | Serum | NMR | ↑ | - | PM | (6) |
| 113 | 2-Hydroxybutyrate | Hydroxy acids and derivatives | Plasma | GC-SIM-MS | ↑ | - | PM | (45) |
|  |  |  | Plasma | LC-MS | ↑ | - | PM | (33) |
| 114 | 3-Hydroxybutyrate | Hydroxy acids and derivatives | Plasma | GC-SIM-MS | ↑ | - | PM | (45) |
| 115 | Lactate | Organic acid | Plasma | GC-SIM-MS | ↑ | - | PM | (45) |
|  |  |  | Plasma | LC-MS | ↑ | - | PM | (33) |
| 116 | Glyoxylate | NA | Plasma | GC-SIM-MS | ↑ | - | PM | (45) |
| 117 | Pyruvic acid | Keto acids and derivatives | Urine | GC-MS | - | - | PM | (44) |
| 118 | Ribose | Organic Oxygen compounds | Urine | GC-MS | - | - | PM | (44) |
| 119 | Citric acid | Carboxylic acids and derivatives | Urine | GC-MS | - | - | PM | (44) |
| 120 | Ribulose | Organic Oxygen compounds | Urine | GC-MS | - | - | PM | (44) |
| 121 | Cysteine | Carboxylic acids and derivatives | Urine | GC-MS | - | - | PM | (44) |
| 122 | meso-Erythritol | Organic Oxygen compounds | Urine | GC-MS | - | - | PM | (44) |
|  |  |  | Plasma | LC-MS | - | - | PM | (33) |
| 123 | Nicotinamide | Pyridines and derivatives | Urine | GC-MS | - | - | PM | (44) |
| 124 | Tryptophan | Indoles and derivatives | Urine | GC-MS | - | - | PM | (44) |
|  |  |  | Plasma | UPLC-Q-TOF/MS | - | - | PM | (17) |
| 125 | Diacylglycerol | Glycerolipids | Plasma | ICR-FT/MS | - | - | PM | (75) |
| 126 | Sphingomyelin | NA | Plasma | ICR-FT/MS | - | - | PM | (75) |
| 128 | Urobilinogen | Tetrapyrroles and derivatives | Plasma | ICR-FT/MS | - | - | PM | (75) |
| 129 | Threonine | Carboxylic acids and derivatives | Plasma | UPLC-Q-TOF/MS | ↓ | - | PM | (17) |
| 130 | Arginine | Carboxylic acids and derivatives | Plasma | UPLC-Q-TOF/MS | ↑ | - | PM | (17) |
| 131 | Glutamine | Carboxylic acids and derivatives | Plasma | UPLC-Q-TOF/MS | ↑ | - | PM | (17) |
| 132 | Proline | Carboxylic acids and derivatives | Plasma | UPLC-Q-TOF/MS | ↑ | - | PM | (17) |
|  |  |  | Plasma | UPLC-Q-TOF/MS | - | - | PM | (5) |
|  |  |  | Plasma | - | - | - | PM | (33) |
| 133 | Tyrosine | Carboxylic acids and derivatives | Plasma | UPLC-Q-TOF/MS | ↑ | - | PM | (17) |
| 134 | Glutamate | Carboxylic acids and derivatives | Plasma | UPLC-Q-TOF/MS | ↑ | - | PM | (17) |
| 135 | N-acetylglycine | Carboxylic acids and derivatives | Plasma | - | ↓ | - | PM | (33) |
| 136 | Citrulline | Carboxylic acids and derivatives | Plasma | - | ↓ | - | PM | (33) |
| 137 | Dimethylarginine | Carboxylic acids and derivatives | Plasma | - | ↓ | - | PM | (33) |
| 138 | 3-Methyl-2-oxobutyrate | Keto acids and derivatives | Plasma | - | ↑ | - | PM | (33) |
| 139 | 3-Methyl-2-oxovalerate | Fatty acyls | Plasma | - | ↑ | - | PM | (33) |
| 140 | 4-Methyl-2-oxopentanoate | Keto acids and derivatives | Plasma | - | ↑ | - | PM | (33) |
| 141 | Fructose | Organic Oxygen compounds | Plasma | - | ↑ | - | PM | (33) |
| 142 | 1,5-Anhydroglucitol | Organic Oxygen compounds | Plasma | - | ↓ | - | PM | (33) |
| 143 | Arabinose | Organic Oxygen compounds | Plasma | LC-MS | ↑ | - | PM | (33) |
| 144 | Octanoylcarnitine | Fatty acyls | Plasma | LC-MS | ↓ | - | PM | (33) |
| 145 | 15-Methylpalmitate | Fatty acyls | Plasma | LC-MS | ↓ | - | PM | (33) |
| 146 | 10-Heptadecenoate | Fatty acyls | Plasma | LC-MS | ↓ | - | PM | (33) |
| 147 | Adrenate (22:4n6) | Fatty acyls | Plasma | LC-MS | ↑ | - | PM | (33) |
| 148 | Arachidonate (20:4n6) | Fatty acyls | Plasma | LC-MS | ↑ | - | PM | (33) |
| 149 | Myristate (14:0) | Fatty acyls | Plasma | LC-MS | ↓ | - | PM | (33) |
| 150 | Myristoleate (14:1n5) | Fatty acyls | Plasma | LC-MS | ↓ | - | PM | (33) |
| 151 | Palmitoleate (16:1n7) | Fatty acyls | Plasma | LC-MS | ↓ | - | PM | (33) |
| 152 | Pentadecanoate (15:0) | Fatty acyls | Plasma | LC-MS | ↓ | - | PM | (33) |
| 153 | 5-Dodecenoate (12:1n7) | Fatty acyls | Plasma | LC-MS | ↓ | - | PM | (33) |
| 154 | Heptanoate (7:0) | Fatty acyls | Plasma | LC-MS | ↓ | - | PM | (33) |
| 155 | Pelargonate (9:0) | Fatty acyls | Plasma | - | ↓ | - | PM | (33) |
| 156 | Palmitoyl sphingomyelin | Sphingolipids | Plasma | LC-MS | ↓ | - | PM | (33) |
| 157 | Cholesterol | Steroids and steroid derivatives | Plasma | LC-MS | ↓ | - | PM | (33) |
| 158 | Urate | Imidazopyrimidines | Plasma | LC-MS | ↑ | - | PM | (33) |
| 159 | Glycerol | Organic Oxygen compounds | Serum | NMR | ↑ | - | PM | (6) |
| 160 | Acetoacetate | Organic acid | Serum | NMR | ↑ | - | PM | (6) |
| 161 | Docosahexaenoic acid | Fatty acyls | Serum | UPLC-Q-TOF/MS | ↑ | - | PM | (65) |
| 162 | Sorbitan oleate | Fatty acyls | Serum | UPLC-Q-TOF/MS | ↑ | - | PM | (65) |
| 163 | Methyltestosterone | Steroids and steroid derivatives | Serum | UPLC-Q-TOF/MS | ↑ | - | PM | (65) |
| 164 | Docosanamide | Fatty acyls | Serum | UPLC-Q-TOF/MS | ↑ | - | PM | (65) |
| 165 | Cholesteryl acetate | Keto acids and derivatives | Serum | UPLC-Q-TOF/MS | ↓ | - | PM | (65) |

**Table S4** Metabolites of T2DM

| No | Metabolites | Class | Sample | Analysis platform | Level | Quantify | Stages | Ref |
| --- | --- | --- | --- | --- | --- | --- | --- | --- |
| 1 | Glycine | Carboxylic acids and derivatives | Serum | GC-MS | ↓ | - | T2DM | (7) |
|  |  |  | Plasma | LC-MS/MS | - | √ | T2DM | (8) |
|  |  |  | Serum | LC-MS & GC-MS | ↑ | - | T2DM | (9) |
|  |  |  | Plasma | LC-MS/MS | - | √ | T2DM | (10) |
|  |  |  | Plasma | LC-MS & GC-MS | - | - | T2DM | (11) |
| 2 | Lysine | Carboxylic acids and derivatives | Plasma | UPLC-Q-TOF/MS | ↑ | - | T2DM | (18) |
| 5 | LysoPC(16:0) | Glycerophospholipids | Plasma | UPLC-Q-TOF/MS | ↑ | - | T2DM | (18) |
|  |  |  | Serum | UPLC-MS | - | - | T2DM | (21) |
| 7 | LysoPC(14:0) | Glycerophospholipids | Plasma | UPLC-Q-TOF/MS | ↑ | - | T2DM | (18) |
| 8 | Leucine | Carboxylic acids and derivatives | Serum | GC-MS | ↑ | - | T2DM | (7) |
|  |  |  | Plasma | NMR | ↑ | - | T2DM | (27) |
|  |  |  | Plasma/Urine | NMR | ↓ | - | T2DM | (28) |
|  |  |  | Plasma | UPLC-Q-TOF/MS | ↓ | - | T2DM | (29) |
|  |  |  | Serum | LC-MS/MS | ↑ | √ | T2DM | (30) |
|  |  |  | Serum | LC-MS & GC-MS | ↑ | - | T2DM | (9) |
|  |  |  | Plasma | UPLC-Q-TOF/MS | ↑ | - | T2DM | (18) |
|  |  |  | Urine | GC-MS | ↓ | - | T2DM | (31) |
|  |  |  | Plasma | UPLC-Q-TOF/MS | ↑ | √ | T2DM | (32) |
| 9 | Valine | Carboxylic acids and derivatives | Serum | GC-MS | ↑ | - | T2DM | (7) |
|  |  |  | Plasma | GC-MS | ↑ | - | T2DM | (34) |
|  |  |  | Serum | ^1^H-NMR | ↓ | - | T2DM | (35) |
|  |  |  | Plasma | NMR | ↑ | - | T2DM | (27) |
|  |  |  | Plasma/Urine | NMR | ↓ | - | T2DM | (27) |
|  |  |  | Serum | LC-MS/MS | ↑ | √ | T2DM | (30) |
|  |  |  | Serum | LC-MS & GC-MS | ↑ | - | T2DM | (9) |
|  |  |  | Plasma | LC-MS/MS | - | - | T2DM | (10) |
|  |  |  | Serum | UPLC-Q-Exactive | - | - | T2DM | (36) |
| 11 | Proline | Carboxylic acids and derivatives | Serum | ^1^H-NMR | ↓ | - | T2DM | (35) |
|  |  |  | Plasma | LC-MS/MS | ↑ | √ | T2DM | (12) |
|  |  |  | Plasma | LC-MS/MS | - | √ | T2DM | (10) |
| 13 | Choline | Organonitrogen compounds | Serum | UPLC-QTRAP | ↓ | - | T2DM | (41) |
| 14 | Histidine | Carboxylic acids and derivatives | Serum | ^1^H-NMR | ↑ | - | T2DM | (35) |
|  |  |  | Plasma | NMR | ↑ | - | T2DM | (27) |
| 15 | α-Glucose | Organic Oxygen compounds | Serum | ^1^H-NMR | ↑ | - | T2DM | (35) |
|  |  |  | Serum | GC-MS | ↑ | - | T2DM | (7) |
|  |  |  | Blood | LC-MS | - | - | T2DM | (46) |
|  |  |  | Plasma | NMR | ↑ | - | T2DM | (27) |
|  |  |  | Plasma | NMR | ↑ | - | T2DM | (29) |
|  |  |  | Plasma | GC×GC-MS | ↑ | - | T2DM | (47) |
|  |  |  | Plasma | LC-MS/MS | - | √ | T2DM | (8) |
|  |  |  | Plasma | LC-MS & GC-MS | - | - | T2DM | (11) |
|  |  |  | Urine | GC-MS | ↑ | - | T2DM | (31) |
|  |  |  | Plasma | UHPLC-MS/MS | - | √ | T2DM | (48) |
|  |  |  | Urine | GC-MS | - | - | T2DM | (49) |
| 28 | Lactic acid | Hydroxy acids and derivatives | Serum | LC-MS & GC-MS | ↑ | - | T2DM | (9) |
| 30 | Linoleic acid | Fatty acyls | Plasma | UPLC-Q-TOF/MS | ↓ | - | T2DM | (29) |
|  |  |  | Serum | LC-MS & GC-MS | ↑ | - | T2DM | (9) |
| 31 | Oleic acid | Fatty acyls | Serum | LC-MS & GC-MS | ↑ | - | T2DM | (9) |
|  |  |  | Urine | GC-MS | ↑ | - | T2DM | (31) |
| 32 | Palmitic acid | Fatty acyls | Urine | UPLC-Q-TOF/MS | ↑ | - | T2DM | (54) |
|  |  |  | Plasma | UPLC-Q-TOF/MS | ↓ | - | T2DM | (29) |
|  |  |  | Serum | LC-MS & GC-MS | ↑ | - | T2DM | (9) |
|  |  |  | Urine | GC-MS | ↑ | - | T2DM | (31) |
|  |  |  | Serum | GC-MS | ↓ | - | T2DM | (13) |
| 34 | Betaine | Carboxylic acids and derivatives | Serum | 1H-NMR | ↓ | - | T2DM | (35) |
| 35 | Arachidonic acid | Fatty acyls | Urine | UPLC-Q-TOF/MS | ↑ | - | T2DM | (54) |
|  |  |  | Urine | GC-MS | ↑ | - | T2DM | (31) |
| 36 | Uric acid | Imidazopyrimidines | Plasma | GC-MS/MS | - | - | T2DM | (55) |
|  |  |  | Plasma | UPLC-Q-TOF/MS | ↓ |  | T2DM | (32) |
| 38 | Creatinine | Carboxylic acids and derivatives | Plasma | NMR | ↑ | - | T2DM | (27) |
| 43 | LysoPC(17:0) | Glycerophospholipids | Serum | LC-Q-TOF/MS | - | - | T2DM | (56) |
| 44 | LysoPC(18:0) | Glycerophospholipids | Plasma | UPLC-Q-TOF/MS | ↓ | - | T2DM | (18) |
| 50 | Citrate | Salts | Serum | 1H-NMR | ↓ | - | T2DM | (35) |
|  |  |  | Plasma | NMR | ↓ | - | T2DM | (27) |
|  |  |  | Plasma/Urine | NMR | ↓ | - | T2DM | (27) |
| 52 | Fumarate | Fatty acyls | Serum | LC-MS/MS | ↑ | - | T2DM | (60) |
| 53 | Succinate | Fatty acyls | Serum | 1H-NMR | ↓ | - | T2DM | (35) |
|  |  |  | Serum | LC-MS/MS | ↑ | √ | T2DM | (60) |
|  |  |  | Plasma | UPLC-Q-TOF/MS | ↓ | √ | T2DM | (32) |
| 54 | Pyruvate | Keto acids and derivatives | Serum | 1H-NMR | ↓ | - | T2DM | (35) |
|  |  |  | Plasma | NMR | ↑ | - | T2DM | (27) |
|  |  |  | Serum | NMR | ↑ | - | T2DM | (27) |
|  |  |  | Serum | LC-MS & GC-MS | ↑ | - | T2DM | (9) |
|  |  |  | Serum | LC-MS | - | - | T2DM | (61) |
| 56 | Pyroglutamic acid | Carboxylic acids and derivatives | Plasma | UPLC-Q-TOF/MS | ↓ | - | T2DM | (29) |
| 62 | LysoPC(18:1) | Glycerophospholipids | Plasma | UPLC-Q-TOF/MS | ↑ | - | T2DM | (18) |
| 63 | LysoPC(18:2) | Glycerophospholipids | Serum | LC-MS/MS | - | √ | T2DM | (8) |
|  |  |  | Plasma | UPLC-Q-TOF/MS | ↑ | - | T2DM | (18) |
| 64 | Glutamate | Carboxylic acids and derivatives | Serum | 1H-NMR | ↓ | - | T2DM | (35) |
|  |  |  | Plasma | LC-MS/MS | ↑ | √ | T2DM | (62) |
| 71 | 2-Aminoadipic acid | Carboxylic acids and derivatives | Plasma | UPLC-QTRAP | ↓ | √ | T2DM | (63) |
| 74 | 3-Methyl-2-oxopentanoic acid | Keto acids and derivatives | Plasma | GC-MS/MS | - | - | T2DM | (55) |
| 77 | Isoleucine | Carboxylic acids and derivatives | Serum | GC-MS | ↑ | - | T2DM | (7) |
|  |  |  | Plasma | NMR | ↓ | - | T2DM | (27) |
|  |  |  | Serum/Urine | NMR | ↑ | - | T2DM | (27) |
|  |  |  | Serum | LC-MS/MS | ↑ | √ | T2DM | (30) |
|  |  |  | Serum | LC-MS/MS | - | - | T2DM | (56) |
|  |  |  | Serum | LC-MS & GC-MS | ↑ | - | T2DM | (9) |
|  |  |  | Plasma | LC-MS/MS | - | √ | T2DM | (10) |
|  |  |  | Plasma | UPLC-Q-Exactive | - | - | T2DM | (36) |
|  |  |  | Serum | LC-MS | - | - | T2DM | (61) |
|  |  |  | Urine | GC-MS | ↓ | - | T2DM | (31) |
| 79 | Phenylalanine | Carboxylic acids and derivatives | Urine | UPLC-Q-TOF/MS | ↑ | - | T2DM | (54) |
|  |  |  | Serum | 1H-NMR | ↓ | - | T2DM | (35) |
|  |  |  | Serum | GC-MS | ↓ | - | T2DM | (7) |
|  |  |  | Plasma | NMR | ↑ | - | T2DM | (27) |
|  |  |  | Plasma | NMR | ↑ | - | T2DM | (29) |
|  |  |  | Plasma | LC-MS/MS | - | √ | T2DM | (8) |
|  |  |  | Serum | LC-Q-TOF/MS | - | - | T2DM | (56) |
|  |  |  | Plasma | LC-MS/MS | - | √ | T2DM | (10) |
|  |  |  | Plasma | UPLC-Q-TOF/MS | ↑ | - | T2DM | (18) |
|  |  |  | Urine | LC-MS/MS | ↓ | √ | T2DM | (66) |
|  |  |  | Urine | GC-MS | ↑ | - | T2DM | (31) |
|  |  |  | Plasma | UHPLC-MS/MS | - | √ | T2DM | (48) |
| 80 | Serine | Carboxylic acids and derivatives | Serum | GC-MS | ↓ | - | T2DM | (7) |
|  |  |  | Serum | LC-MS & GC-MS | ↓ | - | T2DM | (9) |
|  |  |  | Plasma | LC-MS/MS | - | √ | T2DM | (10) |
|  |  |  | Plasma | UPLC-Q-TOF/MS | ↓ | - | T2DM | (18) |
| 82 | Tyrosine | Carboxylic acids and derivatives | Plasma | NMR | ↑ | - | T2DM | (27) |
|  |  |  | Plasma/Urine | NMR | ↓ | - | T2DM | (28) |
|  |  |  | Plasma | LC-MS/MS | - | √ | T2DM | (68) |
|  |  |  | Urine | GC-MS | ↓ | - | T2DM | (31) |
| 89 | LysoPC(18:3) | Glycerophospholipids | Plasma | UPLC-Q-TOF/MS | ↑ | - | T2DM | (18) |
| 90 | LysoPC(20:5) | Glycerophospholipids | Plasma | UPLC-Q-TOF/MS | ↑ | - | T2DM | (18) |
| 92 | LysoPC (22:6) | Glycerophospholipids | Plasma | UPLC-Q-TOF/MS | ↑ | - | T2DM | (18) |
| 96 | Oleamide | Fatty acyls | Plasma | UPLC-Q-TOF/MS | ↑ | - | T2DM | (18) |
| 102 | Glucuronic acid | Organic Oxygen compounds | Urine | UPLC-Q-TOF/MS | ↑ | - | T2DM | (54) |
| 105 | Ornithine | Carboxylic acids and derivatives | Serum | LC-MS & GC-MS | ↓ | - | T2DM | (9) |
|  |  |  | Plasma | LC-MS | - | - | T2DM | (69) |
| 110 | Glucosamine | Organic Oxygen compounds | Blood | LC-MS | - | - | T2DM | (46) |
| 112 | Mannose | Organic Oxygen compounds | Plasma | LC-MS | ↑ | - | T2DM | (33) |
|  |  |  | Blood | LC-MS | - | - | T2DM | (46) |
|  |  |  | Plasma | GC×GC-MS | ↑ | - | T2DM | (46) |
|  |  |  | Plasma | GC-MS/MS | - | - | T2DM | (55) |
|  |  |  | Plasma | LC-MS & GC-MS | - | - | T2DM | (11) |
|  |  |  | Urine | GC-MS | ↑ | - | T2DM | (31) |
|  |  |  | Plasma | UHPLC-MS/MS | - | √ | T2DM | (48) |
| 113 | 2-Hydroxybutyrate | Hydroxy acids and derivatives | Serum | GC-MS | ↑ | - | T2DM | (7) |
|  |  |  | Blood | LC-MS | - | - | T2DM | (46) |
|  |  |  | Serum | LC-MS | - | - | T2DM | (61) |
| 114 | 3-Hydroxybutyrate | Hydroxy acids and derivatives | Plasma | UHPLC-MS/MS | - | √ | T2DM | (48) |
| 115 | Lactate | Organic acid | Serum | 1H-NMR | ↓ | - | T2DM | (35) |
|  |  |  | Plasma | NMR | ↓ | - | T2DM | (27) |
|  |  |  | Serum | LC-MS | - | - | T2DM | (61) |
| 116 | Glyoxylate | NA | Blood | LC-MS | - | - | T2DM | (46) |
| 117 | Pyruvic acid | Keto acids and derivatives | Urine | UPLC-TOF/MS | ↓ | √ | T2DM | (32) |
| 118 | Ribose | Organic Oxygen compounds | Serum | GC-TOF/MS | ↓ | - | T2DM | (71) |
| 119 | Citric acid | Carboxylic acids and derivatives | Urine | GC-MS | ↑ | - | T2DM | (73) |
|  |  |  | Serum | GC-TOF/MS | ↑ | - | T2DM | (71) |
|  |  |  | Urine | GC-MS | ↓ | - | T2DM | (59) |
| 124 | Tryptophan | Indoles and derivatives | Urine | UPLC-Q-TOF/MS | ↓ | - | T2DM | (54) |
|  |  |  | Serum | GC-MS | ↓ | - | T2DM | (7) |
|  |  |  | Urine | HPLC-Q-TOF/MS | ↓ | √ | T2DM | (74) |
| 129 | Threonine | Carboxylic acids and derivatives | Serum | GC-MS | ↓ | - | T2DM | (7) |
|  |  |  | Serum | 1H-NMR | ↓ | - | T2DM | (35) |
|  |  |  | Serum | LC-MS & GC-MS | ↑ | - | T2DM | (9) |
|  |  |  | Urine | GC-MS | ↑ | - | T2DM | (31) |
|  |  |  | Plasma | UHPLC-MS/MS | - | √ | T2DM | (48) |
| 130 | Arginine | Carboxylic acids and derivatives | Serum | NMR | ↓ | - | T2DM | (34) |
|  |  |  | Plasma | LC-MS/MS | - | √ | T2DM | (10) |
|  |  |  | Plasma | LC-MS | - | - | T2DM | (69) |
| 131 | Glutamine | Carboxylic acids and derivatives | Plasma/Urine | NMR | ↓ | - | T2DM | (28) |
|  |  |  | Serum | 1H-NMR | ↓ | - | T2DM | (35) |
|  |  |  | Plasma | UPLC-Q-TOF/MS | ↓ | - | T2DM | (29) |
| 132 | Proline | Carboxylic acids and derivatives | Serum | LC-MS & GC-MS | ↓ | - | T2DM | (56) |
| 133 | Tyrosine | Carboxylic acids and derivatives | Serum | LC-Q-TOF/MS | - | - | T2DM | (56) |
|  |  |  | Urine | UPLC-Q-TOF/MS | ↑ | - | T2DM | (54) |
|  |  |  | Serum | LC-Q-TOF/MS | - | - | T2DM | (56) |
|  |  |  | Serum | LC-MS | - | - | T2DM | (61) |
|  |  |  | Urine | UPLC-Q-TOF/MS | ↓ | - | T2DM | (64) |
| 134 | Glutamate | Carboxylic acids and derivatives | Plasma | UPLC-Q-TOF/MS | ↑ | - | T2DM | (5) |
| 135 | N-acetylglycine | Carboxylic acids and derivatives | Serum | UPLC-MS/MS | - | - | T2DM | (76) |
| 136 | Citrulline | Carboxylic acids and derivatives | Plasma | LC-MS/MS | - | √ | T2DM | (10) |
|  |  |  | Plasma | LC-MS | - | - | T2DM | (69) |
| 141 | Fructose | Organic Oxygen compounds | Plasma | GC×GC-MS | ↑ | - | T2DM | (47) |
|  |  |  | Plasma | LC-MS & GC-MS | ↑ | - | T2DM | (11) |
|  |  |  | Urine | GC-MS | ↑ | - | T2DM | (31) |
| 142 | 1,5-Anhydroglucitol | Organic Oxygen compounds | Saliva | - | ↓ | - | T2DM | (77) |
|  |  |  | Plasma | GC×GC-MS | ↓ | - | T2DM | (47) |
|  |  |  | Plasma | LC-MS & GC-MS | - | - | T2DM | (11) |
|  |  |  | Plasma | UHPLC-MS/MS | - | √ | T2DM | (48) |
| 154 | Heptanoate (7:0) | Fatty acyls | Plasma | UHPLC-MS/MS | - | √ | T2DM | (48) |
| 155 | Pelargonate (9:0) | Fatty acyls | Plasma | UHPLC-MS/MS | - | √ | T2DM | (48) |
| 159 | Glycerol | Organic Oxygen compounds | Serum | GC-MS | ↑ | - | T2DM | (7) |
|  |  |  | Serum | LC-MS & GC-MS | ↓ | - | T2DM | (9) |
|  |  |  | Serum | LC-MS | - | - | T2DM | (61) |
| 160 | Acetoacetate | Organic acid | Serum | 1H-NMR | ↓ | - | T2DM | (35) |
| 166 | Oleic acid(C18:2) | Fatty acyls | Serum | GC-MS | ↑ | - | T2DM | (78) |
| 167 | Linoleic acid(C18:3) | Fatty acyls | Serum | GC-MS | ↑ | - | T2DM | (78) |
| 168 | Linolenic acid(C18:1) | Fatty acyls | Serum | GC-MS | ↑ | - | T2DM | (78) |
| 169 | Glutaric acid | Carboxylic acids and derivatives | Urine | UPLC-Q-TOF/MS | ↑ | - | T2DM | (54) |
|  |  |  | Serum | LC-MS/MS | ↑ | - | T2DM | (60) |
| 170 | Nicotinuric acid | Carboxylic acids and derivatives | Urine | UPLC-Q-TOF/MS | ↑ | - | T2DM | (54) |
| 171 | Paramethasone acetate |  | Urine | UPLC-Q-TOF/MS | ↑ | - | T2DM | (54) |
| 172 | Dopamine | Phenols | Urine | UPLC-Q-TOF/MS | ↓ | - | T2DM | (54) |
| 173 | Aspartic acid | Carboxylic acids and derivatives | Urine | UPLC-Q-TOF/MS | ↑ | - | T2DM | (54) |
|  |  |  | Plasma | GC-MS | - | - | T2DM | (79) |
| 174 | Urea | Organic carbonic acids and derivatives | Urine | GC-MS | - | √ | T2DM | (80) |
|  |  |  | Serum | LC-MS & GC-MS | ↑ | - | T2DM | (9) |
| 175 | Threitol | Organic Oxygen compounds | Urine | GC-MS | - | √ | T2DM | (80) |
| 176 | Sorbitol | Organic Oxygen compounds | Urine | GC-MS | - | √ | T2DM | (80) |
|  |  |  | Plasma | GC-MS/MS | - | - | T2DM | (55) |
| 177 | Inositol | Organic Oxygen compounds | Urine | GC-MS | - | √ | T2DM | (80) |
| 178 | Arabinitol | Organic Oxygen compounds | Urine | GC-MS | - | √ | T2DM | (80) |
| 179 | Trihydroxybutyric acid | Organic Oxygen compounds | Urine | GC-MS | - | √ | T2DM | (80) |
| 180 | 2-Ketoisovalerate | Keto acids and derivatives | Serum | GC-MS | ↑ | - | T2DM | (7) |
| 181 | 2-Ketoisocaproate | Keto acids and derivatives | Serum | GC-MS | ↑ | - | T2DM | (7) |
| 182 | Asparagine | Carboxylic acids and derivatives | Serum | GC-MS | ↓ | - | T2DM | (7)8) |
|  |  |  | Serum | UPLC-Q-Exactive | - | - | T2DM | (36) |
| 183 | 2-Ketoglutarate | Keto acids and derivatives | Serum | GC-MS | ↓ | - | T2DM | (7) |
| 184 | Galactose | Organic Oxygen compounds | Serum | GC-MS | ↓ | - | T2DM | (7) |
|  |  |  | Plasma | GC-MS/MS | - | - | T2DM | (55) |
|  |  |  | Serum | LC-MS & GC-MS | ↑ | - | T2DM | (9) |
| 185 | Canine uric acid | Imidazopyrimidines | Urine | HPLC-Q-TOF/MS | ↓ | √ | T2DM | (74) |
| 186 | Procurinogen canis | NA | Urine | HPLC-Q-TOF/MS | ↑ | √ | T2DM | (74) |
| 189 | Glycocholate | Steroids and steroid derivatives | Serum | UPLC-MS/MS | - | - | T2DM | (76) |
| 190 | Taurochenodeoxycholate | Steroids and steroid derivatives | Serum | UPLC-MS/MS | - | - | T2DM | (76) |
| 191 | Ursodeoxycholate | Steroids and steroid derivatives | Serum | UPLC-MS/MS | - | - | T2DM | (76) |
| 192 | Glycodeoxycholate | Steroids and steroid derivatives | Serum | UPLC-MS/MS | - | - | T2DM | (76) |
| 193 | Glycocholenate sulfate | NA | Serum | UPLC-MS/MS | - | - | T2DM | (76) |
| 194 | Taurocholenate sulfate | Salts | Serum | UPLC-MS/MS | - | - | T2DM | (76) |
| 195 | Glycoursodeoxycholate | Steroids and steroid derivatives | Serum | UPLC-MS/MS | - | - | T2DM | (76) |
| 196 | Methyl indole-3-acetate | Indoles and derivatives | Serum | UPLC-MS/MS | - | - | T2DM | (76) |
| 197 | Indolepropionate | Indoles and derivatives | Serum | UPLC-MS/MS | - | - | T2DM | (76) |
| 198 | Phenyllactate | Phenylpropanoic acids | Serum | UPLC-MS/MS | - | - | T2DM | (76) |
| 199 | Xanthurenate | Quinolines and derivatives | Serum | UPLC-MS/MS | - | - | T2DM | (76) |
| 200 | 3-(4-Hydroxyphenyl)lactate | Phenylpropanoic acids | Serum | UPLC-MS/MS | - | - | T2DM | (76) |
|  |  |  | Serum | UPLC-Q-Exactive | - | - | T2DM | (36) |
| 201 | 4-Hydroxyphenylacetate | Phenol esters | Serum | UPLC-MS/MS | - | - | T2DM | (76) |
| 202 | 3-Phenylpropionate (hydrocinnamate) | Phenylpropanoic acids | Serum | UPLC-MS/MS | - | - | T2DM | (76) |
| 203 | Phenylacetate | Benzene and substituted derivatives | Serum | UPLC-MS/MS | - | - | T2DM | (76) |
| 204 | Phenol sulfate | Organic sulfuric acids and derivatives | Serum | UPLC-MS/MS | - | - | T2DM | (76) |
| 205 | Indolelactate | Indoles and derivatives | Serum | UPLC-MS/MS | - | - | T2DM | (76) |
| 206 | N-acetyltryptophan | Carboxylic acids and derivatives | Serum | UPLC-MS/MS | - | - | T2DM | (76) |
| 207 | Indoleacetylglutamine | Carboxylic acids and derivatives | Serum | UPLC-MS/MS | - | - | T2DM | (76) |
| 208 | 3-Aminoisobutyrate | Carboxylic acids and derivatives | Serum | UPLC-MS/MS | - | - | T2DM | (76) |
| 209 | Imidazole propionate | Azoles | Serum | UPLC-MS/MS | - | - | T2DM | (76) |
| 210 | Hippurate | Carboxylic acids and derivatives | Serum | UPLC-MS/MS | - | - | T2DM | (76) |
| 211 | 2-Hydroxyhippurate (salicylurate) | Benzenoids | Serum | UPLC-MS/MS | - | - | T2DM | (76) |
| 212 | Lactate | Organic acid | Serum | UPLC-MS/MS | - | - | T2DM | (76) |
|  |  |  | Plasma/Urine | NMR | - | - | T2DM | (28) |
| 213 | Isovalerate (i5:0) | Salts | Serum | UPLC-MS/MS | - | - | T2DM | (76) |
| 214 | 2-Hydroxybutyrate/2-Hydroxyisobutyrate | Hydroxy acids and derivatives | Serum | UPLC-MS/MS | - | - | T2DM | (76) |
| 215 | 3-Hydroxyisobutyrate | Hydroxy acids and derivatives | Serum | UPLC-MS/MS | - | - | T2DM | (76) |
|  |  |  | Plasma | LC-MS & GC-MS | - | - | T2DM | (11) |
| 216 | Homovanillate | Phenols | Serum | UPLC-MS/MS | - | - | T2DM | (76) |
| 217 | Serotonin | Indoles and derivatives | Serum | UPLC-MS/MS | - | - | T2DM | (76) |
|  |  |  | Plasma | UPLC-Q-TOF/MS | ↑ | √ | T2DM | (32) |
| 218 | Spermidine | Organonitrogen compounds | Serum | UPLC-MS/MS | - | - | T2DM | (76) |
| 219 | N-acetylputrescine | Carboximidic acids and derivatives | Serum | UPLC-MS/MS | - | - | T2DM | (76) |
| 220 | 1-Linolenoyl-GPC (18:3) | Glycerophospholipids | Serum | UPLC-MS/MS | - | - | T2DM | (76) |
| 221 | 1-Linoleoyl-GPC (18:2) | Glycerophospholipids | Serum | UPLC-MS/MS | - | - | T2DM | (76) |
| 222 | 1-Oleoyl-GPC (18:1) | Glycerophospholipids | Serum | UPLC-MS/MS | - | - | T2DM | (76) |
| 223 | 1-Palmitoyl-GPC (16:0) | Glycerophospholipids | Serum | UPLC-MS/MS | - | - | T2DM | (76) |
| 224 | 1-Lignoceroyl-GPC (24:0) | Glycerophospholipids | Serum | UPLC-MS/MS | - | - | T2DM | (76) |
| 225 | 1-Stearoyl-GPC (18:0) | Glycerophospholipids | Serum | UPLC-MS/MS | - | - | T2DM | (76) |
| 226 | 1-Arachidonoyl-GPE (20:4n6) | Glycerophospholipids | Serum | UPLC-MS/MS | - | - | T2DM | (76) |
| 227 | 1-Linoleoyl-GPE (18:2) | Glycerophospholipids | Serum | UPLC-MS/MS | - | - | T2DM | (76) |
| 228 | 1-Palmitoyl-GPE (16:0) | Glycerophospholipids | Serum | UPLC-MS/MS | - | - | T2DM | (76) |
| 229 | Cholate | Steroids and steroid derivatives | Serum | UPLC-MS/MS | - | - | T2DM | (76) |
| 230 | Chenodeoxycholate | Steroids and steroid derivatives | Serum | UPLC-MS/MS | - | - | T2DM | (76) |
| 231 | Glycochenodeoxycholate | Steroids and steroid derivatives | Serum | UPLC-MS/MS | - | - | T2DM | (76) |
| 232 | Kynurenine | Organic Oxygen compounds | Serum | UPLC-MS/MS | - | - | T2DM | (76) |
| 233 | Kynurenate | Quinolines and derivatives | Serum | UPLC-MS/MS | - | - | T2DM | (76) |
| 234 | 1-Dihomo-linolenylglycerol (20:3) | Glycerolipids | Serum | UPLC-MS/MS | - | - | T2DM | (76) |
| 235 | 1-Myristoylglycerol (14:0) |  | Serum | UPLC-MS/MS | - | - | T2DM | (76) |
| 236 | 1-Oleoylglycerol (18:1) | Glycerolipids | Serum | UPLC-MS/MS | - | - | T2DM | (76) |
| 237 | 1-Palmitoylglycerol (16:0) | Glycerolipids | Serum | UPLC-MS/MS | - | - | T2DM | (76) |
| 238 | 2-Oleoylglycerol (18:1) | Glycerolipids | Serum | UPLC-MS/MS | - | - | T2DM | (76) |
| 239 | 1-Palmitoleoylglycerol (16:1) | NA | Serum | UPLC-MS/MS | - | - | T2DM | (76) |
| 240 | 1-Linolenoylglycerol (18:3) | NA | Serum | UPLC-MS/MS | - | - | T2DM | (76) |
| 241 | Methylamine | Organonitrogen compounds | Serum | 1H-NMR | ↓ | - | T2DM | (35) |
| 242 | Trimethylamine | Organonitrogen compounds | Serum | 1H-NMR | ↓ | - | T2DM | (35) |
| 243 | Cholesteryl ester (C18:1) | Steroids and steroid derivatives | Blood | LC-MS | - | - | T2DM | (46) |
| 244 | Glycochenodeoxycholic acid | Steroids and steroid derivatives | Blood | LC-MS | - | - | T2DM | (46) |
| 245 | Taurochenodeoxycholic acid | Steroids and steroid derivatives | Blood | LC-MS | - | - | T2DM | (46) |
| 246 | Lysophosphatidylcholine (C18:2) | Glycerophospholipids | Blood | LC-MS | - | - | T2DM | (46) |
| 247 | Lignoceric acid (C24:0) | Fatty acyls | Blood | LC-MS | - | - | T2DM | (46) |
| 248 | N-acetyl Glycoprotein | Proteins | Plasma | NMR | ↑ | - | T2DM | (27) |
| 249 | Formate | Salts | Plasma | NMR | ↑ | - | T2DM | (27) |
|  |  |  | Plasma/Urine | NMR | ↓ | - | T2DM | (28) |
| 250 | Acetate | Salts | Plasma | NMR | ↑ | - | T2DM | (27) |
| 251 | Alanine | Carboxylic acids and derivatives | Plasma | NMR | ↓ | - | T2DM | (27) |
|  |  |  | Plasma/Urine | NMR | ↓ | - | T2DM | (28) |
|  |  |  | Serum | LC-Q-TOF/MS | - | - | T2DM | (56) |
|  |  |  | Plasma | LC-MS/MS | - | √ | T2DM | (10) |
|  |  |  | Plasma | LC-MS/MS | ↑ | √ | T2DM | (62) |
| 252 | Glutamine | Carboxylic acids and derivatives | Plasma | NMR | ↓ | - | T2DM | (27) |
| 253 | 3-D-hydroxybutyrate | Hydroxy acids and derivatives | Plasma/Urine | NMR | ↓ | - | T2DM | (28) |
| 254 | Quinovose | Monosaccharide | Plasma | UPLC-Q-TOF/MS | ↓ | - | T2DM | (29) |
| 255 | Dulcitol | Organic Oxygen compounds | Plasma | UPLC-Q-TOF/MS | ↓ | - | T2DM | (29) |
| 256 | Lyxose | Organic Oxygen compounds | Plasma | UPLC-Q-TOF/MS | ↑ | - | T2DM | (29) |
| 257 | Hypoxanthine | Imidazopyrimidines | Plasma | UPLC-Q-TOF/MS | ↓ | - | T2DM | (29) |
| 258 | 1-Oleoyl-L-.alpha.-lysophosphatidic acid | Organic acid | Plasma | UPLC-Q-TOF/MS | ↑ | - | T2DM | (29) |
| 259 | m-Chlorohippuric acid | Benzene and substituted derivatives | Plasma | UPLC-Q-TOF/MS | ↑ | - | T2DM | (29) |
| 260 | L-Gulonic gamma-lactone | Esters and esters derivatives | Plasma | UPLC-Q-TOF/MS | ↑ | - | T2DM | (29) |
| 261 | Taurine | Organic sulfonic acids and derivatives | Plasma | UPLC-Q-TOF/MS | ↑ | - | T2DM | (29) |
|  |  |  | Plasma | LC-MS/MS | - | √ | T2DM | (8) |
|  |  |  | Plasma | UPLC-Q-TOF/MS | ↓ | √ | T2DM | (32) |
| 262 | Myristic acid | Fatty acyls | Plasma | UPLC-Q-TOF/MS | ↑ | - | T2DM | (29) |
| 263 | Lactate | Organic acid | Plasma | UPLC-Q-TOF/MS | ↓ | - | T2DM | (29) |
|  |  |  | Serum | LC-MS/MS | ↑ | √ | T2DM | (60) |
| 264 | 16-Hydroxypalmitic acid | Fatty acyls | Plasma | UPLC-Q-TOF/MS | ↓ | - | T2DM | (29) |
| 265 | Dodecanoic acid | Fatty acyls | Plasma | UPLC-Q-TOF/MS | ↓ | - | T2DM | (29) |
| 266 | 2-Hydroxy-butanoic acid | Organic acid | Plasma | UPLC-Q-TOF/MS | ↑ | - | T2DM | (29) |
| 267 | Linolenic acid | Fatty acyls | Plasma | UPLC-Q-TOF/MS | ↓ | - | T2DM | (29) |
| 268 | Norethindrone Acetate | Estrogens and derivatives | Plasma | UPLC-Q-TOF/MS | ↓ | - | T2DM | (29) |
| 269 | Myristoleic acid | Fatty acyls | Plasma | UPLC-Q-TOF/MS | ↓ | - | T2DM | (29) |
| 270 | cis-9-Palmitoleic acid | Fatty acyls | Plasma | UPLC-Q-TOF/MS | ↓ | - | T2DM | (29) |
| 271 | Capric acid | Fatty acyls | Plasma | UPLC-Q-TOF/MS | ↓ | - | T2DM | (29) |
|  |  |  | Serum | LC-MS/MS | ↑ | - | T2DM | (60) |
| 272 | 2′-Deoxy-D-ribose | Organic Oxygen compounds | Plasma | UPLC-Q-TOF/MS | ↑ | - | T2DM | (29) |
| 273 | Vitamin E | Prenol lipids | Plasma | UPLC-Q-TOF/MS | ↑ | - | T2DM | (29) |
| 274 | Sunitinib | Indoles and derivatives | Plasma | UPLC-Q-TOF/MS | ↓ | - | T2DM | (29) |
| 275 | Cholesterol 3-sulfate | Steroids and steroid derivatives | Plasma | UPLC-Q-TOF/MS | ↑ | - | T2DM | (29) |
| 276 | Ketoisocaproic acid | Keto acids and derivatives | Plasma | UPLC-Q-TOF/MS | ↑ | - | T2DM | (29) |
| 277 | Methylmalonic acid | Carboxylic acids and derivatives | Plasma | UPLC-Q-TOF/MS | ↑ | - | T2DM | (29) |
| 278 | Phenol | Phenols | Plasma | UPLC-Q-TOF/MS | ↑ | - | T2DM | (29) |
| 279 | Deoxycholic acid | Steroids and steroid derivatives | Plasma | UPLC-Q-TOF/MS | ↑ | - | T2DM | (29) |
| 280 | 1-Stearoyl-sn-glycerol | Glycerophospholipids | Plasma | UPLC-Q-TOF/MS | ↑ | - | T2DM | (29) |
| 281 | 2-Ethoxyethanol | Organic Oxygen compounds | Plasma | UPLC-Q-TOF/MS | ↓ | - | T2DM | (29) |
| 282 | Dioctyl phthalate | Benzene and substituted derivatives | Plasma | UPLC-Q-TOF/MS | ↑ | - | T2DM | (29) |
|  |  |  | Serum | GC-TOF/MS | ↑ | - | T2DM | (71) |
| 283 | 1-Stearoyl-sn-glycerol 3-phosphocholine | Esters and esters derivatives | Plasma | UPLC-Q-TOF/MS | ↓ | - | T2DM | (29) |
| 284 | 1-Oleoyl-sn-glycero-3-phosphocholine | Glycerophospholipids | Plasma | UPLC-Q-TOF/MS | ↓ | - | T2DM | (29) |
| 285 | Acetylcarnitine | Fatty acyls | Plasma | UPLC-Q-TOF/MS | ↓ | - | T2DM | (29) |
| 286 | Pipecolic acid | Carboxylic acids and derivatives | Plasma | UPLC-Q-TOF/MS | ↑ | - | T2DM | (29) |
| 287 | Erucamide | NA | Plasma | UPLC-Q-TOF/MS | ↑ | - | T2DM | (29) |
| 288 | 1-Palmitoylglycerol | Glycerolipids | Plasma | UPLC-Q-TOF/MS | ↑ | - | T2DM | (29) |
| 289 | Pristanic acid | Prenol lipids | Plasma | UPLC-Q-TOF/MS | ↓ | - | T2DM | (29) |
| 290 | D(-)-beta-hydroxy butyric acid | Fatty acyls | Plasma | UPLC-Q-TOF/MS | ↑ | - | T2DM | (29) |
| 291 | 1-Stearoyl-rac-glycerol | Glycerolipids | Plasma | UPLC-Q-TOF/MS | ↑ | - | T2DM | (29) |
| 292 | Palmitoyl ethanolamide | Carboximidic acids and derivatives | Plasma | UPLC-Q-TOF/MS | ↓ | - | T2DM | (29) |
| 293 | L-Carnitine | Organonitrogen compounds | Plasma | UPLC-Q-TOF/MS | ↓ | - | T2DM | (29) |
| 294 | Betaine | Carboxylic acids and derivatives | Plasma | UPLC-Q-TOF/MS | ↑ | - | T2DM | (29) |
| 295 | Maltose | Organic Oxygen compounds | Plasma | GC×GC-MS | ↑ | - | T2DM | (47) |
| 296 | Trehalose | Organic Oxygen compounds | Plasma | GC×GC-MS | ↑ | - | T2DM | (47) |
|  |  |  | Serum | UPLC-Q-Exactive | - | - | T2DM | (36) |
| 297 | Sedoheptulose | Organic Oxygen compounds | Plasma | GC×GC-MS | ↓ | - | T2DM | (47) |
| 298 | 12-Dilinoleoyl-Gpc (18:2/18:2) | NA | Serum | UPLC-QTRAP | ↓ | √ | T2DM | (41) |
| 299 | 1-Methylhistamine | Organonitrogen compounds | Serum | UPLC-QTRAP | ↑ | √ | T2DM | (41) |
| 300 | 1-Ribosyl-Imidazoleacetate | Esters and esters derivatives | Serum | UPLC-QTRAP | ↓ | √ | T2DM | (41) |
| 301 | 3-Amino-2-Piperidone | Carboxylic acids and derivatives | Serum | UPLC-QTRAP | ↓ | - | T2DM | (41) |
| 302 | 5-Methylthioadenosine | 5'-deoxyribonucleosides | Serum | UPLC-QTRAP | ↑ | - | T2DM | (41) |
| 303 | Alpha-hydroxyisovalerate | Fatty acyls | Serum | UPLC-QTRAP | ↓ | - | T2DM | (41) |
| 304 | Arachidonoylcholine | Organonitrogen compounds | Serum | UPLC-QTRAP | ↑ | - | T2DM | (41) |
| 305 | Cortisol | Steroids and steroid derivatives | Serum | UPLC-QTRAP | ↓ | - | T2DM | (41) |
| 306 | Docosatrienoate (22:3N3) | Fatty acyls | Serum | UPLC-QTRAP | ↓ | - | T2DM | (41) |
| 307 | Formiminoglutamate | Carboxylic acids and derivatives | Serum | UPLC-QTRAP | ↑ | - | T2DM | (41) |
| 308 | Gamma-tocopherol | Prenol lipids | Serum | UPLC-QTRAP | ↑ | - | T2DM | (41) |
| 309 | Glycerol 3-phosphate | Glycerophospholipids | Serum | UPLC-QTRAP | ↓ | - | T2DM | (41) |
| 310 | Glycerophosphoethanolamine | Glycerophospholipids | Serum | UPLC-QTRAP | ↑ | - | T2DM | (41) |
| 311 | Glycerophosphorylcholine | Glycerophospholipids | Serum | UPLC-QTRAP | ↑ | - | T2DM | (41) |
| 312 | N-Formylanthranilic Acid | Benzene and substituted derivatives | Serum | UPLC-QTRAP | ↑ | - | T2DM | (41) |
| 313 | N-Stearoyl-Sphinganine (D18:0/18:0) | Sphingolipids | Serum | UPLC-QTRAP | ↓ | - | T2DM | (41) |
| 314 | Pipecolate | Carboxylic acids and derivatives | Serum | UPLC-QTRAP | ↓ | - | T2DM | (41) |
| 315 | Ribitol | Organic Oxygen compounds | Serum | UPLC-QTRAP | ↓ | - | T2DM | (41) |
| 316 | LPC acyl C28:1 | Glycerophospholipids | Serum | LC-MS/MS | ↓ | √ | T2DM | (30) |
| 317 | PC diacyl C28:1 | Glycerophospholipids | Serum | LC-MS/MS | ↓ | √ | T2DM | (30) |
| 318 | PC acyl-alkyl C40:1 | Glycerophospholipids | Serum | LC-MS/MS | ↓ | √ | T2DM | (30) |
| 319 | PC diacyl C42:4 | Glycerophospholipids | Serum | LC-MS/MS | ↓ | √ | T2DM | (30) |
| 320 | PC acyl-alkyl C38:1 | Glycerophospholipids | Serum | LC-MS/MS | ↑ | √ | T2DM | (30) |
| 321 | PC acyl-alkyl C42:5 | Glycerophospholipids | Serum | LC-MS/MS | ↓ | √ | T2DM | (30) |
| 322 | PC acyl-alkyl C40:5 | Glycerophospholipids | Serum | LC-MS/MS | ↓ | √ | T2DM | (30) |
| 323 | PC acyl-alkyl C42:4 | Glycerophospholipids | Serum | LC-MS/MS | ↓ | √ | T2DM | (30) |
| 324 | PC acyl-alkyl C36:1 | Glycerophospholipids | Serum | LC-MS/MS | ↓ | √ | T2DM | (30) |
| 325 | PC acyl-alkyl C40:3 | Glycerophospholipids | Serum | LC-MS/MS | ↓ | √ | T2DM | (30) |
| 326 | PC diacyl C40:3 | Glycerophospholipids | Serum | LC-MS/MS | ↓ | √ | T2DM | (30) |
| 327 | PC acyl-alkyl C38:0 | Glycerophospholipids | Serum | LC-MS/MS | ↓ | √ | T2DM | (30) |
| 328 | PC acyl-alkyl C40:2 | Glycerophospholipids | Serum | LC-MS/MS | ↓ | √ | T2DM | (30) |
| 329 | Caprylic acid | Fatty acyls | Serum | LC-MS/MS | ↑ | √ | T2DM | (60) |
| 330 | Citrate | Fatty acyls | Serum | LC-MS/MS | ↑ | √ | T2DM | (60) |
| 331 | Ethylmalonic acid | Fatty acyls | Serum | LC-MS/MS | ↑ | √ | T2DM | (60) |
| 332 | Glycolic acid | Hydroxy acids and derivatives | Serum | LC-MS/MS | ↑ | √ | T2DM | (60) |
|  |  |  | Serum | GC-TOF/MS | ↑ | - | T2DM | (60) |
| 333 | β-Hydroxybutyrate | Beta hydroxy acids and derivatives | Serum | LC-MS/MS | ↑ | √ | T2DM | (60) |
| 334 | α-Hydroxybutyrate | Organic acid derived from alpha-ketobutyrate | Serum | LC-MS/MS | ↑ | √ | T2DM | (60) |
| 335 | Orotic acid | Diazines | Serum | LC-MS/MS | ↑ | √ | T2DM | (60) |
| 336 | Oxalic acid | Carboxylic acids and derivatives | Serum | LC-MS/MS | ↑ | √ | T2DM | (60) |
|  |  |  | Plasma | GC-MS/MS | - | - | T2DM | (55) |
| 337 | Oxaloacetate | Keto acids and derivatives | Serum | LC-MS/MS | ↑ | √ | T2DM | (60) |
| 338 | Sebacic acid | Fatty acyls | Serum | LC-MS/MS | ↑ | √ | T2DM | (60) |
| 339 | Myoinositol | Organic Oxygen compounds | Serum | GC-MS | ↑ | - | T2DM | (73) |
| 340 | Pseudouridine | Nucleoside and nucleotide analogues | Serum | GC-MS | ↑ | - | T2DM | (73) |
|  |  |  | Plasma | LC-MS & GC-MS | - | - | T2DM | (11) |
| 341 | p-hydroxyphenylacetic Acid | Phenols | Serum | GC-MS | ↑ | - | T2DM | (73) |
| 342 | Hippuric Acid | Benzene and substituted derivatives | Serum | GC-MS | ↑ | - | T2DM | (73) |
|  |  |  | Serum | LC-MS & GC-MS | ↑ | - | T2DM | (9) |
| 343 | Hypoxanthine | Imidazopyrimidines | Serum | GC-MS | ↓ | - | T2DM | (73) |
| 344 | Sphingomyelin C24:0 | Sphingolipids | Plasma | LC-MS/MS | - | √ | T2DM | (8) |
| 345 | Diacylglycerol C36:1 | Glycerolipids | Plasma | LC-MS/MS | - | √ | T2DM | (8) |
| 346 | Triacylglycerols C58:11 | Esters and esters derivatives | Plasma | LC-MS/MS | - | √ | T2DM | (8) |
| 347 | 5-Hydroxyindoleacetic acid | Indoles and derivatives | Plasma | LC-MS/MS | - | √ | T2DM | (8) |
| 348 | PC C36:4 | Glycerophospholipids | Plasma | LC-MS/MS | - | √ | T2DM | (8) |
| 351 | 3-Methyladipic acid | Fatty acyls | Plasma | LC-MS/MS | - | √ | T2DM | (8) |
| 350 | 2-Aminodipate | Salts | Plasma | LC-MS/MS | - | √ | T2DM | (8) |
| 351 | Isocitrate | Carboxylic acids and derivatives | Plasma | LC-MS/MS | - | √ | T2DM | (8) |
| 352 | Triacylglycerols C52:1 | Glycerolipids | Plasma | LC-MS/MS | - | √ | T2DM | (8) |
| 353 | Triacylglycerols C48:0 | Glycerolipids | Plasma | LC-MS/MS | - | √ | T2DM | (8) |
|  |  |  | Serum | LC-MS | - | - | T2DM | (61) |
| 354 | Triacylglycerols C48:1 | Glycerolipids | Plasma | LC-MS/MS | - | √ | T2DM | (8) |
|  |  |  | Serum | LC-MS | - | - | T2DM | (61) |
| 355 | Triacylglycerols C54:8 | Glycerolipids | Plasma | LC-MS/MS | - | √ | T2DM | (8) |
| 356 | Cholesterol | Steroids and steroid derivatives | Sreum | UHPLC-Q-TOF/MS | ↑ | - | T2DM | (82) |
| 357 | 25-Hydroxycholesterol | Steroids and steroid derivatives | Sreum | UHPLC-Q-TOF/MS | ↑ | - | T2DM | (82) |
| 358 | 3 α ,7 α -Dihydroxy-5 β-cholestane | Steroids and steroid derivatives | Sreum | UHPLC-Q-TOF/MS | ↑ | - | T2DM | (82) |
| 359 | 4α -Methylzymosterol-4-carboxylate | Prenol lipids | Sreum | UHPLC-Q-TOF/MS | ↑ | - | T2DM | (82) |
| 360 | 24,25-Dihydrolanosterol | Prenol lipids | Sreum | UHPLC-Q-TOF/MS | ↓ | - | T2DM | (82) |
| 361 | Gentisic acid | Benzene and substituted derivatives | Sreum | GC-TOF/MS | ↑ | - | T2DM | (71) |
| 362 | Glucosaminic acid | Organic acid | Sreum | GC-TOF/MS | ↑ | - | T2DM | (71) |
| 363 | Succinic acid | Carboxylic acids and derivatives | Sreum | GC-TOF/MS | ↑ | - | T2DM | (71) |
|  |  |  | Urine | GC-MS | ↓ | - | T2DM | (31) |
| 364 | 2-Hydroxybutanoic acid | Hydroxy acids and derivatives | Sreum | GC-TOF/MS | ↑ | - | T2DM | (71) |
| 365 | D-Erythro-sphingosine | Organonitrogen compounds | Sreum | GC-TOF/MS | ↑ | - | T2DM | (71) |
| 366 | D-Talose | Monosaccharide | Sreum | GC-TOF/MS | ↓ | - | T2DM | (71) |
| 367 | Β-Mannosylglycerate | Esters and esters derivatives | Sreum | GC-TOF/MS | ↓ | - | T2DM | (71) |
| 368 | Allylmalonic acid | Organic acid | Sreum | GC-TOF/MS | ↓ | - | T2DM | (71) |
| 369 | 3-Hydroxy-L-proline | Carboxylic acids and derivatives | Sreum | GC-TOF/MS | ↓ | - | T2DM | (71) |
| 370 | Conduritol b epoxide | Esters and esters derivatives | Sreum | GC-TOF/MS | ↑ | - | T2DM | (71) |
| 371 | 2-Ketovaleric acid | Keto acids and derivatives | Sreum | GC-TOF/MS | ↑ | - | T2DM | (71) |
| 372 | Glucose-1-phosphate | Organic Oxygen compounds | Sreum | GC-TOF/MS | ↑ | - | T2DM | (71) |
| 373 | Galacticol | Organic Oxygen compounds | Plasma | GC-MS/MS | - | - | T2DM | (55) |
| 374 | Glucaric acid-1,4-lactone |  | Plasma | GC-MS/MS | - | - | T2DM | (55) |
| 375 | 2-Hydroxybutyric acid | Hydroxy acids and derivatives | Plasma | GC-MS/MS | - | - | T2DM | (55) |
| 376 | Indolepropionic acid | Indoles and derivatives | Serum | LC-Q-TOF/MS | - | - | T2DM | (56) |
| 377 | PC(18:1/22:6) | Glycerophospholipids | Serum | LC-Q-TOF/MS | - | - | T2DM | (56) |
| 378 | LysoPC(20:1) | Glycerophospholipids | Serum | LC-Q-TOF/MS | - | - | T2DM | (56) |
| 379 | LysoPC(15:1) | Glycerophospholipids | Serum | LC-Q-TOF/MS | - | - | T2DM | (56) |
| 380 | PC(20:4/17:0) | Glycerophospholipids | Serum | LC-Q-TOF/MS | - | - | T2DM | (56) |
| 381 | PC(22:6/17:0) | Glycerophospholipids | Serum | LC-Q-TOF/MS | - | - | T2DM | (56) |
| 382 | PC(15:1/18:2) | Glycerophospholipids | Serum | LC-Q-TOF/MS | - | - | T2DM | (56) |
| 383 | LysoPE(16:0) | Glycerophospholipids | Serum | LC-Q-TOF/MS | - | - | T2DM | (56) |
| 384 | PC(18:2/15:0) | Glycerophospholipids | Serum | LC-Q-TOF/MS | - | - | T2DM | (56) |
| 385 | 2-Aminooctanoic acid | Carboxylic acids and derivatives | Serum | LC-MS & GC/MS | ↓ | - | T2DM | (9) |
| 386 | Aminomalonic acid | Carboxylic acids and derivatives | Serum | LC-MS & GC/MS | ↑ | - | T2DM | (9) |
| 387 | Phosphoserine | Carboxylic acids and derivatives | Serum | LC-MS & GC/MS | ↓ | - | T2DM | (9) |
| 388 | Gluconate | Organic Oxygen compounds | Serum | LC-MS & GC/MS | ↑ | - | T2DM | (9) |
|  |  |  | Serum | LC-MS & GC/MS | - | - | T2DM | (11) |
| 389 | 9-Decenoylcarnitine | Fatty acyls | Serum | LC-MS & GC/MS | ↓ | - | T2DM | (9) |
| 390 | Stearic acid | Fatty acyls | Serum | LC-MS & GC/MS | ↑ | - | T2DM | (9) |
| 391 | LysoPE(20:3) | Glycerophospholipids | Serum | LC-MS & GC/MS | ↓ | - | T2DM | (9) |
| 392 | LysoPE(20:3) | Glycerophospholipids | Serum | LC-MS & GC/MS | ↓ | - | T2DM | (9) |
| 393 | LysoPG(12:0) | Glycerophospholipids | Serum | LC-MS & GC/MS | ↑ | - | T2DM | (9) |
| 394 | LysoPI(16:1) | Glycerophospholipids | Serum | LC-MS & GC/MS | ↑ | - | T2DM | (9) |
| 395 | LysoPI(18:1) | Glycerophospholipids | Serum | LC-MS & GC/MS | ↑ | - | T2DM | (9) |
| 396 | LysoPI(18:2) | Glycerophospholipids | Serum | LC-MS & GC/MS | ↑ | - | T2DM | (9) |
| 397 | LysoPI(20:3) | Glycerophospholipids | Serum | LC-MS & GC/MS | ↑ | - | T2DM | (9) |
| 398 | LysoPI(20:4) | Glycerophospholipids | Serum | LC-MS & GC/MS | ↑ | - | T2DM | (9) |
| 399 | LysoPI(22:6) | Glycerophospholipids | Serum | LC-MS & GC/MS | ↑ | - | T2DM | (9) |
| 400 | 1,3-Propanediol | Polyols | Serum | LC-MS & GC/MS | ↑ | - | T2DM | (9) |
| 401 | Acylcarnitine | 5'-deoxyribonucleosides | Plasma | LC-MS/MS | - | √ | T2DM | (83) |
| 402 | Acylcarnitine C3 | NA | Plasma | LC-MS/MS | - | √ | T2DM | (10) |
| 403 | Acylcarnitine C4 | NA | Plasma | LC-MS/MS | - | √ | T2DM | (10) |
| 404 | Acylcarnitine C5 | NA | Plasma | LC-MS/MS | - | √ | T2DM | (10) |
| 405 | Acylcarnitine C6 | NA | Plasma | LC-MS/MS | - | √ | T2DM | (10) |
| 406 | Acylcarnitine C10 | NA | Plasma | LC-MS/MS | - | √ | T2DM | (10) |
| 407 | Acylcarnitine C10:1 | NA | Plasma | LC-MS/MS | - | √ | T2DM | (10) |
| 408 | Methionine | Carboxylic acids and derivatives | Plasma | LC-MS/MS | - | √ | T2DM | (10) |
|  |  |  | Serum | LC-MS | - | - | T2DM | (61) |
| 409 | Propionyl carnitine | Amino acids and derivatives | Plasma | UPLC-Q-TOF/MS | ↑ | - | T2DM | (18) |
| 410 | Octanoyl carnitine | Amino acids and derivatives | Plasma | UPLC-Q-TOF/MS | ↑ | - | T2DM | (18) |
| 411 | Decanoyl carnitine | Amino acids and derivatives | Plasma | UPLC-Q-TOF/MS | ↑ | - | T2DM | (18)9 |
| 412 | Dodecanoyl carnitine | Amino acids and derivatives | Plasma | UPLC-Q-TOF/MS | ↑ | - | T2DM | (18) |
| 413 | Heptadecanoyl carnitine | Amino acids and derivatives | Plasma | UPLC-Q-TOF/MS | ↑ | - | T2DM | (18) |
| 414 | Linoleyl carnitine | Fatty acyls | Plasma | UPLC-Q-TOF/MS | ↑ | - | T2DM | (18) |
| 415 | Vaccenyl carnitine | - | Plasma | UPLC-Q-TOF/MS | ↑ | - | T2DM | (18) |
| 416 | LysoPC(16:1) | Glycerophospholipids | Plasma | UPLC-Q-TOF/MS | ↑ | - | T2DM | (18) |
| 417 | LysoPE(18:1) | Glycerophospholipids | Plasma | UPLC-Q-TOF/MS | ↓ | - | T2DM | (18) |
| 418 | LysoPE(18:2) | Glycerophospholipids | Plasma | UPLC-Q-TOF/MS | ↑ | - | T2DM | (18) |
| 419 | LysoPE(22:6) | Glycerophospholipids | Plasma | UPLC-Q-TOF/MS | ↑ | - | T2DM | (18) |
| 420 | PC ae C34:3 | Phosphatidylcholine | Plasma | LC-MS/MS | ↓ | √ | T2DM | (62) |
| 421 | PC ae C44:6 | Phosphatidylcholine | Plasma | LC-MS/MS | ↓ | √ | T2DM | (62) |
| 422 | PC ae C42:4 | Phosphatidylcholine | Plasma | LC-MS/MS | ↓ | √ | T2DM | (62) |
| 423 | PC ae C32:2 | Phosphatidylcholine | Plasma | LC-MS/MS | ↓ | √ | T2DM | (62) |
| 424 | PC ae C44:3 | Phosphatidylcholine | Plasma | LC-MS/MS | ↓ | √ | T2DM | (62) |
| 425 | PC ae C44:4 | Phosphatidylcholine | Plasma | LC-MS/MS | ↓ | √ | T2DM | (62) |
| 426 | PC aa C42:0 | Phosphatidylcholine | Plasma | LC-MS/MS | ↓ | √ | T2DM | (62) |
| 427 | PC aa C42:2 | Phosphatidylcholine | Plasma | LC-MS/MS | ↓ | √ | T2DM | (62) |
| 428 | Erythritol | Saccharides and derivatives | Serum | UPLC-Q-Exactive | - | - | T2DM | (36) |
| 429 | PC(O-34:2) | Phosphatidylcholine | Serum | LC-MS | - | - | T2DM | (61) |
| 430 | 2-Oxoglutaric acid | Gamma-keto acids and derivatives | Serum | LC-MS | - | - | T2DM | (61) |
| 431 | Glycine betaine | Small N-trimethylated amino acid | Serum | LC-MS | - | - | T2DM | (61) |
| 432 | Triacylglycerols C50:5 | Esters and esters derivatives | Serum | LC-MS | - | - | T2DM | (61) |
| 433 | Cyclic AMP | Adenine nucleotide containing one phosphate group | Urine | LC-MS/MS | ↓ | √ | T2DM | (66) |
| 434 | 5′-Methylthioadenosine | 5'-deoxy-5'-thionucleosides | Urine | LC-MS/MS | ↑ | √ | T2DM | (66) |
| 435 | Acetylhistidine | Histidine and derivatives | Urine | LC-MS/MS | ↓ | √ | T2DM | (66) |
| 436 | 3-Hydroxypyruvate | Salts | Plasma | LC-MS & GC-MS | - | - | T2DM | (11) |
| 437 | 2-Hydroxybutyrate | Salts | Plasma | LC-MS & GC-MS | - | - | T2DM | (11) |
| 438 | 2-Aminobutyrate | Salts | Plasma | LC-MS & GC-MS | - | - | T2DM | (11) |
| 439 | 2-Ketobutyrate | Esters and esters derivatives | Plasma | LC-MS & GC-MS | - | - | T2DM | (11) |
| 440 | 3-Hydroxypropanoate | Salts | Plasma | LC-MS & GC-MS | - | - | T2DM | (11) |
| 441 | Pyroglutamine | Alpha amino acids and derivatives | Plasma | LC-MS & GC-MS | - | - | T2DM | (11) |
| 442 | 3-Methoxytyrosine | Tyrosine and derivatives | Plasma | LC-MS & GC-MS | - | - | T2DM | (11) |
| 443 | O-Sulfo-L-tyrosine | Phenylalanine and derivatives | Plasma | LC-MS & GC-MS | - | - | T2DM | (11) |
| 444 | γ-Glutamylglutamine | Dipeptide | Plasma | LC-MS & GC-MS | - | - | T2DM | (11) |
| 445 | Acetacetic acid | Organic acid | Urine | GC-MS | ↑ | - | T2DM | (31) |
| 446 | PC(22:2/16:0) | Phosphatidylcholine | Serum | UPLC-MS | - | - | T2DM | (21) |
| 447 | PC(20:2/18:0) | Phosphatidylcholine | Serum | UPLC-MS | - | - | T2DM | (21) |
| 448 | PC(20:1/18:1) | Phosphatidylcholine | Serum | UPLC-MS | - | - | T2DM | (21) |
| 449 | PC(O-20:0/16:0) | Phosphatidylcholine | Serum | UPLC-MS | - | - | T2DM | (21) |
| 450 | PC(O-18:0/18:0) | Phosphatidylcholine | Serum | UPLC-MS | - | - | T2DM | (21) |
| 451 | PC(O-16:0/20:0) | Phosphatidylcholine | Serum | UPLC-MS | - | - | T2DM | (21) |
| 452 | PC(O-14:0/22:0) | Phosphatidylcholine | Serum | UPLC-MS | - | - | T2DM | (21) |
| 453 | GlcCer(d18:0/22:0) | Glycosphingolipid | Serum | UPLC-MS | - | - | T2DM | (21) |
| 454 | Tetrahydroxyhexanoic acid | Medium-chain hydroxy acids and derivatives | Serum | UPLC-MS | - | - | T2DM | (21) |
| 455 | Itaconic acid | Branched fatty acids | Plasma | UPLC-Q-TOF/MS | ↑ | √ | T2DM | (32) |
| 456 | Inosine | Purine nucleosides | Plasma | UPLC-Q-TOF/MS | ↓ | √ | T2DM | (32) |
| 457 | 3-Hydroxymethyl-glutaric acid | Organic acid | Plasma | UPLC-Q-TOF/MS | ↓ | √ | T2DM | (32) |
| 458 | PC(18:0/0:0) | Phosphatidylcholine | Plasma | UPLC-Q-TOF/MS | ↓ | √ | T2DM | (32) |
| 459 | Sphingosine-1-phosphate | Phosphosphingolipids | Plasma | UPLC-Q-TOF/MS | ↑ | √ | T2DM | (32) |
| 460 | PE(P-16:0/22:6) | Glycerophospholipids | Plasma | UPLC-Q-TOF/MS | ↓ | √ | T2DM | (32) |
| 461 | PG(18:0/18:1) | Phosphatidylglycerol or glycerophospholipid | Plasma | UPLC-Q-TOF/MS | ↑ | √ | T2DM | (32) |
| 462 | 2-Ketobutyric acid | Organic acid | Plasma | UPLC-Q-TOF/MS | ↓ | √ | T2DM | (32) |
| 463 | 2-Ketoglutaric acid | Organic acid | Plasma | UPLC-Q-TOF/MS | ↓ | √ | T2DM | (32) |
| 464 | 1-Methylhistidine | Histidine and derivatives | Plasma | UPLC-Q-TOF/MS | ↓ | √ | T2DM | (32) |
| 465 | N-Acetyl-D-phenylalanine |  | Plasma | UPLC-Q-TOF/MS | ↑ | √ | T2DM | (32) |
| 466 | Xanthurenic acid | Quinoline carboxylic acids | Plasma | UPLC-Q-TOF/MS | ↓ | √ | T2DM | (32) |
| 467 | Desoxyhexose | Monosaccharide | Plasma | UHPLC-MS/MS | - | √ | T2DM | (48) |
| 468 | Uronic acid | Saccharides and derivatives | Plasma | UHPLC-MS/MS | - | √ | T2DM | (48) |
| 469 | Dihexose(2H) | Saccharides and derivatives | Plasma | UHPLC-MS/MS | - | √ | T2DM | (48) |
| 470 | Caproate (6:0) | Salts | Plasma | UHPLC-MS/MS | - | √ | T2DM | (48) |
| 471 | PC a C20:4 | Phosphatidylcholine | Plasma | UHPLC-MS/MS | - | √ | T2DM | (48) |
| 472 | PC aa (OH, COOH) C28:4 | Phosphatidylcholine | Plasma | UHPLC-MS/MS | - | √ | T2DM | (48) |
| 473 | PC aa C34:4 | Phosphatidylcholine | Plasma | UHPLC-MS/MS | - | √ | T2DM | (48) |
| 474 | Glutamylvaline | Dipeptide | Plasma | UHPLC-MS/MS | - | √ | T2DM | (48) |
| 475 | gamma-glutamylisoleucine | Dipeptide | Plasma | UHPLC-MS/MS | - | √ | T2DM | (48) |
| 476 | Phenylacetylglutamine | Amino acids and derivatives | Plasma | UHPLC-MS/MS | - | √ | T2DM | (48) |
| 477 | 3-indoxyl sulfate | Dietary protein | Plasma | UHPLC-MS/MS | - | √ | T2DM | (48) |
| 478 | Homocitrulline | Saccharides and derivatives | Plasma | UHPLC-MS/MS | - | √ | T2DM | (48) |
| 479 | Isobutyrylcarnitine | Amino acids and derivatives | Plasma | LC-MS/MS | ↑ | √ | T2DM | (12) |
| 480 | 2,3 methylbutyryl-carnitine (C5) | Amino acids and derivatives | Plasma | LC-MS/MS | ↓ | √ | T2DM | (12) |
| 481 | b-Hydroxybutyryl-carnitine (C4-OH) | Amino acids and derivatives | Plasma | LC-MS/MS | ↑ | √ | T2DM | (12) |
| 482 | Methylmalonyl/succinyl-carnitine (C4-DC) | Amino acids and derivatives | Plasma | LC-MS/MS | ↓ | √ | T2DM | (12) |
| 483 | Glutaryl-carnitine (C5-DC) | Amino acids and derivatives | Plasma | LC-MS/MS | ↓ | √ | T2DM | (12) |
| 484 | 3-Hydroxyisovaleryl-carnitine (C5-OH) | Amino acids and derivatives | Plasma | LC-MS/MS | ↓ | √ | T2DM | (12) |

**Table S5** Metabolites of T2DK

| No | Metabolites | Class | Sample | Analysis platform | Level | Quantify | Stages | Ref |
| --- | --- | --- | --- | --- | --- | --- | --- | --- |
| 1 | Glycine | Carboxylic acids and derivatives | Plasma | LC-MS/MS | ↑ | √ | T2DK | (12) |
| 14 | Histidine | Carboxylic acids and derivatives | Plasma | LC-MS/MS | ↑ | √ | T2DK | (12) |
| 80 | Serine | Carboxylic acids and derivatives | Plasma | LC-MS/MS | ↑ | √ | T2DK | (12) |
| 107 | Acetylcarnitine C2 | Fatty acyls | Plasma | LC-MS/MS | ↓ | √ | T2DK | (12) |
| 131 | Glutamine | Carboxylic acids and derivatives | Plasma | LC-MS/MS | ↓ | √ | T2DK | (12) |
| 251 | Alanine | Carboxylic acids and derivatives | Plasma | LC-MS/MS | ↑ | √ | T2DK | (12) |
| 408 | Methionine | Carboxylic acids and derivatives | Plasma | LC-MS/MS | ↑ | √ | T2DK | (12) |

| No | Metabolites | Class | Sample | Analysis platform | Level | Quantify | Stages | Ref |
| --- | --- | --- | --- | --- | --- | --- | --- | --- |
| 1 | Glycine | Carboxylic acids and derivatives | Serum | GC-MS | ↓ | - | T2DPN | (13) |
| 30 | Linoleic acid | Fatty acyls | Serum | GC-MS | ↓ | - | T2DPN | (13) |
| 96 | Oleamide | Fatty acyls | Serum | GC-MS | ↓ | - | T2DPN | (13) |
| 129 | Threonine | Carboxylic acids and derivatives | Serum | GC-MS | ↑ | - | T2DPN | (13) |
| 174 | Urea | Organic carbonic acids and derivatives | Serum | GC-MS | ↓ | - | T2DPN | (13) |
| 178 | Arabinitol | Organic Oxygen compounds | Serum | GC-MS | ↓ | - | T2DPN | (13) |
| 261 | Taurine | Organic sulfonic acids and derivatives | Plasma | GC-MS | ↓ | - | T2DPN | (13) |
| 342 | Hippuric Acid | Benzene and substituted derivatives | Serum | GC-MS | ↑ | - | T2DPN | (13) |
| 485 | Hexuronic acid |  | Serum | GC-MS | ↓ | - | T2DPN | (13) |
| 486 | β-hydroxymyristic acid | Fatty acid | Serum | GC-MS | ↓ | - | T2DPN | (13) |
| 487 | Nicotinic acid | Water-soluble vitamin | Serum | GC-MS | ↓ | - | T2DPN | (13) |
| 488 | Phenylethanol | Alcohols | Serum | GC-MS | ↓ | - | T2DPN | (13) |
| 489 | 3-Urea propionic acid | Isocoumarans | Serum | GC-MS | ↓ | - | T2DPN | (13) |
| 490 | Benzyl alcohol | Alcohols | Serum | GC-MS | ↓ | - | T2DPN | (13) |
| 491 | Octoic acid | Organic acid | Serum | GC-MS | ↓ | - | T2DPN | (13) |
| 492 | Pyroglutamate | Salts | Serum | GC-MS | ↓ | - | T2DPN | (13) |
| 493 | D-xylose | Polysaccharide | Serum | GC-MS | ↓ | - | T2DPN | (13) |
| 494 | Maleimide | Nitrogen mustard compounds | Serum | GC-MS | ↓ | - | T2DPN | (13) |
| 495 | Boric acid | Metalloid oxides | Serum | GC-MS | ↑ | - | T2DPN | (13) |

**Table S6** Metabolites of T2DPN

**Table S7** Metabolites of T2DR

| No | Metabolites | Class | Sample | Analysis platform | Level | Quantify | Stages | Ref |
| --- | --- | --- | --- | --- | --- | --- | --- | --- |
| 112 | Mannose | Organic Oxygen compounds | Plasma | GC-MS | ↑ | - | T2DR | (70) |
| 118 | Ribose | Organic Oxygen compounds | Plasma | GC-MS | ↑ | - | T2DR | (70) |
| 129 | Threonine | Carboxylic acids and derivatives | Aqueous humor | NMR | ↑ | - | T2DR | (42) |
| 131 | Glutamine | Carboxylic acids and derivatives | Aqueous humor | NMR | ↑ | - | T2DR | (42) |
| 132 | Proline | Carboxylic acids and derivatives | Plasma | HPLC-QTrap-MS/MS | ↑/↑ | √ | T2DR | (14) |
| 174 | Urea | Organic carbonic acids and derivatives | Plasma | GC-MS | ↑ | - | T2DR | (70) |
| 182 | Asparagine | Carboxylic acids and derivatives | Plasma | GC-MS | ↑ | - | T2DR | (70) |
| 184 | Galactose | Organic Oxygen compounds | Plasma | GC-MS | ↑ | - | T2DR | (70) |
| 212 | Lactate | Organic acid | Aqueous humor | NMR | ↓ | - | T2DR | (42) |
| 295 | Maltose | Organic Oxygen compounds | Plasma | GC-MS | ↑ | - | T2DR | (70) |
| 428 | Erythritol | Saccharides and derivatives | Plasma | GC-MS | ↑ | - | T2DR | (70) |
| 496 | PC[14:1(9Z) /22:2(13Z,16Z)] | Phosphatidylcholine | Serum | UPLC-Q-TOF/MS | ↓ | - | T2DR | (84) |
| 497 | 3-Hydroxysuberic acid | Medium-chain hydroxy acids and derivatives | Serum | UPLC-Q-TOF/MS | ↑ | - | T2DR | (84) |
| 498 | Kynurenic acid | NA | Serum | UPLC-Q-TOF/MS | ↓ | - | T2DR | (84) |
| 499 | PC[14:0/20:2(11Z,14Z)] | Phosphatidylcholine | Serum | UPLC-Q-TOF/MS | ↓ | - | T2DR | (84) |
| 500 | Ritalinic acid | Beta Amino Acids and Derivatives | Serum | UPLC-Q-TOF/MS | ↓ | - | T2DR | (84) |
| 501 | Hydroxycotinine | Pyrrolidinylpyridines | Serum | UPLC-Q-TOF/MS | ↑ | - | T2DR | (84) |
| 502 | 5’- Hydroxytenoxicam | Thienothiazines | Serum | UPLC-Q-TOF/MS | ↓ | - | T2DR | (84) |
| 503 | PC[14: 0/22: 5(4Z,7Z,10Z,13Z,16Z)] | Phosphatidylcholine | Serum | UPLC-Q-TOF/MS | ↓ | - | T2DR | (84) |
| 504 | Dimethylsulfide | Volatile sulfur compound | Serum | UPLC-Q-TOF/MS | ↑ | - | T2DR | (84) |
| 505 | Acetic acid | Carboxylic acids | Serum | UPLC-Q-TOF/MS | ↑ | - | T2DR | (84) |
| 506 | 1,5-Gluconolactone | Lactone or oxidized derivative of glucose | Plasma | GC-MS | ↑ | - | T2DR | (70) |
| 507 | 1,5-Anhydroglucitol | Saccharides and derivatives | Plasma | GC-MS | ↓ | - | T2DR | (70) |
|  |  |  | Plasma | GC-MS | - | - | T2DN | (67) |
| 508 | 2-Deoxyribonic acid | Nucleotide | Plasma | GC-MS | ↑ | - | T2DR | (70) |
| 509 | 3,4-Dihydroxybutyric acid | Organic acid | Plasma | GC-MS | ↑ | - | T2DR | (70) |
| 510 | Gluconic acid | Saccharides and derivatives | Plasma | GC-MS | ↑ | - | T2DR | (70) |
| 511 | Glutathione | Peptides and derivatives | Plasma | LC-ESI-MS/MS | ↑ | - | T2DR | (85) |
| 512 | Glutathione hydropersulfide | Peptides and derivatives | Plasma | LC-ESI-MS/MS | ↓ | - | T2DR | (85) |
| 513 | Glutathione disulfide | Peptides and derivatives | Plasma | LC-ESI-MS/MS | ↑ | - | T2DR | (85) |
| 514 | Glutathione trisulfide | Peptides and derivatives | Plasma | LC-ESI-MS/MS | ↓ | - | T2DR | (85) |
| 515 | Cysteine persulfides | Amino acids | Plasma | LC-ESI-MS/MS | ↑ | - | T2DR | (85) |
| 516 | Piperamide | Benzodioxoles | Plasma | HPLC-QTrap-MS/MS | ↑/↑ | √ | T2DR | (14) |

**Table S8** Metabolites of T2DN

| No | Metabolites | Class | Sample | Analysis platform | Level | Quantify | Stages | Ref |
| --- | --- | --- | --- | --- | --- | --- | --- | --- |
| 1 | Glycine | Carboxylic acids and derivatives | Plasma | HPLC-QTrap-MS/MS | ↑/↑ | √ | T2DN | (14) |
|  |  |  | Urine | GC-TOF/MS | ↓ | - | T2DN | (15) |
|  |  |  | Serum | UPLC-oaTOF-MS | ↑ | - | T2DN | (16) |
| 2 | Lysine | Carboxylic acids and derivatives | Serum | UPLC-oaTOF-MS | ↑ | - | T2DN | (16) |
| 5 | LysoPC(16:0) | Glycerophospholipids | Plasma | UPLC-MS/MS | ↑ | √ | T2DN | (22) |
| 7 | LysoPC(14:0) | Glycerophospholipids | Serum | LC-MS & GC-MS | ↓ | - | T2DN | (23) |
| 8 | Leucine | Carboxylic acids and derivatives | Plasma | HPLC-QTrap-MS/MS | ↑/↑ | √ | T2DN | (14) |
|  |  |  | Serum | UPLC-oaTOF-MS | ↓ | - | T2DN | (16) |
| 9 | Valine | Carboxylic acids and derivatives | Plasma | HPLC-QTrap-MS/MS | ↑/↑ | √ | T2DN | (14) |
|  |  |  | Serum | LC-MS & GC-MS | ↑ | - | T2DN | (23) |
|  |  |  | Blood | HPLC-MRM | ↑ | √ | T2DN | (37) |
|  |  |  | Urine | UPLC-Q-TOF/MS | - | - | T2DN | (38) |
| 13 | Choline | Organonitrogen compounds | Plasma | HPLC-UV-MS/MS | ↓ | √ | T2DN | (40) |
| 14 | Histidine | Carboxylic acids and derivatives | Aqueous humor | NMR | ↑ | - | T2DN | (42) |
|  |  |  | Plasma | HPLC-QTrap-MS/MS | ↑/↑ | √ | T2DN | (14) |
|  |  |  | Urine | UPLC-Q-TOF/MS | - | - | T2DN | (38) |
|  |  |  | Plasma/Urine | HPLC-FIA-MS/MS | ↑ | √ | T2DN | (43) |
| 15 | α-Glucose | Organic Oxygen compounds | Serum | LC-MS & GC-MS | ↑ | - | T2DN | (23) |
| 30 | Linoleic acid | Fatty acyls | Plasma | UHPLC-Q-Exactive/MS | ↓ | - | T2DN | (53) |
| 31 | Oleic acid | Fatty acyls | Plasma | UHPLC-Q-Exactive/MS | ↓ | - | T2DN | (53) |
| 32 | Palmitic acid | Fatty acyls | Serum | LC-MS & GC-MS | ↑ | - | T2DN | (23) |
|  |  |  | Urine | GC-TOF/MS | ↑ | - | T2DN | (23) |
|  |  |  | Plasma | UPLC-Q-Exactive/MS | ↓ | - | T2DN | (16) |
| 35 | Arachidonic acid | Fatty acyls | Plasma | UPLC-Q-Exactive/MS | ↑ | - | T2DN | (53) |
| 36 | Uric acid | Imidazopyrimidines | Urine | UPLC-Q-TOF/MS | - | - | T2DN | (38) |
|  |  |  | Urine | GC-TOF/MS | ↑ | - | T2DN | (15) |
|  |  |  | Plasma | HPLC-UV-MS/MS | ↑ | √ | T2DN | (40) |
| 37 | Xanthine | Imidazopyrimidines | Urine | UPLC-Q-TOF/MS | - | - | T2DN | (38) |
|  |  |  | Plasma | HPLC-UV-MS/MS | ↑ | √ | T2DN | (40) |
| 38 | Creatinine | Carboxylic acids and derivatives | Plasma | HPLC-UV-MS/MS | ↑ | √ | T2DN | (40) |
| 44 | LysoPC(18:0) | Glycerophospholipids | Plasma | UPLC-MS/MS | ↑ | √ | T2DN | (22) |
| 49 | Phytosphingosine | Organonitrogen compounds | Serum | UPLC-oaTOF-MS | ↓ | - | T2DN | (16) |
| 51 | cis-Aconitic acid | Carboxylic acids and derivatives | Urine | GC-MS | ↓ | - | T2DN | (59) |
|  |  |  | Urine | GC-TOF/MS | ↓ | - | T2DN | (15) |
| 62 | LysoPC(18:1) | Glycerophospholipids | Plasma | UPLC-MS/MS | ↑ | √ | T2DN | (22) |
| 77 | Isoleucine | Carboxylic acids and derivatives | Serum | LC-MS & GC-MS | ↑ | - | T2DN | (32) |
|  |  |  | Urine | UPLC-Q-TOF/MS | ↑ | - | T2DN | (64) |
| 79 | Phenylalanine | Carboxylic acids and derivatives | Blood | GC-MS | ↑ | √ | T2DN | (37) |
|  |  |  | Urine | UPLC-Q-TOF/MS | ↑ | - | T2DN | (64) |
|  |  |  | Plasma | GC-MS | - | - | T2DN | (67) |
| 80 | Serine | Carboxylic acids and derivatives | Urine | GC-TOF/MS | ↓ | - | T2DN | (15) |
| 82 | Tyrosine | Carboxylic acids and derivatives | Blood | HPLC-MRM | ↓ | - | T2DN | (37) |
| 91 | LysoPC(20:4) | Glycerophospholipids | Plasma | UPLC-MS/MS | ↑ | √ | T2DN | (22) |
| 105 | Ornithine | Carboxylic acids and derivatives | Plasma | HPLC-QTrap-MS/MS | ↑/↑ | √ | T2DN | (14) |
|  |  |  | Blood | HPLC-MRM | ↓ | √ | T2DN | (37) |
| 108 | Hexose | Organic Oxygen compounds | Urine/Plasma | HPLC-FIA-MA/MS | ↑ | √ | T2DN | (43) |
| 118 | Ribose | Organic Oxygen compounds | Urine | GC-MS | - | - | T2DN | (72) |
| 121 | Cysteine | Carboxylic acids and derivatives | Plasma | HPLC-QTrap-MS/MS | ↑ | √ | T2DN | (14) |
| 124 | Tryptophan | Indoles and derivatives | Plasma | HPLC-QTrap-MS/MS | ↑/↑ | √ | T2DN | (14) |
|  |  |  | Urine | UPLC-Q-TOF/MS | ↓ | - | T2DN | (64) |
|  |  |  | Plasma | GC-MS | - | - | T2DN | (67) |
| 129 | Threonine | Carboxylic acids and derivatives | Plasma | HPLC-QTrap-MS/MS | ↑/↑ | √ | T2DN | (14) |
| 130 | Arginine | Carboxylic acids and derivatives | Plasma | HPLC-QTrap-MS/MS | ↑/↓ | √ | T2DN | (14) |
| 131 | Glutamine | Carboxylic acids and derivatives | Serum | LC-MS & GC-MS | ↓ | - | T2DN | (23) |
|  |  |  | Plasma/Urine | HPLC-FIA-MS/MS | ↑ | √ | T2DN | (43) |
| 133 | Tyrosine | Carboxylic acids and derivatives | Plasma | GC-MS | - | - | T2DN | (67) |
|  |  |  | Urine/Plasma | HPLC-FIA-MS/MS | ↑ | √ | T2DN | (43) |
| 136 | Citrulline | Carboxylic acids and derivatives | Plasma | HPLC-QTrap-MS/MS | ↑/↑ | √ | T2DN | (14) |
|  |  |  | Blood | HPLC-MRM | ↓ | √ | T2DN | (37) |
| 141 | Fructose | Organic Oxygen compounds | Urine | GC-MS | - | - | T2DN | (72) |
| 142 | 1,5-Anhydroglucitol | Organic Oxygen compounds | Urine | GC-TOF/MS | ↑ | - | T2DN | (15) |
| 175 | Threitol | Organic Oxygen compounds | Urine | GC-MS | - | - | T2DN | (49) |
| 182 | Asparagine | Carboxylic acids and derivatives | Plasma | HPLC-QTrap-MS/MS | ↑/↑ | √ | T2DN | (14) |
| 251 | Alanine | Carboxylic acids and derivatives | Plasma | HPLC-QTrap-MS/MS | ↑/↑ | √ | T2DN | (14) |
| 279 | Deoxycholic acid | Steroids and steroid derivatives | Urine | UPLC-Q-TOF/MS | ↑ | - | T2DN | (64) |
| 293 | L-Carnitine | Organonitrogen compounds | Serum | LC-MS & GC-MS | ↓ | - | T2DN | (23) |
|  |  |  | Blood | HPLC-MRM | ↑ | √ | T2DN | (37) |
|  |  |  | Urine | UPLC-Q-TOF/MS | ↓ | - | T2DN | (64) |
| 332 | Glycolic acid | Hydroxy acids and derivatives | Urine | GC-MS | - | - | T2DN | (72) |
|  |  |  | Urine | GC-MS | ↓ | - | T2DN | (59) |
|  |  |  | Urine | GC-TOF/MS | ↓ | - | T2DN | (15) |
| 335 | Orotic acid | Diazines | Plasma | HPLC-UV-MS/MS | ↑ | √ | T2DN | (40) |
| 340 | Pseudouridine | Nucleoside and nucleotide analogues | Plasma | UPLC-MS/MS  GC-MS | - | - | T2DN | (81) |
| 342 | Hippuric Acid | Benzene and substituted derivatives | Blood | HPLC-MS | ↓ | - | T2DN | (64) |
|  |  |  | Urine | GC-TOF/MS | ↑ | - | T2DN | (15) |
| 343 | Hypoxanthine | Imidazopyrimidines | Plasma | HPLC-UV-MS/MS | ↑ | √ | T2DN | (40) |
|  |  |  | Urine | UPLC-Q-TOF/MS | - | - | T2DN | (38) |
| 345 | Diacylglycerol C36:1 | Glycerolipids | Serum | LC-MS & GC-MS | ↓ | - | T2DN | (23) |
| 347 | 5-Hydroxyindoleacetic acid | Indoles and derivatives | Urine | UPLC-Q-TOF/MS | ↑ | - | T2DN | (64) |
| 363 | Succinic acid | Carboxylic acids and derivatives | Urine | GC-TOF/MS | ↓ | - | T2DN | (15) |
| 375 | 2-Hydroxybutyric acid | Hydroxy acids and derivatives | Urine | GC-MS | - | √ | T2DN | (49) |
| 390 | Stearic acid | Fatty acyls | Urine | GC-TOF/MS | ↑ | - | T2DN | (15) |
| 408 | Methionine | Carboxylic acids and derivatives | Plasma | HPLC-QTrap-MS/MS | ↑/↑ | √ | T2DN | (14) |
|  |  |  | Blood | HPLC-MRM | ↓ | √ | T2DN | (37) |
| 428 | Erythritol | Saccharides and derivatives | Urine | GC-MS | - | - | T2DN | (72) |
| 456 | Inosine | Purine nucleosides | Plasma | HPLC-UV-MS/MS | ↑ | √ | T2DN | (40) |
| 517 | 3-Hydroxybutanoic acid | Organic acid | Urine | GC-MS | - | - | T2DN | (49) |
| 518 | 3,4-Dihydroxybutanoic acid | Organic acid | Urine | GC-MS | - | - | T2DN | (49) |
| 519 | Acylcoenzyme A | NA | Serum | LC-MS & GC-MS | ↓ | - | T2DN | (23) |
| 520 | PC(9:0/0:0) | Phosphatidylcholine | Serum | LC-MS & GC-MS | ↓ | - | T2DN | (23) |
| 521 | Phosphoric acid | Inorganic acid | Urine | LC-MS & GC-MS | - | - | T2DN | (72) |
| 522 | 2,3,4-Trihydroxybutyric acid | Organic acid | Urine | LC-MS & GC-MS | - | - | T2DN | (72) |
| 523 | 2,3-Dihydroxy-propionic acid | Organic acid | Urine | LC-MS & GC-MS | - | - | T2DN | (72) |
| 524 | D-Ribofuranose | Monosaccharide | Urine | LC-MS & GC-MS | - | - | T2DN | (72) |
| 525 | Xylitol | Five-carbon sugar alcohol | Urine | LC-MS & GC-MS | - | - | T2DN | (72) |
| 526 | 2,3,4,5-Tetrahydroxy-pentanal oxime | NA | Urine | LC-MS & GC-MS | - | - | T2DN | (72) |
| 527 | Glucitol | Saccharides and derivatives | Urine | LC-MS & GC-MS | - | - | T2DN | (72) |
| 528 | Glucosone | Saccharides and derivatives | Urine | LC-MS & GC-MS | - | - | T2DN | (72) |
| 529 | Glucose acid | Saccharides and derivatives | Urine | LC-MS & GC-MS | - | - | T2DN | (72) |
| 530 | 3-Hydroxy isovalerate | Salts | Urine | GC-MS | ↓ | - | T2DN | (59) |
| 531 | 2-Ethyl 3-OH propionate | Esters and esters derivatives | Urine | GC-MS | ↓ | - | T2DN | (59) |
| 532 | Uridine | Pyrimidine nucleosides | Urine | GC-MS | ↓ | - | T2DN | (59) |
|  |  |  | Plasma | HPLC-UV-MS/MS | ↓ | √ | T2DN | (40) |
| 533 | 3-Hydroxy isobutyrate | Organic acid | Urine | GC-MS | ↓ | - | T2DN | (59) |
| 534 | 3-Methyl adipic acid | Tricarboxylic acids and derivatives | Urine | GC-MS | ↓ | - | T2DN | (59) |
| 535 | Tiglylglycine | Acyl glycine | Urine | GC-MS | ↓ | - | T2DN | (59) |
|  |  |  | Urine | UPLC-Q-TOF/MS | ↓ | - | T2DN | (64) |
| 536 | 3-Methyl-crotonyl glycine | Amino acids and derivatives | Urine | GC-MS | ↓ | - | T2DN | (59) |
| 537 | 2-Methyl Acetoacetate | Menthane monoterpenoids | Urine | GC-MS | ↓ | - | T2DN | (59) |
| 538 | Homovanillic acid | Methoxyphenols | Urine | GC-MS | ↓ | - | T2DN | (59) |
| 539 | 3-Hydroxy propionate |  | Urine | GC-MS | ↓ | - | T2DN | (59) |
| 540 | 8-Hydroxy-7-methylguanine | Methylated nucleoside | Urine | UPLC-Q-TOF/MS | ↑ | - | T2DN | (64) |
| 541 | Nutriacholic acid | Bile acid | Urine | UPLC-Q-TOF/MS | ↑ | - | T2DN | (64) |
| 542 | 3-Hydroxyhippuric acid | Acyl glycine | Urine | UPLC-Q-TOF/MS | ↓ | - | T2DN | (64) |
| 543 | Indole-3-carboxylic acid | Indolecarboxylic acids and derivatives | Urine | UPLC-Q-TOF/MS | ↓ | - | T2DN | (64) |
| 544 | Deoxyadenosine | Derivative of the nucleoside adenosine | Urine | UPLC-Q-TOF/MS | ↓ | - | T2DN | (64) |
| 545 | Indolelactic acid | Organic acid | Urine | UPLC-Q-TOF/MS | ↓ | - | T2DN | (64) |
| 546 | 3,4,5-Trihydroxypentanoic acid | Organic acid | Plasma | GC-MS | - | - | T2DN | (67) |
| 547 | 3-Bromo-1-propanol | Alpha-haloketones | Plasma | GC-MS | - | - | T2DN | (67) |
| 548 | Heptadecanoic acid | Fatty acid | Plasma | GC-MS | - | - | T2DN | (67) |
| 549 | Norvaline | Non-proteinogenic branched-chain amino acid | Plasma | GC-MS | - | - | T2DN | (67) |
| 550 | Monomephyl phosphate | 1,2-diacylglycerol-3-phosphates | Plasma | GC-MS | - | - | T2DN | (67) |
| 551 | Propylene glycol | 1,2-propanediol | Plasma | GC-MS | - | - | T2DN | (67) |
| 552 | Octadecanol | Fatty alcohol | Plasma | GC-MS | - | - | T2DN | (67) |
| 553 | Galactofuranoside | Psoralens | Plasma | GC-MS | - | - | T2DN | (67) |
| 554 | Succinyl-CoA | NA | Serum | UPLC-QE-Orbitrap | ↑ | - | T2DN | (79) |
| 555 | Dihydroxyacetone phosphate | Monosaccharide phosphates | Serum | UPLC-QE-Orbitrap | ↑ | - | T2DN | (79) |
| 556 | Oxaloacetate | Short-chain keto acids and derivatives | Serum | UPLC-QE-Orbitrap | ↑ | - | T2DN | (79) |
| 557 | N1-methylguanosine | Methylated nucleoside | Urine | UPLC-Q-TOF/MS | - | - | T2DN | (38) |
| 558 | 7-Methyluric acid | Xanthines | Urine | UPLC-Q-TOF/MS | - | - | T2DN | (38) |
| 559 | Guanosine | Purine nucleosides | Urine | UPLC-Q-TOF/MS | - | - | T2DN | (38) |
| 560 | Guanine | Nucleobases | Urine | UPLC-Q-TOF/MS | - | - | T2DN | (38) |
| 561 | Aspartate | Amino acids and derivatives | Urine | UPLC-Q-TOF/MS | - | - | T2DN | (38) |
| 562 | Tagatose | natural hexoketose | Urine | GC-TOF/MS | ↓ | - | T2DN | (15) |
| 563 | Uracil | Pyrimidones | Urine | GC-TOF/MS | ↓ | - | T2DN | (15) |
| 564 | Cytidine | Nucleoside | Urine | GC-TOF/MS | ↓ | - | T2DN | (15) |
|  |  |  | Plamsa | UHPLC-UV-MS/MS | ↑ | √ | T2DN | (40) |
| 565 | Ethanolamine | 1,2-aminoalcohols | Urine | GC-TOF/MS | ↓ | - | T2DN | (15) |
| 566 | Isocitric acid | Tricarboxylic acids and derivatives | Urine | GC-TOF/MS | ↓ | - | T2DN | (15) |
| 567 | 4-Hydroxybutyrate | Salts | Urine | GC-TOF/MS | ↓ | - | T2DN | (15) |
| 568 | Threonic acid | Sugar acids and derivatives | Urine | GC-TOF/MS | ↓ | - | T2DN | (15) |
| 569 | Hydroxylamine | Hydroxylamine | Urine | GC-TOF/MS | ↑ | - | T2DN | (15) |
| 570 | 3-Hexenedioic acid | Medium-chain fatty acids | Urine | GC-TOF/MS | ↑ | - | T2DN | (15) |
| 571 | 2-Deoxyerythritol | Tetrahydropyridines | Urine | GC-TOF/MS | ↓ | - | T2DN | (15) |
| 572 | Dihydrosphingosine | Ceramide | Serum | UPLC-oaTOF-MS | ↓ | - | T2DN | (16) |
| 573 | Steric acid | Aromatic monoterpenoids | Plasma | UHPLC-Q-Exactive/MS | ↓ | - | T2DN | (16) |
| 574 | Palmitoleic acid | Unsaturated fatty acid | Plasma | UHPLC-Q-Exactive/MS | ↓ | - | T2DN | (16) |
| 575 | Thymine | Hydroxypyrimidines | Plasma | HPLC-UV-MS/MS | ↑ | √ | T2DN | (40) |
| 576 | Deoxyuridine | Pyrimidine 2'-deoxyribonucleosides | Plasma | HPLC-UV-MS/MS | ↓ | √ | T2DN | (40) |
| 577 | Adenine | Purine base | Plasma | HPLC-UV-MS/MS | ↑ | √ | T2DN | (40) |
| 578 | Thymidine | Pyrimidine 2'-deoxyribonucleosides | Plasma | HPLC-UV-MS/MS | ↑ | √ | T2DN | (40) |
| 579 | Adenosine | Nucleoside | Plasma | HPLC-UV-MS/MS | ↑ | √ | T2DN | (40) |
| 580 | Butenoylcarnitine | Fatty ester lipid | Urine/Plasma | HPLC-FIA-MS/MS | ↓ | √ | T2DN | (43) |
| 581 | C-glycosyltryptophan | Indolyl carboxylic acids and derivatives | Plasma | UPLC-MS/MS & GC-MS | - | - | T2DN | (81) |
| 582 | N-acetylthreonine | N-acyl-L-alpha-amino acids | Plasma | UPLC-MS/MS & GC-MS | - | - | T2DN | (81) |
| 583 | PC(16:0/18:2) | Phosphatidylcholine | Plasma | UPLC-MS/MS & GC-MS | ↑ | √ | T2DN | (22) |
| 584 | PC(16:0/18:0) | Phosphatidylcholine | Plasma | UPLC-MS/MS & GC-MS | ↓ | √ | T2DN | (22) |
| 585 | PC(18:0/20:4) | Phosphatidylcholine | Plasma | UPLC-MS/MS & GC-MS | ↓ | √ | T2DN | (22) |
| 586 | PE(16:0/18:1) | Phosphatidylethanolamine | Plasma | UPLC-MS/MS & GC-MS | ↑ | √ | T2DN | (22) |
| 587 | PE(16:0/20:4) | Phosphatidylethanolamine | Plasma | UPLC-MS/MS & GC-MS | ↑ | √ | T2DN | (22) |
| 588 | PE(18:0/20:4) | Phosphatidylethanolamine | Plasma | UPLC-MS/MS & GC-MS | ↑ | √ | T2DN | (22) |
| 589 | PG(18:0/18:2) | Phosphatidylglycerol | Plasma | UPLC-MS/MS & GC-MS | ↑ | √ | T2DN | (22) |

**Reference**

1. Xiao Ji ZL, Ma Mingkun, Yan Weili, Liu Na, Liu Shuye. Serum Metabonomics in Patients with Abnormal Glucose Metabolism. *Chinese Journal of Clinical Laboratory Science* (2014) 32(012):909-11.

2. Cobb J, Eckhart A, Motsinger-Reif A, Carr B, Groop L, Ferrannini E. Α-Hydroxybutyric Acid Is a Selective Metabolite Biomarker of Impaired Glucose Tolerance. *Diabetes Care* (2016).

3. Tulipani S, Palau-Rodriguez M, Alonso AM, Cardona F, Marco-Ramell A, Zonja B, et al. Biomarkers of Morbid Obesity and Prediabetes by Metabolomic Profiling of Human Discordant Phenotypes. *Clinica Chimica Acta* (2016).

4. Wang-Sattler R, Yu Z, Herder C, Messias AC, Illig T. Novel Biomarkers for Pre-Diabetes Identified by Metabolomics. *Molecular Systems Biology* (2012) 8(1):615.

5. Gar C, Rottenkolber M, Prehn C, Adamski J, Seissler J, Lechner A. Serum and Plasma Amino Acids as Markers of Prediabetes, Insulin Resistance, and Incident Diabetes. *Critical reviews in clinical laboratory sciences* (2018) 55(1):21-32. doi: 10.1080/10408363.2017.1414143.

6. Andersson-Hall U, Gustavsson C, Pedersen A, Malmodin D, Joelsson L, Holmäng A. Higher Concentrations of Bcaas and 3-Hib Are Associated with Insulin Resistance in the Transition from Gestational Diabetes to Type 2 Diabetes. *Journal of diabetes research* (2018) 2018:4207067. doi: 10.1155/2018/4207067.

7. Hai-zhen GYZPLJL. Serum Metabonomics Study on Type 2 Diabetes by Using Gas Chromatography/Mass Spectrometry. *Journal of Shanxi Datong University(Natural Science Edition)* (2017) (33):33-5.

8. Merino J, Leong A, Liu CT, Porneala B, Walford GA, von Grotthuss M, et al. Metabolomics Insights into Early Type 2 Diabetes Pathogenesis and Detection in Individuals with Normal Fasting Glucose. *Diabetologia* (2018) 61(6):1315-24. Epub 2018/04/08. doi: 10.1007/s00125-018-4599-x.

9. Lu Y, Wang Y, Ong CN, Subramaniam T, Choi HW, Yuan JM, et al. Metabolic Signatures and Risk of Type 2 Diabetes in a Chinese Population: An Untargeted Metabolomics Study Using Both Lc-Ms and Gc-Ms. *Diabetologia* (2016) 59(11):2349-59. Epub 2016/08/16. doi: 10.1007/s00125-016-4069-2.

10. Mihalik SJ, Michaliszyn SF, de las Heras J, Bacha F, Lee S, Chace DH, et al. Metabolomic Profiling of Fatty Acid and Amino Acid Metabolism in Youth with Obesity and Type 2 Diabetes: Evidence for Enhanced Mitochondrial Oxidation. *Diabetes Care* (2012) 35(3):605-11. Epub 2012/01/24. doi: 10.2337/DC11-1577.

11. Yu D, Moore SC, Matthews CE, Xiang YB, Zhang X, Gao YT, et al. Plasma Metabolomic Profiles in Association with Type 2 Diabetes Risk and Prevalence in Chinese Adults. *Metabolomics* (2016) 12. Epub 2016/11/15. doi: 10.1007/s11306-015-0890-8.

12. Patel SG, Hsu JW, Jahoor F, Coraza I, Bain JR, Stevens RD, et al. Pathogenesis of a(-)Beta(+) Ketosis-Prone Diabetes. *Diabetes* (2013) 62(3):912-22. Epub 2012/11/20. doi: 10.2337/db12-0624.

13. GOU Xiao-jun ZS-x, LI Guang-ping, ZHANG Cheng, CHENG Wen, CHEN Fei, WANG Hua, ZHANG Lu, CHEN Yue. Urinary Metabolomics Study in Patients with Diabetic Peripheral Neuropathy. *Chinese Journal of Hospital Pharmacy* (2019) 039(024):2512-9.

14. Zongmiao J. Preliminary Screening of Metabolic Markers Reiated to Diabetic Nephropathy (2019).

15. Li L, Wang C, Yang H, Liu S, Lu Y, Fu P, et al. Metabolomics Reveal Mitochondrial and Fatty Acid Metabolism Disorders That Contribute to the Development of Dkd in T2dm Patients. *Mol Biosyst* (2017) 13(11):2392-400. Epub 2017/09/29. doi: 10.1039/c7mb00167c.

16. Zhang J, Yan L, Chen W, Lin L, Song X, Yan X, et al. Metabonomics Research of Diabetic Nephropathy and Type 2 Diabetes Mellitus Based on Uplc-Oatof-Ms System. *Anal Chim Acta* (2009) 650(1):16-22. Epub 2009/09/02. doi: 10.1016/j.aca.2009.02.027.

17. Jun G, Aguilar D, Evans C, Burant C, Hanis C. Metabolomic Profiles Associated with Subtypes of Prediabetes among Mexican Americans in Starr County, Texas, USA. *Diabetologia* (2020) 63(2):287-95. doi: 10.1007/s00125-019-05031-4.

18. Ha CY, Kim JY, Paik JK, Kim OY, Paik YH, Lee EJ, et al. The Association of Specific Metabolites of Lipid Metabolism with Markers of Oxidative Stress, Inflammation and Arterial Stiffness in Men with Newly Diagnosed Type 2 Diabetes. *Clin Endocrinol (Oxf)* (2012) 76(5):674-82. Epub 2011/10/01. doi: 10.1111/j.1365-2265.2011.04244.x.

19. Kim Minjoo SG, Kang Miso. Replacing Carbohydrate with Protein and Fat in Prediabetes or Type-2 Diabetes: Greater Effect on Metabolites in Pbmc Than Plasma. *Nutrition & Metabolism* (2016) 13(1):3.

20. Kumar AA, Satheesh G, Vijayakumar G, Chandran M, Jaleel A. Postprandial Metabolism Is Impaired in Overweight Normoglycemic Young Adults without Family History of Diabetes. *entific Reports* (2020) 10(1).

21. Drogan D, Dunn WB, Lin W, Buijsse B, Schulze MB, Langenberg C, et al. Untargeted Metabolic Profiling Identifies Altered Serum Metabolites of Type 2 Diabetes Mellitus in a Prospective, Nested Case Control Study. *Clin Chem* (2015) 61(3):487-97. Epub 2014/12/20. doi: 10.1373/clinchem.2014.228965.

22. Zhu C, Liang QL, Hu P, Wang YM, Luo GA. Phospholipidomic Identification of Potential Plasma Biomarkers Associated with Type 2 Diabetes Mellitus and Diabetic Nephropathy. *Talanta* (2011) 85(4):1711-20. Epub 2011/08/30. doi: 10.1016/j.talanta.2011.05.036.

23. Xufang W, Mengjie L, Yongchun G, Weisong Q, Jiye A, Jinhua H, et al. Serum and Urinary Metabolomic Analysis in Patients with Diabetic Nephropathy. *Chinese Journal of Nephrology,Dialysis & Transplantation* (2012) (3):201-9.

24. Meihua Y. Research on Mechanism of Female Impaired Glucose Regulation (2012).

25. Hongfu Z. The Applications of Liquid Chromatography Combined with Mass Spectrometry in Impaired Glucose Tolerance and Liver Cirrhosis: East China University of Science and Technology (2011).

26. Kujala UM, Markku P, Laine MK, Jaakko K, Heinonen OJ, Jouko S, et al. Branched-Chain Amino Acid Levels Are Related with Surrogates of Disturbed Lipid Metabolism among Older Men. *Frontiers in Medicine* (2016) 3:57-.

27. Lin H, Cheng M, Lo C, Lin G, Lin S, Yeh J, et al. H Nuclear Magnetic Resonance (Nmr)-Based Cerebrospinal Fluid and Plasma Metabolomic Analysis in Type 2 Diabetic Patients and Risk Prediction for Diabetic Microangiopathy. *Journal of clinical medicine* (2019) 8(6). doi: 10.3390/jcm8060874.

28. Doorn MV, Vogels J, Tas A, Hoogdalem EJV, Burggraaf J, Cohen A, et al. Evaluation of Metabolite Profiles as Biomarkers for the Pharmacological Effects of Thiazolidinediones in Type 2 Diabetes Mellitus Patients and Healthy Volunteers. *British Journal of Clinical Pharmacology* (2010) 63(5):562-74.

29. Liu X, Gao X, Zhang R, Liu Z, Shen N, Di Y, et al. Discovery and Comparison of Serum Biomarkers for Diabetes Mellitus and Metabolic Syndrome Based on Uplc-Q-Tof/Ms. *Clin Biochem* (2020) 82:40-50. Epub 2020/03/21. doi: 10.1016/j.clinbiochem.2020.03.007.

30. Chailurkit LO, Paiyabhroma N, Sritara P, Vathesatogkit P, Yamwong S, Thonmung N, et al. Independent and Opposite Associations between Branched-Chain Amino Acids and Lysophosphatidylcholines with Incident Diabetes in Thais. *Metabolites* (2020) 10(2). Epub 2020/02/26. doi: 10.3390/metabo10020076.

31. Zhang N, Geng F, Hu ZH, Liu B, Li LJ. Preliminary Study of Urine Metabolism in Type Two Diabetic Patients Based on Gc-Ms. *American Journal of Translational Research* (2016) 8(7):2889.

32. Kaur P, Rizk N, Ibrahim S, Luo Y, Younes N, Perry B, et al. Quantitative Metabolomic and Lipidomic Profiling Reveals Aberrant Amino Acid Metabolism in Type 2 Diabetes. *Mol Biosyst* (2013) 9(2):307-17. Epub 2012/12/19. doi: 10.1039/c2mb25384d.

33. Menni C, Fauman E, Erte I, Perry J, Kastenmüller G, Shin S, et al. Biomarkers for Type 2 Diabetes and Impaired Fasting Glucose Using a Nontargeted Metabolomics Approach. *Diabetes* (2013) 62(12):4270-6. doi: 10.2337/db13-0570.

34. Liao X, Liu B, Qu H, Zhang L, Lu Y, Xu Y, et al. A High Level of Circulating Valine Is a Biomarker for Type 2 Diabetes and Associated with the Hypoglycemic Effect of Sitagliptin. *Mediators Inflamm* (2019) 2019:8247019. Epub 2019/12/13. doi: 10.1155/2019/8247019.

35. Rawat A, Misra G, Saxena M, Tripathi S, Dubey D, Saxena S, et al. (1)H Nmr Based Serum Metabolic Profiling Reveals Differentiating Biomarkers in Patients with Diabetes and Diabetes-Related Complication. *Diabetes Metab Syndr* (2019) 13(1):290-8. Epub 2019/01/16. doi: 10.1016/j.dsx.2018.09.009.

36. Rebholz CM, Yu B, Zheng Z, Chang P, Tin A, Kottgen A, et al. Serum Metabolomic Profile of Incident Diabetes. *Diabetologia* (2018) 61(5):1046-54. Epub 2018/03/21. doi: 10.1007/s00125-018-4573-7.

37. Ibarra-Gonzalez I, Cruz-Bautista I, Bello-Chavolla OY, Vela-Amieva M, Pallares-Mendez R, Ruiz de Santiago YND, et al. Optimization of Kidney Dysfunction Prediction in Diabetic Kidney Disease Using Targeted Metabolomics. *Acta Diabetol* (2018) 55(11):1151-61. Epub 2018/09/03. doi: 10.1007/s00592-018-1213-0.

38. Chen CJ, Liao WL, Chang CT, Lin YN, Tsai FJ. Identification of Urinary Metabolite Biomarkers of Type 2 Diabetes Nephropathy Using an Untargeted Metabolomic Approach. *J Proteome Res* (2018) 17(11):3997-4007. Epub 2018/09/29. doi: 10.1021/acs.jproteome.8b00644.

39. Savolainen O, Lind M, Bergström G, Fagerberg B, Sandberg A, Ross A. Biomarkers of Food Intake and Nutrient Status Are Associated with Glucose Tolerance Status and Development of Type 2 Diabetes in Older Swedish Women. *The American journal of clinical nutrition* (2017) 106(5):1302-10. doi: 10.3945/ajcn.117.152850.

40. Xia JF, Liang QL, Liang XP, Wang YM, Hu P, Li P, et al. Ultraviolet and Tandem Mass Spectrometry for Simultaneous Quantification of 21 Pivotal Metabolites in Plasma from Patients with Diabetic Nephropathy. *J Chromatogr B Analyt Technol Biomed Life Sci* (2009) 877(20-21):1930-6. Epub 2009/06/09. doi: 10.1016/j.jchromb.2009.05.047.

41. Al-Sulaiti H, Diboun I, Agha MV, Mohamed FFS, Atkin S, Domling AS, et al. Metabolic Signature of Obesity-Associated Insulin Resistance and Type 2 Diabetes. *J Transl Med* (2019) 17(1):348. Epub 2019/10/24. doi: 10.1186/s12967-019-2096-8.

42. Jin H, Zhu B, Liu X, Jin J, Zou H. Metabolic Characterization of Diabetic Retinopathy: An (1)H-Nmr-Based Metabolomic Approach Using Human Aqueous Humor. *J Pharm Biomed Anal* (2019) 174:414-21. Epub 2019/06/19. doi: 10.1016/j.jpba.2019.06.013.

43. Pena MJ, Lambers Heerspink HJ, Hellemons ME, Friedrich T, Dallmann G, Lajer M, et al. Urine and Plasma Metabolites Predict the Development of Diabetic Nephropathy in Individuals with Type 2 Diabetes Mellitus. *Diabet Med* (2014) 31(9):1138-47. Epub 2014/03/26. doi: 10.1111/dme.12447.

44. Wei H, Pasman W, Rubingh C, Wopereis S, Tienstra M, Schroen J, et al. Urine Metabolomics Combined with the Personalized Diagnosis Guided by Chinese Medicine Reveals Subtypes of Pre-Diabetes. *Molecular Biosystems* (2012) 8(5):1482-91.

45. Inken P, Erik P, Sandra G-M, Henning W, Matthias M, Tanja W, et al. A New Metabolomic Signature in Type-2 Diabetes Mellitus and Its Pathophysiology. *Plos One* (2014) 9(1):e85082.

46. Carter TC, Rein D, Padberg I, Peter E, Rennefahrt U, David DE, et al. Validation of a Metabolite Panel for Early Diagnosis of Type 2 Diabetes. *Metabolism Clinical & Experimental* (2016):1399-408.

47. Mack CI, Ferrario PG, Weinert CH, Egert B, Hoefle AS, Lee YM, et al. Exploring the Diversity of Sugar Compounds in Healthy, Prediabetic, and Diabetic Volunteers. *Mol Nutr Food Res* (2020) 64(9):e1901190. Epub 2020/03/15. doi: 10.1002/mnfr.201901190.

48. Suhre K, Meisinger C, Doring A, Altmaier E, Belcredi P, Gieger C, et al. Metabolic Footprint of Diabetes: A Multiplatform Metabolomics Study in an Epidemiological Setting. *PLoS One* (2010) 5(11):e13953. Epub 2010/11/19. doi: 10.1371/journal.pone.0013953.

49. Yuhua M. Urine Metababonomics Study on the Biochemical Profiles of Diabetic Nephropathy: Xinjiang University (2014).

50. YU Huan LQ-l, LI Li. Effects of Tianqijiangtang Capsule on Lipid Metabolomics in Impaired Glucose Tolerance (Igt) Volunteers. *CHINESE JOURNAL OF DIABETES* (2011). doi: 10.3969/j.issn.1006-6187.2011.05.008.

51. Zhengzhen LBJMZNLCWYZXZYW. Effects of Aerobic Exercise on Plasma Metabolites in Prediabetes Subjects. *Chinese Journal of Sports Medicine* (2018) 37(4):301-8.

52. Lokhov PG, Trifonova OP, Maslov DL, Balashova EE, Archakov AI, Shestakova EA, et al. Diagnosing Impaired Glucose Tolerance Using Direct Infusion Mass Spectrometry of Blood Plasma. *Plos One* (2014) 9(9):e105343.

53. Devi S, Nongkhlaw B, Limesh M, Pasanna RM, Thomas T, Kuriyan R, et al. Acyl Ethanolamides in Diabetes and Diabetic Nephropathy: Novel Targets from Untargeted Plasma Metabolomic Profiles of South Asian Indian Men. *Sci Rep* (2019) 9(1):18117. Epub 2019/12/04. doi: 10.1038/s41598-019-54584-2.

54. Xiao-li M, Lei M, Xin-xia L, Lin-lin L, Ye W, Xin-min M. Urine Metabonomics Study on Diabetes Patients by Uplc /Q-Tof Ms. *Journal of Instrumental Analysis* (2014) 33(006):621-7.

55. Savolainen O, Fagerberg B, Vendelbo Lind M, Sandberg AS, Ross AB, Bergstrom G. Biomarkers for Predicting Type 2 Diabetes Development-Can Metabolomics Improve on Existing Biomarkers? *PLoS One* (2017) 12(7):e0177738. Epub 2017/07/12. doi: 10.1371/journal.pone.0177738.

56. de Mello VD, Paananen J, Lindstrom J, Lankinen MA, Shi L, Kuusisto J, et al. Indolepropionic Acid and Novel Lipid Metabolites Are Associated with a Lower Risk of Type 2 Diabetes in the Finnish Diabetes Prevention Study. *Sci Rep* (2017) 7:46337. Epub 2017/04/12. doi: 10.1038/srep46337.

57. Zhao S, Zheng H, Lu X, Liu Y, Su B, Xu G. [Metabonomics and Phospholipid Metabolic Profiling of Abnormal Glucose Metabolism Based on High Performance Liquid Chromatography-Electrospray Mass Spectrometry]. *Se pu = Chinese journal of chromatography* (2011) 29(4):307-13. doi: 10.3724/sp.j.1123.2011.00307.

58. Liu R, Zhao J, Guo J, Liu X, Yu J, Wang H, et al. Postprandial Metabolomics: Gc-Ms Analysis Reveals Differences in Organic Acid Profiles of Impaired Fasting Glucose Individuals in Response to Highland Barley Loads. *Food & Function* (2019).

59. Sharma K, Karl B, Mathew AV, Gangoiti JA, Wassel CL, Saito R, et al. Metabolomics Reveals Signature of Mitochondrial Dysfunction in Diabetic Kidney Disease. *J Am Soc Nephrol* (2013) 24(11):1901-12. Epub 2013/08/21. doi: 10.1681/ASN.2013020126.

60. Chou J, Liu R, Yu J, Liu X, Zhao X, Li Y, et al. Fasting Serum Alphahydroxybutyrate and Pyroglutamic Acid as Important Metabolites for Detecting Isolated Post-Challenge Diabetes Based on Organic Acid Profiles. *J Chromatogr B Analyt Technol Biomed Life Sci* (2018) 1100-1101:6-16. Epub 2018/09/30. doi: 10.1016/j.jchromb.2018.09.004.

61. Liu J, Semiz S, van der Lee SJ, van der Spek A, Verhoeven A, van Klinken JB, et al. Metabolomics Based Markers Predict Type 2 Diabetes in a 14-Year Follow-up Study. *Metabolomics* (2017) 13(9):104. Epub 2017/08/15. doi: 10.1007/s11306-017-1239-2.

62. Lo CJ, Tang HY, Huang CY, Lin CM, Ho HY, Shiao MS, et al. Metabolic Signature Differentiated Diabetes Mellitus from Lipid Disorder in Elderly Taiwanese. *J Clin Med* (2018) 8(1). Epub 2018/12/24. doi: 10.3390/jcm8010013.

63. Wang TJ, Ngo D, Psychogios N, Dejam A, Gerszten RE. 2-Aminoadipic Acid Is a Biomarker for Diabetes Risk. *Journal of Clinical Investigation* (2013) 123(10):4309-17.

64. Liu Y, Chen X, Liu Y, Chen T, Zhang Q, Zhang H, et al. Metabolomic Study of the Protective Effect of Gandi Capsule for Diabetic Nephropathy. *Chem Biol Interact* (2019) 314:108815. Epub 2019/09/10. doi: 10.1016/j.cbi.2019.108815.

65. Ju L. Serum Metabonornios of Impaired Giucose Reguiation with Uplciq-Tof Ms: JiLin University (2018).

66. Tam ZY, Ng SP, Tan LQ, Lin CH, Rothenbacher D, Klenk J, et al. Metabolite Profiling in Identifying Metabolic Biomarkers in Older People with Late-Onset Type 2 Diabetes Mellitus. *Sci Rep* (2017) 7(1):4392. Epub 2017/07/01. doi: 10.1038/s41598-017-01735-y.

67. Tavares G, Venturini G, Padilha K, Zatz R, Pereira AC, Thadhani RI, et al. 1,5-Anhydroglucitol Predicts Ckd Progression in Macroalbuminuric Diabetic Kidney Disease: Results from Non-Targeted Metabolomics. *Metabolomics* (2018) 14(4):39. Epub 2019/03/05. doi: 10.1007/s11306-018-1337-9.

68. Li J, Cao YF, Sun XY, Han L, Li SN, Gu WQ, et al. Plasma Tyrosine and Its Interaction with Low High-Density Lipoprotein Cholesterol and the Risk of Type 2 Diabetes Mellitus in Chinese. *J Diabetes Investig* (2019) 10(2):491-8. Epub 2018/07/13. doi: 10.1111/jdi.12898.

69. Cao YF, Li J, Zhang Z, Liu J, Sun XY, Feng XF, et al. Plasma Levels of Amino Acids Related to Urea Cycle and Risk of Type 2 Diabetes Mellitus in Chinese Adults. *Front Endocrinol (Lausanne)* (2019) 10:50. Epub 2019/03/06. doi: 10.3389/fendo.2019.00050.

70. Chen L, Cheng CY, Choi H, Ikram MK, Sabanayagam C, Tan GS, et al. Plasma Metabonomic Profiling of Diabetic Retinopathy. *Diabetes* (2016) 65(4):1099-108. Epub 2016/01/30. doi: 10.2337/db15-0661.

71. Wang H, Zhang H, Yao L, Cui L, Zhang L, Gao B, et al. Serum Metabolic Profiling of Type 2 Diabetes Mellitus in Chinese Adults Using an Untargeted Gc/Tofms. *Clin Chim Acta* (2018) 477:39-47. Epub 2017/12/05. doi: 10.1016/j.cca.2017.11.036.

72. Yang L. Metabonomics Study on the Biochemical Profiles of Diabetic Nephropathy: Xin (2011).

73. Park JE, Jeong GH, Lee IK, Yoon YR, Liu KH, Gu N, et al. A Pharmacometabolomic Approach to Predict Response to Metformin in Early-Phase Type 2 Diabetes Mellitus Patients. *Molecules* (2018) 23(7). Epub 2018/07/04. doi: 10.3390/molecules23071579.

74. Jun FHLXYWZLZDW. Screening of Urinary Biomarkers in Patients with Type 2 Diabetes Mellitus. *Journal of Hygiene Research* (2013) 42(006):907-14.

75. Lucio M, Fekete A, Weigert C, Wagele B, Zhao X, Chen J, et al. Insulin Sensitivity Is Reflected by Characteristic Metabolic Fingerprints--a Fourier Transform Mass Spectrometric Non-Targeted Metabolomics Approach. *PLoS One* (2010) 5(10):e13317. Epub 2010/10/27. doi: 10.1371/journal.pone.0013317.

76. Vangipurapu J, Silva LF, Kuulasmaa T, Smith U, Laakso M. Microbiota-Related Metabolites and the Risk of Type 2 Diabetes. *Diabetes Care* (2020) 43(6):dc192533.

77. Mook-Kanamori DO, El-Din SMM, Takiddin AH, Hala AH, Al-Mahmoud KAS, Amina AO, et al. 1,5-Anhydroglucitol in Saliva Is a Noninvasive Marker of Short-Term Glycemic Control. *J Clin Endocrinol Metab* (3):479-83.

78. Liyan L, Ying L, Cheng W, Rennan F, Changhao S. Free Fatty Acid Metabolic Profile and Biomarkers of Isolated Post-Challenge Diabetes Based on Gc-Ms and Multivariate Statistical Analysis. *Nutrition New sletter* (2012) (1):12-7.

79. Jiang Hong SX, Jia Sha. The Mitochondria-Targeted Metabolic Tubular Injury in Diabetic Kidney Disease. *Cell Physiol Biochem* (2019). doi: 10.1159/000000011.

80. Kailong Y, Xianzhe S, Xin L, Peng G, Guowang X. Assessment of Therapeutic Effect of Losartan on Diabetes Mellitus with Gas Chromatography-Based Metabonomics. *Acta Academiae Medicinae Sinicae* (2007) 29(006):719-24.

81. Solini A, Manca ML, Penno G, Pugliese G, Cobb JE, Ferrannini E. Prediction of Declining Renal Function and Albuminuria in Patients with Type 2 Diabetes by Metabolomics. *J Clin Endocrinol Metab* (2016) 101(2):696-704. Epub 2015/12/20. doi: 10.1210/jc.2015-3345.

82. Lee Y, Pamungkas AD, Medriano CAD, Park J, Hong S, Jee SH, et al. High-Resolution Metabolomics Determines the Mode of Onset of Type 2 Diabetes in a 3-Year Prospective Cohort Study. *Int J Mol Med* (2018) 41(2):1069-77. Epub 2017/12/06. doi: 10.3892/ijmm.2017.3275.

83. Sun L, Liang L, Gao X, Zhang H, Yao P, Hu Y, et al. Early Prediction of Developing Type 2 Diabetes by Plasma Acylcarnitines: A Population-Based Study. *Diabetes Care* (2016) 39(9):1563-70. Epub 2016/07/09. doi: 10.2337/dc16-0232.

84. Jinkui ZXYFLJCXYGXRFJY. Plasma Metabolomic Profiling of Proliferative Diabetic Retinopathy. *Journal of Capital Medical University* (2020).

85. Kunikata H, Ida T, Sato K, Aizawa N, Sawa T, Tawarayama H, et al. Metabolomic Profiling of Reactive Persulfides and Polysulfides in the Aqueous and Vitreous Humors. *Sci Rep* (2017) 7:41984. Epub 2017/02/09. doi: 10.1038/srep41984.
